# The *Oxytricha trifallax* Macronuclear Genome: A Complex Eukaryotic Genome with 16,000 Tiny Chromosomes

**DOI:** 10.1371/journal.pbio.1001473

**Published:** 2013-01-29

**Authors:** Estienne C. Swart, John R. Bracht, Vincent Magrini, Patrick Minx, Xiao Chen, Yi Zhou, Jaspreet S. Khurana, Aaron D. Goldman, Mariusz Nowacki, Klaas Schotanus, Seolkyoung Jung, Robert S. Fulton, Amy Ly, Sean McGrath, Kevin Haub, Jessica L. Wiggins, Donna Storton, John C. Matese, Lance Parsons, Wei-Jen Chang, Michael S. Bowen, Nicholas A. Stover, Thomas A. Jones, Sean R. Eddy, Glenn A. Herrick, Thomas G. Doak, Richard K. Wilson, Elaine R. Mardis, Laura F. Landweber

**Affiliations:** 1 Department of Ecology and Evolutionary Biology, Princeton University, Princeton, New Jersey, United States of America; 2 The Genome Institute, Washington University School of Medicine, St. Louis, Missouri, United States of America; 3 Department of Genetics, Washington University School of Medicine, St. Louis, Missouri, United States of America; 4 Department of Molecular Biology, Princeton University, Princeton, New Jersey, United States of America; 5 Institute of Cell Biology, University of Bern, Bern, Switzerland; 6 Janelia Farm Research Campus, Howard Hughes Medical Institute, Ashburn, Virginia, United States of America; 7 Sequencing Core Facility, Lewis-Sigler Institute for Integrative Genomics, Princeton University, Princeton, New Jersey, United States of America; 8 Bioinformatics Group, Lewis-Sigler Institute for Integrative Genomics, Princeton University, Princeton, New Jersey, United States of America; 9 Department of Biology, Hamilton College, Clinton, New York, United States of America; 10 Biology Department, Bradley University, Peoria, Illinois, United States of America; 11 Biology Department, University of Utah, Salt Lake City, Utah, United States of America; 12 Department of Biology, University of Indiana, Bloomington, Indiana, United States of America; University of California Davis, United States of America

## Abstract

The macronuclear genome of the ciliate *Oxytricha trifallax* displays an extreme and unique eukaryotic genome architecture with extensive genomic variation. During sexual genome development, the expressed, somatic macronuclear genome is whittled down to the genic portion of a small fraction (∼5%) of its precursor “silent” germline micronuclear genome by a process of “unscrambling” and fragmentation. The tiny macronuclear “nanochromosomes” typically encode single, protein-coding genes (a small portion, 10%, encode 2–8 genes), have minimal noncoding regions, and are differentially amplified to an average of ∼2,000 copies. We report the high-quality genome assembly of ∼16,000 complete nanochromosomes (∼50 Mb haploid genome size) that vary from 469 bp to 66 kb long (mean ∼3.2 kb) and encode ∼18,500 genes. Alternative DNA fragmentation processes ∼10% of the nanochromosomes into multiple isoforms that usually encode complete genes. Nucleotide diversity in the macronucleus is very high (SNP heterozygosity is ∼4.0%), suggesting that *Oxytricha trifallax* may have one of the largest known effective population sizes of eukaryotes. Comparison to other ciliates with nonscrambled genomes and long macronuclear chromosomes (on the order of 100 kb) suggests several candidate proteins that could be involved in genome rearrangement, including domesticated MULE and IS1595-like DDE transposases. The assembly of the highly fragmented *Oxytricha* macronuclear genome is the first completed genome with such an unusual architecture. This genome sequence provides tantalizing glimpses into novel molecular biology and evolution. For example, *Oxytricha* maintains tens of millions of telomeres per cell and has also evolved an intriguing expansion of telomere end-binding proteins. In conjunction with the micronuclear genome in progress, the *O. trifalla*x macronuclear genome will provide an invaluable resource for investigating programmed genome rearrangements, complementing studies of rearrangements arising during evolution and disease.

## Abstract 2

With more chromosomes than any other sequenced genome, the macronuclear genome of *Oxytricha trifallax* has a unique and complex architecture, including alternative fragmentation and predominantly single-gene chromosomes.

## Author Summary

The macronuclear genome of the ciliate *Oxytricha trifallax*, contained in its somatic nucleus, has a unique genome architecture. Unlike its diploid germline genome, which is transcriptionally inactive during normal cellular growth, the macronuclear genome is fragmented into at least 16,000 tiny (∼3.2 kb mean length) chromosomes, most of which encode single actively transcribed genes and are differentially amplified to a few thousand copies each. The smallest chromosome is just 469 bp, while the largest is 66 kb and encodes a single enormous protein. We found considerable variation in the genome, including frequent alternative fragmentation patterns, generating chromosome isoforms with shared sequence. We also found limited variation in chromosome amplification levels, though insufficient to explain mRNA transcript level variation. Another remarkable feature of *Oxytricha*'s macronuclear genome is its inordinate fondness for telomeres. In conjunction with its possession of tens of millions of chromosome-ending telomeres per macronucleus, we show that *Oxytricha* has evolved multiple putative telomere-binding proteins. In addition, we identified two new domesticated transposase-like protein classes that we propose may participate in the process of genome rearrangement. The macronuclear genome now provides a crucial resource for ongoing studies of genome rearrangement processes that use *Oxytricha* as an experimental or comparative model.

## Introduction

*Oxytricha trifallax* is a distinctive ciliate [1]—an ancient lineage of protists named for their coats of cilia. Like all ciliates, *Oxytricha* has two types of nuclei: a micronucleus, a germline nucleus that is largely transcriptionally inactive during vegetative growth, and a macronucleus, which is the transcriptionally active somatic nucleus [2]. Compared to the micronucleus, *Oxytricha*'s macronucleus is massively enlarged due to ∼2,000-fold [2] amplification resulting from two rounds of DNA amplification [3] during development.

**Figure 1:**
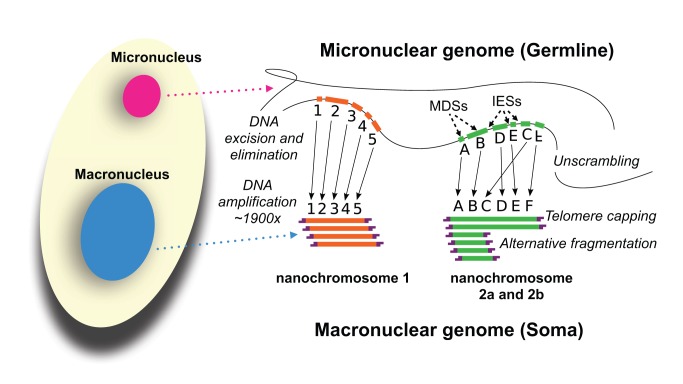
Development of the *Oxytricha* macronuclear genome from the micronuclear genome. During conjugation of *Oxytricha* cells, segments of the micronuclear genome (MDSs) are excised and stitched together to form the nanochromosomes of the new macronuclear genome, and the remainder of the micronuclear genome is eliminated (including the IESs interspersed between MDSs). The old macronuclear genome is also degraded during development. The segments that are stitched together may be either in order (e.g., forming nanochromosome 1, on the left) or out of order or inverted (e.g., forming the two forms of nanochromosome 2), in which case they need to be “unscrambled.” Two rounds of DNA amplification produce nanochromosomes at an average copy number of ∼1,900 [2]. Alternative fragmentation of DNA during nanochromosome development may also occur, irrespective of unscrambling, giving rise to longer (2a) and shorter (2b) nanochromosome isoforms. The mature nanochromosomes are capped on both ends with telomeres.

In the model ciliates *Oxytricha trifallax*, *Tetrahymena thermophila*, and *Paramecium tetraurelia*, varying amounts of micronuclear DNA are deleted (including the “internally eliminated sequences,” or IESs, interspersed between “macronuclear destined sequences,” or MDSs) during conjugation or autogamy (two forms of sexual development) to give rise to the information-rich macronuclear genome (Figure 1). A much larger fraction of the *Oxytricha* micronuclear genome—∼96% of the micronuclear complexity [2]—is eliminated during the macronuclear formation than in the oligohymenophoreans, *Tetrahymena* and *Paramecium* (which both eliminate ∼30% of their micronuclear genomes [4],[5]). The most remarkable difference in macronuclear development between *Oxytricha* and the two oligohymenophoreans is that the micronuclear-encoded MDSs that give rise to the macronuclear chromosomes may be nonsequential, or even in different orientations in the micronuclear genome [6]. Consequently, unlike the oligohymenophoreans, *Oxytricha* needs to unscramble its micronuclear genome during macronuclear development.

**Figure 2:**
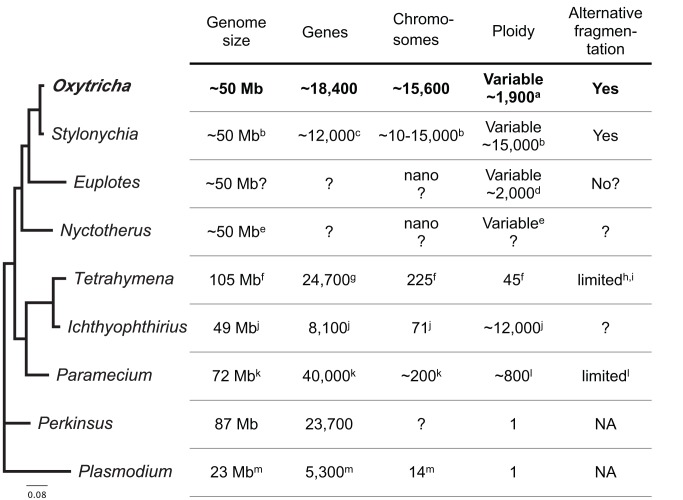
Comparison of key ciliate macronuclear genomes. The phylogeny represents the bootstrap consensus of 100 replicates from PhyML (with the HKY85 substitution model) based on a MUSCLE multiple sequence alignment of 18S rRNA genes from seven ciliates (*Oxytricha trifallax*—FJ545743; *Stylonychia lemnae*—AJJRB310497; *Euplotes crassus*—AJJRB310492; *Nyctotherus ovalis*—AJ222678; *Tetrahymena thermophila*—M10932; *Ichthyophthirius multifiliis*—IMU17354; and *Paramecium tetraurelia*—AB252009) rooted with two other alveolates (*Perkinsus marinus*—X75762 and *Plasmodium falciparum*—NC_004325). All bootstrap values are ≥80, except for the node between *Nyctotherus* and *Oxytricha*/*Stylonychia*/*Euplotes*, which has a boostrap value of 60. *Euplotes* and *Nyctotherus* both have nanochromosomes, like *Oxytricha*. Other than the genome statistics for *Oxytricha trifallax*, which were determined in this study, table statistics were obtained from the following sources: ^a^ - [2], ^b^ - [22],[116], ^c^ - [117], ^d^ - [99], ^e^ - [94], ^f^ - [56] (the number of chromosomes is an estimate), ^g^ -[118], ^h^ - [119], ^i^ - [120], ^j^- [64] (for a single stage of the *Ichthyophthirius* life cycle), ^k^ - [121], ^l^ - [69], ^m^ - [122]. Table statistics for *Perkinsus marinus* are for the current assembly deposited in GenBank (GCA_000006405.1).

**Figure 3:**
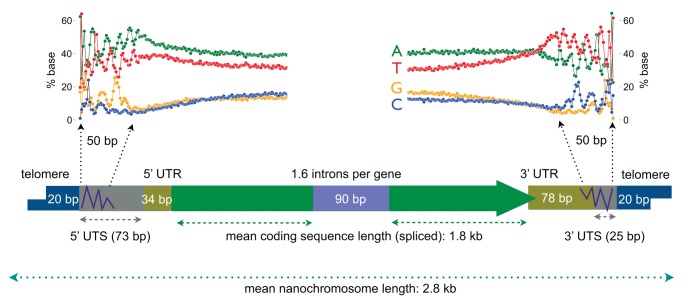
Key features of *Oxytricha* protein-coding nanochromosomes. Representative nanochromosome features are not drawn to scale, but their lengths are indicated. UTR, untranslated region; UTS, untranscribed region. 3′ UTRs and the subtelomeric signal overlap. The subtelomeric base composition bias signal found on either end of the nanochromosome is shown above the nanochromosome diagram.

Two fundamental differences distinguish *Oxytricha*'s macronuclear chromosomes from those of *Tetrahymena* and *Paramecium*: *Oxytricha*'s chromosomes are tiny (“nanochromosomes,” with a mean length ∼3.2 kb reported in this study), each typically encoding just a single gene with a minimal amount of surrounding non-protein-coding DNA [7], and they are differentially amplified (Figure 2 and 3) [8],[9]. In some cases, alternative fragmentation of macronuclear-destined micronuclear DNA produces different nanochromosome isoforms (Figure 2) 10–12, which may be present at very different levels of amplification (differing by as much as 10-fold [13]). Gene expression and nanochromosome copy number may be moderately correlated [14]. Macronuclear chromosomes in all the model ciliates segregate by amitosis during cellular replication (without a mitotic spindle) [15],—a process that may lead to allelic fixation 17–20. In ciliates with nanochromosomes, major fluctuations of nanochromosome copy number [8] may arise, since copy number is unregulated during normal cellular replication [21]. Theoretical models propose that these fluctuations are a cause of senescence in these ciliates [22]. In contrast to the lack of copy number regulation during cellular replication, both genetic [23]–[25] and epigenetic mechanisms [9],[26] may influence chromosome copy number during sexual development in ciliates.

As a consequence of the extensive fragmentation of the *Oxytricha* macronuclear genome, each macronucleus possesses tens of millions of telomeres, an abundance that enabled the first isolations of telomere end-binding proteins [27],[28]. *Oxytricha* also has micronuclear transposons bearing telomeric repeats (C_4_A_4_) that resemble those of nanochromosomes. These telomere-bearing elements, or TBE transposons [29], play an important role in macronuclear genome development [30]. The exact site of telomere addition may vary for some nanochromosome ends [31] and is followed by a roughly 50 bp subtelomeric region of biased base composition with an approximately 10 bp periodicity of the bias (Figure 3) [32],[33].

We report on the assembly and analysis of the first *Oxytricha* macronuclear genome, from the reference JRB310 strain. During and after assembly, we have addressed a number of challenges arising from the unusual structure of this genome, which we discuss. We focus on the most interesting and unique biological characteristics of this genome and place them in the context of the characteristics of the other sequenced ciliate macronuclear genomes.

## Results and Discussion

### Macronuclear Genome Assemblies

To assemble the *Oxytricha* macronuclear genome for the type strain—JRB310 [1]—we chose to build upon three assemblies, from ABySS [34], IDBA [35], and PE-Assembler [36]/SSAKE [37], based on Illumina sequences, and supplemented by a Sanger/454 assembly. To combine these assemblies, we developed a specialized meta-assembly pipeline (see Materials and Methods and Figure S1). Current genome assembly strategies for second-generation sequence data often employ multiple, hybrid strategies to overcome the experimental biases leading to low sequence coverage in particular genomic regions and repetitive DNA [38]. Since *Oxytricha*'s macronuclear genome was expected to have a low repeat content [2], repetitive DNA was expected to be a relatively insignificant issue, and thus even greedy genome assemblers were able to produce useful preliminary assemblies. However, unlike conventional genomes, the *Oxytricha* macronuclear genome provides assembly challenges by virtue of its fragmented architecture, variable processing (“alternative chromosome fragmentation”), and nonuniform nanochromosome copy number. We resolved these challenges during and after assembly.

The initial 454/Sanger genome assemblies contained a mixture of bacterial DNA, mitochondrial DNA, and up to two additional alleles other than those expected from the strain we originally proposed to sequence (JRB310) due to accidental contamination by a commonly used strain in our lab (JRB510—a complementary mating type), whereas the Illumina assemblies were produced from purified macronuclear DNA from the type strain (JRB310) alone. Given these contamination issues, we built our final assembly with the Illumina assemblies as the primary data source, rather than the 454/Sanger assemblies, to maintain the purity of our final assembly. This excluded virtually all bacterial and mitochondrial contamination in our final assembly since very few contigs in the Illumina assemblies derive from these sources (sucrose gradient purification of macronuclei eliminated almost all such contaminants) that could potentially be extended by the 454/Sanger data. We also kept JRB510 allelic data to a minimum in our final assembly, (i) by preferring best extensions, which were most likely to be from the more similar JRB310 derived contigs or reads, either from the Illumina assemblies or from the Sanger/454 assemblies and raw Sanger data (see Materials and Methods), and (ii) by the sequence consensus majority rule (the most abundant base at each position from the assembled contigs) of the CAP3 assembler [39] used to combine the three Illumina assemblies versus one 454/Sanger assembly during contig construction. The conclusion that our final assembly strategy succeeded in keeping JRB510 allelic information to a minimum is supported by matches to the three pure JRB310 Illumina starting assemblies. Our final assembly is 82.0% covered by identical BLAT matches ≥100 bp long to one of these pure JRB310 assemblies and 92.5% covered by 99.5% identity, ≥100 bp BLAT matches to these assemblies (note that consensus building by CAP3 may result in alternating selection of JRB310 polymorphisms from the original assemblies, and hence even meta-contigs assembled from the pure JRB310 assemblies may have BLAT matches that differ from all the original assemblies).

We chose to keep alleles apart by applying moderately strict criteria for merging during our meta-assembly (e.g., by merging contigs with overlaps at least 40 bp long and ≥99% identical with CAP3 [39]; see Materials and Methods). However, in order to maximize the number of complete nanochromosomes in our assembly, we collapsed some alleles (i.e., producing “quasi-nanochromosomes” derived from two alleles; see “Extensive Genome Homozygosity and High SNP Heterozygosity”). Merging of contigs is also complicated by alternative fragmentation, which affects ∼10% of the nanochromosomes and may either result in collapsing or splitting of nanochromosome isoforms (see “Extensive Alternative Nanochromosome Fragmentation”).

We discriminated between homozygous and heterozygous nanochromosomes after assembly (see the next section). We have not attempted to determine the haplotypes of the heterozygous contigs due to computational complications arising from both alternative nanochromosome fragmentation and variable representation of alleles (which need not be 1∶1).

A comparison of the *Oxytricha* macronuclear genome assemblies and meta-assembly is given in Table 1 (also see Tables S11–S17 for the progressive improvements in the genome assembly through the successive steps of our meta-assembly approach shown in Figure S1). Since the size selection used in the construction of our paired-end (PE) sequence library results in poor sequence coverage for a span of approximately 160 bp, roughly 100 bp from the telomeric ends (see Materials and Methods), the incorporation of single-end (SE) sequence data allowed ABySS to assemble far more contigs with telomeric sequences than either IDBA or the PE-Assembler/SSAKE assembler combination, neither of which could use SE and PE sequences simultaneously (Table 1). The ABySS assembly is larger (78.0 Mb) than the other assemblies (47.8 Mb for PE-Asm/SSAKE and 57.7 Mb for IDBA) and also more complete, as evidenced by a higher fraction of reads that can be mapped to this assembly (Table 1). The ABySS assembly also incorporates a substantially higher proportion of telomeric reads than the other two assemblers (91.8% for ABySS versus 45.4% for PE-Asm/SSAKE and 42.4% for IDBA) and contains a larger proportion of telomere-containing contigs (66.5% versus 18.3% for PE-Asm/SSAKE and 8.6% for IDBA) and longer contigs (mean length of 2,273 bp for ABySS versus 2,090 bp for PE-Asm/SSAKE and 1,204 bp for IDBA). Consequently, the ABySS assembler produced almost an order of magnitude more full-length nanochromosomes than the other two assemblers. Even though the ABySS assembly incorporates more telomeric reads than the other assemblies, it also excludes a higher proportion of telomeric reads than nontelomeric reads (92% telomeric PE reads versus 98% total PE reads map to the assembly; telomeric reads comprise ∼13% of all the reads). While the ABySS assembly appears to be the most complete, the majority of its contigs (81.3%) are still missing one or both telomeres.

**Table 1:** Comparison of *Oxytricha* macronuclear genome assemblies.

Assembler	PE-Asm/SSAKE	IDBA	ABySS	Final Assembly
Assembly size (bp)	47,753,834	57,684,531	78,039,140	67,172,481
Number of contigs	22,840	47,882	34,330	22,450
Number of telomeres	4,883	3,929	30,071	38,724
N50 (bp)	2,579	1,938	3,473	3,736
Mean contig length (bp)	2,090	1,204	2,273	2,982
2-telomere contigs	683	262	6,421	15,993
Mean 2-telomere contig length (bp)	3,070	3,148	3,243	3,187
Number of 1-telomere contigs	3,497	3,376	16,399	5,303
Mean 1-telomere contig length (bp)	2,773	2,260	2,182	2,694
Number of 0-telomere contigs	18,660	44,244	11,510	1,154
Mean 0-telomere contig length (bp)	1,927	1,112	1,861	1,655
Number of multitelomere contigs	20	28	776	1,279
Mean multitelomere contig length (bp)	3,438	3,436	5,049	4,208
Raw PE read coverage	78.7%	87.2%	98.0%	98.0%
PE telomeric read coverage	45.4%	42.4%	91.8%	91.0%
Raw SE read coverage	72.5%	80.5%	88.2%	88.5%
SE telomeric read coverage	29.0%	27.5%	64.4%	69.6%

The 2-telomere contigs have both 5′ (CCCCAAAACCCC; with degenerate bases—see Materials and Methods) and 3′ (GGGGTTTTGGGG; with degenerate bases) telomeric repeats. Note that 2-telomere contigs are mostly complete nanochromosomes but may also be alternatively fragmented nanochromosomes with one or more additional missing ends and that multitelomere contigs may be either alternatively fragmented nanochromosomes or nanochromosomes with internal telomere-like repeats. Raw read coverage is calculated from LAST (default parameters; version 159; contig telomeres were masked) matches (≥70 bp long and ≥90% identical) to the assemblies. Read coverage was calculated for the total high quality PE sequence data set and one of the three lanes of SE sequence data. Of the PE reads 13% were telomere bearing, as opposed to 4.7% of the SE reads.

The initial meta-assembly of the ABySS, IDBA, and PE-Asm assemblies yielded a modest improvement in the total number of full-length nanochromosomes relative to ABySS alone, with the ratio of full-length nanochromosomes to contigs increasing from 21% to 24% of the total number of contigs. Since the meta-assembly was still highly fragmented, and our aim was to assemble full-length nanochromosomes with complete genes, we developed a strategy that consisted of two rounds of extension of nontelomeric contig ends and reassembly (see Materials and Methods). This strategy produced an assembly where the majority (77%) of the contigs had both 5′ and 3′ telomeres. For the final meta-assembly, the average contig length (2,982 bp) is longer than that of any of the original assemblies (2,273 for ABySS versus 2,090 for PE-Asm/SSAKE and 1,204 for IDBA) and the read coverage is as high as or higher than the most complete ABySS assembly (98.0% coverage for PE reads for ABySS and the final assembly; 88.2% SE read coverage for ABySS and 88.5% SE read coverage for the final assembly). However, a larger fraction of telomeric reads than nontelomeric reads still do not map to the final assembly (e.g., 9.0% of telomeric PE reads versus 2.0% of all PE reads do not map to the assembly), indicating that some telomeric regions are still missing from the final assembly.

### Extensive Genome Homozygosity and High SNP Heterozygosity

Since there are currently no published effective population size estimates for *Oxytricha trifallax*, we wanted to obtain an estimate from allelic diversity of the macronuclear genome. Furthermore, current estimates of effective population size for other free-living model ciliates, *Paramecium* and *Tetrahymena*, differ [40]–[42], so additional estimates from other species will be necessary to determine if there are general trends in population size within ciliates.

Given our assembly conditions, we expect many allelic sequences to co-assemble, but visual inspection of reads mapped to the final assembly suggested that a substantial fraction of the genome is homozygous. A trivial explanation for this observed homozygosity is that the micronuclear genome regions (MDS) that form the nanochromosomes are homozygous, however it is also possible that a combination of other factors may be responsible for some of the observed homozygosity. These factors include both nanochromosomal allelic drift, arising from stochastic nanochromosome segregation during amitosis (during normal cellular replication) and nanochromosomal allelic selection, both of which could lead, in principle, to haplotype fixation (also known as “allelic assortment”), as well as allelic biases introduced during conjugation.

Some well-studied macronuclear nanochromosomes, including the 81 locus [43], and the nanochromosomes encoding the telomere end-binding proteins α and β (Contig22209.0 and Contig22260.0) are homozygous in the *Oxytricha* JRB310 strain upon which our reference macronuclear genome assembly is based. Knowledge of the fraction of homozygous and heterozygous nanochromosomes is also necessary to obtain a reasonable estimate of macronuclear genome size. To determine nanochromosomal homozygosity, we focused on nonalternatively fragmented nanochromosomes in order to avoid both ambiguous alignments and possible false identification of heterozygosity due to the presence of alternative telomere locations. Of the nonalternatively fragmented nanochromosomes, 66% (7,487 out of 11,297) had no substantial BLAT [44] nonself matches (≥100 bp and ≥90% identical; default BLAT parameters) to any other contig in the final assembly (“matchless” nanochromosomes).

Matchless nanochromosomes with variants present at ≥0.5% of the positions were considered heterozygous (see Materials and Methods for our precise definition of heterozygosity); otherwise they were considered to be homozygous. Note that our read mapping cutoff may reduce polymorphism estimates by filtering out reads with more polymorphisms (see Text S1, “Read Mapping Rationale”). These criteria overestimate low frequency variants at lower coverage sites and underestimate higher frequency variants at lower coverage sites (Figure S2) but comprise a small proportion of all the sites (e.g., 11.8% of sites identified as heterozygous are at 20–40× coverage). By these criteria, the well-characterized *Oxytricha* actin I [45],[46] nanochromosome (Contig19101.0) was correctly classified as heterozygous, with variants at 0.89% of the examined positions (12/1,350). Mismapped reads do not affect the identification of the heterozygous sites in this nanochromosome, since only two of the reads mapped to this nanochromosome map to any of the other contigs. For the actin I nanochromosome, the frequency of allelic variants varies from just over 5% (three positions) to 13.1% (mean 8.7%) corresponding to a roughly 1∶11 ratio of the minor∶major allele. Of the matchless nanochromosomes, 63% have sequence variants identified at 0%–0.5% of positions; hence, 37% of matchless nanochromosomes are classified as heterozygous by this criterion. Read mapping appears to be adequately sensitive to detect most SNPs (single nucleotide polymorphisms), since the mean number of reads per bp mapped to heterozygous and homozygous nanochromosomes is almost identical (see “Nanochromosome Copy Number Is Nonuniform”).

Approximately 42% of nanochromosomes are homozygous when the proportion of putative homozygous nanochromosomes is calculated from the total number of matchless and matched nanochromosomes. The high levels of macronuclear genome homozygosity agree with preliminary observations of micronuclear sequence data for this strain (Chen et al., unpublished), suggesting that the majority of nanochromosomal homozygosity may derive from homozygosity in the micronuclear genome, rather than other possible factors (allelic assortment and/or developmental biases). Once the micronuclear genome is more complete, it will be possible to assess how much these factors have contributed to the observed homozygosity. Nevertheless, the abundance of homozygous nanochromosomes in the final assembly (∼42%) suggests that the wild-isolate JRB310 [47] may actually be substantially inbred and that this inbreeding arose at its source. Deleterious inbreeding effects may contribute to the complexity of *Oxytricha trifallax* mating types [47]. It will be interesting to determine whether the two “promiscuous” *Oxytricha* strains (JRB27 and JRB51 [47]) that mate with the broadest set of other mating types are less inbred.

**Figure 4:**
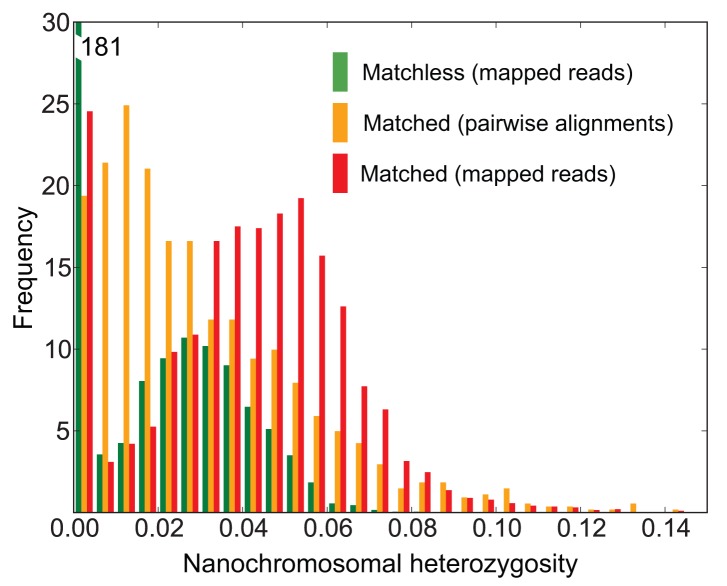
Nanochromosomal SNP heterozygosity. The green histogram (left) corresponds to SNP heterozygosity estimated from mapped reads (see Materials and Methods) for “matchless” nanochromosomes (which have no non-self contig matches to the genome assembly) and includes homozygous nanochromosomes. The red histogram (right) corresponds to SNP heterozygosity estimated from mapped reads for “matched” nanochromosomes. The orange histogram (center) corresponds to SNP diversity assessed from pairwise alignments of matched nanochromosomes. The smallest bin is 0–0.005 (0%–0.5%) heterozygosity.

Sequence polymorphisms are abundant in the *Oxytricha* macronuclear genome: excluding the homozygous nanochromosomes (which may have arisen from inbreeding), the mean SNP heterozygosity is 4.0% (SD = 1.8%; Figure 4). From mapped reads, for heterozygous matchless nanochromosomes, mean SNP heterozygosity is 3.1% (SD = 1.3%), and for heterozygous matched nanochromosomes, it is 4.6% (SD = 1.9%; Figure 4). For alignments of heterozygous matched nanochromosomes (with BLAT matches ≥100 bp and ≥90% identical to each other) produced by MUSCLE (for nanochromosome pairs where one of the nanochromosomes is no more than 10% longer than the other and for alignments with ≤15% differences), mean SNP heterozygosity is 3.0% (SD = 2.5%). These estimates of SNP heterozygosity indicate that assembly has masked a substantial amount of allelic variation. Similar statistics were obtained for the subset of the final assembly's nanochromosomes that were present in the pure JRB310 strain ABySS assembly (e.g., heterozygous matchless mean SNP heterozygosity is 2.8%, SD = 1.2%, and for MUSCLE alignments of heterozygous matched nanochromosomes, mean heterozygosity is 3.2%, SD = 2.9%). Hence it is unlikely that any residual JRB510 strain allelic information in our final assembly has had a considerable effect upon our estimates of heterozygosity. Nevertheless, these are first estimates from a complex genome assembly, complicated by homozygosity due to potential inbreeding, and hence inferences based upon them (including our subsequent population size estimate) should be treated with caution until better estimates from the micronuclear genome and additional strains become available.

At 4-fold synonymous sites from heterozygous nanochromosomes with matches (see Materials and Methods), the mean SNP heterozygosity is 8.3% (SD = 9.4%). This underestimates sequence diversity at 4-fold synonymous sites, since pairwise alignments of contigs underestimate SNP heterozygosity at all sites (see previous paragraph). If we apply a correction for the missing SNP heterozygosity based on our overall estimate of SNP heterozygosity, we obtain an estimate of 11.1% mean SNP heterozygosity at 4-fold synonymous sites [8.3%×(4.0%/3.0%)]. This 4-fold synonymous site SNP heterozygosity is very high—even higher than the current SNP heterozygosity record holder, *Ciona savignyi*, which has 8.0% 4-fold synonymous site mean SNP heterozygosity [48]. These high levels of SNP heterozygosity suggest that *O*xytricha *trifallax* has a large effective population size.

Assuming a mutation rate μ ∼10^−9^ per base per generation, as in Snoke et al. 2006 [42], for nucleotide diversity at 4-fold synonymous sites and π_4S_ = 4N_e_μ, we estimate an effective population size of 2.6×10^7^. This effective population size is on the same order estimated for *P. tetraurelia* (using silent site diversity, π_S_, which will yield a smaller population size estimate than one based on π_4S_) [42]. However, this estimate of the *P. tetraurelia* effective population size may be an overestimate due to incorrect classification of species within the *Paramecium aurelia* species complex and may be closer to the order of 10^6^ [40]. In contrast, the *T. thermophila* effective population size is estimated to be considerably smaller than *Oxytricha*'s, at N_e_ = 7.5×10^5^ for π_S_ = 0.003 (with μ = 10^−9^) [41],[42].

In laboratory culture conditions, *Oxytricha trifallax* tends to replicate asexually and rarely conjugates (resulting in meiotic recombination). Conjugation in the laboratory is induced by starvation as long as cells of compatible mating types are available. However, we do not know the frequency of conjugation relative to replication of *Oxytricha trifallax* in its natural environment. The relationships between the frequency of asexual reproduction, and additional population genetic factors arising from asexuality, such as the variance of asexual and sexual reproductive contributions, are complex and can result in increases or decreases in estimates of effective population size [49],[50]. As a result, effective population size estimates for *Oxytricha* should be treated with caution until these factors are better understood.

**Figure 5:**
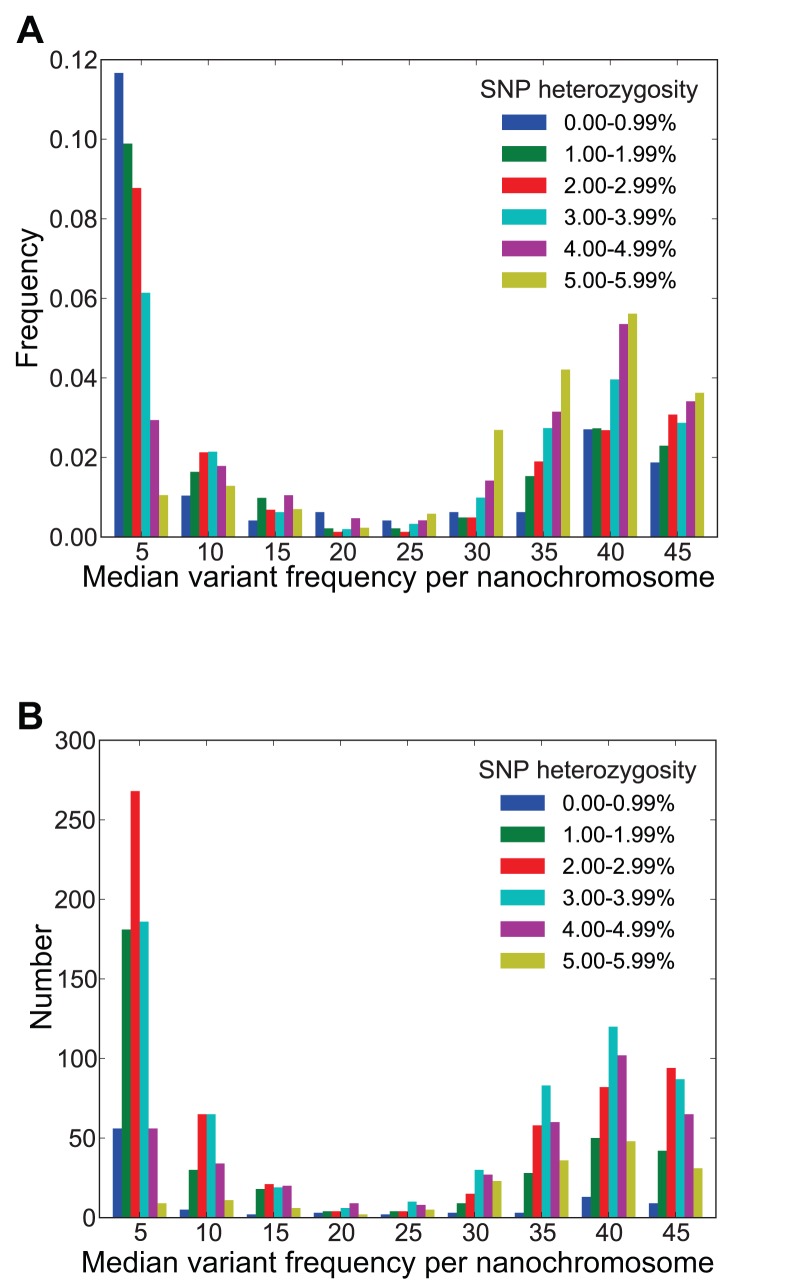
Nanochromosomal variant frequencies. (A) Normalized to form a probability density (cumulative frequency of 1) and (B) unnormalized median nanochromosomal variant frequencies for six increasing ranges of mean SNP heterozygosity. Variant frequencies were determined for nanochromosomes with no non-self matches to the genome assembly (the same nanochromosomes underlying the SNP heterozygosity histogram for “matchless” nanochromosomes in Figure 4), with variant positions called at the same minimum variant frequency as that used to determine potentially heterozygous sites (5% for sites with ≥20× read coverage). To exclude potentially paralogous mapped reads, we only analyzed nanochromosomes with ≤4 reads mapped to other contigs (using all nanochromosomes does not substantially change the form of the distributions). Variant frequency bins are labeled by their lower bounds. Variant frequencies ≥40 bp from either nanochromosome end were counted to avoid possible incorrect variant calling resulting from telomeric bases that were not masked (due to sequencing errors).

As indicated for the well-characterized actin I locus, which has a roughly 1∶11 ratio of minor∶major allelic variant, there may be substantial deviations from the expected 1∶1 ratio for the two possible allelic variants at each site. For matchless nanochromosomes, we found that the distribution of median nanochromosomal variant frequency (i.e., the median frequency of putative allelic polymorphisms) is bimodal, with one mode close to the expectation at 40%–45% and the other at 5%–10% (Figure 5; since the lower peak is bounded by the cutoff we chose to assess variants, the true lower peak may be lower than this). The bimodality of this distribution persists even if we only consider nanochromosomes where the mean coverage of the variant sites is high (e.g., at mean coverage of ≥110× at variant sites). Though some deviation from the 1∶1 variant ratio might result from allele-specific read mapping biases [51], given the relatively relaxed read mapping parameters we used (see Materials and Methods, “Read Mapping and Variant Detection”), the two variant frequency modes differ too much to explain the lower mode's existence. Instead, the deviation from the expected ratio may indicate that allelic assortment has occurred, or that there are developmentally specific allelic biases. Since a high proportion of nanochromosomes deviate substantially from the 1∶1 expected allelic ratio, it is also possible that allelic assortment has occurred for some nanochromosomes, which may contribute to the observed abundance of homozygous nanochromosomes. Nanochromosomes that deviate the most from the expected 1∶1 allelic ratio tend to have lower mean SNP heterozygosities, which likely reflects the diminished ability to detect SNPs with more distorted variant frequencies (Figure 5).

### Analysis of Assembly Redundancy and Estimation of Macronuclear Genome Size

We desired an estimate for the total haploid *Oxytricha trifallax* macronuclear genome size since it is unknown. To obtain a reasonable estimate, we needed to determine the extent of redundancy in our genome assembly. As judged by visual inspection of our original assemblies, the main sources of redundancy are (i) the two alleles from the partially diallelic genome (see “Extensive Genome Homozygosity and High SNP Heterozygosity”), (ii) alternative nanochromosome fragmentation (see “Extensive Alternative Nanochromosome Fragmentation”), (iii) erroneous base calling that may result from high copy number regions and relatively abundant sequencing errors, and (iv) paralogous genes. The assembly with the most redundancy—from ABySS (Figure S3)—has approximately half of its contigs with nonself matches that are identical or almost identical (matches that are ≥100 bp and ≥99% identical). Visual inspection of the ABySS assembly revealed that much of the redundancy arose from the combined effect of high copy number DNA and sequencing errors. Our assembly strategy eliminated most of the redundancy from erroneous base calling, because it collapsed regions that are nearly identical. A small quantity of additional redundancy may have also been introduced by the inclusion of non-reference (JRB510) allelic sequences from the Sanger/454 genome assemblies, though the strategy we used prefers the inclusion of reference allelic sequences (see Materials and Methods, “Princeton Illumina Assembly and TGI Sanger/454 Assembly Integration”). Though some redundancy remains in our final genome assembly, this is counteracted by ∼1/4 of the nanochromosomes that have had their alleles collapsed during the assembly (see “Extensive Genome Homozygosity and High SNP Heterozygosity”).

Given the ∼42% estimate of nanochromosomal homozygosity, we estimate that the haploid number of nanochromosomes is ∼15,600 [from 15,993+1,279 two- and multitelomere contigs and 5,303 single-telomere contigs (Table 1); we also estimate that ∼10% of nanochromosomes are alternatively fragmented (see “Extensive Alternative Nanochromosome Fragmentation”)]. With a mean nanochromosomal length of ∼3.2 kb, we estimate that the haploid macronuclear genome size is 50 Mb, which is similar to earlier experimental estimates [52],[53].

### The Macronuclear Genome Encodes All the Genes Necessary for Vegetative Growth

Traditional assessments of genome completeness are not very meaningful in *Oxytricha* because they usually measure genomes of uniform coverage with relatively long chromosomes. In two key ways, the *Oxytricha* macronuclear genome assembly is more similar to a de novo transcript assembly than to a conventional genome assembly: it contains multiple nanochromosome isoforms produced by alternative nanochromosome fragmentation (see “Extensive Alternative Nanochromosome Fragmentation”), and it is an assembly of nonuniformly amplified DNA (see “Nanochromosome Copy Number Is Nonuniform”). Unlike RNA transcripts, nanochromosome levels remain relatively stable during asexual growth [22], and variation of nanochromosome copy number is considerably lower than that of transcripts, so we are able to completely sample the genome's DNA over time.

Simple genome metrics indicate that our assembly is largely complete. Firstly, we have sequenced the genome to a substantial depth: we have >62× haploid coverage of the genome assembly by Illumina 100 bp PE reads and >48× haploid coverage by SE reads. Secondly, nearly all high-quality reads map to our final assembly (98% of high quality PE reads) and the majority of contigs (71.3%) represent complete nanochromosomes, with only 5.1% of the contigs missing both telomeres and 23.6% missing one telomere (Table 1). Finally, our 50 Mb haploid genome assembly size estimate is similar to an earlier estimate of ∼55 Mb for the DNA complexity of the *Oxytricha* macronucleus [2].

To assess genome completeness we analyzed the completeness of two specific, functionally related gene sets—encoding ribosomal proteins and tRNAs—and one general gene data set in *Oxytricha*. All of these measures of completeness indicate that the macronuclear genome assembly is essentially complete. Firstly, the final genome assembly contains all 80 of the standard eukaryotic ribosomal proteins (32 small subunit and 48 large subunit proteins). Secondly, the *Oxytricha* macronuclear genome has a haploid complement of ∼59 unique tRNA nanochromosomes (including a selenocysteine tRNA on Contig21859.0). These tRNAs are sufficient to translate all of its codons if wobble position anticodon rules [54] are accounted for. As judged by searches of tRNAdb [55], codons without cognate tRNAs in *Oxytricha* are either absent or rare in other eukaryotes. Furthermore, with the exception of a *Tetrahymena* glycine tRNA that has a CCC anticodon [56], *Oxytricha*'s tRNAs share the same anticodons as *Tetrahymena*.

We also assessed the completeness of the macronuclear genome by searches of predicted proteins against 248 “core eukaryotic genes” (CEGs: defined by KOGS [57] based on the complete protein catalogs of *H. sapiens*, *D. melanogaster*, *C. elegans*, *A. thaliana*, *S. cerevisiae*, and *S. pombe* [58]). Using a strategy similar to that used to assess completeness during analyses of ncRNAs in *Oxytricha* [59], and based on the CEGMA analysis strategy [58], we searched for all the core proteins using a match coverage of ≥70% of the mean CEG sequence length, accumulated over the span of the query CEG sequence matches from BLASTP (BLAST+ [60]; with at least one of the matches for each query having an E-value≤1e-10). Of our predicted proteins 231 had substantial sequence similarity to the core eukaryotic protein sequences. The numbers of core eukaryotic proteins we found in the latest *Tetrahymena* (223) and *Paramecium* (230) gene predictions were similar to those found in *Oxytricha* (within the limits of the sensitivity of the searches we used and possible gene prediction failures). However, since CEGS are defined for just five genomes of animals, fungi, and one plant and exclude a diversity of other eukaryotes, the true set of CEGs may be somewhat smaller than this. Given the great evolutionary divergences of ciliates from these eukaryotes (possibly in excess of 1.5 billion years ago [61]), it is also possible that the BLAST criteria employed by CEGMA are not sufficiently sensitive to detect more distant ciliate homologs. This suggests that the predicted proteomes of all three ciliates are largely complete.

Given that the deep divergences of ciliates might prevent detection of their homologs to the remaining 17 CEGS without matches, we attempted more sensitive searches at the domain level using HMMER3 [62]. We assigned Pfam domains to each KOG if the domains were best Pfam hits to the majority of the members of each KOG. Using domain searches, 13 of the remaining 17 core proteins had matches in Pfam-A (with domain, full-sequence E-values<1e-3; see Text S1, “Pfam Domains Detected for CEGs Missing in *Oxytricha*”). With the exception of KOG2531, these CEGs are relatively short, single-domain proteins.

Of the four undetectable CEGs remaining after HMMER3 searches, one, KOG3285, is an ortholog group corresponding to the MAD2 [63] spindle assembly checkpoint (SAC) protein. We were unable to detect homologs of additional SAC proteins such as MAD1 and MAD3, suggesting that these checkpoint proteins have either been lost or that they are very divergent. The *Tetrahymena* macronuclear genome paper reported the absence of the checkpoint kinase CHK1 [56], but this is difficult to establish unambiguously given both the considerable divergence of ciliate proteins from the model organisms in which this protein was discovered and that the single defining domain for this protein is the widely distributed and extremely common protein kinase domain (PF00069). There is no evidence of a mitotic spindle in ciliate macronuclei [16], so they may not need SAC proteins, unlike conventional nuclei. It is also possible that the lack of these proteins contributed to the evolution of ciliate amitosis. However, micronuclei do appear to undergo a spindle-guided mitosis [2],[16]. Since ciliates need to coordinate the division of multiple nuclei, their nuclear cycle checkpoints may be more complex than most eukaryotes; hence, they may use genes that are nonorthologous to conventional checkpoint genes in nonciliates.

The other three undetectable CEGs—KOG0563, KOG3147, and KOG2653—correspond to three key oxidative pentose phosphate pathway (OPPP) enzymes: glucose-6-phosphate dehydrogenase (G6PD), gluconolactonase (6PGL), and 6-phosphogluconate dehydrogenase (6PGD), which are also missing in *Paramecium* and *Tetrahymena*, and hence may have been lost in ciliates (Figure S4). Two of these enzymes—G6PD and 6PGL—were also noted to be missing in *Paramecium*, *Tetrahymena*, and *Ichthyophthirius* [64]. Thus, excluding these three CEGs that appear to be absent from ciliates, *Oxytricha*'s macronuclear genome is only missing one CEG (KOG3285/Mad2).

Together, since the macronuclear genome has 244/245 (99.6%) of the ciliate-restricted CEGs, it (together with the mitochondrial genome [65]) is likely to encode a complete set of genes required for vegetative growth. This is consistent with the observation of amicronucleate *Oxytricha* species in the wild, which are capable of vigorous replication for hundreds of generations in culture [2],[66], and with temporary dispensability of the micronucleus. Currently TBE transposon genes are the only published examples of micronuclear-limited genes (not encoded on any of the nanochromosomes in our assembly) in *Oxytricha*. These genes are exclusively expressed during sexual development and appear to be essential for accurate genome rearrangements [30], and hence may only need to be expressed from the micronucleus.

### Extensive Alternative Nanochromosome Fragmentation

A characteristic feature of the *Oxytricha* macronuclear genome is the existence of multiple, stable “versions” of nanochromosomes that share genic regions [10]–[12]. “Alternative processing” or “alternative fragmentation” of DNA is analogous to alternative splicing of introns from pre-mRNAs, but unlike alternative RNA splicing, macronuclear DNA is simply fragmented (with telomere addition), rather than joined together. Variable deletion of micronuclear DNA in *Paramecium* also gives rise to alternatively fragmented macronuclear chromosomes, though it produces much longer multigene chromosomes and this alternative fragmentation is much less frequent than that in *Oxytricha* [67]–[69]. Initial surveys of *Oxytricha fallax* nanochromosomes revealed a substantial amount of alternative nanochromosome fragmentation, with 40% (6/15) of the surveyed nanochromosomes alternatively fragmented [11], so we wanted to assess this on a genome-wide scale. We also sought evidence of possible functional relationships between alternative fragmentation and gene expression, since, in principle, alternative nanochromosome fragmentation may affect gene expression by (i) permitting variable amplification of nanochromosome isoforms, thereby affecting basal transcription levels of the genes encoded on these isoforms (see “Nanochromosome Copy Number Is Nonuniform”), (ii) gene truncation, and (iii) affecting regulation of gene expression by modulating which regulatory elements are present on nanochromosomes.

The creation of contigs during assembly merges shorter alternative nanochromosomes into longer isoforms, obscuring telomeric repeats when they contribute a minority of bases, thus making it difficult to identify the alternative isoforms directly from the contig sequences. Therefore, we exploited two sources of raw sequence data to uncover this kind of variation: 454 telomeric read pairs and Illumina telomeric reads (see Materials and Methods). From alternative fragmentation sites predicted by either data source, almost 1/4 of all the nanochromosomes (3,369/14,390) in our final assembly are predicted to be alternatively fragmented (only counting contigs with terminal telomeres ≤100 bp from either end of the contig). We predict 11% (1,909/17,372) and 14% (2,380/17,372) of nanochromosomes are alternatively fragmented from 454 telomeric reads alone and Illumina telomeric reads alone, respectively. Of the nanochromosomes predicted to be alternatively fragmented by Illumina telomeric reads, 63% are also predicted to be alternatively fragmented by 454 telomeric reads, and 68% of the nanochromosomes predicted to be alternatively fragmented by 454 telomeric reads are also predicted to be alternatively fragmented by Illumina telomeric reads. The actual portion of alternatively fragmented nanochromosomes may be closer to 10% since many of the predicted sites are only supported by a few reads (which results in a poor correspondence between the predictions from the two data sources when there are few telomeric reads at a putative alternative fragmentation site; see Text S1, “Classification of Strongly and Weakly Supported Alternative Fragmentation Sites”). We propose that most of the nanochromosomes arising from weakly supported sites with few supporting telomeric reads (e.g., <9 llumina telomeric reads) may represent “developmental noise” or healing of broken nanochromosomes by capping the broken ends with telomeres, rather than functional nanochromosomes.

We may not have recovered some alternatively fragmented nanochromosome isoforms due to limitations of our genome assembly. Since we focused on nanochromosomes with telomeres at both ends, some alternative fragmentation will be missed on nanochromosomes that lack telomeric ends (e.g., the alternatively fragmented 81-Mac locus [10],[11], represented by Contig13637.0.1, is missing both ends). Another possible failure to detect alternative fragmentation is a consequence of the semigreedy nature of our genome assembly strategy, since we stop extending nanochromosomes once we have detected at least one 5′ and at least one 3′ telomeric repeat (see Materials and Methods), which means that we may miss some longer unfragmented nanochromosome isoforms. Consequently, we consider our estimates of the level of alternative fragmentation to be conservative.

Alternative fragmentation sites tend to map between predicted genes in intergenic regions rather than within intragenic regions [Table S1; we use inter-CDS regions rather than intergenic regions since CDS (coding sequence) predictions are more reliable than UTRs (untranslated regions)]. For contigs with single internal alternative telomere fragmentation sites, strongly supported alternative fragmentation sites are 58 times more likely to be located in inter-CDS regions than in intra-CDS regions (per bp of these sequence regions). For nanochromosomes with single-gene predictions, strongly supported alternative fragmentation sites are 27 times more likely to reside within non-CDS regions (i.e., introns, UTRs, subtelomeric regions, or regions with no predicted gene), than within CDSs (Table S2). Strongly supported, noncoding alternative fragmentation sites typically have more telomere-containing reads than do coding alternative fragmentation sites for both single (mean 213 versus 95 reads) and two-gene [mean 186 (intergenic region) versus 116 reads] nanochromosomes.

For strongly supported alternative fragmentation sites predicted by Illumina telomeric reads, 74% (1,208/1,622) of alternatively fragmented nanochromosomes have one site of alternative fragmentation (giving rise to two nanochromosome isoforms: a long unfragmented form and a shorter fragmented isoform), and 21% of alternatively fragmented nanochromosomes have two sites of alternative fragmentation (similar statistics were obtained from strongly supported sites predicted from 454 telomeric reads). This means that typically only a few possible nanochromosome isoforms are produced for each of our assembled nanochromosomes and also that most alternative fragmentation is “directional,” giving rise to only one of the two possible single shorter isoforms. The most extreme example has seven alternative fragmentation sites predicted from the Illumina telomeric reads (at most nine from 454 telomeric reads) for Contig14329.0 (GenBank Accession: AMCR01001519.1).

The observation of directional alternative fragmentation suggests there must either be differential amplification of particular isoforms or degradation of specific forms following excision. The higher amplification levels of alternatively fragmented nanochromosomes relative to nonalternatively fragmented nanochromosomes (see next section) provides support for the first model but does not exclude the second. In the future, it will be interesting to determine how these fragmentation signals relate to chromosome amplification, since the timing of DNA fragmentation correlates with nanochromosome copy number in *Euplotes* [25].

**Figure 6:**
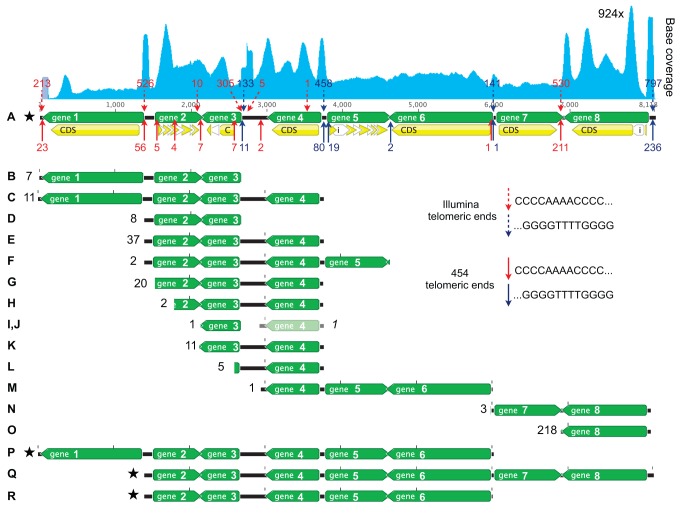
Extreme nanochromosomal fragmentation. Contig14329.0 is shown with coordinates in bp. Predicted genes, coding sequences, and introns are indicated by horizontal green, yellow, and white arrows, respectively. 5′ and 3′ fragmentation sites predicted from telomeric read pairs are indicated by red and navy arrows, respectively, with upward pointing solid arrows for sites predicted by 454 telomeric reads and downward pointing dashed arrows for sites predicted by Illumina telomeric reads. Numbers above/below the arrows indicate the number of telomeric reads found at each site for the two telomeric read sources. Alternative nanochromosome isoforms predicted from the 454 telomeric reads (isoforms B–O) are shown below the main locus, with the number of supporting read pairs next to each form. One additional isoform missed by our prediction method but documented in the 454 telomeric read pairs is indicated in pale green. Since the two fragmentation positions at 3,762 and 3,806 bp are in close proximity to each other, they were treated as a single point during alternative isoform prediction. Additional nanochromosome isoforms that were not detected by 454-telomeric reads, including the full eight-gene nanochromosome, but were detected by Southern blotting are indicated by stars (isoforms A, P, Q, and R). Sequence coverage, indicated by the cyan graph, shows the cumulative DNA amplification for all the nanochromosome isoforms. Sequence coverage is calculated from both Illumina telomereless and telomeric reads; telomeric read pairs appear as twin peaks ∼300 bp apart.

The longest isoform of the most extreme case of alternative fragmentation we discovered (Contig14329.0) is about 8 kb long with eight distinct protein-coding regions. This contig has 15 predicted telomere addition sites (TASs) (nine 5′ and six 3′ sites relative to the contig orientation in the assembly) from the 454 telomeric reads (with 11 strongly supported sites, including the two terminal sites), giving rise to up to 14 distinct nanochromosome isoforms from the same 8.1 kb region (Figure 6). An alternative fragmentation site at ∼6,000 bp is weakly supported by 454 telomeric reads but strongly supported by Illumina telomeric reads, suggesting that this site largely gives rise to longer nanochromosome isoforms that the 454 telomeric reads are less likely to detect. Every one of the alternative fragmentation sites predicted from Illumina telomeric reads, with the exception of a single weakly supported site at 4,767 bp, was corroborated by 454 telomeric reads within 100 bp of the site. The 454 telomeric reads suggest that each of the seven intergenic regions in this contig is a site of alternative fragmentation (no Illumina telomeric reads map to the site between genes 5 and 6). Consistent with the genome-wide pattern, this contig's alternative fragmentation sites typically reside between, not in the middle of, genes. For the 454 telomeric reads, only a small portion (2/15 sites) of the fragmentation sites are predicted to be in coding regions, and these sites are weakly supported, whereas the Illumina telomeric reads do not predict any sites in coding sequence regions.

To experimentally validate the predicted extreme fragmentation of Contig14329.0, we performed Southern hybridization (Figure S5) on the same vegetative *Oxytricha* JRB310 macronuclear DNA sequenced by Illumina. With two exceptions, our Southern analysis confirmed all tested nanochromosomes and identified four novel isoforms: the full length ∼8 kb isoform A, and isoforms P, Q, and R (Figure 6, Figure S5; Text S1, “Examination of Discrepancies Between Predicted and Experimentally Determined Alternative Fragmentation Isoforms of the Highly Fragmented Contig14329.0”). Since the process of generating 454 telomeric reads included a size selection (see Text S1, “Whole Nanochromosome Telomere-Based Library Construction”), it is unsurprising that sequencing missed the longer isoforms we were able to detect by Southern hybridization (A, P, Q, and R, at 8.1 kb, 6 kb, 6.5 kb, and 4.5 kb long, respectively).

The eight genes encoded on these alternatively fragmented nanochromosomes are (1) an RNAse HII domain containing protein (Pfam: PF01351), (2) a dsDNA-binding domain (PF01984) protein, (3) a Tim10/DDP family zinc finger domain protein (PF02953), (4) a protein with no significant BLASTP (to GenBank NR; E-value<1e-3) or Pfam matches (E-value<1e-3), (5) a COPI-associated protein domain (PF08507) protein, (6) an uncharacterized conserved protein (DUF2036) protein (PF09724), (7) another protein with no significant BLASTP or Pfam matches, and (8) a translation initiation factor eIF3 subunit (PF08597) protein. From the domain annotations, no obvious functional relationship amongst these genes is evident. From Figure 6, it can be seen that the representation of genes 2, 3, 4, and 8 in the different nanochromosomal isoforms is greater than for the remainder of the genes. Two of the shortest *Oxytricha* proteins (encoded by genes 2 and 3; see also Text S1, “Analysis of Short Protein and ncRNA-Encoding Nanochromosome”) are encoded on the most abundant nanochromosomal isoforms. Remarkably, in contrast to the surrounding, heterozygous DNA encoding genes 1–3 and gene 8, the ∼4.2 kb DNA region encoding genes 4–7 appears to be completely homozygous, suggesting the possibility that these regions derive from different micronuclear sources.

### Nanochromosome Copy Number Is Nonuniform

**Figure 7:**
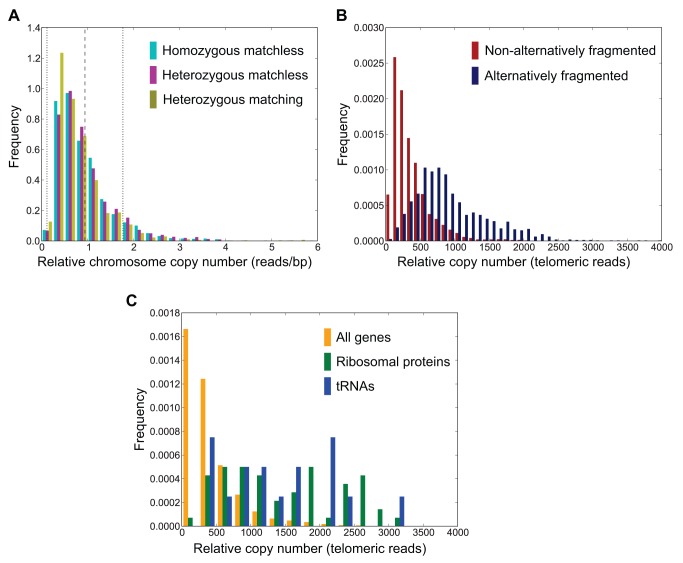
Nanochromosome copy number variation. (A) Relative nanochromosome copy number distribution (number of telomere-less reads/bp of nonsubtelomeric nanochromosome; see Materials and Methods) for homozygous matchless, heterozygous matchless, and heterozygous matching nanochromosomes. The mean copy number of the combined homozygous matchless and heterozygous matchless nanochromosomes is indicated by a dashed line at 0.94, with dotted lines corresponding to an interval of ∼1.3σ (∼0.12 to 1.76) either side of the mean, which includes ∼90% of all nanochromosomes. (B) Relative nanochromosome copy number of nonalternatively fragmented (with a single, directional fragmentation site per nanochromosome) versus alternatively fragmented nanochromosomes measured by the number of telomeric reads per nanochromosome. (C) Relative nanochromosome copy number of nonalternatively fragmented chromosomes versus nonalternatively fragmented chromosomes encoding ribosomal proteins and tRNAs.

In contrast to the oligohymenophorean ciliates, which typically have uniformly amplified macronuclear genomes [56],[69], there is considerable variation in nanochromosome copy number in *Oxytricha*. The distribution of copy number for nonalternatively fragmented nanochromosomes is right-skewed and is restricted around a mean relative copy number of 0.94, with ∼90% of the nanochromosomes contained within a relative copy number range of 0.12–1.76 centered on the mean (Figure 7A). It is possible that some lower copy number nanochromosomes may not have completely assembled since the combined depth of sequence coverage is <120× and lower bound copy number estimation is constrained by the >62× coverage of the PE reads. Mindful of these limitations, within the sequenced JRB310 clonal population of cells, nanochromosome copy number does not appear to vary as much as gene transcription. The most highly amplified nanochromosome, encoding the 18S, 5.8S, and 28S rRNA (Contig451.1), has a copy number that is ∼56× the mean of nonalternatively fragmented nanochromosomes, yet its transcripts typically yield more than 90% of the RNA in our non-poly(A)-selected RNA-seq samples. There is a roughly 2-fold difference between the most highly amplified nanochromosome and the next most highly amplified nanochromosome, encoding the 5S rRNA (Contig14476.0/Contig17968.0; quasi-allelic contigs).

Since the method we used to estimate nanochromosome copy number combines reads from both possible alleles for heterozygous nanochromosomes, it is necessary to map the reads sensitively to avoid exclusion of reads and to minimize incorrect mapping in order to obtain accurate estimates (see “Genome Homozygosity and SNP Heterozygosity”). Our mapping procedure seems to be appropriate for matchless nanochromosomes, since there is no substantial difference in copy number distributions for homozygous and heterozygous matchless nanochromosomes (mean copy number of 0.93, SD = 0.61, and 0.97, SD = 0.67, respectively; Figure 7A). However, for heterozygous nanochromosomes with matches, the mean nanochromosome copy number is lower (0.81; SD = 0.59; Figure 7A) than for matchless nanochromosomes. This is likely because some of the nanochromosomes with matches exhibit higher heterozygosity regions than matchless heterozygous nanochromosomes (>6% mean SNP heterozygosity; see “Genome Homozygosity and SNP Heterozygosity”) and the mapping criteria (≥94% read identity to the mapped contig) eliminated some of the more heterozygous reads.

To assess nanochromosome copy number of alternatively fragmented versus nonalternatively fragmented nanochromosomes, we examined the relationship between the number of telomeric reads and the number of nontelomeric reads per bp of the nanochromosomes (see Materials and Methods). We found that there was a good correlation between telomeric reads from either end of the nonalternatively fragmented nanochromosomes (Figure S6B) with *r* = 0.90. However, there are examples where the number of reads from each nanochromosome end differs substantially (e.g., Contig22209.0 and Contig5780.0 from Table S3). This may indicate the failure to extend the ends of some nanochromosomes completely or that the ends derive from the DNA of the nonreference strain (JRB510) and are relatively divergent with few reads from the reference DNA mapped to them (e.g., Contig5780.0). Alternatively, there may be experimental biases that skew the numbers of reads mapped to the two ends (e.g., Contig22209.0, which has JRB310 telomeric reads mapped to the nanochromosome end with fewer reads but no reads extending further, even with relaxed read mapping parameters). The correlation between the number of reads per bp and the number of telomeric reads per nanochromosome is also strong (*r* = 0.89; Figure S6A), indicating that assessment of telomeric reads alone is appropriate for large-scale analyses of nanochromosome copy number. Furthermore, our estimates of relative nanochromosome copy number, either via reads per bp or the number of telomeric reads per contig, are in good agreement with those obtained by qPCR (Table S3; Figure S7).

For relative nanochromosome copy number measured by telomeric reads, the mean number of telomeric reads per alternatively fragmented nanochromosome with a single (directional) alternative fragmentation site (i.e., only two nanochromosome isoforms) is 2.4 times (885 reads, SD = 768 reads) that of nonalternatively fragmented nanochromosomes (363 reads; SD = 290 reads; K-S one-sided test D = 0.59 and *p* value<1e-9, with the alternative hypothesis that alternatively fragmented nanochromosome copy number>nonalternatively fragmented nanochromosome copy number; Figure 7B). It follows that the DNA of the shorter alternative nanochromosome isoforms is even more highly amplified than that of nonalternatively fragmented nanochromosomes. The greater amplification of alternatively fragmented nanochromosomes relative to nonalternatively fragmented nanochromosomes supports a model of net overamplification of specific alternatively fragmented nanochromosomes isoforms rather than a model of net destruction. The higher amplification of alternatively fragmented nanochromosomes may indicate a commensal DNA relationship between two genes, arising when one of the genes benefits from the amplification signal of a more highly amplified nanochromosome isoform bearing another gene. This relationship requires no functional association between the genes on alternatively fragmented nanochromosomes, consistent with our general observations (e.g., no specific functional associations between nonribosomal genes and ribosomal genes on alternatively fragmented nanochromosomes).

For nonalternatively fragmented nanochromosomes, the ribosomal protein-encoding nanochromosomes are ∼3.9× more highly amplified than nonribosomal protein nanochromosomes, and tRNA-encoding nanochromosomes are ∼3.6× more highly amplified than non-tRNA-encoding nanochromosomes (Figure 7C; for ribosomal versus nonribosomal nanochromosomes: K-S one-sided test D = 0.64 and *p* value<1e-9, with the alternative hypothesis that ribosomal nanochromosome copy number>nonribosomal nanochromosome copy number; for tRNA versus non-tRNA nanochromosomes: K-S one-sided test D = 0.62 and *p* value<1e-6, with the alternative hypothesis that tRNA nanochromosome copy number>non-tRNA nanochromosome copy number). Similarly, the ribosomal protein- and tRNA-encoding nanochromosome isoforms arising from alternative fragmentation are typically overamplified relative to the isoforms that encode other genes (50/54 alternatively fragmented ribosomal nanochromosomes and 25/28 alternatively fragmented tRNA nanochromosomes; Figure S8).

Given the modest variation in nanochromosome copy number, most notably the limited overamplification of nanochromosomes encoding highly expressed genes (rRNAs, tRNAs, and ribosomal proteins), even if a strong correlation exists between nanochromosome copy number and transcription levels, copy number may only be a modest contributor to the final RNA and protein expression levels. Regulation of expression at the transcriptional/posttranscriptional level may be essential to buffer the variation in DNA copy number that arises during extended periods of vegetative growth.

### Nanochromosome Length Variation

**Figure 8:**
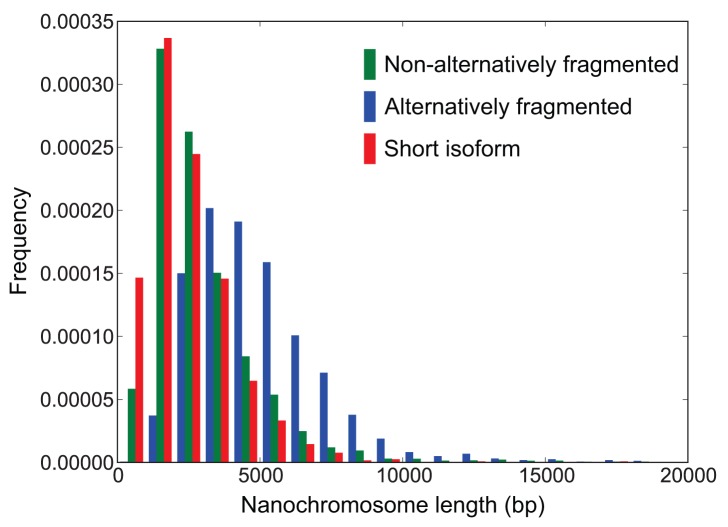
Length distributions of alternatively and nonalternatively fragmented nanochromosomes. The shortest nanochromosome isoforms produced from single (directional) alternative fragmentation sites are labeled as “Short isoform.” The histograms show normalized frequencies for 1,587 alternatively fragmented nanochromosomes and 15,219 nonalternatively fragmented nanochromosomes. Alternatively fragmented nanochromosomes have at least one strongly supported (≥10 Illumina reads) alternative fragmentation site >250 bp from either end of the nanochromosome (these nanochromosomes are >500 bp long).

*Oxytricha* nanochromosomes range in length from ∼500 bp to 66 kb, with a mean size of ∼3.2 kb (Figure 8). Few nanochromosomes were assembled at either extremity of the length distribution, with just 32 shorter than 600 bp long and 61 longer than 15 kb, consistent with observations of macronuclear DNA on electrophoretic gels [2],[70]. While the mean length of two-telomere nanochromosomes in the final *Oxytricha* macronuclear genome assembly is ∼3.2 kb (Table 1), the true average length of nanochromosomes is shorter than this because the longest isoform of alternatively fragmented nanochromosomes is the one that tends to be assembled. On electrophoretic gels, *Oxytricha* nanochromosomes are visibly longer than those of *Euplotes* [2],[71], which we propose is primarily a consequence of the lack of alternative fragmentation in *Euplotes* (inspection of mapped reads to our preliminary *Euplotes crassus* assembly indicated no signs of alternative fragmentation; unpublished data). The longest isoforms of alternatively fragmented nanochromosomes average 5.0 kb (SD = 2.4 kb), while nonalternatively fragmented nanochromosomes have a mean length of 3.0 kb (SD = 2.4 kb; Figure 8). The mean length of the shortest nanochromosome isoforms produced by alternative fragmentation is 2.4 kb (SD = 1.6 kb). For single-gene nonalternatively fragmented nanochromosomes, the mean nanochromosome length is 2.2 kb (SD = 1.0 kb).

The shortest assembled nanochromosome (Contig20269.0) is a mere 248 bp, excluding the telomeric sequences. Though we were unable to identify any ORFs or any ncRNAs on this nanochromosome by RFAM searches, we found two matching RNA-seq PE reads, suggesting that there is expression from this nanochromosome. The shortest nanochromosome (Contig19982.0) with a known protein is 469 bp (excluding the telomeres) and encodes a 98 aa ThiS/MoaD family protein, while the shortest ncRNA-bearing nanochromosome we found is 540 bp (excluding telomeres) and encodes tRNA-Gln(CUG) (see Text S1, “Analysis of Short Protein and ncRNA-Encoding Nanochromosomes”). Searches for shorter possible nanochromosomes in the Illumina and Sanger reads did not reveal additional plausible nanochromosome candidates (Text S1, “Reads Containing Both Putative Telomeric Repeats Are Not Genuine Nanochromosomes”).

The longest nanochromosomes (>15 kb) typically encode a single large structural protein (Table S4), such as dynein heavy chain proteins (e.g., Contig354.1). None of the 20 longest nanochromosomes are alternatively fragmented. Seven of these 20 nanochromosomes contain multiple predicted genes (up to a maximum of four); however, all but one of these gene predictions are oriented head-to-tail, consistent with the possibility that their predictions may have been incorrectly split. Hence most of the longest nanochromosomes are likely still single-gene nanochromosomes. One ∼20 kb nanochromosome (Contig289.1) does indeed contain multiple genes, since it encodes a Pkinase domain (PF00069) protein on the opposite strand to two predicted PAS_9 domain (PF14326) proteins (though these latter two proteins may also be incorrectly split). Six of the longest nanochromosomes encode single proteins with no detectable Pfam domains (Pfam-A 26; independent E-value<0.01) but all have BLASTP NCBI non-redundant database (nrdb) matches (E-value<1e-10), typically to large proteins (>2,000 aa).

The longest nanochromosome (Contig7580.0) is 66 kb (65,957 bp; excluding telomeres) and encodes a single giant protein (“Jotin,” after a Norse giant) with BLASTP best hits to *Titin*-like genes in the NCBI nrdb (see Text S1, “Characterization of the Jotin Protein”). We note that this single-gene nanochromosome is comparable in size to the entire, relatively large and gene-rich ∼70 kb *Oxytricha* mitochondrial genome [65], which was largely eliminated by the sucrose gradient isolation of macronuclei (see Materials and Methods). The *Oxytricha* Jotin ORF is 64,614 bp. AUGUSTUS predicts four short introns (117, 151, 77, and 63 bp), two of which are supported by and one of which conflicts with RNA-seq reads. This gene's entire coding sequence is well supported by pooled RNA-seq reads (covered from end to end).

### Gene Predictions

The gene prediction software, AUGUSTUS, predicted complete genes on 15,387 of the complete nanochromosomes we surveyed (96%) and 91% of the final assembly's contigs. Examination of three developmental time points (0, 10, and 20 h after initiation of conjugation) confirms transcription of 97% of *Oxytricha* nanochromosomes (94% of all contigs). AUGUSTUS predicts genes on 94% of nanochromosomes with expression evidence.

Most *Oxytricha* nanochromosomes (80%) contain single genes, consistent with earlier studies (Figure S9) [2],[33]. Alternatively fragmented nanochromosomes tend to encode more genes per nanochromosome: only 15% of alternatively fragmented nanochromosomes have single gene predictions, versus 90% of all nonalternatively fragmented nanochromosomes. Roughly half (48%) of multigene nanochromosomes have alternative fragmentation. All nanochromosomes with five or more (maximum eight) predicted genes are alternatively fragmented (Figure S9), and only two nonalternatively fragmented nanochromosomes encode four genes. The nanochromosome with the largest number of separate gene products (Contig8800.0; ∼6.8 kb) is alternatively fragmented, with a shorter ∼3.5 kb nanochromosome isoform that encodes 12 C/D snoRNAs [59] and a putative protein-coding gene encoded by the remainder of the full-length isoform.

Key properties of *Oxytricha*'s gene predictions are consistent with a pilot survey [33], including relative AT-richness (34% GC) with noncoding regions that are more AT-rich than coding regions (e.g., introns are 23.6% GC), and 1.6 introns per gene (Table 2). *Oxytricha* gene lengths (mean length 1,839 bp excluding UTRs) are similar to those predicted for *Tetrahymena* [56].

**Table 2:** Properties of gene predictions.

Feature	Number	Mean (bp)	Min (bp)	Max (bp)	%GC
Genes	17,040	1,839	150	65,451	34.0
Exons	43,759	661	3	45,409	34.2
Introns	26,719	90	28	549	23.6
5′ upstream of start codon	11,439	266	35	5,398	26.1
3′ downstream of stop codon	11,439	210	10	3,859	26.7

Prediction features were obtained for complete nanochromosomes (14,388 in total) only. Gene lengths are from the start to stop codons and exclude UTRs. Up- and downstream regions were only determined for single-gene nanochromosomes and include the 5′ and 3′ UTRs. %GC estimates exclude telomeric bases.

### Possible Functional Differences in the Predicted Proteomes of Ciliates With and Without Genome Unscrambling

Functional differences between the model ciliates may have evolved in numerous ways, given their tremendous divergence. Here we focus on two key differences: absence/presence of protein domains in specific ciliates and expansions of protein families at the level of protein domain. We were particularly interested in comparing the protein domains present in either ciliates with gene scrambling (*Oxytricha*) or that lack evidence of gene scrambling (*Paramecium*, *Tetrahymena*, and *Euplotes*; Figure 2; Tables S5 and S6; also see Table S7 for genes found in *Paramecium* and *Tetrahymena* that are absent in *Oxytricha*), since many species-specific proteins appear associated with macronuclear genome differentiation. Examples include the transposases in *Oxytricha* (micronuclear-limited TBE transposases) [30], *Tetrahymena* and *Paramecium* (piggyBac transposase—“*PiggyMac*”) [72],[73], and the *Paramecium* RNA binding Nowa proteins [74].

Since we were interested in DNA rearrangement, we searched for differences in the nucleic acid binding and nucleic acid metabolism domain content between the ciliates with and without evidence of extensive gene scrambling (see Materials and Methods). We identified 43 such nucleic-acid-related domains that are present in *Oxytricha* (“*Oxytricha*-specific” domains) but absent from both *Tetrahymena* and *Paramecium* (Table S5 and Table S6).

#### Domesticated macronuclear transposases

**Figure 9:**
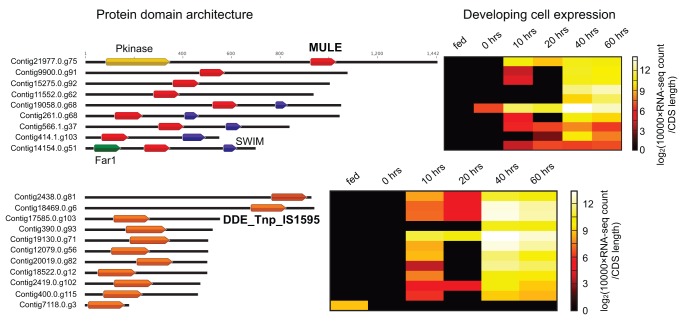
Transposase-like domains of proteins found in *Oxytricha* but neither *Paramecium* nor *Tetrahymena*. Proteins are shown with black lines with a scale in amino acids indicated above the longest protein. Protein names are to the left of the protein diagrams. Domain coordinates are the Pfam domain envelope coordinates. Representative domains are given their Pfam names, with transposase-like domains shown in bold. Gene expression levels are log_2_[10,000×normalized RNA-seq counts (see Text S1; Supporting Materials and Methods) divided by CDS length (in bp)] before (“fed”) and during conjugation (0–60 h).

The most striking difference in the nucleic-acid-associated protein domains of *Oxytricha* and *Paramecium*/*Tetrahymena* is the number of *Oxytricha*-specific transposon-like or transposon-associated domains—27 out of 83 Pfam domain matches (Table S5 and Table S6). All of the proteins that possess these transposase domains, with the exception of the proteins with the DDE_Tnp_1 and DDE_Tnp_1_3 domains (whose genes are on telomere-lacking contigs, Contig6077.0 and Contig4212.0, and whose best GenBank BLASTP matches are all bacterial), have typical *Oxytricha* glutamine codon usage (including UAG and UAA codons), which precludes bacterial contamination. We discovered three types of *Oxytricha*-specific macronuclear-encoded transposase-like domains: Phage_integrase, DDE_Tnp_IS1595, and MULE. All of these domains belong to proteins encoded on complete nanochromosomes. Since the nanochromosomes encoding either DDE transposase domain (DDE_Tnp_IS1595 or MULE) lack terminal inverted repeats characteristic of transposons with these types of transposases, they appear to be domesticated versions, like the macronucleus-encoded *PiggyMac* transposases in *Paramecium* and *Tetrahymena* [72],[73]. The DDE_Tnp_IS1595 and MULE domains are the two most abundant nucleic-acid-related, *Oxytricha*-specific protein domains, present in 11 and 9 distinct proteins (Figure 9).

The MULE domain name derives from the class of transposons known as *Mutator*-like transposable elements, which are widely distributed among angiosperms [75]. The domain is found in transposases that regulate the activity of the most mutagenic plant transposon, *Mutator* (reviewed in [75]). Domesticated MULE transposases are present in other eukaryotes, having most notably given rise to the FAR1 domain-containing FAR1 and FHY3 transcription factors involved in regulating light signaling in *Arabidopsis* [76],[77]. Within ciliates, the FAR1 domain is specific to *Oxytricha* and is encoded on proteins that both contain (Contig14154.0.g51) or lack (Contig18814.0.g95) MULE domains. The dinoflagellate *Perkinsus marinus*, a sister clade to the ciliates [133], contains 21 MULE domain-containing proteins, just one of which also contains a predicted SWIM domain (C5KSG6_9ALVE), like *Oxytricha* (Contig14154.0.g51), and no proteins with the FAR1 domain. The DDE catalytic motif is present in some of the *Oxytricha* MULE domains, at a similar spacing to that found in other MULE domains [78], suggesting that these proteins are functional. We also found two MULE matches (independent E-value<1e-6) in six-frame translations of contigs from a preliminary *Euplotes* genome assembly.

We identified multiple short matches to the DDE_Tnp_IS1595 domain in a preliminary macronuclear genome assembly of *Stylonychia*, a species with gene scrambling, and this domain is also present in *Perkinsus* (C5KB61 and C5KQ59). We found no matches to this domain in six-frame translations of our preliminary *Euplotes* genome assembly. This domain is rarely present in eukaryotes: HMMER3 searches of the current UniProt database (2011_06) identified it in 20 eukaryotic species (compared to hundreds of bacterial species). Seven of the 11 DDE_Tnp_IS1595 *Oxytricha* proteins possess the transposase “DDD” catalytic residues.

Intriguingly, both the DDE_Tnp_IS1595 and MULE domain proteins are almost exclusively expressed during conjugation (peaking at 40 h after the onset of conjugation, with little or no transcription in fed and 0 hour cells; Figure 9), but later than micronuclear-limited TBE transposases (with peak expression at 24 h after the onset of conjugation [30]).

Interestingly, the Pfam domain for the *Oxytricha* TBE transposases, DDE_3, is found in both *Tetrahymena* (e.g., TTHERM_00227320) and *Paramecium* (e.g., GSPATP00034752001) proteins, and in ORF1 of the *Euplotes* Tec transposon (GenBank accession: AAA62601). Curiously, the *Tetrahymena* DDE_3 protein is upregulated in the early stages of *Tetrahymena* conjugation (http://tfgd.ihb.ac.cn/search/detail/gene/TTHERM_00227320). On the other hand, the DDE_Tnp_1_7 domain that is characteristic of the domesticated *piggyBac* [79] transposases found in *Tetrahymena* [73] and *Paramecium* [72] is absent in *Oxytricha*. Only one other transposase-like domain—HTH_Tnp_1—was detected in *Tetrahymena* and *Paramecium* but not *Oxytricha*. All of the DDE transposase domains we have characterized in *Oxytricha*, including the MULE domain, belong to a Pfam domain clan known as RNase_H (CL0219).

We found one other *Oxytricha*-specific transposon-associated domain—the Phage_integrase domain (PF00589). This domain belongs to a Pfam clan that is distinct from the RNase_H clan called DNA-mend (CL0382). This domain was also detected in the *Euplotes* Tec transposon ORF2 protein (GenBank accession: AAA62602), which is a similar length to the *Oxytricha* protein (479 aa versus 487 aa, respectively), but the two proteins are very divergent (14% identity; aligned with MUSCLE with default parameters). Both proteins have the typical catalytic residues of phage integrases/tyrosine recombinases. The phage integrase protein appears to be transcribed at very low levels in the developmental time points we examined, hence we cannot establish how its pattern of expression changes during development.

Two of the key unsolved questions about *Oxytricha* genome rearrangements is which enzymes are responsible for DNA processing and which motifs they recognize. In ciliates, lineage-specific transposon recruitment [80] may have introduced the machinery for DNA excision. *Oxytricha*'s Tc1/*mariner*-family transposases (encoded by TBE transposons) [81] were presumably acquired independently from other known ciliate transposons [80] and have a functional role in genome unscrambling [30]. One possible explanation for the apparent absence of distinct DNA excision signals in *Oxytricha* may be the involvement of multiple enzymes. While some DNA cutting may be provided by micronuclear-encoded transposases, such as TBE transposase, the functions can be macronuclear-encoded too, as in *Paramecium*'s and *Tetrahymena*'s PiggyMac transposases [72],[73]. Hence, the two developmentally expressed classes of transposase-like proteins in *Oxytricha* (with either MULE or DDE_Tnp_IS1595 domains) that we report here and that are absent from oligohymenophoreans offer new candidate proteins to supply some of the functions required for genome rearrangement.

#### Reverse transcriptases

Other than the *Oxytricha*-specific DNA transposase domains, we inspected all weaker transposase domain Pfam matches to *Oxytricha* proteins by eye to determine whether any of these weaker domain matches were also shared by *Tetrahymena* and *Paramecium* but found no evidence of additional transposase domains. We also searched for reverse transcriptase domains (i.e., RVT_1, RVT_2, and RVT_3) associated with retrotransposons, but aside from the reverse transcriptase domain (RVT_1) of the telomerase protein (Contig1013.1.g89), only one protein has one of these reverse transcriptase domains—an RVT_3 domain (Contig17363.0.g63; this protein's gene was transcribed in all the developmental time points we examined, with only small changes in expression—<7×—across time points). Likewise, *Paramecium*, *Tetrahymena*, and *Ichthyophthirius* have few proteins with either DNA transposase domains or reverse transcriptase domains (see http://trifallax.princeton.edu/cms/raw-data/gene_annotation/pfam_annotations/other_ciliates/). This means that, like the oligohymenophorean macronuclear genomes, the *Oxytricha* macronuclear genome may be almost entirely devoid of transposons.

#### Telomere binding protein paralogs

In Table S5, three paralogous proteins with the TEBP_beta domain (PF07404) appear to be *Oxytricha*-specific. The TEBP_beta domain defines the well-characterized *Oxytricha* telomere end-binding protein beta (TeBP-β), the binding partner of telomere end-binding protein alpha (TeBP-α; which has the Pfam domain Telo_bind—PF02765) [27],[82]. Both TeBP proteins are comprised of multiple oligonucleotide/oligosaccharide/oligopeptide-binding (OB)-folds (reviewed in Horvath 2008 [82]). The *Oxytricha* TeBP-β paralogs have diverged considerably from one another; the two most similar paralogs, TeBP-β1, the original TeBP-β (Contig22260.0.g8; 387 aa), and TeBP-β2 (Contig11834.0.g57; 422 aa), are 29.3% identical, while the N-terminal, TEBP_beta domain-containing portion (400 aa) of the third paralog, TeBP-β3, a 1,399 aa protein (Contig1486.1.g68), is only 17.4% and 19.1% identical to TeBP-β2 and TeBP-β1, respectively (all pairwise alignments produced by MUSCLE with default parameters). The extreme divergences between these paralogs suggest that they have been evolving rapidly. We detected no other domains in the TeBP-β3 ∼1,000 aa C-terminal extension and using this region as a query retrieved no BLASTP hits with statistically significant matches in GenBank (E-value<10). A notable feature of the TeBP-β3 C-terminal region is a long glutamic acid/glutamine-rich region, ∼400 aa over the protein interval 450–850, with 71 glutamic acid residues (27 of which are in repeats of ≥3 aa) and 58 glutamine residues.

Since the Pfam TEBP_beta domain hidden Markov model is currently based on the alignment of three seed sequences from *Oxytricha trifallax*, *Oxytricha nova*, and another close relative, *Stylonychia mytilus*, our ability to detect homologs of this domain in other organisms is limited, and may be the reason we have failed to detect this protein in *Tetrahymena* and *Paramecium*. A putative human homolog of this protein (TPP1) was proposed based on protein threading onto *Oxytricha* TeBP-β [83] (TeBP-β1—Contig22260.0.g8). A homolog of this protein (Tpt1) has also been proposed for *Tetrahymena* (corresponding to the N-terminus of the *Tetrahymena* protein prediction TTHERM_00523050) [84]. Clearly, if the human and *Tetrahymena* proteins are homologs of *Oxytricha* TeBP-β, they are extremely divergent and hence suggest that TeBP-β's may generally be evolving rapidly. We also detected a *Euplotes* TeBP-β homolog—the first in *Euplotes*—both by HMMER3 searches of *Euplotes* ORFs and TBLASTN searches of the *Oxytricha* TeBP-β's versus our *Euplotes* macronuclear genome assembly (encoded on contig388729 of our *Euplotes* assembly; HMMER3 independent E-value 1.1e-31).

*Oxytricha*'s predicted macronuclear-encoded proteome also contains six paralogous TeBP-α proteins with the Telo_bind domain. The primary TeBP-α (TeBP-α1) in *Oxytricha* specifically recognizes telomeric repeats [85]. This domain is also the characteristic domain of mammalian/fission yeast POT1 and yeast CDC13 [86],[87]. Multiple homologs of TeBP-α/POT1 have been reported in *Arabidopsis* (three paralogs [88]) and Mouse (POT1a and POT1b [89]) as well as the ciliates *Tetrahymena* (POT1a [90]—TTHERM_00378990 and POT1b [91]—TTHERM_00378980) and *Euplotes* [92] and are typically functionally differentiated. *Tetrahymena*'s POT1a is an essential gene that regulates telomere length and prevents a DNA damage response [90], while its POT1b is upregulated during conjugation and localizes in the developing macronucleus during chromosome fragmentation [91]. Though we failed to detect the Telo_bind motif in the *Tetrahymena* TeBP-α homologs in our HMMER3 searches, there was a convincing match in *Paramecium* (GSPATP00001065001; independent E-value = 2.5e-17). This suggests that these proteins are too divergent in *Tetrahymena* to have been detected. The *Euplotes* TeBP-α paralogs may be functionally differentiated to bind to either the shorter macronuclear or longer micronuclear telomeres, which share the same telomeric repeat [92],[93]. HMMER3 searches of translated *Euplotes* ORFs also identified a third TeBP-α paralog in *Euplotes crassus* and one TeBP-α homolog in the *Perkinsus* proteome (C5LFS3_PERM5). TBLASTX searches of *Oxytricha* TeBP-α paralogs against a partial *Nyctotherus ovalis* macronuclear genome [94] suggest that it also has multiple TeBP-α paralogs (corresponding to *Nyctotherus* contigs AM893732, AM892595, and AM893843 in GenBank). Hence, multiple TeBP-α paralogs may be common in spirotrichs and other ciliates with nanochromosomes.

**Figure 10:**
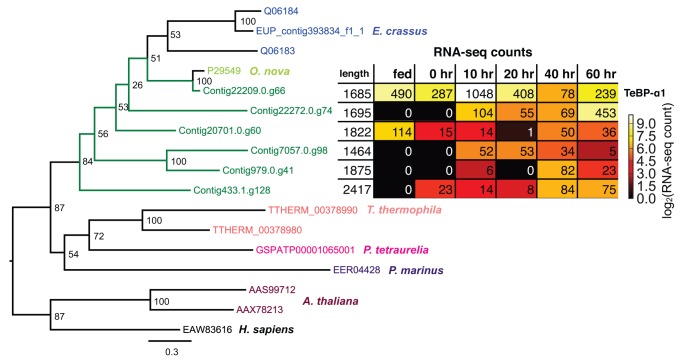
Telomere end-binding protein-α paralogs in ciliates. The phylogeny is an ML tree generated by PhyML [123] with a single substitution rate category and the JTT substitution model, optimized for tree topology and branch length. Bootstrap percentages for 1,000 replicates are indicated at the tree nodes. The multiple sequence alignments underlying the phylogeny were produced with MAFFT (v 6.418b [124]) (default parameters; BLOSUM 62 substitution matrix) and were trimmed with trimal1.2 [125] with the “-automated1” parameter to remove excess gaps and poorly aligned regions. GenBank accessions are provided for the taxa unless otherwise indicated. *Euplotes crassus* is indicated in blue (Q06184 and Q06183), and an additional match from our preliminary *Euplotes* genome assembly is EUP_contig393834_f1_1. *Perkinsus marinus* is purple (EER00428) and *Oxytricha nova* is light green (P29549). *Tetrahymena thermophila* (salmon color) accessions are from the *Tetrahymena* genome database [126]—TTHERM_00378980 and TTHERM_00378990; *Paramecium tetraurelia*'s TeBP-α protein (pink) is from ParameciumDB [127] (GSPATP00001065001). All the nodes beginning with “Contig” are *Oxytricha trifallax* TeBP-α paralogs (dark green) and Contig22209.0.g66 is TeBP-α1, the original TeBP-α. The tree is rooted at the midpoint of the branch between *Arabidopsis thaliana* (Pot1a—AAX78213 and Pot1b—AAS99712) and *Homo sapiens* (Pot1—EAW83616; black) and the rest of the phylogeny. Gene expression levels are normalized RNA-seq counts (see Text S1; Supporting Materials and Methods) before (“fed”) and during conjugation (0–60 h) are shown for the *Oxytricha trifallax* TeBP-α paralogs; coding sequence lengths are also indicated (in bp) for each of these paralogs.

Like the TeBP-β, *Oxytricha*'s TeBP-α paralogs are also extremely divergent (average 21% identity, aligned with MUSCLE using default parameters). The extreme divergence among *Oxytricha* TeBPs is consistent with the rapid evolution of OB-fold proteins in general (reviewed in Horvath 2008 [82]). The TeBP-α paralogs appear to have originated from an independent duplication after the divergence of the common ancestor of the oligohymenophoreans and the spirotrichs (Figure 10). The *Euplotes* paralogs and the first *Oxytricha* TeBP-α that was discovered (TeBP-α1—Contig22209.0.g66) appear monophyletic, but the branching of the *Euplotes* TeBP-α clade within *Oxytricha* TeBP-α paralogs is not well supported (with a bootstrap value of 51%). The extreme divergences and independent duplications of some of the *Oxytricha* TeBP-α paralogs suggest they may perform very different functions from the other ciliate TeBP-α paralogs. Since the highly fragmented macronuclear genome architecture appears to be a polyphyletic trait [95], future determination of the distribution of TeBP paralogs in nonspirotrichous ciliates with highly fragmented genomes will be relevant to our understanding of ciliate evolution.

If these additional telomere end-binding proteins bind as dimers, like TeBP-α1 and TeBP-β1 [27],[82], different combinations of TeBP-β/α partners may be possible, since there are fewer TeBP-β paralogs than TeBP-α paralogs. In total there may be up to 18 (3×6) TeBP-α/β complexes. Clearly, there are far more paralogs of these proteins than would be necessary to simply differentiate between micronuclear and macronuclear telomeres. *Oxytricha* mitochondrial chromosomes also have telomeres, though they have a very different, longer repeat unit [65], but none of the TeBP paralogs has a predicted mitochondrial signal peptide as judged from Mitoprot [96] and Predotar [97] predictions, so it is unlikely that they cap mitochondrial telomeres.

Our RNA-seq data show that the first TeBPs that were discovered (TeBP-α1 and TeBP-β1) are dominant in all the time points we examined, but the overall pattern of expression of the different TeBP paralogs during conjugation is complex. Most of the TeBPs are transcribed at all stages during conjugation (10 h and beyond). Three of the TeBP-α paralogs appear to be conjugation specific (Contig22272.0.g74, Contig7057.0.g98, and Contig979.0.g41), while the remainder of the TeBP paralogs are expressed in fed and 0 h cells in addition to later time points during development (Figure 10). The two *Oxytricha* TeBP-α paralogs expressed in fed cells (Contig2209.0.g66 and Contig20701.0.g60) are more closely related to each other than to the other four TeBP-α paralogs (Figure 10). Interestingly, all six TeBP-α paralogs are expressed at similar levels at the 40 h conjugation time point, and one of the conjugation-specific TeBP-α paralogs (Contig22272.0.g74) is about twice as highly expressed as TeBP-α1 during later conjugation (60 h).

Since *Oxytricha*'s micronuclear chromosomes terminate with the same repeats as nanochromosomes [98], it is possible that some TeBPs may have specialized for either macronuclei or micronuclei, as proposed for *Euplotes* [92]. However, given that there are multiple possible sets of macronuclear/micronuclear TeBP complexes, could some of these protein complexes have acquired different functions? *Euplotes* TeBPs appear to be important determinants of transcription initiation specificity [99]. Transcription termination is also likely to be affected by TeBPs, since many transcripts have polyadenylation sites close to the site of telomere addition (median distance ∼25 bp from the TAS). Telomere-TeBP complexes may also serve a role in DNA organization: nanochromosomes may be locally structured as “rosettes,” with nanochromosomes looping out of a central telomere-TeBP protein core [2],[100], and TeBPs appear to be key components of telomere-matrix interactions involved in large-scale macronuclear DNA organization [101],[102]. The synthesis of RNA templates that guide nanochromosome development may also be affected by the presence of TeBPs since these templates may also incorporate telomeric repeats [103].

### Additional Results

For additional results, see Text S1, Figures S1–S30 and Tables S1–S28.

### Conclusion and Future Directions

The unique architecture of the *Oxytricha* macronuclear genome expands our perspective on the limits of genome organization. We summarize our main findings below.

- The *Oxytricha* macronuclear genome assembly is comprised of ∼16,000 complete two-telomere capped nanochromosomes that vary from 469 bp to 66 kb long (mean ∼3.2 kb), encode ∼18,500 genes, and correspond to a haploid genome size of approximately 50 Mb.
- Despite the potential range of possible nanochromosome lengths (0.5–66 kb), 90% of nonalternatively fragmented nanochromosomes still encode single genes.
- The macronuclear genome inherits considerable diversity from the micronuclear genome, in the form of abundant nucleotide polymorphisms (SNP heterozygosity is ∼4.0%). Additional macronuclear genome variation arises during and after nuclear development, through a complex interplay between genetic and epigenetic forces, resulting in modest variation in DNA amplification levels and TASs, as well as thousands of alternative nanochromosome isoforms.
- A substantial fraction of nanochromosomes are homozygous (∼42%), suggesting that the JRB310 wild isolate may have been substantially inbred prior to laboratory cultivation. Possible allelic assortment may contribute to the high levels of homozygosity.
- Alternative macronuclear chromosome fragmentation, which occurs in ∼10% of nanochromosomes, rarely disrupts genes, though there is variation in the exact fragmentation position. Typically a single alternative fragmentation site gives rise to just one of two possible shorter nanochromosome isoforms. In the most extreme case of alternative fragmentation, the longest isoform is approximately 8 kb long with eight distinct protein-coding regions that yield up to 13 additional shorter isoforms.
- Overall, nanochromosome copy number is nonuniformly distributed, with modest variation compared to RNA expression levels, and very little overamplification of nanochromosomes for even the most highly expressed genes, such as those encoding ribosomal proteins, rRNA (∼56× the mean copy number for 18S rRNA) or tRNAs. While high amplification of particular nanochromosomes may have biological importance, DNA copy number, and hence gene dosage, may be less likely to regulate gene expression, since nanochromosome copy number can wildly fluctuate during prolonged cellular growth.
- The macronuclear genome encodes all the genes required for vegetative growth, consistent with the observation of amicronucleate *Oxytricha* species capable of vigorous growth in laboratory conditions [2],[66].
- Two newly discovered, developmentally expressed classes of domesticated transposase-like proteins are potentially unique to *Oxytricha*, relative to *Tetrahymena* and *Paramecium*. These *Oxytricha* proteins possess MULE and DDE_Tnp_IS1595 domains and, like TBE transposases [30], might supply DNA cutting or pasting functions in genome rearrangement.
- Other than its nanochromosome architecture, the most remarkable feature of the *Oxytricha* macronuclear genome is the absolute preponderance of the nanochromosomes' telomeres, and thus telomere biology likely plays central roles in both normal cellular growth and macronuclear development. Given the abundance of telomeres in the macronuclear genome, it is intriguing that *Oxytricha* has acquired multiple telomere end-binding protein paralogs—with six for TeBP-α and three for TeBP-β.

The *Oxytricha* macronuclear genome now enables both comparative genomics in the same cell with its micronuclear precursor, as well as comparative macronuclear genomics with other species that possess a nanochromosome architecture and more divergent model organisms with no or less genome fragmentation. Broader taxonomic sampling of other ciliate macronuclear and micronuclear genomes will greatly enhance evolutionary studies of nuclear development and genome rearrangement. In conjunction with the macronuclear genome, transcriptome data provide the first tantalizing glimpses into sweeping cellular changes during nuclear development and merit more investigation. Specific protein studies will be necessary to identify key genome rearrangement players from the extensive candidate list of development-specific genes. To facilitate these and other studies, the *Oxytricha* macronuclear genome, which is available both in GenBank (AMCR00000000) and at oxy.ciliates.org, will continue to incorporate future refinements in the genome assembly, gene predictions and annotations.

## Materials and Methods

### Cell Culture, Macronuclear DNA Isolation, and Genomic Library Construction

Briefly, to obtain macronuclear DNA for Sanger and 454 sequencing, *Oxytricha trifallax* strain JRB310 was cultured in inorganic salts medium according to an established protocol [104] with *Chlamydomonas reinhardtii* and *Klebsiella oxytoca* as food sources. The JRB310 cells we used here are likely to have undergone less than 200 divisions since they were originally isolated and have been raised from cultures with two intervening encystments. *Oxytricha* cells were harvested by filtering through several layers of gauze to remove large particles, and then a 15 µm Nitex membrane was used to concentrate cells and remove bacteria and small contaminants.

The harvested cells were washed by low-speed centrifugation through a 0.25 M sucrose solution, then lysed in 0.25 M sucrose and 0.5% Nonidet P-40. This lysis disrupts the cell membrane, leaving nuclei intact. Nuclei were then spun through 0.25 M sucrose twice to remove bacteria, mitochondria, and other cell debris. Most micronuclei were also removed in this process. DNA was extracted using the AquaPure genomic DNA isolation kit (Bio-Rad) following the manufacturer's protocol.

To obtain pure macronuclear DNA for Illumina sequencing, *Oxytricha trifallax* strain JRB310 was cultured in inorganic salts medium and starved for 3 d at 4°C to allow consumption of most of the food source (*Chlamydomonas reinhardtii*) in culture. Cells were harvested by filtering through several layers of gauze to remove large particles. Then, a 10 µm Nitex membrane was used to concentrate cells and remove small contaminants.

We collected a macronuclear fraction in 40% sucrose from a standard sucrose gradient centrifugation protocol designed to separate macronuclei and micronuclei [105]. We then purified the macronuclei an additional time, by passing them through a 70% sucrose gradient at 12,000 rcf for 10 min. DNA was isolated from the macronuclei with a NucleoSpin tissue DNA isolation kit (Machery-Nagel) according to the standard protocol for cultured cells and then RNAse A treated prior to preparation of the Illumina libraries. Since considerable streaking of the DNA was evident from electrophoretic gels, excess salt was suspected in the samples and so the DNA was precipitated in ethanol (>8 h) at 4°C, then centrifuged at 16,000 rcf for 30 min, and washed twice, for 10 min in 70% ethanol, before resuspension in the kit's elution buffer.

Genomic shotgun libraries were prepared for different size fractions of *Oxytricha* macronuclear DNA using standard methods employed at The Genome Institute (TGI) for both Sanger and 454 sequencing (see Text S1, “Preparation of Nanochromosome DNA for Sanger/454 Sequencing” to “454 Sequencing of DNA from Nanochromosome Size Fractions”), while a special method was developed for the construction and 454 sequencing of paired telomeric ends (Text S1, “Whole Nanochromosome Telomere-Based Library Construction”). Both PE and SE Illumina libraries were prepared at Princeton University from pure JRB310 Macronuclear DNA using standard Illumina kits (Text S1, “llumina Genomic Library Construction and Sequencing”).

### Genome Assembly

We developed a meta-assembly method (Figure S1) to build a reference genome assembly that is primarily derived from Illumina sequence data (Princeton Illumina assembly) but also takes advantage of an earlier Sanger/454 hybrid assembly (TGI 2.1.8 assembly). The data that were used for each of the assemblies are summarized in Table S8.

#### TGI 2.1.8 Sanger/454 assembly

In total we obtained ∼900 Mb of ABI3730 Sanger reads from whole genome shotgun (WGS) sequencing of *Oxytricha* macronuclear DNA (Tables S8, S9, S10). To coassemble Sanger and 454 data, the PCAP (2.x series assemblies) [106] and Newbler (6.0–9.0 assemblies) assemblers were used. Four hundred and fifty-four reads were added to the PCAP assemblies with the add454Reads.perl script from Consed [107],[108]. The Newbler assemblies coassembled Sanger and 454 reads. The PCAP assemblies were improved by Consed's autoedit utility and manually edited after visual inspection of the assemblies in Consed. The final TGI assembly (2.1.8) was produced by manually merging the 2.1.7 PCAP assembly and Newbler 9.0 contigs and scaffolds. We removed a small quantity of vector sequences not masked in the raw Sanger reads from the 2.1.8 assembly (∼5.4 Mb from 138 Mb) using cross_match (-minmatch 10 -minscore 15) [109] postassembly. Both the PCAP and Newbler assemblies are available at http://trifallax.princeton.edu/cms/databases/raw-data/genome/mac/assembly/WUGSC/ and additional notes about the production of these assemblies are at http://trifallax.princeton.edu/cms/raw-data/genome/mac/assembly/WUGSC/README.txt.

#### Princeton Illumina assembly and TGI Sanger/454 assembly integration

We selected high-quality reads ≥100 bp long for assembly, trimmed by TQS_fastq.py (from the SSAKE package [37]) with the parameters -t 20 (phred quality threshold) -c 40 (min number of bases passing quality threshold). Quality trimming produced 4.8 Gb of lllumina GAIIX SE reads and 6.2 Gb of Illumina HiSeq2000 PE reads (mean outer paired distance 362 bp, SD = 52 bp).

Only PE reads were assembled with the PE-Assembler [36] version “pe_asm_hg18” (default parameters) and IDBA version 0.18 (default parameters and –mink = 60 –maxk = 90 –scaffold) [35], as neither of these assemblers was designed to use a mixture of PE and SE reads. Since the PE sequence coverage was poor over a ∼160 bp span, ∼100 bp from the end of the nanochromosomes, due to the size selection procedure used to create the Illumina PE library (e.g., Figure S8), we extended the PE-Assembler contigs with SE reads using SSAKE (v3.7) [37] in TASR mode [110]. Extension of the PE-assembler contigs yielded only a modest improvement, from 20 full-length nanochromosomes to 741, and from 999 single-telomere contigs to 3,933. We assembled both the SE and PE reads with ABySS version 1.2.7 (parameters: k = 50, *n* = 10; parameters were chosen to maximize the number of full-length nanochromosomes in the assembly) [34]. We split the scaffolds created by ABySS, where spans between the contigs were unresolved (i.e., filled with “N” characters; affecting approximately ∼10% of the nanochromosomes; typically with one unresolved span per scaffold), since the presence of these spans hinders subsequent meta-assembly.

We produced a meta-assembly from the three assembly programs by assembling them with the CAP3 assembler with strict overlap parameters (-o 40 -p 99) (Figure S1) [39]. After the meta-assembly with CAP3, there was a large fraction of incompletely assembled nanochromosomes (Table S11) and so we developed a strategy of two successive end-extensions and re-assembly to increase the proportion of complete nanochromosomes (Figure S1, Table S11–S17). We attempted to extend every non-telomere-bearing contig end by finding 100 bp contig or read BLAST matches to the ends of the contigs from each of the original assemblies. In the following order of priority, we extended the contigs with three different data sources: (a) contigs from the CAP3 Illumina meta-assembly, (b) contigs from the 454/Sanger assembly (2.1.8 assembly), and (c) high-quality, vector filtered Sanger sequence reads. In the case of the 454/Sanger contigs and Sanger reads, we may have created hybrid contigs with JRB510 polymorphisms, rather than the desired JRB310 polymorphisms, but this is mitigated by the application of the majority rule during meta-assembly with CAP3 since three Illumina JRB310 assemblies were used versus one Sanger/454 JRB310/510 assembly. To integrate the contig extensions from the three different assemblies, the longest end-extension for each possible end was selected.

The contigs used for extensions were selected based on the highest identity match (≥94% identity). We desired a range of match identities to accommodate both sequencing and assembly errors as well as allelic rate variation. This resulted in a fraction of “quasi-allelic” contigs when closely related alleles were merged. The end extension conditions we chose rely on the assumption that there are relatively few close paralogs in the *Oxytricha* macronuclear genome and that close paralogs are typically more divergent than the most divergent alleles (at a <94% identity cutoff). This assumption is supported by the lack of evidence of extensive nanochromosomal paralogy in key gene families that we carefully inspected, in particular the *Oxytricha* tRNAs, which have few paralogs in *Oxytricha*, but many paralogs in the less fragmented macronuclear genomes of *Tetrahymena* and *Paramecium*.

We chose the extension identity cutoff of 6% pairwise identity after examining the fraction of validated telomeric ends in the end-extended assembly (Figure S10). As the fraction of validated ends decreases when we relax the match identity, there may be an increasing rate of chimeric extensions that we attempted to avoid. The majority of contigs have extensions with high similarity matches (Figure S10) and so the match threshold was chosen to permit co-assembly of alleles but prevent excessive nonallelic, chimeric extension. Since the extensions at higher difference thresholds were a small fraction of the total extensions (e.g., most extensions were for identical matches) and the fraction of potential incorrect extensions was small, the fraction of potential chimeras was minimized.

End extension introduced redundancy into our assembly, which was collapsed by further assembly. We reassembled the extended contigs under strict conditions (99% identity, 50 bp overlaps), and then repeated the end extension and assembly (Figure S1). Since chimeric contigs may be produced both by the initial assemblers and by our custom assembly approach, we split contigs wherever there was potential chimerism—that is, wherever PE reads did not span part of the contig (Figure S1; “spans” are inter-PE-read regions, excluding the reads themselves). Illumina PE reads were mapped to the second last assembly, by gmapper from the SHRiMP 2.1.1b [111] distribution, with default parameters, in PE mode (“-p opp-in”). Sanger mate pairs were mapped with gmapper in unpaired mode and were then filtered to select read pairs, where each of the two read matches had an edit distance ≤0.06. The mapped Illumina and Sanger read pairs were combined before detecting missing spans. We did not split contigs with missing spans 400 bp from either end of the contig, as the size selection procedure we employed in the Illumina DNA-sequence library construction eliminated most of the sequence coverage in a 160 bp span starting at 100 bp upstream of telomeres. Approximately 9% of the contigs were split across the spans missing in the mapped reads. Next, we trimmed back the split ends of the contigs by 200 bp, as the precise site of the chimerism was unknown. We also trimmed off contig ends with no mapped PE reads (2.3% of the contigs). The split, trimmed contigs were then coassembled with the unsplit contigs by CAP3 using stringent assembly parameters (overlap ≥100 bp and 99% identical) to produce our final assembly.

### Read Mapping and Variant Detection

gmapper version 2.1.1b [111] was used to map reads to the final genome assembly in SE mapping mode with default parameters, and then filtered to contain read pairs that had both members matching with ≥94% identity to the assembly (further details about the read mapping are provided in Text S1, “Read Mapping Rationale”).

To identify potential heterozygous sites that were hidden by the majority rule applied in calling contig consensi during assembly, we identified SNPs (base substitutions and not indels) at positions with ≥20× read coverage and ≥5% frequency for telomere-masked, PE reads mapped to nanochromosomes both with (“matched”) and without (“matchless”) non-self BLAT matches (≥100 bp and ≥90% identical; default parameters), from VarScan (version 2.2.8, with a minimum variant frequency of 0.001) [112] output processed by a custom Python script. We pairwise aligned heterozygous “matched” nanochromosomes with MUSCLE (default parameters; for nanochromosome pairs where one of the nanochromosomes is no more than 10% longer than the other) and estimated heterozygosity for these nanochromosomes for alignments that were ≤15% identical. SNP data can be obtained from http://trifallax.princeton.edu/cms/raw-data/genome/mac/assembly/combined_assembly/snps/varscan_snps.tar.gz/view.

SNP heterozygosity at 4-fold synonymous sites was determined from 649 coding sequence pairs, corresponding to 1,298 matched nanochromosomes, aligned with MACSE (with parameters “-Xmx1000 m” and “-d 6”) [113] with no more than 5% gaps in each of the aligned sequences.

### Detection of Alternative Nanochromosome Fragmentation and Prediction of Nanochromosome Isoforms

After splitting out and removing the adaptors used in the circular telomere-capturing constructs (see Text S1, “Whole Nanochromosome Telomere-Based Library Construction” and Figure S28 for the procedure used to produce these constructs), we selected all 454 PE reads with telomeric sequence repeats on either end and hard-masked all the telomeric repeats (matching the regular expression [AC]*CCCCAAAACCCC) with a single “N,” then selected all pairs where both reads were ≥30 bp long (719,566 pairs in total) and were terminated by telomeric repeats. We then mapped all the 454 telomeric PE reads to our final genome assembly with gmapper version 2.0.2 [111] in paired mode and the following parameters: -r 50; -p col-bw; -I 0,30000 (-r was set to 50 to accommodate the high indel error rate of 454 reads). Illumina telomeric reads, masked in the same manner as the 454 telomeric reads, were mapped with gmapper version 2.1.2b with default parameters, then filtered so that both members of the pair were ≥94% identical to the contig to which they mapped. Next we identified both 5′ and 3′ TASs for each contig in 200 bp windows around sites with maximal telomeric read coverage (this provides a lower bound estimate of the number of TASs, since these sites may span at least a couple hundred bases). Predicted sites are available at http://trifallax.princeton.edu/cms/raw-data/genome/mac/assembly/combined_assembly/telomere_addition_sites/. Alternative fragmentation sites were classified as strongly supported if they had ≥10 supporting Illumina telomeric reads and weakly supported if they had fewer matching reads than this (additional details about this classification are provided in Text S1, “Classification of Strongly and Weakly Supported Alternative Fragmentation Sites”).

Putative alternative nanochromosome isoforms were predicted based on 454 telomeric read pairs that provide a link between the ends of nanochromosomes, with any read pair ending 100 bp up- or downstream of each site providing a link. This provides a minimum estimate of the number of alternative nanochromosome isoforms produced by each locus and cannot predict longer alternative nanochromosome isoforms (much larger than 5 kb) due to the size selection limits on the initial sequence constructs. Predicted nanochromosome isoforms, with the number of reads supporting each isoform, can be found at http://trifallax.princeton.edu/cms/raw-data/genome/mac/assembly/combined_assembly/454_alt_forms.txt/view.

### Nanochromosome Copy Number Estimation

Relative nanochromosome copy numbers were estimated for nanochromosomes ≥1,800 bp long, from the total number of telomereless paired reads mapped in the intervening, nonsubtelomeric interval 600 bp from either end of each nanochromosome (http://trifallax.princeton.edu/cms/raw-data/genome/mac/assembly/combined_assembly/copy_number/copy_num_sam_filter6.nonsubtelomeric.txt/view). We excluded these subtelomeric regions, since the experimental protocol used to generate the reads lead to uneven coverage (e.g., see Figure 6 and Figure S8), which may in turn lead to poor estimates of nanochromosome copy number for shorter nanochromosomes. The total number of mapped reads was normalized by the total nanochromosome length minus the combined 1,200 bp subterminal interval. We also estimated relative copy number from the number of telomeric reads mapped to each nanochromosome (http://trifallax.princeton.edu/cms/raw-data/genome/mac/assembly/combined_assembly/copy_number/copy_num_sam_filter6.teloreads.unrestricted.txt/view).

### RNA-Seq, RNA-Seq Mapping, and Gene Prediction

RNA was isolated for five developmental time points (0, 10, 20, 40, and 60 h postmixing of JRB310 and JRB510 cells for conjugation) and used to create RNA-seq libraries with the Ovation RNA-Seq System (NuGEN Technologies, Inc. San Carlos, CA). Details of the RNA-seq library construction and sequencing are provided in Text S1, “RNA Isolation, NuGEN cDNA Synthesis and Illumina Sequencing.” To produce spliced mapped reads, RNA-seq data were mapped with BLAT [44] (“-noHead -stepSize = 5 -minIdentity = 92”) and then postprocessed to remove mapping artifacts (see Text S1, “RNA-Seq Mapping and Read Counting” for further details).

Gene predictions were produced by AUGUSTUS (version 2.5.5) [114],[115] using mapped RNA-seq data as “hints” for predictions (details about the training and prediction are provided in Text S1, “Gene Prediction”).

### Genome Data Availability

The final genome assembly has been deposited in GenBank with accession number AMCR00000000. Note that there are 20,162 contigs in GenBank, rather than the 22,450 reported for our final assembly, as some contigs were removed (e.g., if they were too short after vector trimming). Tables S8, S9, S10 and Text S1 provide links to the other assemblies and raw data.

### Additional Methods

For additional methods, see Text S1, Figure S28 and Tables S28, S29.

## Supporting Information

Figure S1 **Genome meta-assembly method. The meta-assembly started by reassembling the contigs produced from three different assemblers (see Materials and Methods for parameters used) with the CAP3 assembler.** Two cycles of contig extension and re-assembly were performed before splitting of potentially chimeric contigs and trimming back at the sites of potential chimerism. CAP3 was run one final time on the split/trimmed contigs.
(TIFF)

Figure S2 **Nanochromosomal variant frequencies in relation to sequence coverage.** Variant frequency distribution over all positions with detected variants for low (≥20× to <40×; blue) and high sequence coverage (≥40×; green); variant frequencies ≥40 bp from either nanochromosome end were counted to avoid possible incorrect variant calling resulting from telomeric bases that were not masked (due to sequencing errors).
(TIFF)

Figure S3 **Genome assembly redundancy analysis. Distribution of matching contigs for non-self BLAT matches (≥100 bp long) within the *Oxytricha* macronuclear genome assemblies.** The number of matching contigs, not the number of contig matches, is counted. The graphs (A–D) represent the ≥90%, ≥99%, ≥90% to <99%, and ≥95% match identity thresholds, respectively.
(TIFF)

Figure S4 **Missing pentose phosphate pathway (PPP) enzymes in ciliates.** Enzymes that are confirmed to be absent/present are highlighted in color, with enzymes that are present in *Paramecium*, *Tetrahymena* and *Ichthyophthirius*, but not *Oxytricha* highlighted in pink, and a single enzyme missing in *Paramecium* but present in *Oxytricha*, *Tetrahymena*, and *Ichthyophthirius* highlighted in light orange (deoxyribose-phosphate aldolase). The PPP image is used with permission from Kanehisa Laboratories and was obtained from KEGG (http://www.genome.jp/dbget-bin/www_bget?map00030) [128],[129]. For the sake of clarity, only the PPP pathways with ciliate enzymes are shown.
(TIFF)

Figure S5 **Southern blot analysis of Contig14329.0. Total macronuclear DNA was run on an electrophoretic gel.** Two probes were created to investigate alternative fragmentation of this contig (“gene 1 probe” and “gene 2 probe”). For the gene 1 probe, the forward and reverse primers, 257_F and 1264_R, are CAGGCCCACAACATCTTCCTTCTTTG and CCATCTAGCACTACTCCATTAAGCACAG, respectively, and for gene 2 probe, the forward and reverse primers, 1546_F and 1785_R, are CTCACAAGAAGCTCAGATGCAG and GCCTTCTCTGGCTTAACCACTG, respectively.
(TIFF)

Figure S6 **Association between nanochromosome copy number and number of telomeric reads.** (A) Hexagonal binning plot of relative nanochromosome copy number measured in reads/bp versus copy number measured in number of telomeric reads for nonalternatively fragmented nanochromosomes (nanochromosomes without strongly supported alternative fragmentation sites). (B) Hexagonal binning plot of number of 5′ telomeric reads versus 3′ telomeric reads for nonalternatively fragmented nanochromosomes. Linear regressions are indicated by dashed lines for both graphs [y = 389x+6.1 (r^2^ = 0.79) for (A) and y = 0.92x (r^2^ = 0.81) for (B)].
(TIFF)

Figure S7 **Linear regressions of relative estimates of nanochromosome copy number.** Squares (red) are total telomeric reads for each contig; triangles (green) are 5′ telomeric reads for each contig; and diamonds (blue) are total reads/nanochromosome length (bp). The *x*-axis units are values obtained from qPCR (see Table S3). Linear regressions were determined without the intercept term. For the linear regression of the qPCR estimate versus copy number (measured in terms of total reads/bp) r^2^ = 0.997 and for the regression of the qPCR estimate versus copy number (measured in terms of total telomeric reads) r^2^ = 0.999 (removal of the overamplified rDNA nanochromosome estimate has a negligible effect on r^2^). The following PCR primers were used: 28S-rDNA–GGTAAGAACCCTGGCCTTTC (forward) and ATCTGATGAGCGTGCAGTTG (reverse); TEBP-alpha–TGGCTCTGTGGATTCTGATG (forward) and ATTACGCCACCCTTGTCTTG (reverse); Xrcc-3–TCAACAATCCAGCTGCAAAC (forward) and TGCAGGTTCAGTACCCAAAA (reverse); DNA Pol-alpha–ACCATGCCTCCACTACCAAG (forward) and GTCATCCAGCATGGACCTCT (reverse); RNA-Pol-II GTCCAGGTTCGCATTTGTCT (forward) and CGCATTACCTGTGGGAGAAT (reverse).
(TIFF)

Figure S8 **Examples of overamplification of ribosomal protein-encoding nanochromosome isoforms (red) relative to the isoforms only containing nonribosomal genes (blue).** High peaks and deep troughs indicate subtelomeric sequence biases (see Materials and Methods).
(TIFF)

Figure S9 **Genes per contig or nanochromosome. Nanochromosomes are defined as contigs with TASs no more than 100 bp away from both ends of the contig (14,388 in total).** Alternatively fragmented nanochromosomes are those that are strongly supported by Illumina telomeric reads (≥10 reads per site), and nonalternatively fragmented nanochromosomes are all the remaining nanochromosomes.
(TIFF)

Figure S10 **Heat map of extended contigs verified with 454-telomeric end reads.** Axes indicate percent difference of the 100 bp end matches. The first number in each cell indicates the fraction of complete nanochromosomes with paired matches to within 50 bases of each end of the nanochromosome; the second number indicates the total number of extended nanochromosomes for the extension match percentage identity pair [e.g., (0, 0) is the entry where both left and right extensions were perfect 100 bp matches]. The last column indicates nanochromosomes with just a single extension (*) or no extension (**). The data underlying the matrix are nanochromosomes extracted from the contigs that were extended by the 454/Sanger assembly (2.1.8 assembly), initial Illumina meta-assembly, and Sanger reads, with extensions that differ up to 12%. A 6% difference cutoff was selected for the extensions used in our meta-assembly approach (Figure S1).
(TIFF)

Figure S11 **Validation of nanochromosomes from the final assembly.** Nanochromosomes were validated both by 454 telomeric end reads and/or Sanger reads/mate pairs. (A) Length distribution of nanochromosomes validated by either 454 telomeric end reads (green) or Sanger read/mate pairs (cyan) or both (purple), or not validated by either method (pink) (see Materials and Methods for match details). (B) Nanochromosome copy number (Illumina reads/contig length×fraction GC) versus nanochromosome length for validated and unvalidated nanochromosomes; linear regressions of the two data sets are plotted with dashed lines (with r^2^ = 0.002 and r^2^ = 0.051, respectively).
(TIFF)

Figure S12 **Length distribution of nanochromosomes validated by Sanger mate pairs.** Nanochromosomes were validated according to the method illustrated in Figure S11.
(TIFF)

Figure S13 **p_N_/p_S_ values for matchless nanochromosomes.** p_N_/p_S_ values were calculated by PAML (see Text S1: Determination of pN/pS values). A cut-off of p_N_/p_S_ = 0.6 is shown by the dashed red line.
(TIFF)

Figure S14 **Alternative nanochromosome fragmentation in a predicted intron-containing region.** Gene predictions for Contig17419.0 are shown. Predicted genes are indicated by green arrows and predicted CDSs by yellow arrows; predicted introns are indicate by white arrows, and those introns that are supported by RNA-seq evidence have two white arrows; neon green blocks indicate mapped RNA-seq data. The red arrow indicates an alternative fragmentation site and it points in the direction that the alternative nanochromosome isoform (isoform 2) is formed. Only part of the complete 7,380 bp nanochromosome (isoform 1) is shown.
(TIFF)

Figure S15 **Intra-CDS alternative nanochromosome fragmentation.** Nanochromosomes are indicated by black bars in descending order of length, with gene annotations below them. Predicted genes are indicated by green arrows and predicted CDSs by yellow arrows. Red arrows indicate alternative fragmentation sites and point in the direction that the alternative nanochromosome isoforms are produced.
(TIFF)

Figure S16 **Nonalternatively fragmented tRNA nanochromosomes.** Nanochromosomes are indicated by black bars in descending order of length, with gene annotations below them. Where multiple allelic versions of nanochromosomes are present, we have selected just a single representative nanochromosome. Predicted genes are indicated by green arrows, predicted CDSs by yellow arrows, and tRNAs by pink arrows.
(TIFF)

Figure S17 **Positional variation of TASs.** TASs are contig-derived (see Text S1: Determination of sequences surrounding telomere addition sites). TASs within a 200 bp window surrounding and centered on strongly supported, alternatively fragmented, and nonalternatively sites were counted. The frequency distributions of the TASs for alternatively fragmented sites are indicated in pale green and nonalternatively fragmented sites in blue.
(TIFF)

Figure S18 **Base compositional biases surrounding TASs.** Contig consensus sequences surrounding strongly supported site TASs (≥10 supporting Illumina telomeric reads) were extracted for (A–C) (see Text S1: Determination of sequences surrounding telomere addition sites). The telomere position is 0. We only illustrate base composition biases for one end of the nanochromosome since the complementary base frequencies are identical for both ends. (A) indicates the present region “upstream” of alternatively fragmented TASs (AF-TASs) that are present on the resulting nanochromosome. (B) indicates the absent region “downstream” of alternatively fragmented TASs (non-AF-TASs). (C) indicates the present region upstream of nonalternatively fragmented TASs.
(TIFF)

Figure S19 **Nanochromosome subtelomeric base composition of *Stylonychia* compared to that of *Oxytricha*.** *Oxytricha* base compositions are indicated by dots behind the *Stylonychia* base composition lines.
(TIFF)

Figure S20 **TAS sequence logos. Sequence logos showing nucleotide frequencies (generated with WebLogo [130]) for method 1 are for contig-derived sequences; while the logos for method 2 are for read-derived sequences. Sequence logos show base frequencies.**
(TIFF)

Figure S21 **Sequence logo of *Euplotes crassus* subtelomeric regions.** Sequence logos show base frequencies. Note that some of the motifs may be slightly misaligned (usually by 1 base), and hence the motif centered on position −20 would be even more prominent if they were correctly aligned.
(TIFF)

Figure S22 **Intron length distribution.** The green histogram is for all introns predicted by AUGUSTUS, including those with experimental support from RNA-seq data; the blue histogram is for all introns determined from RNA-seq data that were used as hints for AUGUSTUS during the gene prediction. The inset shows the size distribution over a longer length scale (with 5 bp bins).
(TIFF)

Figure S23 **Intron length distribution for *Tetrahymena thermophila* gene predictions.** Intron lengths determined from 2008 *Tetrahymena* gene predictions (downloaded from http://www.ciliate.org/system/downloads/oct2008_release.gff).
(TIFF)

Figure S24 **Sequence logos of experimentally determined and predicted intron donor sites.** Sequence logos generated by WebLogo show base frequencies. Experimentally determined introns were obtained from RNA-seq data (see Text S1: Determination of sequences surrounding telomere addition sites). Predicted introns are all the introns predicted by AUGUSTUS, including those that have supporting RNA-seq evidence. Sequence logos were produced for introns in 11 bp intron length windows centered on the 37, 53, and 80 bp intron length modes, and for introns longer than 150 bp. Judging from the base composition of the third sequence position, which is almost exclusively “A” in introns extracted from the RNA-seq data, ∼10% of intron predictions may be incorrectly predicted for introns 53 bp and longer. Introns ≥53 bp constitute 39% of experimentally derived introns and 62% of predicted introns.
(TIFF)

Figure S25 **Sequence logos of experimentally determined and predicted intron acceptor sites.** Sequence logos show base frequencies. Experimentally determined introns were obtained from RNA-seq data (see Text S1: Gene prediction). Predicted introns are all the introns predicted by AUGUSTUS, including those that have supporting RNA-seq evidence. Sequence logos were produced for introns in 11 bp intron length windows centered on the 37, 53, and 80 bp intron length modes, and for introns longer than 150 bp.
(TIFF)

Figure S26 **Length distributions of untranscribed (UTS) and untranslated (UTR) regions.** Length distributions are for single-gene, nonalternatively fragmented nanochromosomes. (A) 5′ UTS length from the transcription start site to telomere (determined from 5′-RLM RACE Sanger reads). (B) 3′ UTS length from polyadenylation site to telomere (determined from RNA-seq reads); the two graphs are for the site closest to the telomere (red) and the most frequently used polyadenylation site (pink); negative lengths indicate polyadenylation sites extending beyond the telomere. The 3′ UTS median length is 25 bp for the most frequently used polyadenylation sites and 19 bp for polyadenylation sites closest to telomeres. (C) 5′ UTR lengths from 5′-RLM RACE Sanger reads. (D) 3′ UTR lengths from RNA-seq reads; negative lengths indicate polyadenylation sites upstream of the stop codon; the two graphs are for the site closest to the telomere (dark gold) and the most frequently used polyadenylation site (gold). 3′ UTRs have a median length of 78 bp when the most frequently used polyadenylation sites are counted or 87 bp for polyadenylation sites closest to telomeres.
(TIFF)

Figure S27 **Assessment of potential paralogy in model ciliate genomes.** UCLUST from the USEARCH suite (version 5.1.221) [131] was used for clustering at increasing global sequence alignment identity clustering thresholds, with the query and target alignment fractions both set to 80% coverage (i.e., number of letters in the query that are aligned to letters in the target), and the parameters –maxaccepts 3 –maxrejects 128 to increase clustering sensitivity at medium sequence identity levels. The increase in number of clusters from the 95% to 100% cluster identity threshold for *Oxytricha* reflects clustering of protein alleles.
(TIFF)

Figure S28 **Subtelomeric DNA capture method for 454 subtelomeric sequencing.** Adaptor ends with a 5′-phosphate are shown in bold; otherwise 5′-phosphate is absent. The biotinylated thyamine residue in the internal adaptor is indicated in green.
(TIFF)

Figure S29 **End-to-end validation by Sanger mate pair reads.** Paired and SE reads are shown with gray arrows. First, “outer spans” between the ends of paired-end reads or consisting of the entire SE read are found. Next, we attempt to greedily find a path through the spans, so that there are ≥100 bp overlaps between the spans comprising the path. If we find such a path, the contig is considered to be validated.
(TIFF)

Figure S30 **Distribution of supporting telomeric reads per alternative fragmentation site versus reads per contig length.** Alternative fragmentation sites >400 bp from contig ends are hexagonally binned. The *x*-axis units are an estimate of relative nanochromosome copy number based on the total number of mapped Illumina reads, both telomeric and nontelomeric, per bp for each contig. On the *y*-axis, reads per fragmentation site were calculated for a 100 bp window centered on the site with the most reads supporting the alternative fragmentation site. (A) 454-telomeric end reads (2,792 contigs; 3,634 sites); (B) Illumina telomeric reads (3,331 contigs; 4,392 sites).
(TIFF)

Table S1 **Location of alternative fragmentation sites relative to inter- and intracoding sequence regions for two-gene nanochromosomes.** Alternative fragmentation sites with decreasing numbers of supporting telomeric reads are shown in three successive columns. To exclude conventional TASs, only alternative fragmentation sites at least 100 bp away from either end of the contig were selected. Nanochromosomes with single alternative fragmentation sites were selected. AUGUSTUS gene predictions were used to determine inter-/intra-CDS regions. Similar trends were found for 454 telomeric reads. %GC was determined for a 50 bp window either side of alternative fragmentation sites.
(RTF)

Table S2 **Location of alternative fragmentation sites relative to coding and noncoding sequence regions for single-gene nanochromosomes.** Alternative fragmentation sites with decreasing numbers of supporting telomeric reads are shown in three successive columns. To exclude conventional TASs, only alternative fragmentation sites at least 100 bp away from either end of the contig were selected. Nanochromosomes with single alternative fragmentation sites were selected. CDS/non-CDS regions were determined from the AUGUSTUS gene predictions. Similar trends were observed for 454 telomeric reads (not shown). %GC was determined for a 50 bp window either side of alternative fragmentation sites.
(RTF)

Table S3 **Estimates of nanochromosome copy number.** Only the rRNA nanochromosome in this table is alternatively fragmented (with a site at 634 bp supported by 11 reads and two sites at 1,253 bp and 6,077 bp supported by a single read). 5′- and 3′-telomeric reads refer to reads that are mapped either to the 5′ or 3′ end as it is oriented in the genome assembly.
(RTF)

Table S4 **Large predicted proteins.** The 20 longest nanochromosomes, excluding cases that appear to be redundant (i.e., quasi-alleles), are shown. None of these nanochromosomes is alternatively fragmented. Nanochromosome lengths include telomeres. Protein domain names are abbreviations from Pfam-A (version 26). Semicolons separate predicted protein lengths and protein domain architectures.
(RTF)

Table S5 ***Oxytricha* nucleic-acid-associated protein domains not found in *Paramecium* and *Tetrahymena*.** ^a^Judging from multiple sequence alignments, domain appears to exist in *Paramecium* (GSPATP00020413001) and *Tetrahymena* (TTHERM_00721450) but was not detected by hmmscan (HMMER3) ^b^independent E-value greater than the threshold (0.001), but domain exists (e.g., protein TTHERM_01211800 in *Tetrahymena*). ^c^Protein encoded on contigs, with no telomeric repeats, that are likely bacterial contaminants ^d^Predicted protein for an incompletely assembled part of a previously characterized mitochondrial plasmid encoding a viral/organellar type DNA polymerase [69]. ^e^Independent E-value greater than the threshold (0.001) used, but domain exists in *Paramecium* [124] and *Tetrahymena*. ^f^Independent E-value greater than the threshold (0.001) used, but sequence alignments of proteins containing N-terminal domain (TFIIA_gamma_N) to homologs from *Oxytricha* suggest that hmmscan failed to detect this domain in *Tetrahymena* and *Paramecium*.
(RTF)

Table S6 ***Oxytricha* putative nucleic-acid-associated protein domains (not annotated in pfam2go) not found in *Paramecium* and *Tetrahymena*.** Domains in this table are considered to have putative nucleic-acid-related functions based on Pfam descriptions and literature cited for these domains. ^a^Proteins encoded on contigs with no telomeric repeats. ^b^Protein is truncated due to an incorrect intron prediction; this protein is an allelic variant of the protein Contig14154.0.g51, which is the correctly predicted allelic variant. ^c^Protein encoded on contigs with no telomeric repeats that are likely bacterial contaminants.
(RTF)

Table S7 **Nucleic-acid-associated protein domains found in both *Paramecium* and *Tetrahymena* but not *Oxytricha*.** Domains marked with * are present in translated ORFs, but were not originally detected as AUGUSTUS failed to predict them. Protein IDs are given for *Tetrahymena*.
(RTF)

Table S8 **Data sources for genome assemblies.** Data for the genome assemblies incorporated in the final meta-assembly may be downloaded from http://dx.doi.org/10.5061/dryad.d1013 [132].
(RTF)

Table S9 **Genomic and RNA libraries Sanger sequenced on ABI3730 sequencers.**
(RTF)

Table S10 **454 genomic DNA libraries.** Short read archive data can be downloaded from http://www.ncbi.nlm.nih.gov/sra.
(RTF)

Table S11 **Meta-contig statistics after first CAP3 assembly before extension.** “Single” refers to an SE being complete (≥1 5′ or 3′ telomeres). “Both” refers to one or more telomeres on both ends of the contig (≥1 5′ and ≥1 3′ ends). “Multiple” refers to greater than two ends on either end of the contig (≥2 5′ or ≥2 3′ ends). All lengths are given in bp.
(RTF)

Table S12 **Meta-contig statistics after first extension.** “Single” refers to an SE being complete (≥1 5′ or 3′ telomeres). “Both” refers to one or more telomeres on both ends of the contig (≥1 5′ and ≥1 3′ ends). “Multiple” refers to greater than two ends on either end of the contig (≥2 5′ or ≥2 3′ ends). All lengths are given in bp.
(RTF)

Table S13 **Meta-contig statistics after CAP3 reassembly of extended contigs.** “Single” refers to an SE being complete (≥1 5′ or 3′ telomeres). “Both” refers to one or more telomeres on both ends of the contig (≥1 5′ and ≥1 3′ ends). “Multiple” refers to greater than two ends on either end of the contig (≥2 5′ or ≥2 3′ ends). All lengths are given in bp.
(RTF)

Table S14 **Meta-contig statistics after second extension.** “Single” refers to an SE being complete (≥1 5′ or 3′ telomeres). “Both” refers to one or more telomeres on both ends of the contig (≥1 5′ and ≥1 3′ ends). “Multiple” refers to greater than two ends on either end of the contig (≥2 5′ or ≥2 3′ ends). All lengths are given in bp.
(RTF)

Table S15 **Meta-contig statistics after CAP3 reassembly of second round of extended contigs.** “Single” refers to an SE being complete (≥1 5′ or 3′ telomeres). “Both” refers to one or more telomeres on both ends of the contig (≥1 5′ and ≥1 3′ ends). “Multiple” refers to greater than two ends on either end of the contig (≥2 5′ or ≥2 3′ ends). All lengths are given in bp.
(RTF)

Table S16 **Meta-contig statistics after chimera splitting and end trimming. “Single” refers to an SE being complete (≥1 5′ or 3′ telomeres).** “Both” refers to one or more telomeres on both ends of the contig (≥1 5′ and ≥1 3′ ends). “Multiple” refers to greater than two ends on either end of the contig (≥2 5′ or ≥2 3′ ends). All lengths are given in bp.
(RTF)

Table S17 **Meta-contig statistics for the final CAP3 assembly.** “Single” refers to an SE being complete (≥1 5′ or 3′ telomeres). “Both” refers to one or more telomeres on both ends of the contig (≥1 5′ and ≥1 3′ ends). “Multiple” refers to greater than two ends on either end of the contig (≥2 5′ or ≥2 3′ ends). All lengths are given in bp.
(RTF)

Table S18 **Properties of intergenic regions.** Prediction features were obtained for complete nanochromosomes (14,388 in total) only. Intergenic regions are between start and stop codons, including UTRs. Alternative fragmentation sites are those that are strongly supported by Illumina telomeric reads. %GC estimates exclude telomeric bases. Intergenic regions are subdivided according to whether they have a site of alternative fragmentation within the region or not.
(RTF)

Table S19 **Missing Moco biosynthesis enzymes in ciliates.**
(RTF)

Table S20 **Small ribosomal proteins.** Gene identifiers are given as contig identifiers with a gene suffix beginning with “g” followed by a number (which is arbitrary in this context). Only proteins ≤100 aa with domains found in Pfam 26.0 with an E-value<0.01 and with some homologs in UniProt that are ≤120 aa (to ensure that they are genuine small proteins) are listed. Where alternative fragmentation occurs, the nanochromosome length of the shortest putative isoform encoding the small protein is shown. Protein domains are taken from Pfam 26.0. Contig22302.0 is a multigene nanochromosome. ^a^These proteins are incorrectly predicted as gene fusions on the longer ribosomal/nonribosomal protein-encoding nanochromosome.
(RTF)

Table S21 **Small nonribosomal proteins.** Gene identifiers are given as contig identifiers with a gene suffix beginning with “g” followed by a number (which is arbitrary in this context). All nanochromosomes in this table longer than 1 kb are predicted to be multigene nanochromosomes. Proteins 50–100 aa long with domains found in Pfam 26.0 (independent E-value<0.01) and with at least some homologs in UniProt that are ≤120 aa (to ensure that they are genuine small proteins) are listed. We excluded a few proteins that appeared to be truncated by incomplete assembly of their nanochromosomes. Nanochromosome lengths exclude telomeres. Where alternative fragmentation occurs, the nanochromosome length of the shortest putative isoform encoding the small protein is shown. Protein domain names are from Pfam 26.0.
(RTF)

Table S22 **RNA-seq counts for transcription initiation factor II domain protein genes.** RNA expression values are given in normalized read counts for vegetative (“Fed”) cells and cells developing during conjugation (see Text S1: RNA-seq mapping and read counting).
(RTF)

Table S23 **Top 40 elevated domain counts in *Oxytricha* relative to *Tetrahymena*.** The “enrichment” column measures the number of UCLUST clustered proteins in *Oxytricha* relative to proteins in *Tetrahymena*. The columns after “enrichment” count the number of proteins in which the Pfam domains are found for *Oxytricha* (*Oxy*), *Tetrahymena* (*Tet*), *Paramecium* (*Par*), and *Perkinsus* (*Per*). Nucleic-acid-related domains (bold) were classified from Pfam domains annotated with the “nucleic acid binding” GO term (GO:0003676) in pfam2go. Where there were missing Pfam domain annotations in pfam2go or there was literature associated with the Pfam identifier that suggested the domain was nucleic acid binding, we also classified the protein as nucleic-acid-related.
(RTF)

Table S24 **RNA-seq counts for homeodomain protein genes.** RNA expression values are given in normalized read counts for vegetative (“Fed”) cells and cells developing during conjugation (see Text S1: RNA-seq mapping and read counting).
(RTF)

Table S25 **Zinc finger protein domain counts in *Oxytricha*.** The “enrichment” column measures the number of UCLUST clustered proteins in *Oxytricha* relative to proteins in *Tetrahymena*. The columns after “enrichment” count the number of proteins in which the Pfam domains are found for *Oxytricha* (*Oxy*), *Tetrahymena* (*Tet*), *Paramecium* (*Par*), *Ichthyophthirius* (*Ich*), and *Perkinsus* (*Per*).
(RTF)

Table S26 **RNA-seq counts for poly-adenylate binding protein domain protein genes.** RNA expression values are given in normalized read counts for vegetative (“Fed”) cells and cells developing during conjugation (see Text S1: RNA-seq mapping and read counting).
(RTF)

Table S27 **RNA-seq counts for replication protein A domain protein genes.** RNA expression values are given in normalized read counts for vegetative (“Fed”) cells and cells developing during conjugation (see Text S1: RNA-seq mapping and read counting).
(RTF)

Table S28 **Total RNA sources for poly(A)-selected mRNA.** ^a^RiboMinus Eukaryote Kit (Invitrogen, Carlsbad, CA).
(RTF)

Text S1 **Supporting Results, Materials and Methods.**
Contents of file:
*Supporting Results, p. 3*.
Macronuclear genome validation, p. 3.
Analysis of low frequency variants, p. 4.
Pfam domains detected for CEGs missing in *Oxytricha*, p. 5.
Investigation of alternative fragmentation sites in relation to mapped RNA-seq data, p. 6.
Frequent colocation of ncRNA- and protein-encoding genes, p. 8.
Examination of discrepancies between predicted and experimentally determined alternative fragmentation isoforms of the highly fragmented Contig14329.0, p. 9.
Telomere addition sites, p. 10.
Introns and untranscribed and untranslated regions, p. 13.
Gene-less contigs or nanochromosomes, p. 15.
Reads containing both putative telomeric repeats are not genuine nanochromosomes, p. 16.
Analysis of short protein and ncRNA-encoding nanochromosomes, p. 17.
Characterization of the longest predicted *Oxytricha* protein, p. 21.
The *Oxytricha* macronucleus encodes a smaller proteome than *Tetrahymena*, p. 22.
Additional differences in the nucleic acid related predicted proteomes of ciliates, p. 24.
Proliferation of nucleic-acid binding domains in *Oxytricha*, p. 26.
Differences in the non-nucleic acid related predicted proteomes of ciliates, p. 31.
*Supporting Materials and Methods, p. 35*.
Southern analysis of alternative nanochromosome isoforms, p. 35.
qPCR to estimate relative nanochromosome copy number, p. 35.
Preparation of nanochromosome DNA for Sanger/454 sequencing, p. 36.
Whole nanochromosome shotgun library construction, p. 37.
Whole genome shotgun (1.5–35 kb) library construction and sequencing, p. 37.
454 sequencing of DNA from nanochromosome size fractions, p. 38.
Whole genome shotgun (>10 kb) fosmid library construction, p. 38.
Whole nanochromosome telomere-based library construction, p. 39.
Illumina genomic library construction and sequencing, p. 43.
Read mapping rationale, p. 43.
Determination of pN/pS values, p. 45.
Genome assembly validation and redundancy analysis, p. 45.
Classification of strongly and weakly supported alternative fragmentation sites, p. 47.
Determination of sequences surrounding telomere addition sites, p. 48.
RNA isolation, NuGEN cDNA synthesis and Illumina sequencing, p. 48.
RNA-seq mapping and read counting, p. 50.
Gene prediction, p. 52.
Length determination of “untranscribed” and untranslated regions, p. 55.
Protein domain identification and GO term selection, p. 55.
tRNA searches, p. 57.
*Euplotes crassus* culturing, DNA isolation and preliminary macronuclear genome assembly, p. 57.
*Supporting References, p. 59*.
(RTF)

## Abbreviations

6PGD: 6-phosphogluconate dehydrogenase
6PGL: gluconolactonase
AF-TAS: alternatively fragmented telomere addition site
CDS: coding sequence
CEG: core eukaryotic genes
CEGMA: core eukaryotic gene mapping approach
G6PD: glucose-6-phosphate dehydrogenase
IES: internally eliminated sequence
KOGS: eukaryotic clusters of orthologous groups
K-S: Kolmogorov-Smirnov
MDS: macronuclear destined sequence
MULE: Mutator-like transposable element
OPPP: oxidative pentose phosphate pathway
ORF: open reading frame
PE: paired end
PPP: pentose phosphate pathway
qPCR: quantitative PCR
RACE: Rapid amplification of cDNA ends
RLM RACE: RNA Ligase Mediated Rapid Amplification of cDNA Ends
rRNA: ribosomal RNA
SAC: spindle assembly checkpoint
SE: single end
snoRNA: small nucleolar RNA
SNP: single nucleotide polymorphism
TAS: telomere addition site
TBE: telomere-bearing element
TeBP: telomere end-binding protein
UTR: untranslated region
UTS: untranscribed region
