# Supplementary figures and images for: The Oxytricha trifallax Macronuclear Genome: A Complex Eukaryotic Genome with 16,000 Tiny Chromosomes
Source: PLoS Biol. 2013 Jan 29;11(1):e1001473. doi: 10.1371/journal.pbio.1001473 (PMC3558436; doi:10.1371/journal.pbio.1001473)

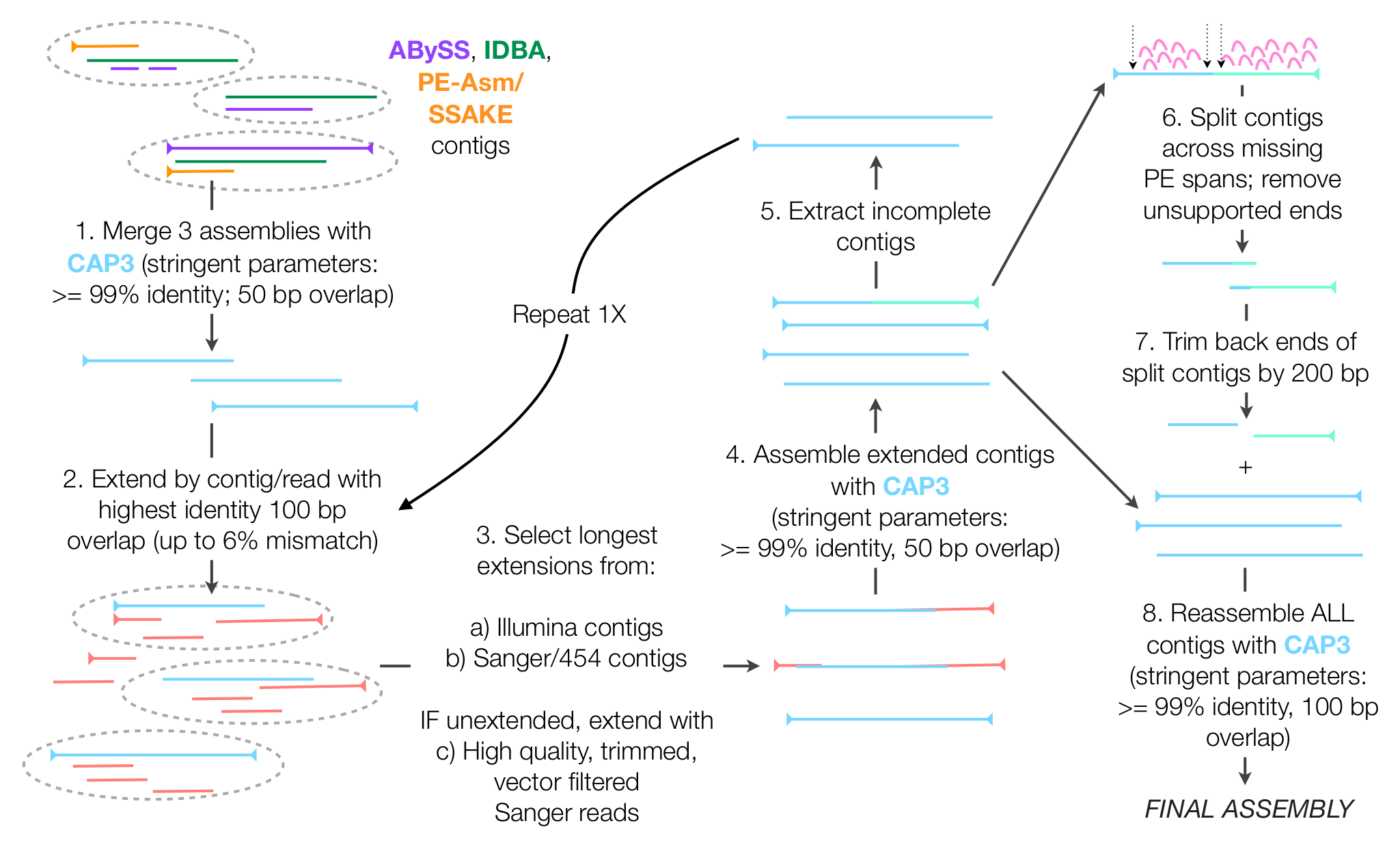

Supplement: Figure S1 — Genome meta-assembly method. The meta-assembly started by reassembling the contigs produced from three different assemblers (see Materials and Methods for parameters used) with the CAP3 assembler. Two cycles of contig extension and re-assembly were performed before splitting of potentially chimeric contigs and trimming back at the sites of potential chimerism. CAP3 was run one final time on the split/trimmed contigs. (TIFF) [file pbio.1001473.s001.tif]

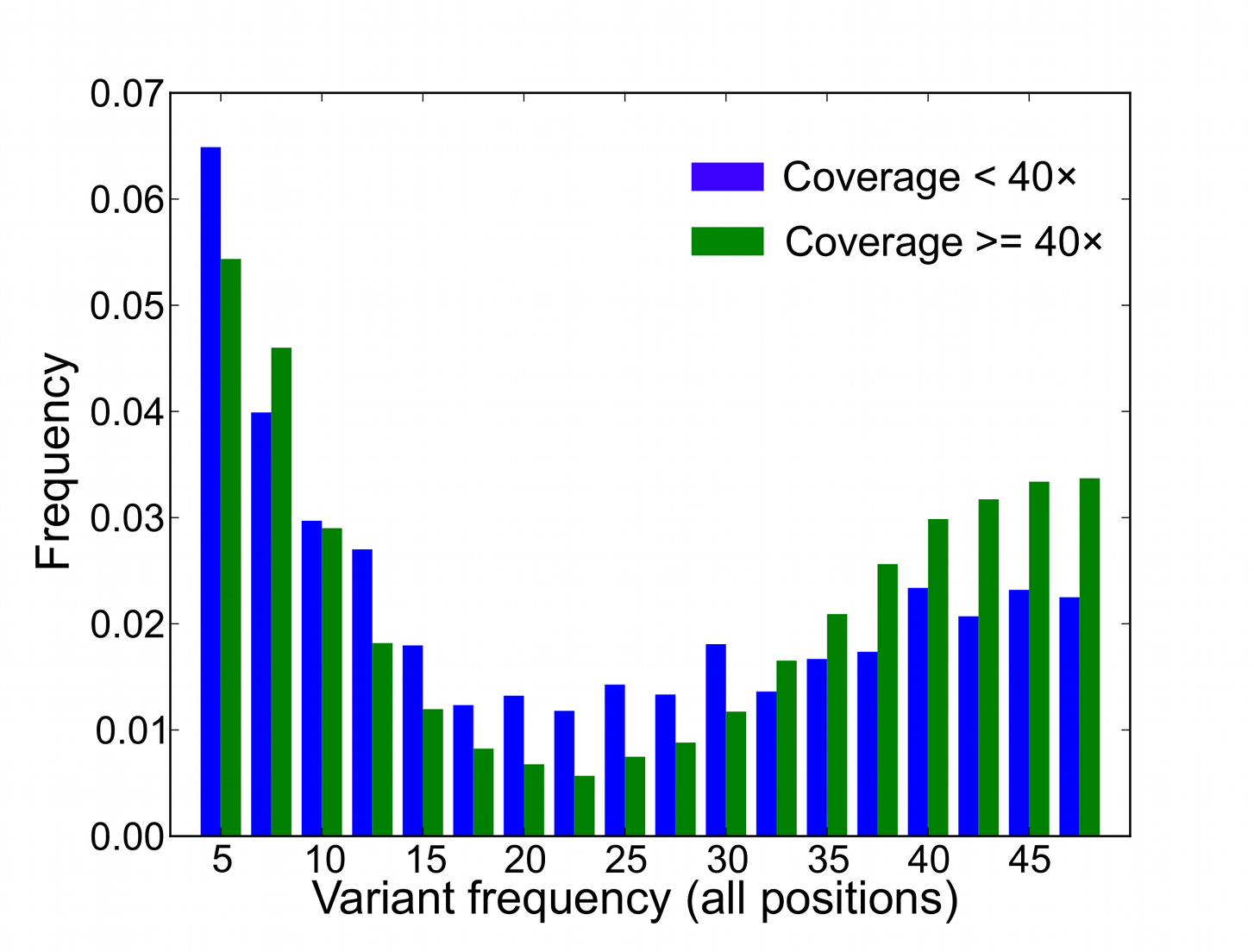

Supplement: Figure S2 — Nanochromosomal variant frequencies in relation to sequence coverage. Variant frequency distribution over all positions with detected variants for low (≥20× to <40×; blue) and high sequence coverage (≥40×; green); variant frequencies ≥40 bp from either nanochromosome end were counted to avoid possible incorrect variant calling resulting from telomeric bases that were not masked (due to sequencing errors). (TIFF) [file pbio.1001473.s002.tif]

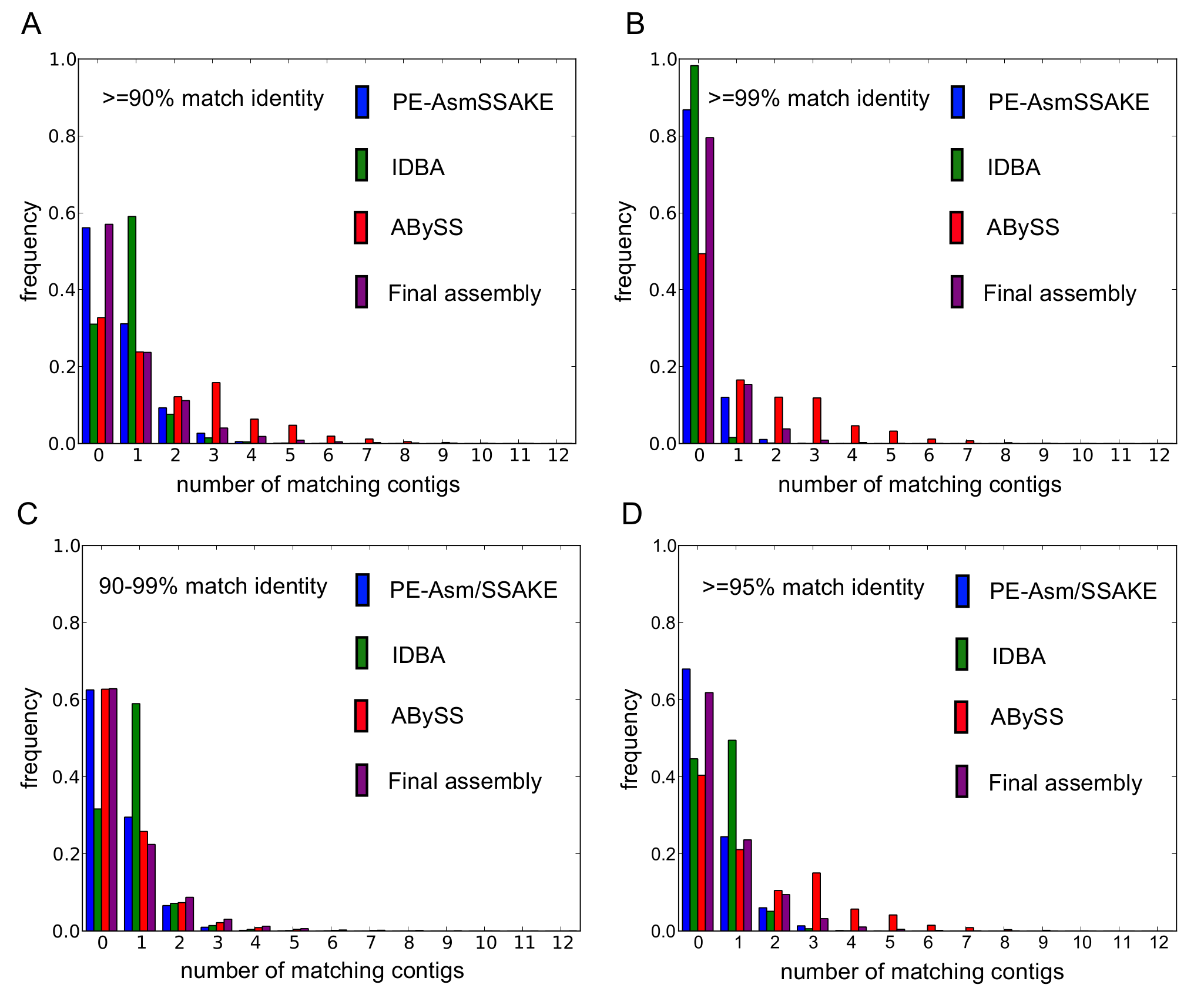

Supplement: Figure S3 — Genome assembly redundancy analysis. Distribution of matching contigs for non-self BLAT matches (≥100 bp long) within the Oxytricha macronuclear genome assemblies. The number of matching contigs, not the number of contig matches, is counted. The graphs (A–D) represent the ≥90%, ≥99%, ≥90% to <99%, and ≥95% match identity thresholds, respectively. (TIFF) [file pbio.1001473.s003.tif]

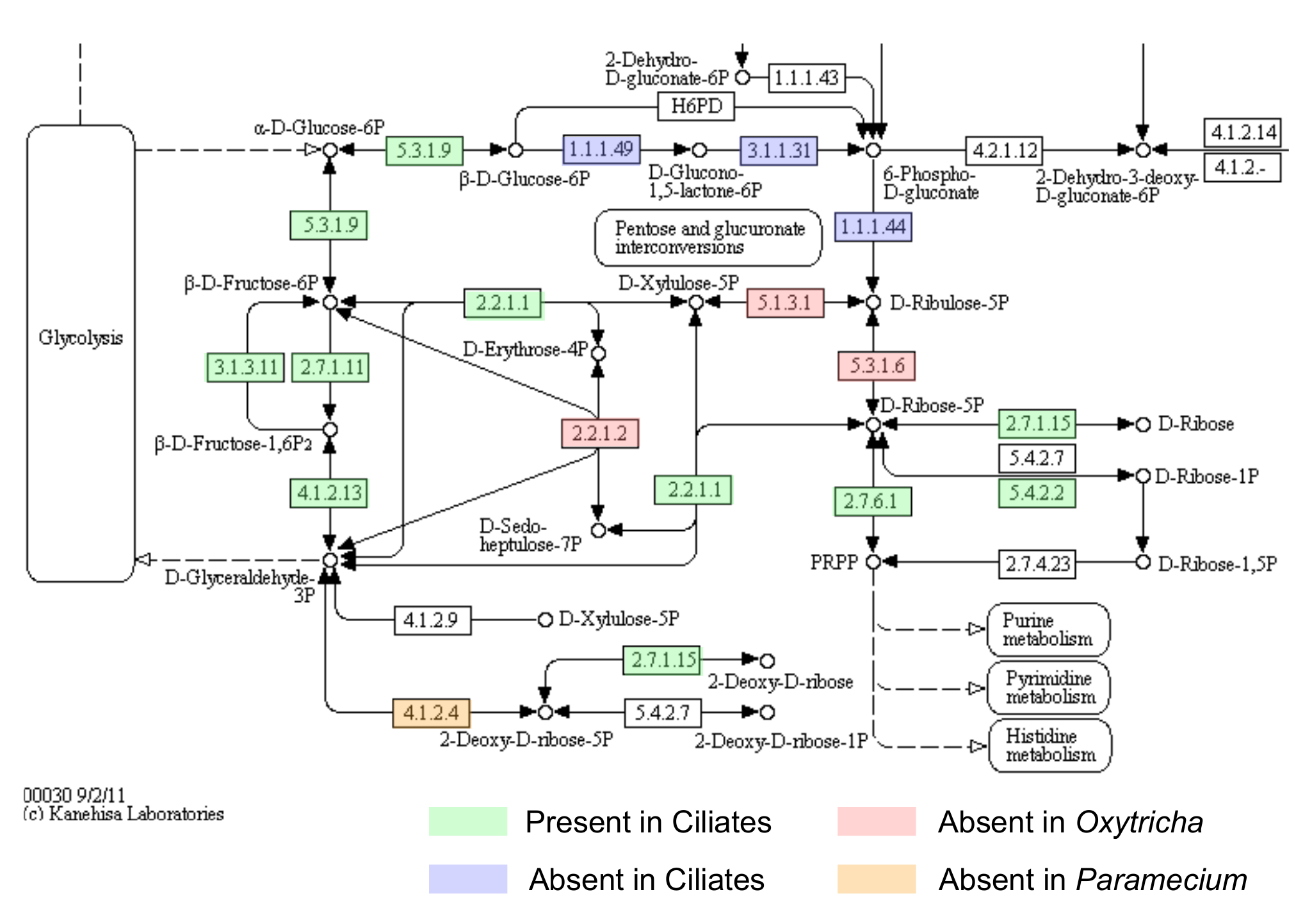

Supplement: Figure S4 — Missing pentose phosphate pathway (PPP) enzymes in ciliates. Enzymes that are confirmed to be absent/present are highlighted in color, with enzymes that are present in Paramecium, Tetrahymena and Ichthyophthirius, but not Oxytricha highlighted in pink, and a single enzyme missing in Paramecium but present in Oxytricha, Tetrahymena, and Ichthyophthirius highlighted in light orange (deoxyribose-phosphate aldolase). The PPP image is used with permission from Kanehisa Laboratories and was obtained from KEGG (http://www.genome.jp/dbget-bin/www_bget?map00030) [128],[129]. For the sake of clarity, only the PPP pathways with ciliate enzymes are shown. (TIFF) [file pbio.1001473.s004.tif]

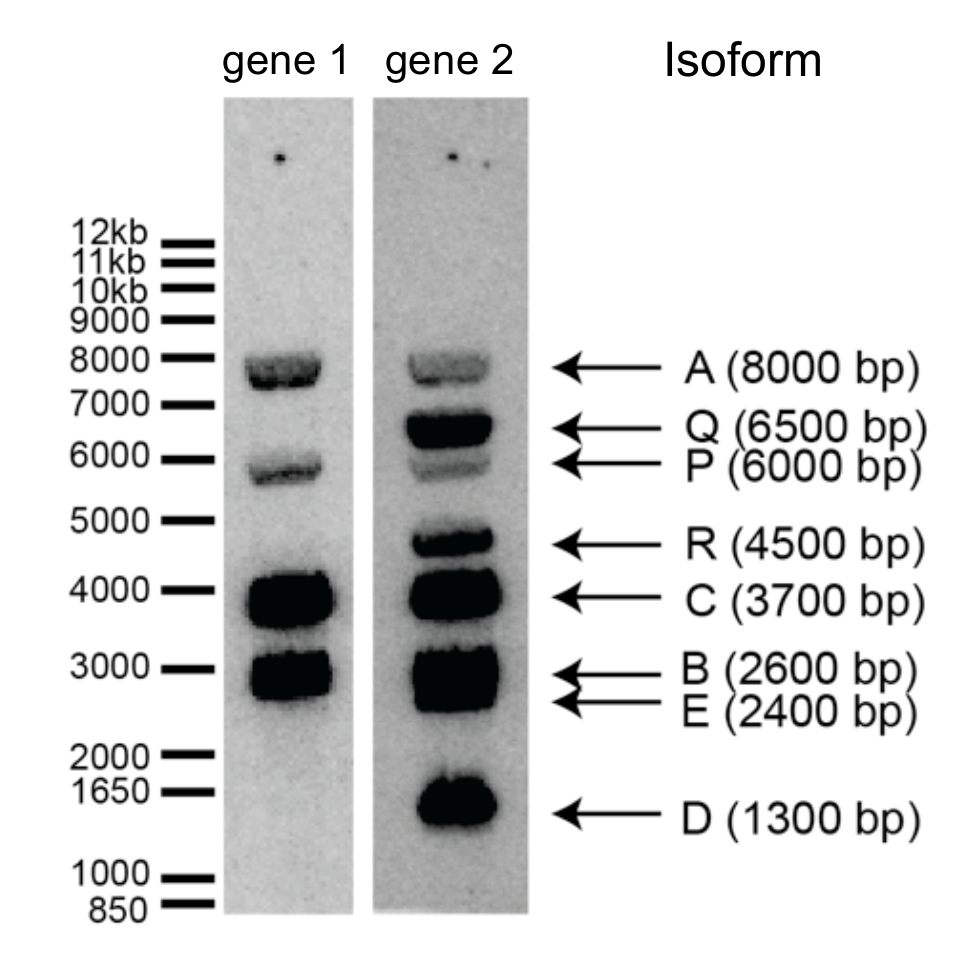

Supplement: Figure S5 — Southern blot analysis of Contig14329.0. Total macronuclear DNA was run on an electrophoretic gel. Two probes were created to investigate alternative fragmentation of this contig (“gene 1 probe” and “gene 2 probe”). For the gene 1 probe, the forward and reverse primers, 257_F and 1264_R, are CAGGCCCACAACATCTTCCTTCTTTG and CCATCTAGCACTACTCCATTAAGCACAG, respectively, and for gene 2 probe, the forward and reverse primers, 1546_F and 1785_R, are CTCACAAGAAGCTCAGATGCAG and GCCTTCTCTGGCTTAACCACTG, respectively. (TIFF) [file pbio.1001473.s005.tif]

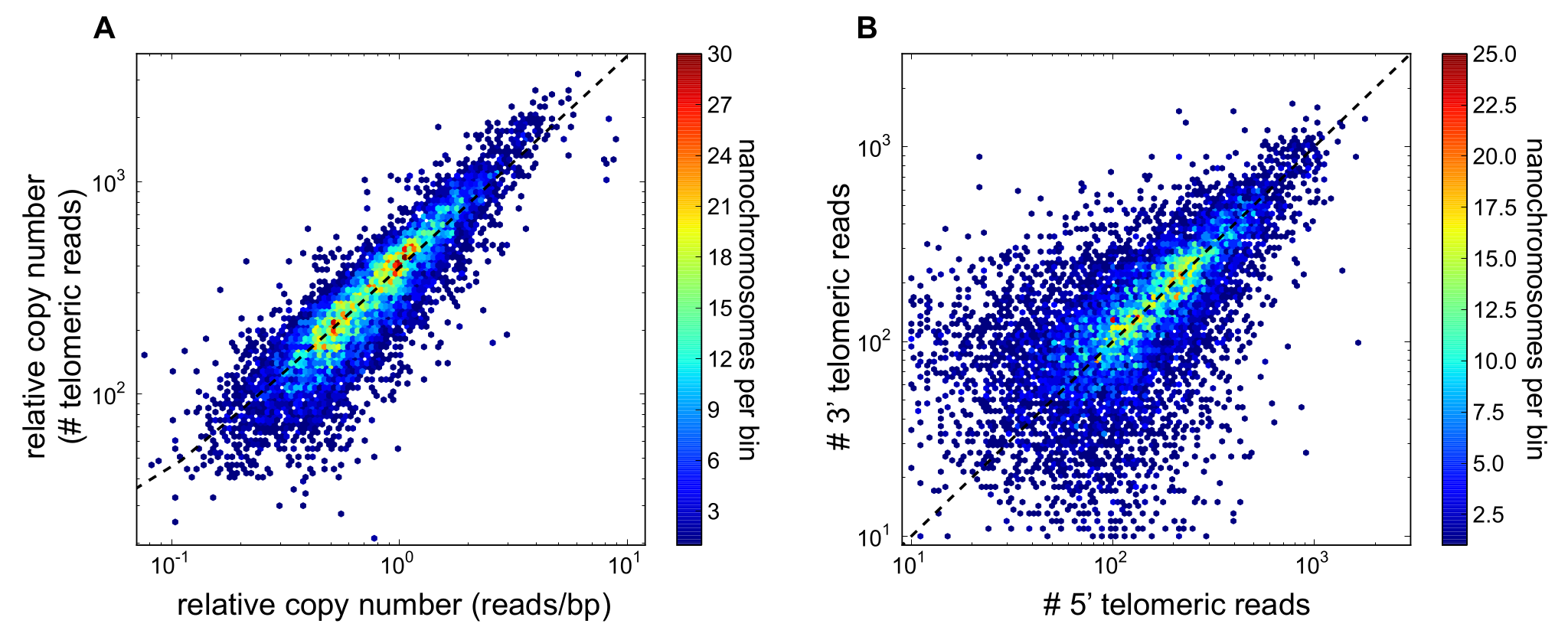

Supplement: Figure S6 — Association between nanochromosome copy number and number of telomeric reads. (A) Hexagonal binning plot of relative nanochromosome copy number measured in reads/bp versus copy number measured in number of telomeric reads for nonalternatively fragmented nanochromosomes (nanochromosomes without strongly supported alternative fragmentation sites). (B) Hexagonal binning plot of number of 5′ telomeric reads versus 3′ telomeric reads for nonalternatively fragmented nanochromosomes. Linear regressions are indicated by dashed lines for both graphs [y = 389x+6.1 (r2 = 0.79) for (A) and y = 0.92x (r2 = 0.81) for (B)]. (TIFF) [file pbio.1001473.s006.tif]

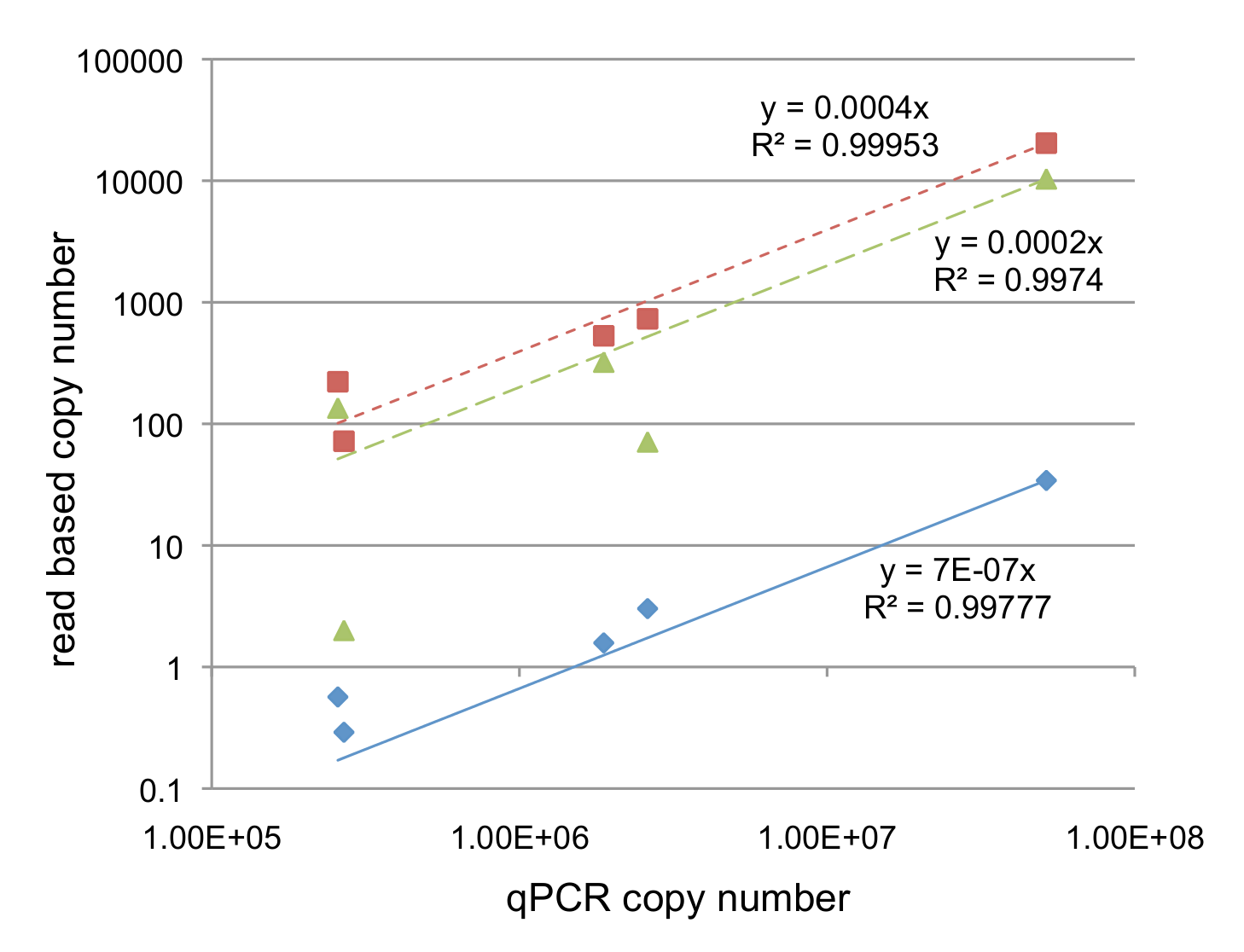

Supplement: Figure S7 — Linear regressions of relative estimates of nanochromosome copy number. Squares (red) are total telomeric reads for each contig; triangles (green) are 5′ telomeric reads for each contig; and diamonds (blue) are total reads/nanochromosome length (bp). The x-axis units are values obtained from qPCR (see Table S3). Linear regressions were determined without the intercept term. For the linear regression of the qPCR estimate versus copy number (measured in terms of total reads/bp) r2 = 0.997 and for the regression of the qPCR estimate versus copy number (measured in terms of total telomeric reads) r2 = 0.999 (removal of the overamplified rDNA nanochromosome estimate has a negligible effect on r2). The following PCR primers were used: 28S-rDNA–GGTAAGAACCCTGGCCTTTC (forward) and ATCTGATGAGCGTGCAGTTG (reverse); TEBP-alpha–TGGCTCTGTGGATTCTGATG (forward) and ATTACGCCACCCTTGTCTTG (reverse); Xrcc-3–TCAACAATCCAGCTGCAAAC (forward) and TGCAGGTTCAGTACCCAAAA (reverse); DNA Pol-alpha–ACCATGCCTCCACTACCAAG (forward) and GTCATCCAGCATGGACCTCT (reverse); RNA-Pol-II GTCCAGGTTCGCATTTGTCT (forward) and CGCATTACCTGTGGGAGAAT (reverse). (TIFF) [file pbio.1001473.s007.tif]

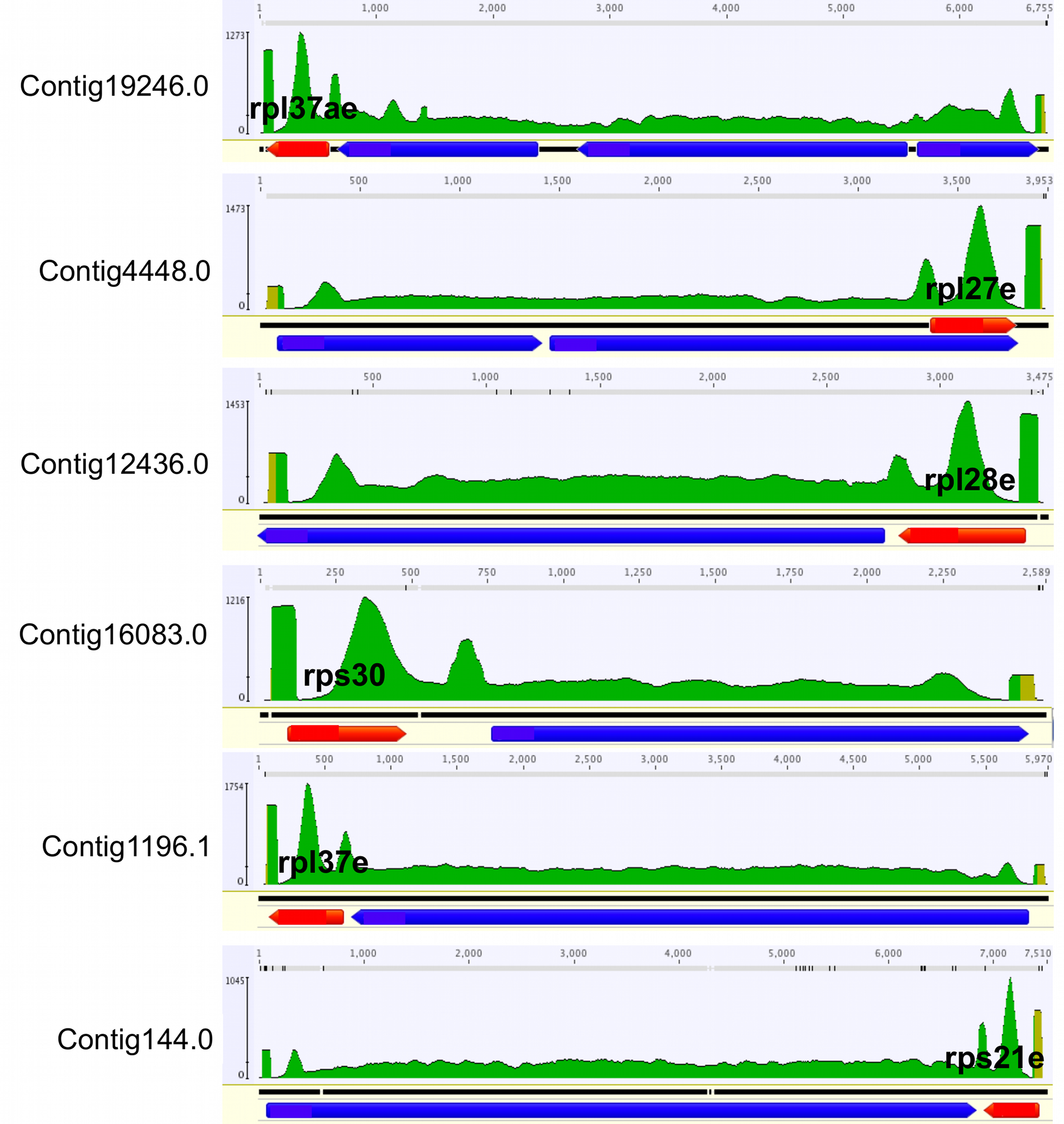

Supplement: Figure S8 — Examples of overamplification of ribosomal protein-encoding nanochromosome isoforms (red) relative to the isoforms only containing nonribosomal genes (blue). High peaks and deep troughs indicate subtelomeric sequence biases (see Materials and Methods). (TIFF) [file pbio.1001473.s008.tif]

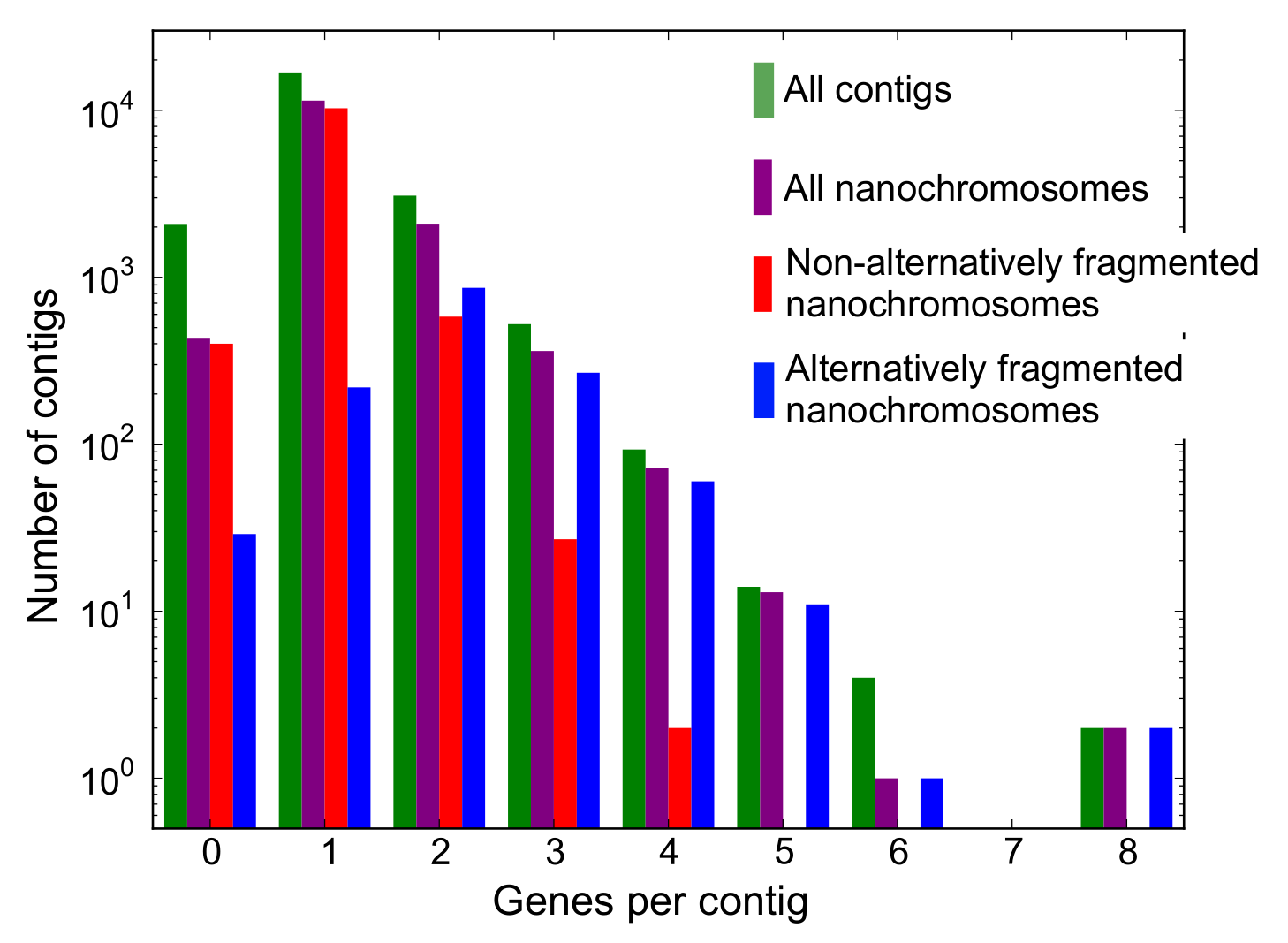

Supplement: Figure S9 — Genes per contig or nanochromosome. Nanochromosomes are defined as contigs with TASs no more than 100 bp away from both ends of the contig (14,388 in total). Alternatively fragmented nanochromosomes are those that are strongly supported by Illumina telomeric reads (≥10 reads per site), and nonalternatively fragmented nanochromosomes are all the remaining nanochromosomes. (TIFF) [file pbio.1001473.s009.tif]

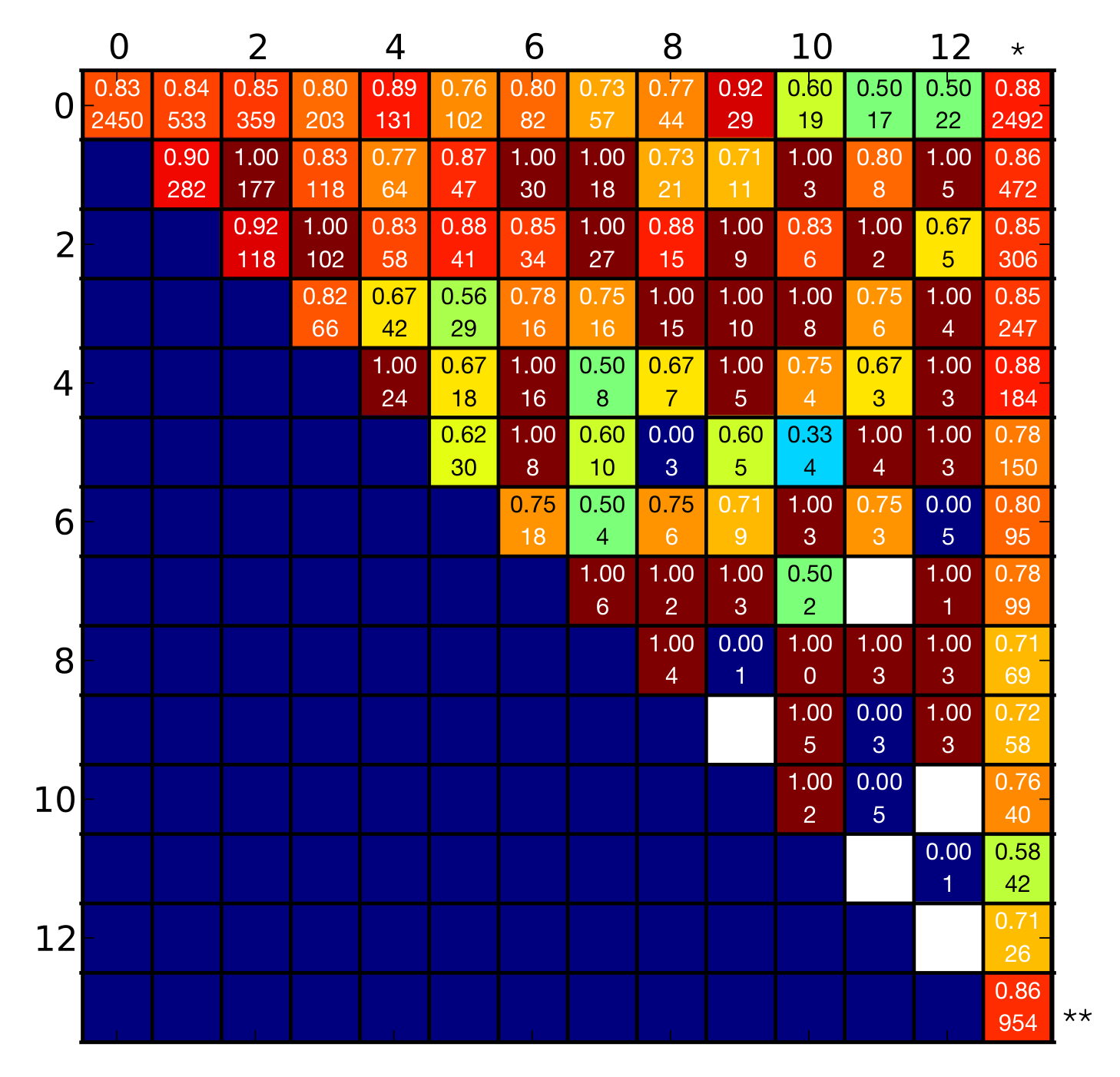

Supplement: Figure S10 — Heat map of extended contigs verified with 454-telomeric end reads. Axes indicate percent difference of the 100 bp end matches. The first number in each cell indicates the fraction of complete nanochromosomes with paired matches to within 50 bases of each end of the nanochromosome; the second number indicates the total number of extended nanochromosomes for the extension match percentage identity pair [e.g., (0, 0) is the entry where both left and right extensions were perfect 100 bp matches]. The last column indicates nanochromosomes with just a single extension (*) or no extension (**). The data underlying the matrix are nanochromosomes extracted from the contigs that were extended by the 454/Sanger assembly (2.1.8 assembly), initial Illumina meta-assembly, and Sanger reads, with extensions that differ up to 12%. A 6% difference cutoff was selected for the extensions used in our meta-assembly approach (Figure S1). (TIFF) [file pbio.1001473.s010.tif]

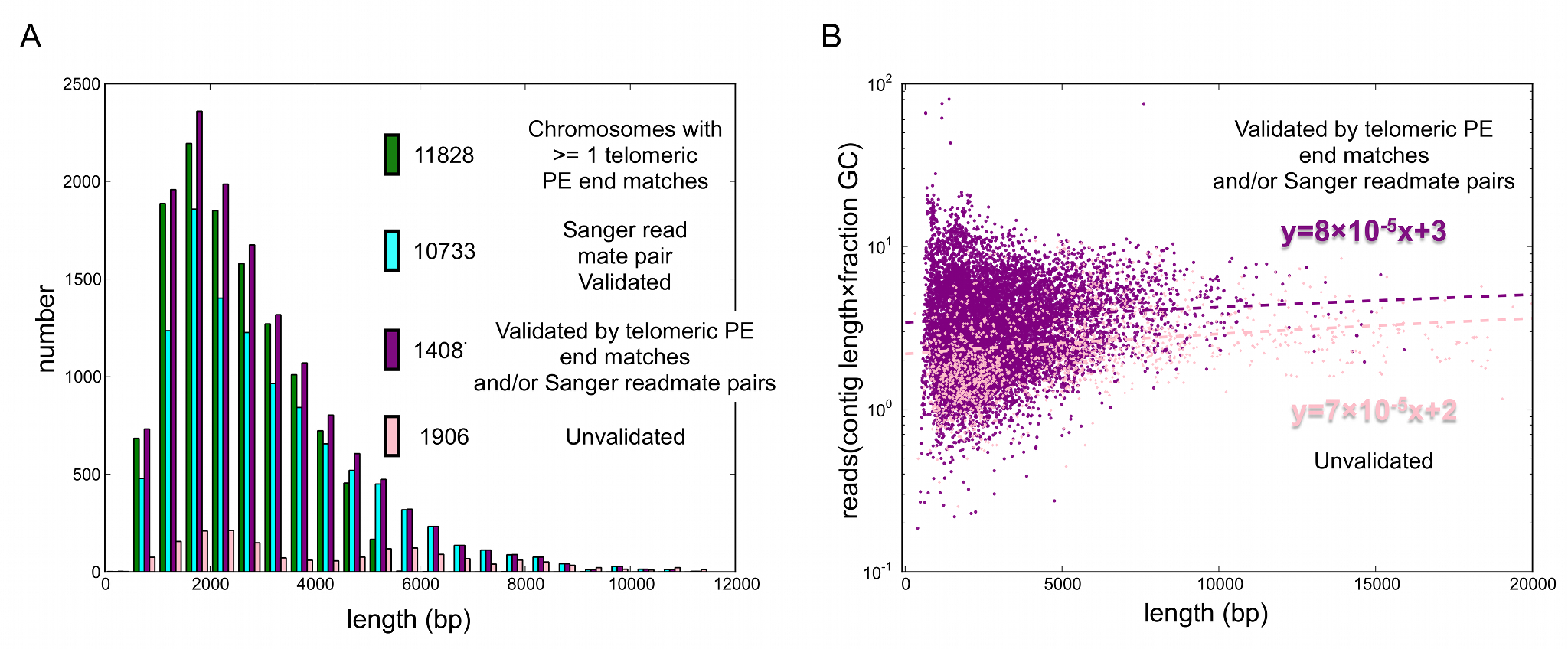

Supplement: Figure S11 — Validation of nanochromosomes from the final assembly. Nanochromosomes were validated both by 454 telomeric end reads and/or Sanger reads/mate pairs. (A) Length distribution of nanochromosomes validated by either 454 telomeric end reads (green) or Sanger read/mate pairs (cyan) or both (purple), or not validated by either method (pink) (see Materials and Methods for match details). (B) Nanochromosome copy number (Illumina reads/contig length×fraction GC) versus nanochromosome length for validated and unvalidated nanochromosomes; linear regressions of the two data sets are plotted with dashed lines (with r2 = 0.002 and r2 = 0.051, respectively). (TIFF) [file pbio.1001473.s011.tif]

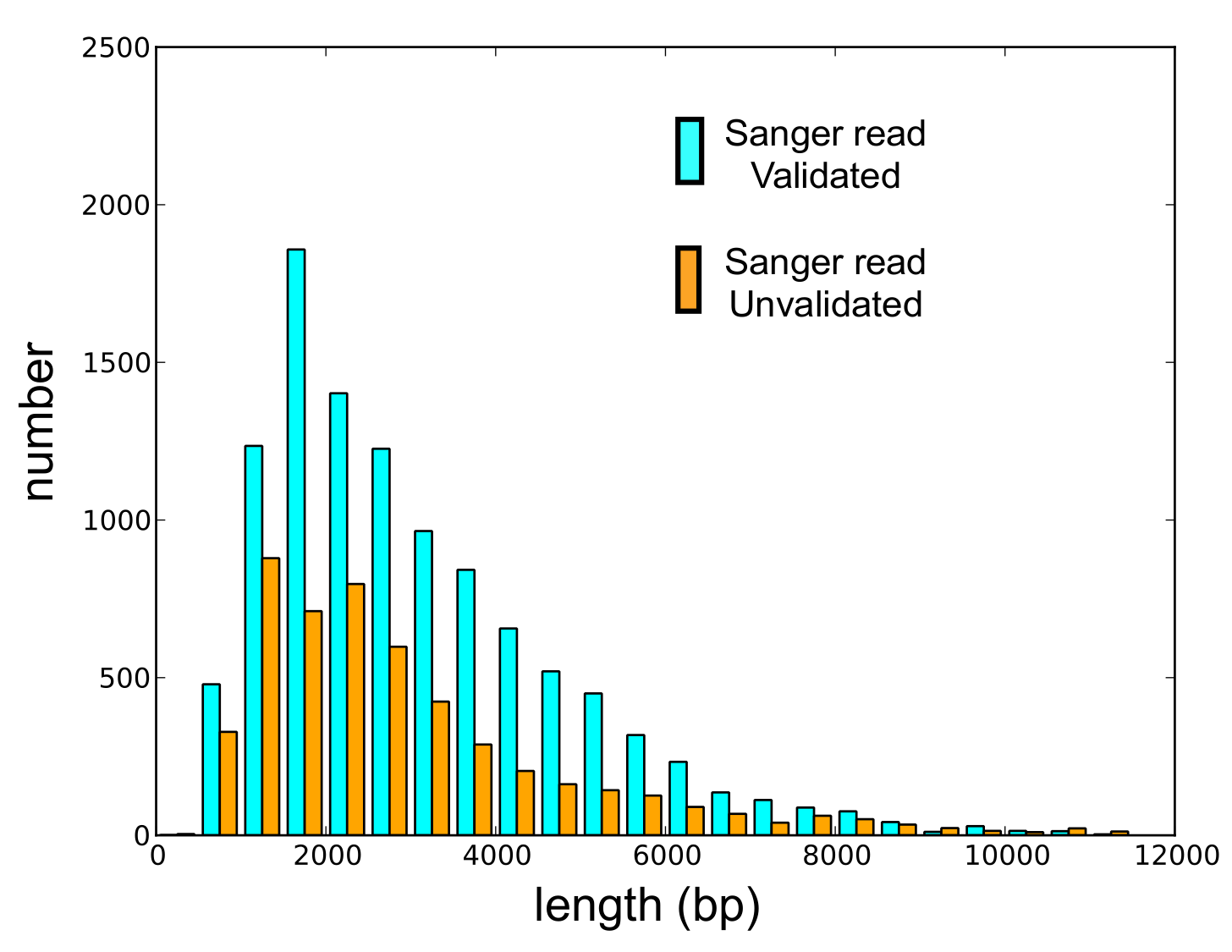

Supplement: Figure S12 — Length distribution of nanochromosomes validated by Sanger mate pairs. Nanochromosomes were validated according to the method illustrated in Figure S11. (TIFF) [file pbio.1001473.s012.tif]

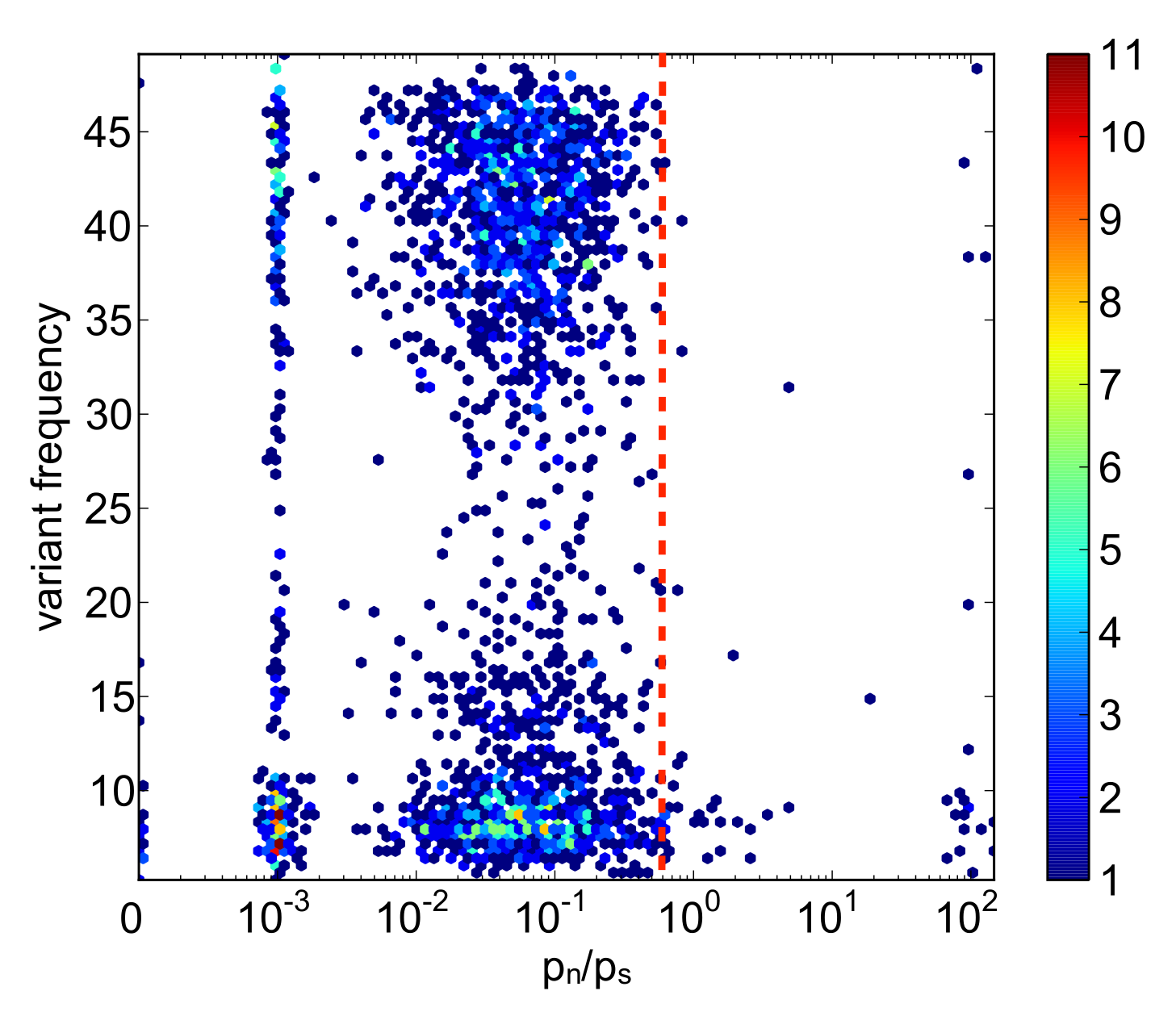

Supplement: Figure S13 — pN/pS values for matchless nanochromosomes. pN/pS values were calculated by PAML (see Text S1: Determination of pN/pS values). A cut-off of pN/pS = 0.6 is shown by the dashed red line. (TIFF) [file pbio.1001473.s013.tif]

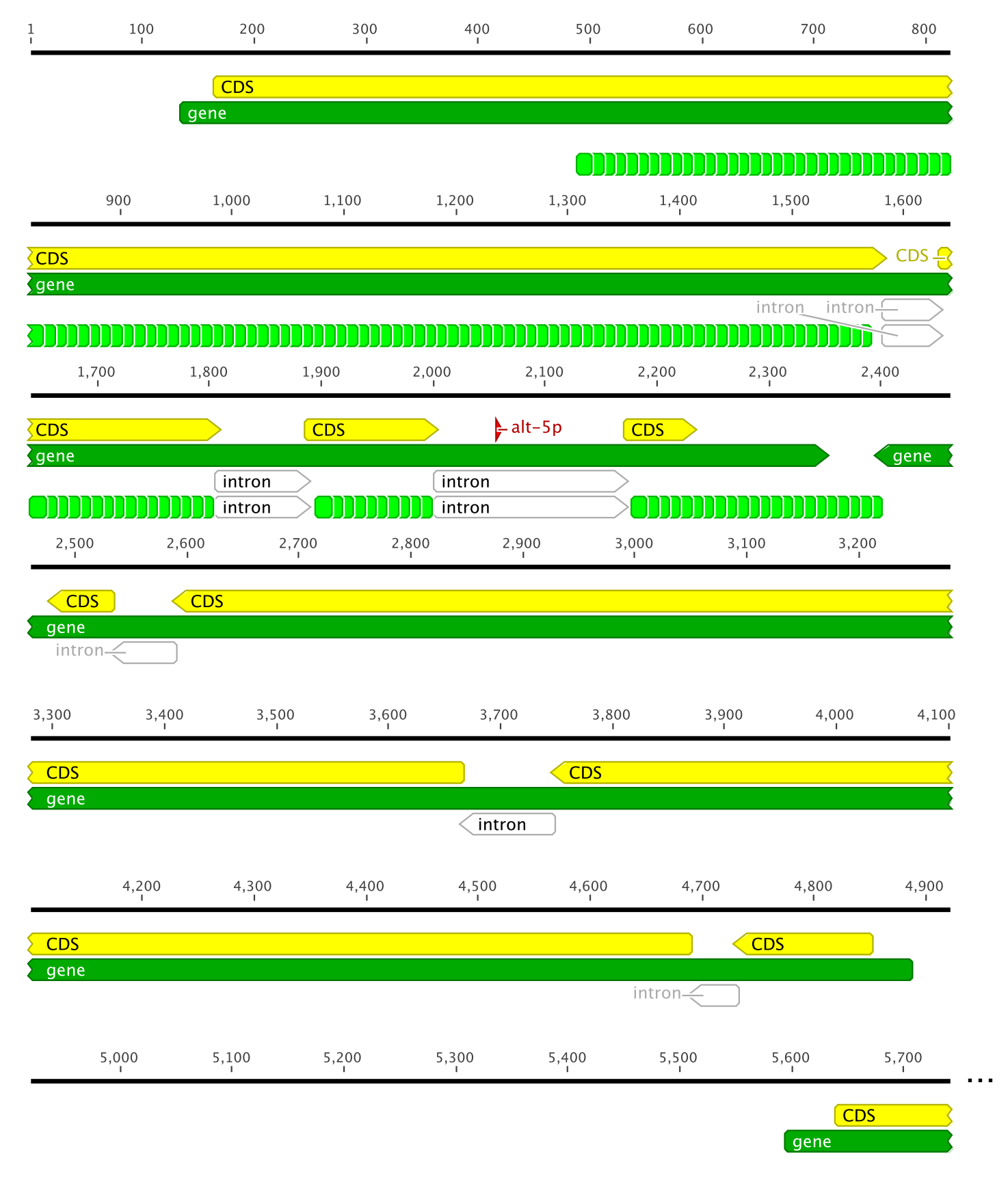

Supplement: Figure S14 — Alternative nanochromosome fragmentation in a predicted intron-containing region. Gene predictions for Contig17419.0 are shown. Predicted genes are indicated by green arrows and predicted CDSs by yellow arrows; predicted introns are indicate by white arrows, and those introns that are supported by RNA-seq evidence have two white arrows; neon green blocks indicate mapped RNA-seq data. The red arrow indicates an alternative fragmentation site and it points in the direction that the alternative nanochromosome isoform (isoform 2) is formed. Only part of the complete 7,380 bp nanochromosome (isoform 1) is shown. (TIFF) [file pbio.1001473.s014.tif]

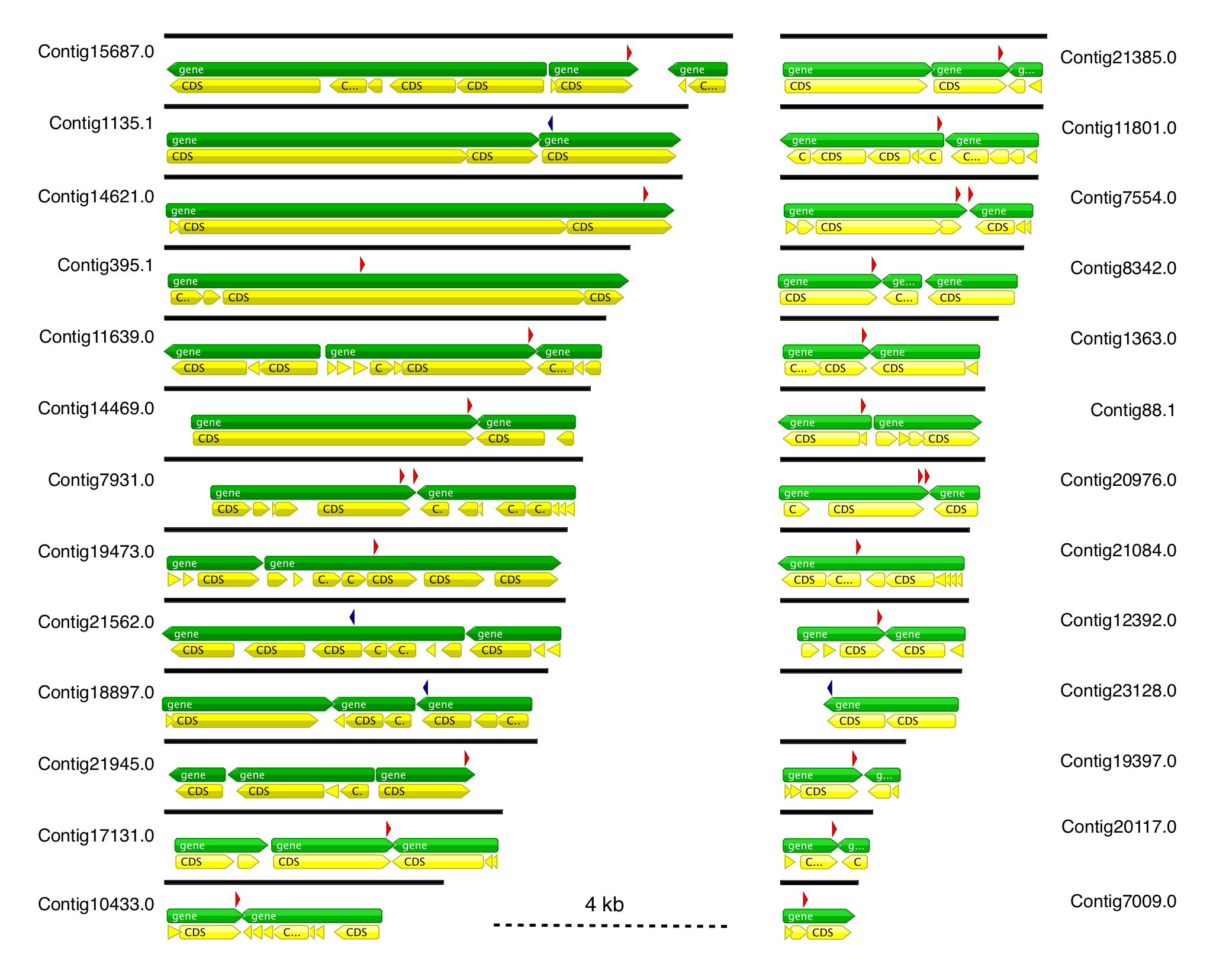

Supplement: Figure S15 — Intra-CDS alternative nanochromosome fragmentation. Nanochromosomes are indicated by black bars in descending order of length, with gene annotations below them. Predicted genes are indicated by green arrows and predicted CDSs by yellow arrows. Red arrows indicate alternative fragmentation sites and point in the direction that the alternative nanochromosome isoforms are produced. (TIFF) [file pbio.1001473.s015.tif]

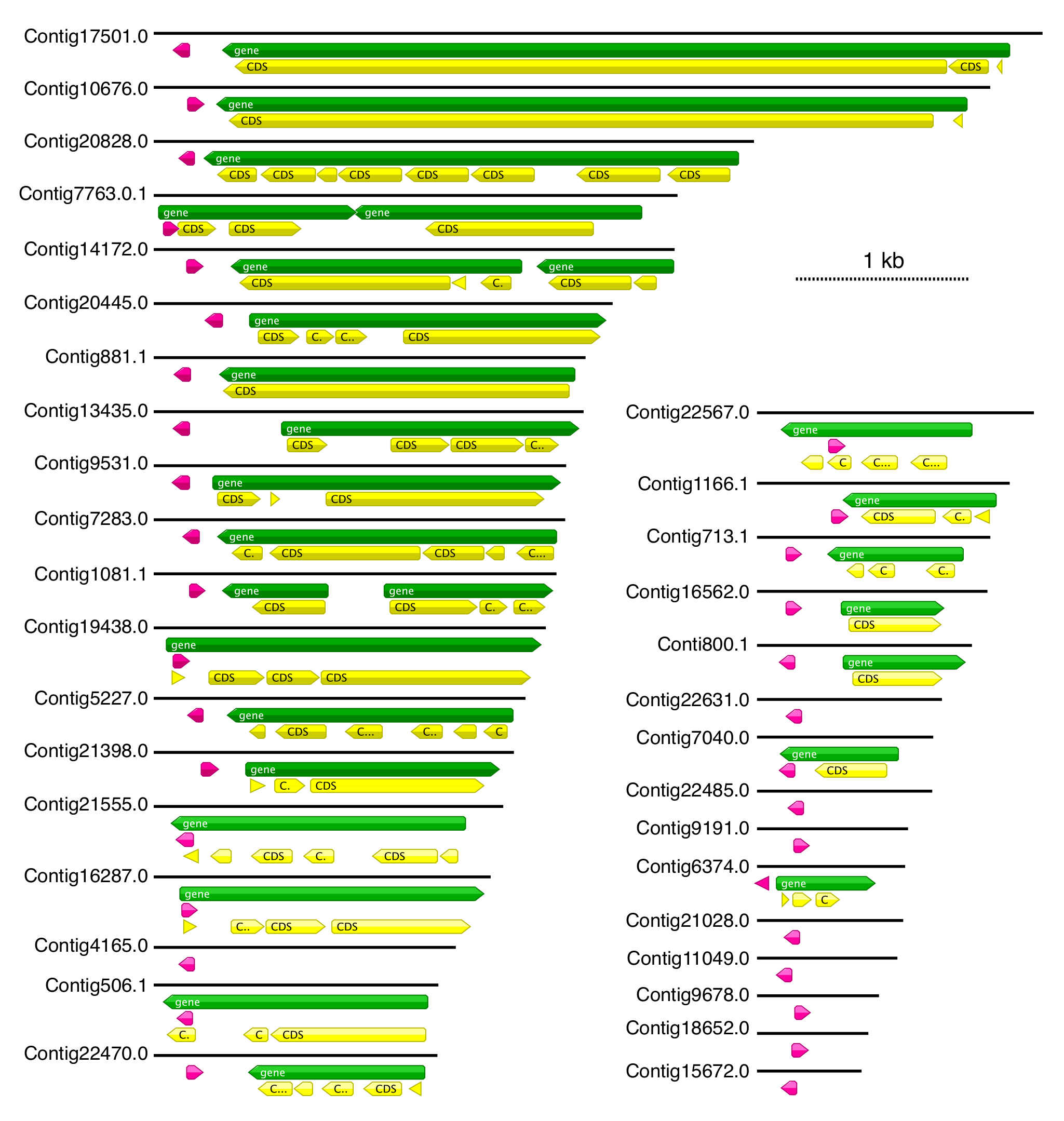

Supplement: Figure S16 — Nonalternatively fragmented tRNA nanochromosomes. Nanochromosomes are indicated by black bars in descending order of length, with gene annotations below them. Where multiple allelic versions of nanochromosomes are present, we have selected just a single representative nanochromosome. Predicted genes are indicated by green arrows, predicted CDSs by yellow arrows, and tRNAs by pink arrows. (TIFF) [file pbio.1001473.s016.tif]

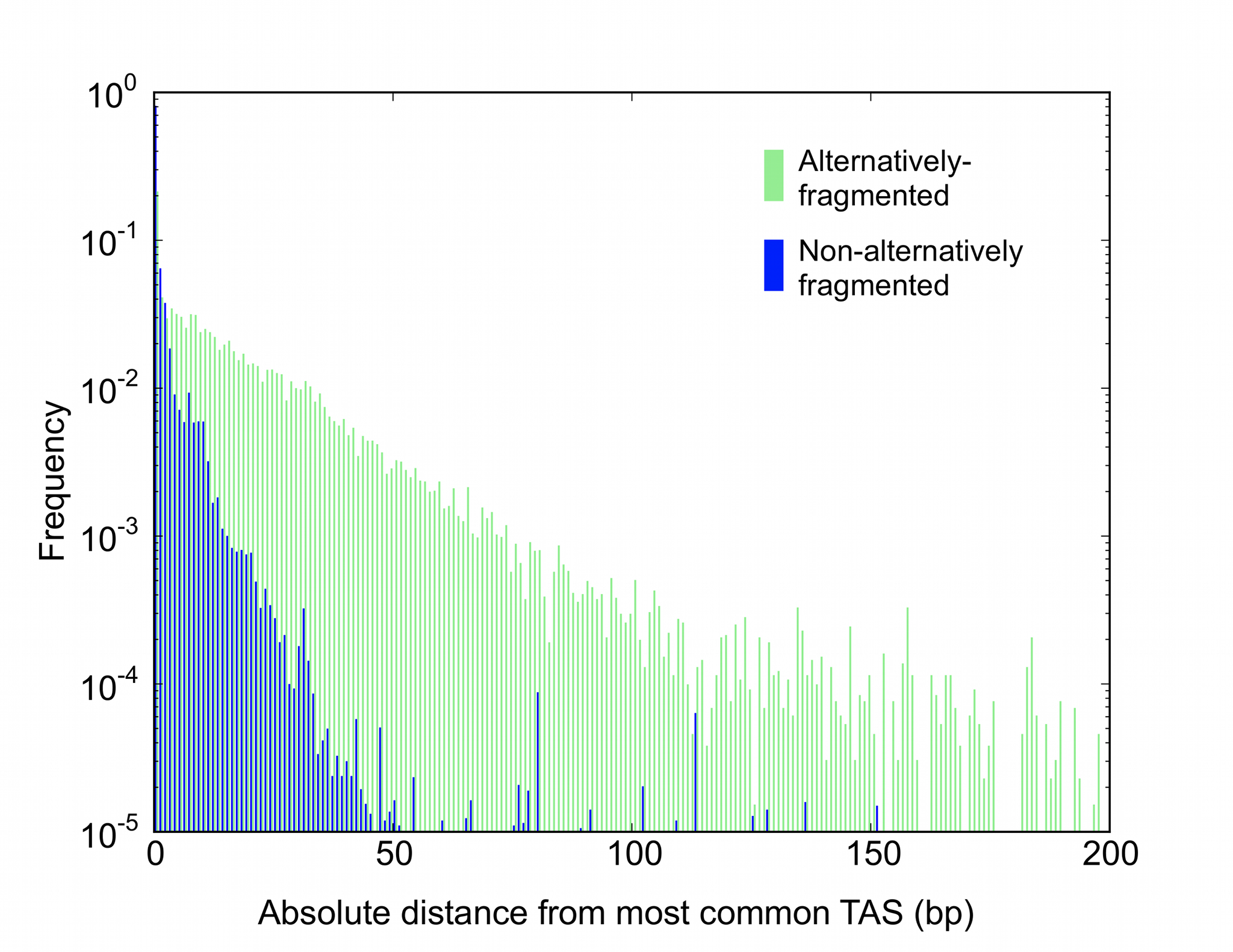

Supplement: Figure S17 — Positional variation of TASs. TASs are contig-derived (see Text S1: Determination of sequences surrounding telomere addition sites). TASs within a 200 bp window surrounding and centered on strongly supported, alternatively fragmented, and nonalternatively sites were counted. The frequency distributions of the TASs for alternatively fragmented sites are indicated in pale green and nonalternatively fragmented sites in blue. (TIFF) [file pbio.1001473.s017.tif]

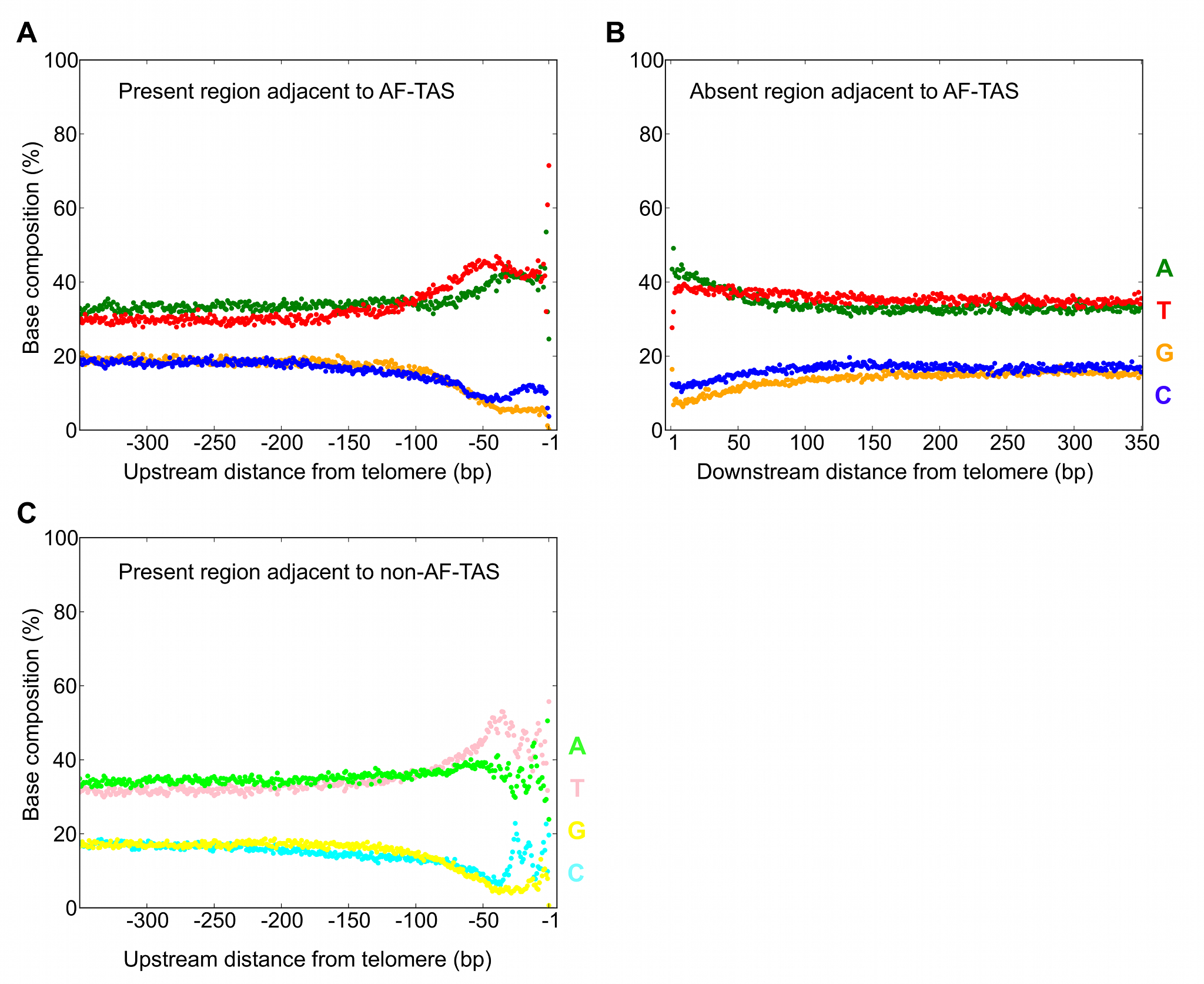

Supplement: Figure S18 — Base compositional biases surrounding TASs. Contig consensus sequences surrounding strongly supported site TASs (≥10 supporting Illumina telomeric reads) were extracted for (A–C) (see Text S1: Determination of sequences surrounding telomere addition sites). The telomere position is 0. We only illustrate base composition biases for one end of the nanochromosome since the complementary base frequencies are identical for both ends. (A) indicates the present region “upstream” of alternatively fragmented TASs (AF-TASs) that are present on the resulting nanochromosome. (B) indicates the absent region “downstream” of alternatively fragmented TASs (non-AF-TASs). (C) indicates the present region upstream of nonalternatively fragmented TASs. (TIFF) [file pbio.1001473.s018.tif]

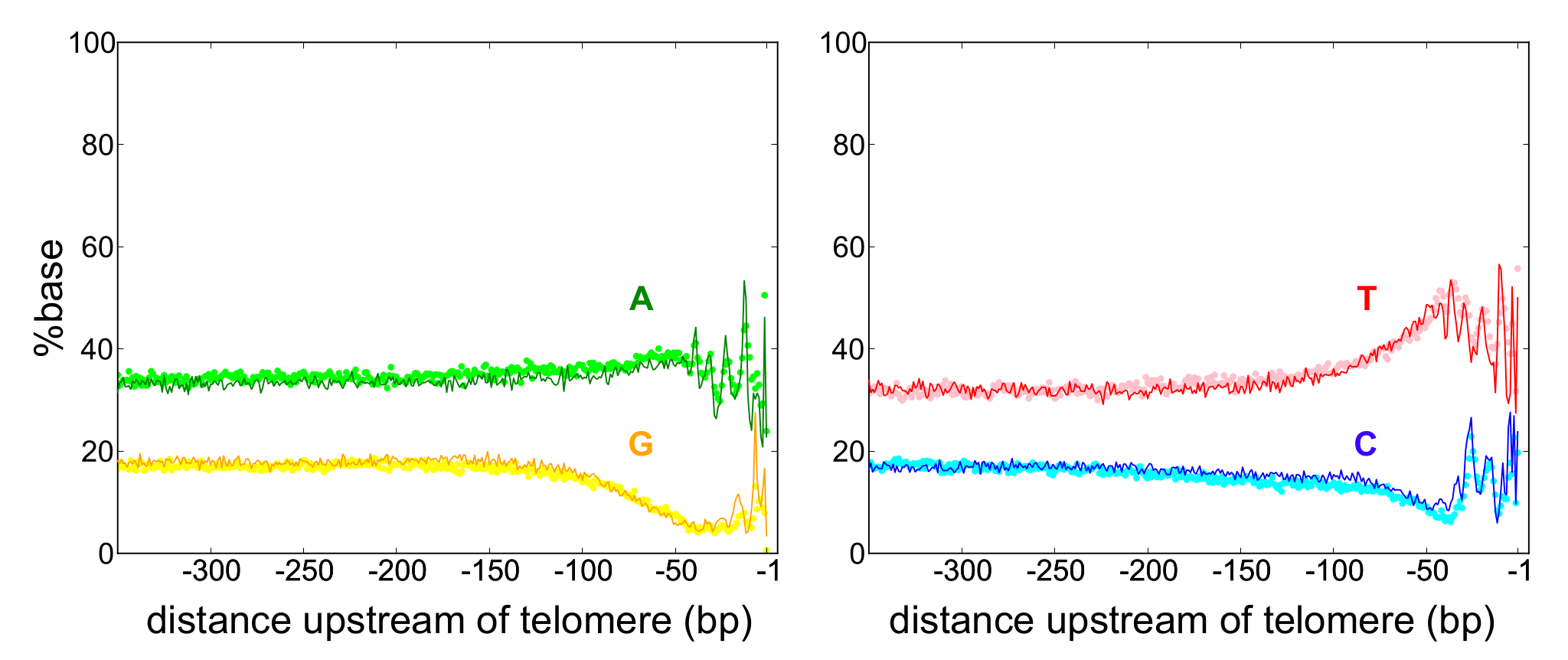

Supplement: Figure S19 — Nanochromosome subtelomeric base composition of Stylonychia compared to that of Oxytricha. Oxytricha base compositions are indicated by dots behind the Stylonychia base composition lines. (TIFF) [file pbio.1001473.s019.tif]

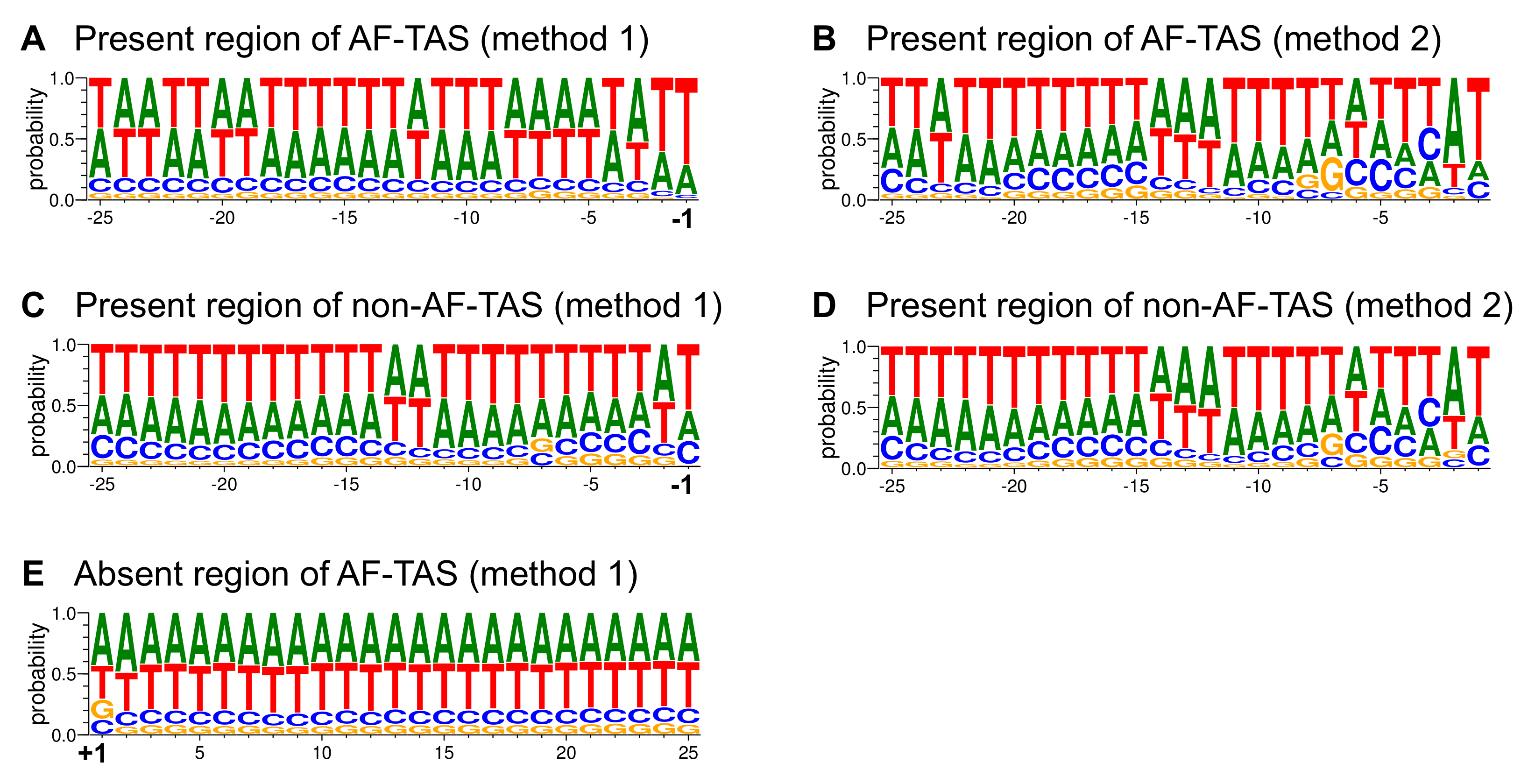

Supplement: Figure S20 — TAS sequence logos. Sequence logos showing nucleotide frequencies (generated with WebLogo [130]) for method 1 are for contig-derived sequences; while the logos for method 2 are for read-derived sequences. Sequence logos show base frequencies. (TIFF) [file pbio.1001473.s020.tif]

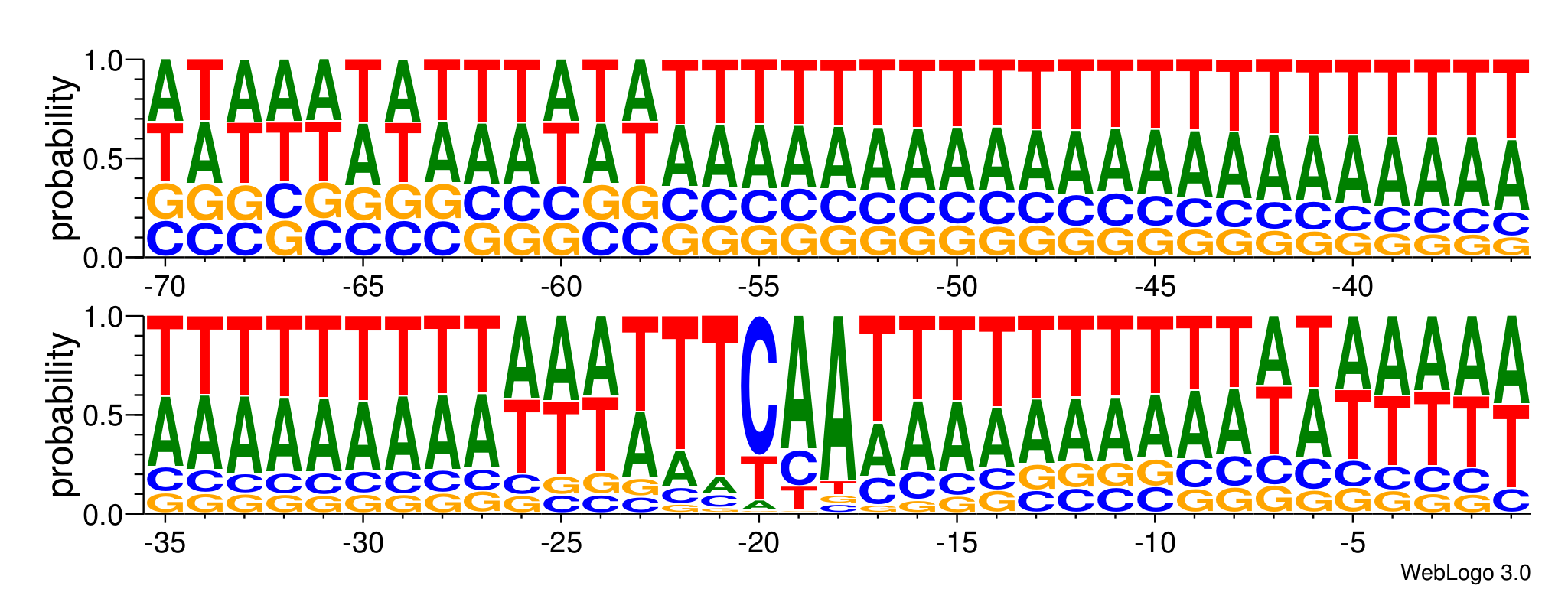

Supplement: Figure S21 — Sequence logo of Euplotes crassus subtelomeric regions. Sequence logos show base frequencies. Note that some of the motifs may be slightly misaligned (usually by 1 base), and hence the motif centered on position −20 would be even more prominent if they were correctly aligned. (TIFF) [file pbio.1001473.s021.tif]

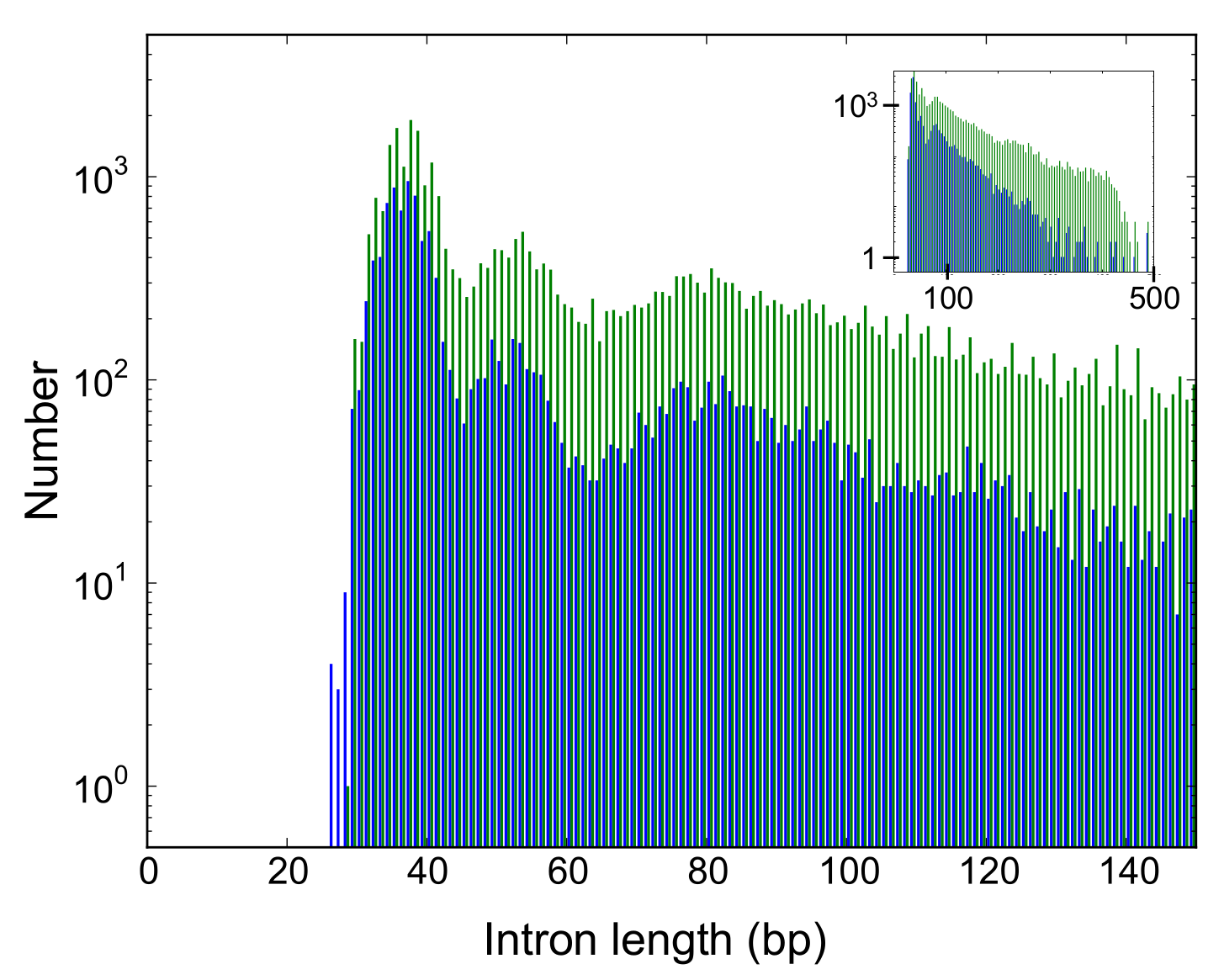

Supplement: Figure S22 — Intron length distribution. The green histogram is for all introns predicted by AUGUSTUS, including those with experimental support from RNA-seq data; the blue histogram is for all introns determined from RNA-seq data that were used as hints for AUGUSTUS during the gene prediction. The inset shows the size distribution over a longer length scale (with 5 bp bins). (TIFF) [file pbio.1001473.s022.tif]

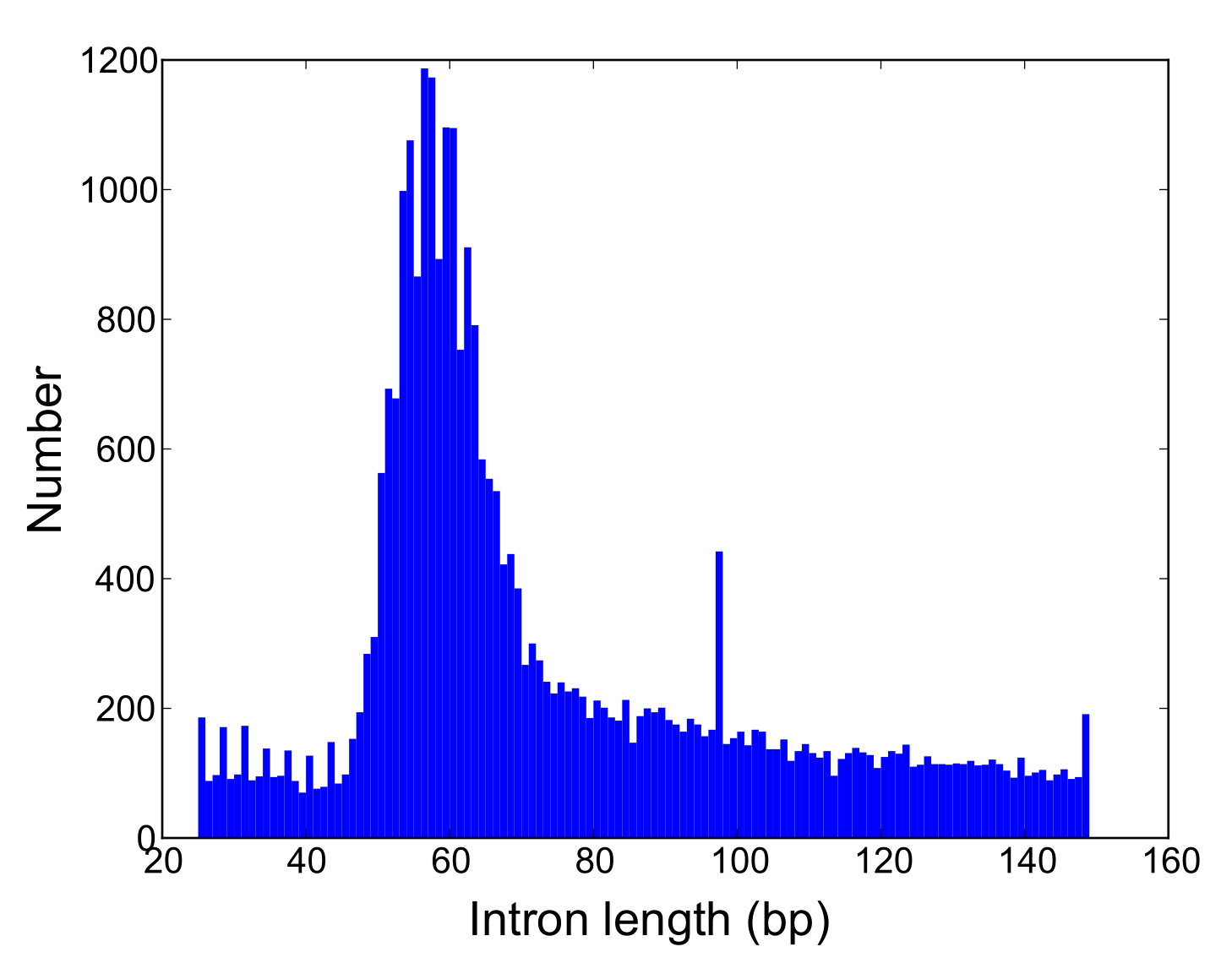

Supplement: Figure S23 — Intron length distribution for Tetrahymena thermophila gene predictions. Intron lengths determined from 2008 Tetrahymena gene predictions (downloaded from http://www.ciliate.org/system/downloads/oct2008_release.gff). (TIFF) [file pbio.1001473.s023.tif]

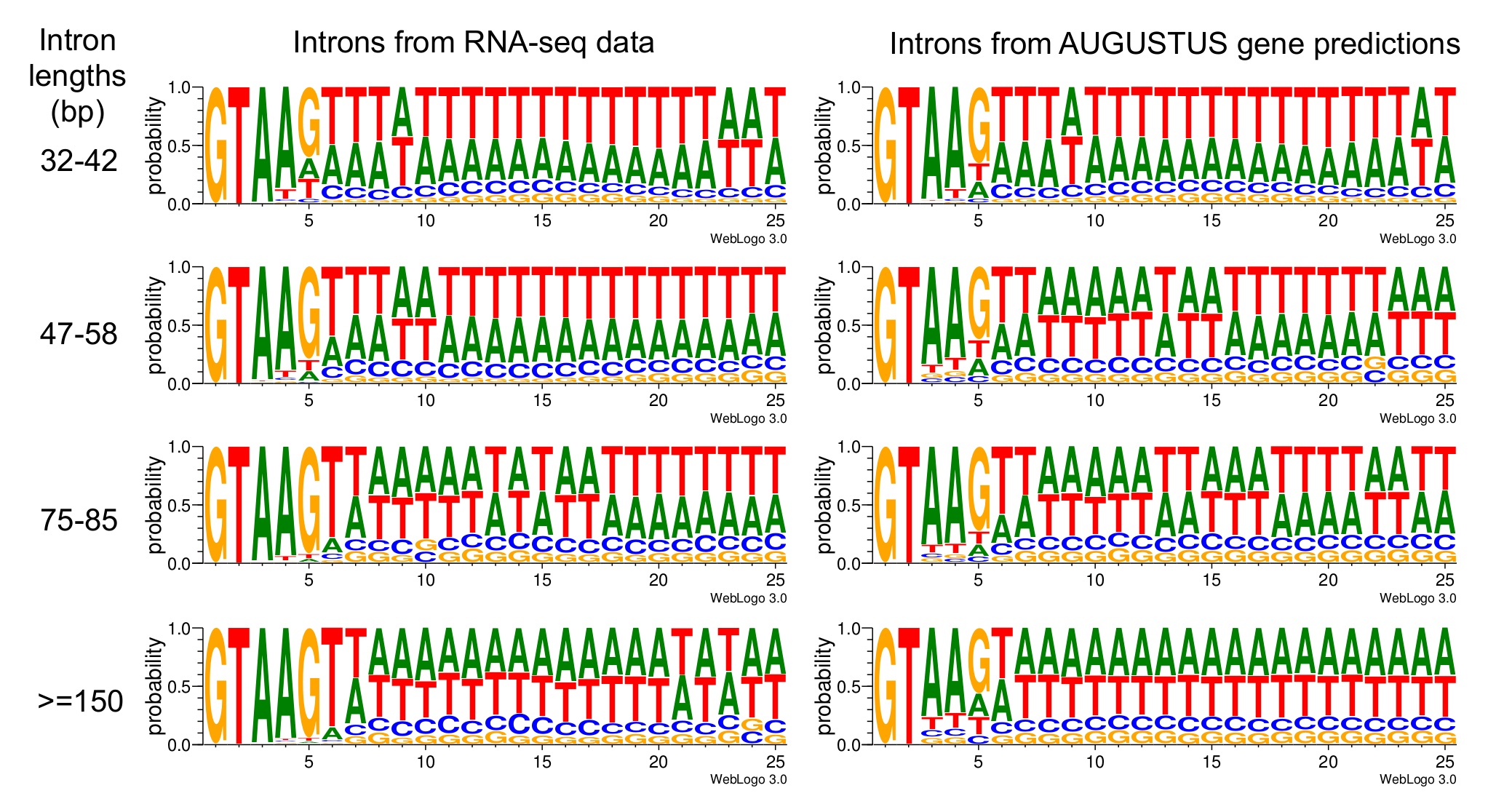

Supplement: Figure S24 — Sequence logos of experimentally determined and predicted intron donor sites. Sequence logos generated by WebLogo show base frequencies. Experimentally determined introns were obtained from RNA-seq data (see Text S1: Determination of sequences surrounding telomere addition sites). Predicted introns are all the introns predicted by AUGUSTUS, including those that have supporting RNA-seq evidence. Sequence logos were produced for introns in 11 bp intron length windows centered on the 37, 53, and 80 bp intron length modes, and for introns longer than 150 bp. Judging from the base composition of the third sequence position, which is almost exclusively “A” in introns extracted from the RNA-seq data, ∼10% of intron predictions may be incorrectly predicted for introns 53 bp and longer. Introns ≥53 bp constitute 39% of experimentally derived introns and 62% of predicted introns. (TIFF) [file pbio.1001473.s024.tif]

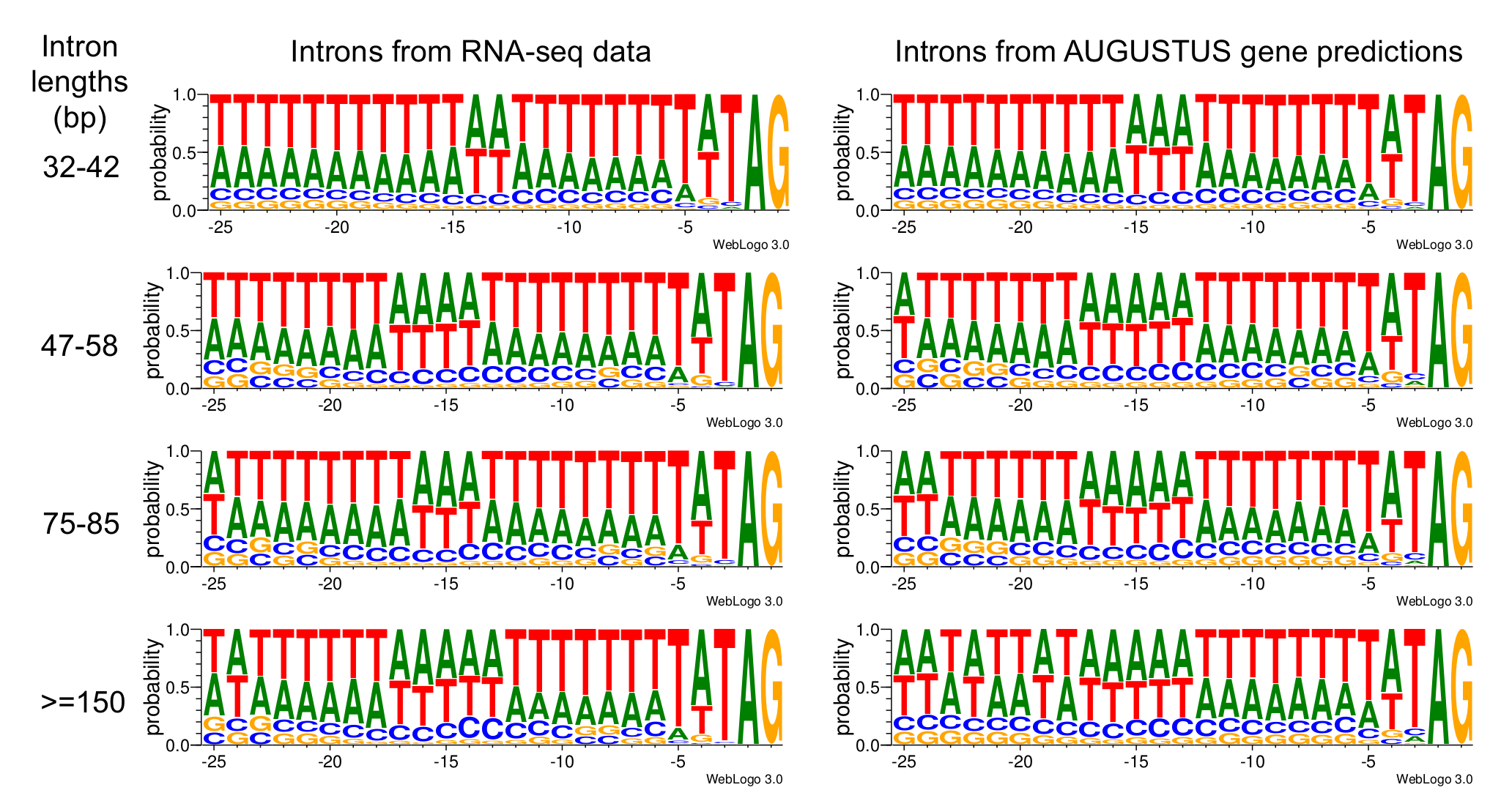

Supplement: Figure S25 — Sequence logos of experimentally determined and predicted intron acceptor sites. Sequence logos show base frequencies. Experimentally determined introns were obtained from RNA-seq data (see Text S1: Gene prediction). Predicted introns are all the introns predicted by AUGUSTUS, including those that have supporting RNA-seq evidence. Sequence logos were produced for introns in 11 bp intron length windows centered on the 37, 53, and 80 bp intron length modes, and for introns longer than 150 bp. (TIFF) [file pbio.1001473.s025.tif]

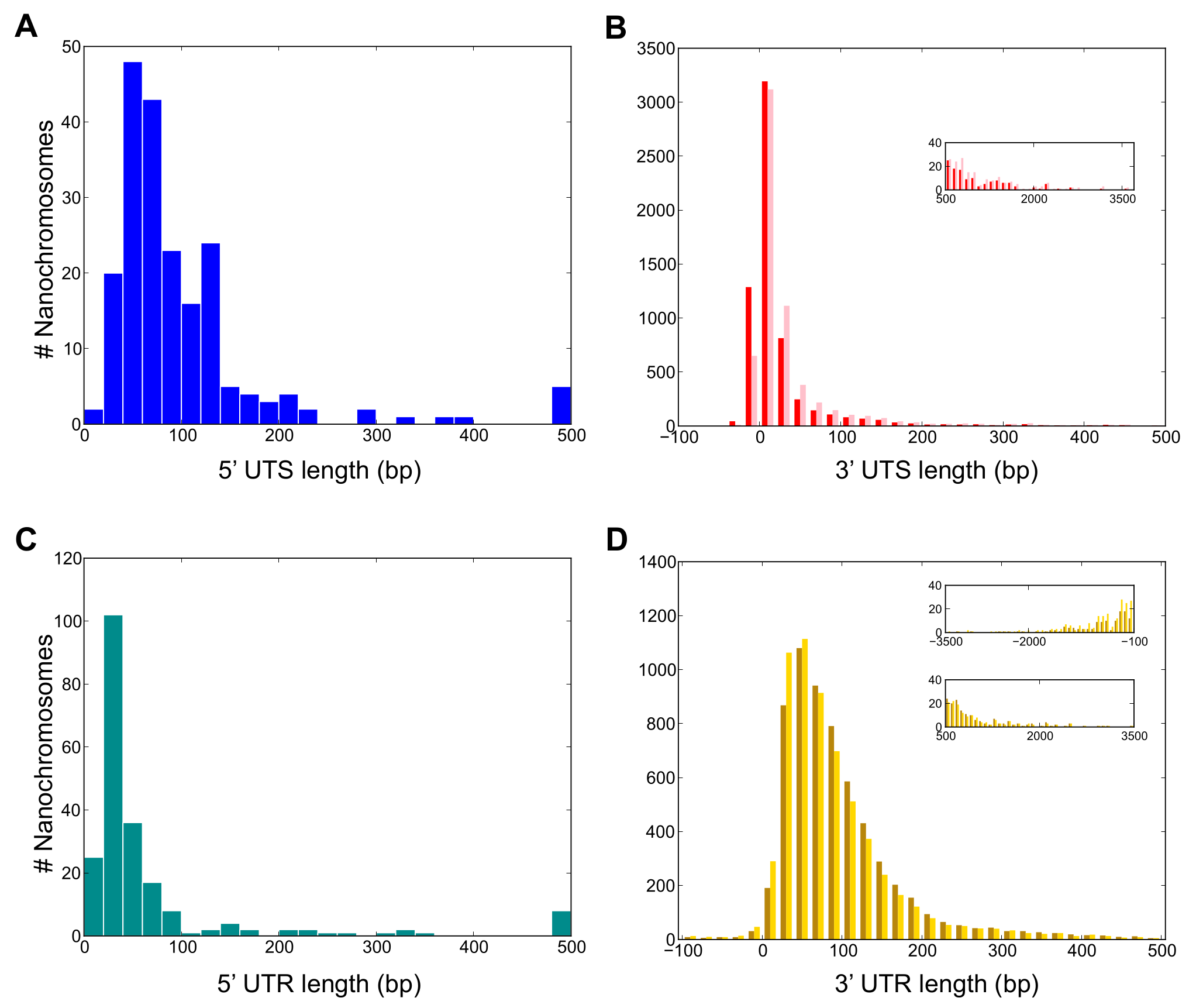

Supplement: Figure S26 — Length distributions of untranscribed (UTS) and untranslated (UTR) regions. Length distributions are for single-gene, nonalternatively fragmented nanochromosomes. (A) 5′ UTS length from the transcription start site to telomere (determined from 5′-RLM RACE Sanger reads). (B) 3′ UTS length from polyadenylation site to telomere (determined from RNA-seq reads); the two graphs are for the site closest to the telomere (red) and the most frequently used polyadenylation site (pink); negative lengths indicate polyadenylation sites extending beyond the telomere. The 3′ UTS median length is 25 bp for the most frequently used polyadenylation sites and 19 bp for polyadenylation sites closest to telomeres. (C) 5′ UTR lengths from 5′-RLM RACE Sanger reads. (D) 3′ UTR lengths from RNA-seq reads; negative lengths indicate polyadenylation sites upstream of the stop codon; the two graphs are for the site closest to the telomere (dark gold) and the most frequently used polyadenylation site (gold). 3′ UTRs have a median length of 78 bp when the most frequently used polyadenylation sites are counted or 87 bp for polyadenylation sites closest to telomeres. (TIFF) [file pbio.1001473.s026.tif]

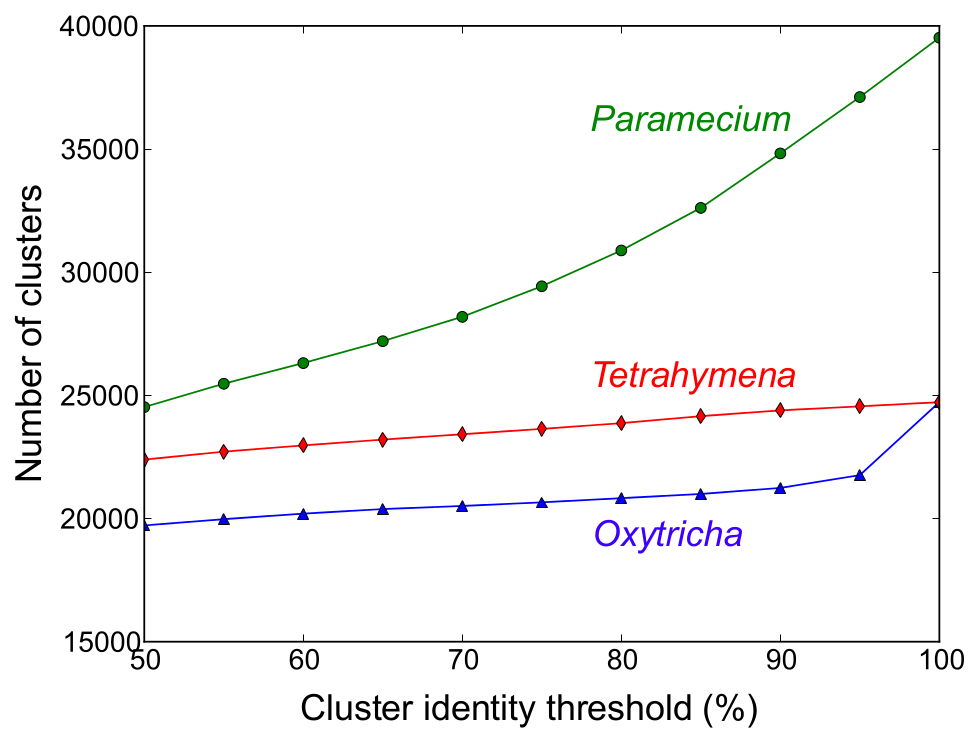

Supplement: Figure S27 — Assessment of potential paralogy in model ciliate genomes. UCLUST from the USEARCH suite (version 5.1.221) [131] was used for clustering at increasing global sequence alignment identity clustering thresholds, with the query and target alignment fractions both set to 80% coverage (i.e., number of letters in the query that are aligned to letters in the target), and the parameters –maxaccepts 3 –maxrejects 128 to increase clustering sensitivity at medium sequence identity levels. The increase in number of clusters from the 95% to 100% cluster identity threshold for Oxytricha reflects clustering of protein alleles. (TIFF) [file pbio.1001473.s027.tif]

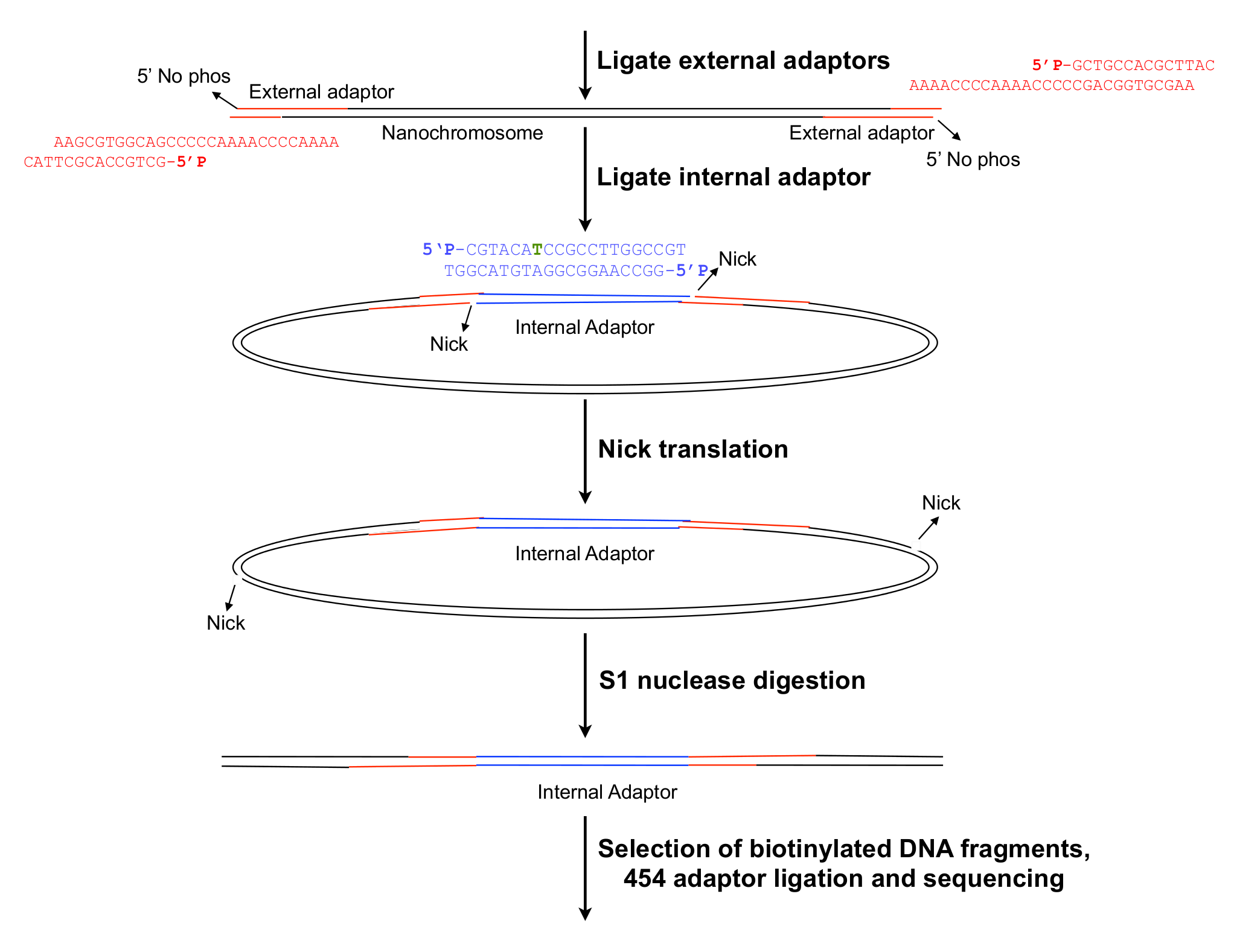

Supplement: Figure S28 — Subtelomeric DNA capture method for 454 subtelomeric sequencing. Adaptor ends with a 5′-phosphate are shown in bold; otherwise 5′-phosphate is absent. The biotinylated thyamine residue in the internal adaptor is indicated in green. (TIFF) [file pbio.1001473.s028.tif]

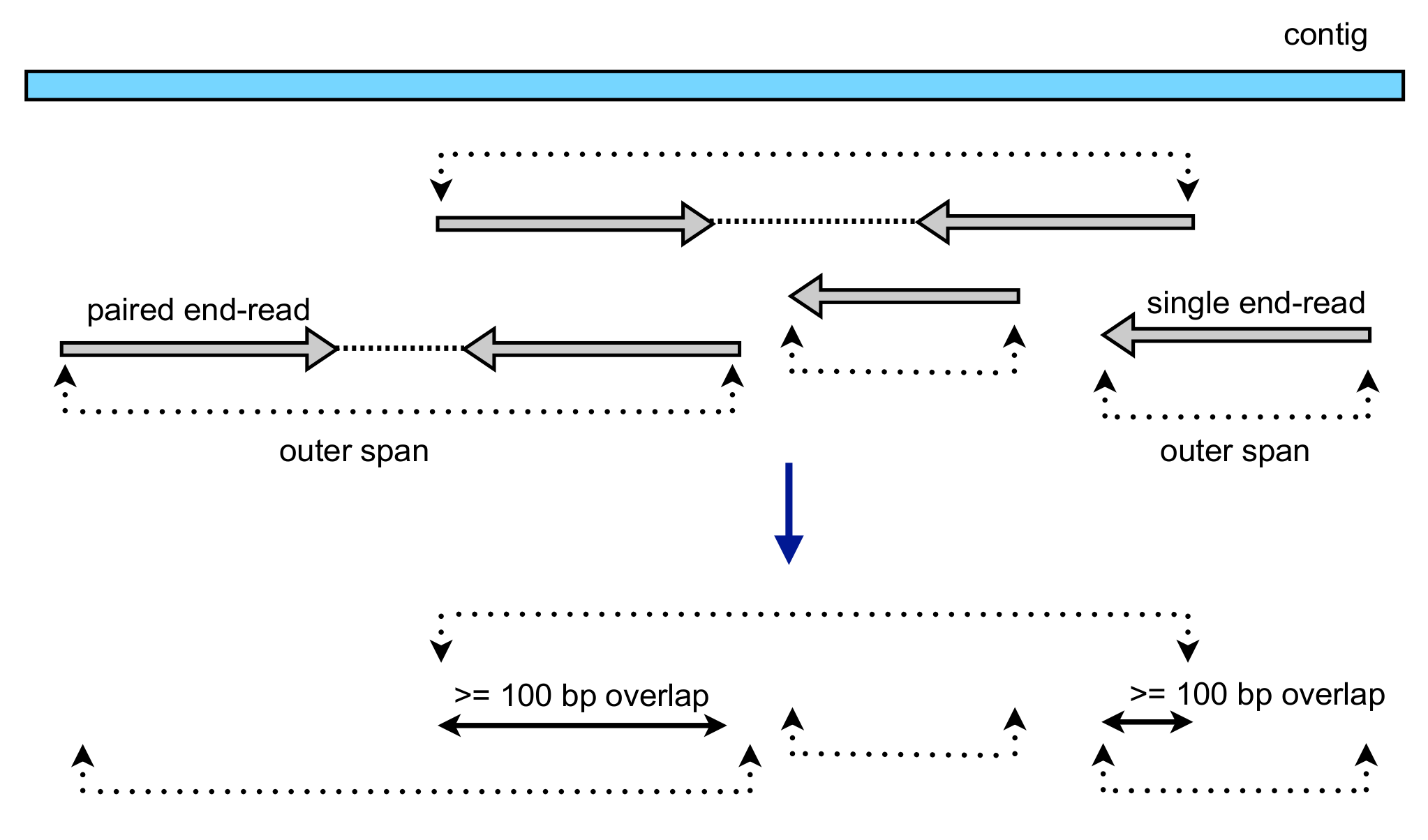

Supplement: Figure S29 — End-to-end validation by Sanger mate pair reads. Paired and SE reads are shown with gray arrows. First, “outer spans” between the ends of paired-end reads or consisting of the entire SE read are found. Next, we attempt to greedily find a path through the spans, so that there are ≥100 bp overlaps between the spans comprising the path. If we find such a path, the contig is considered to be validated. (TIFF) [file pbio.1001473.s029.tif]

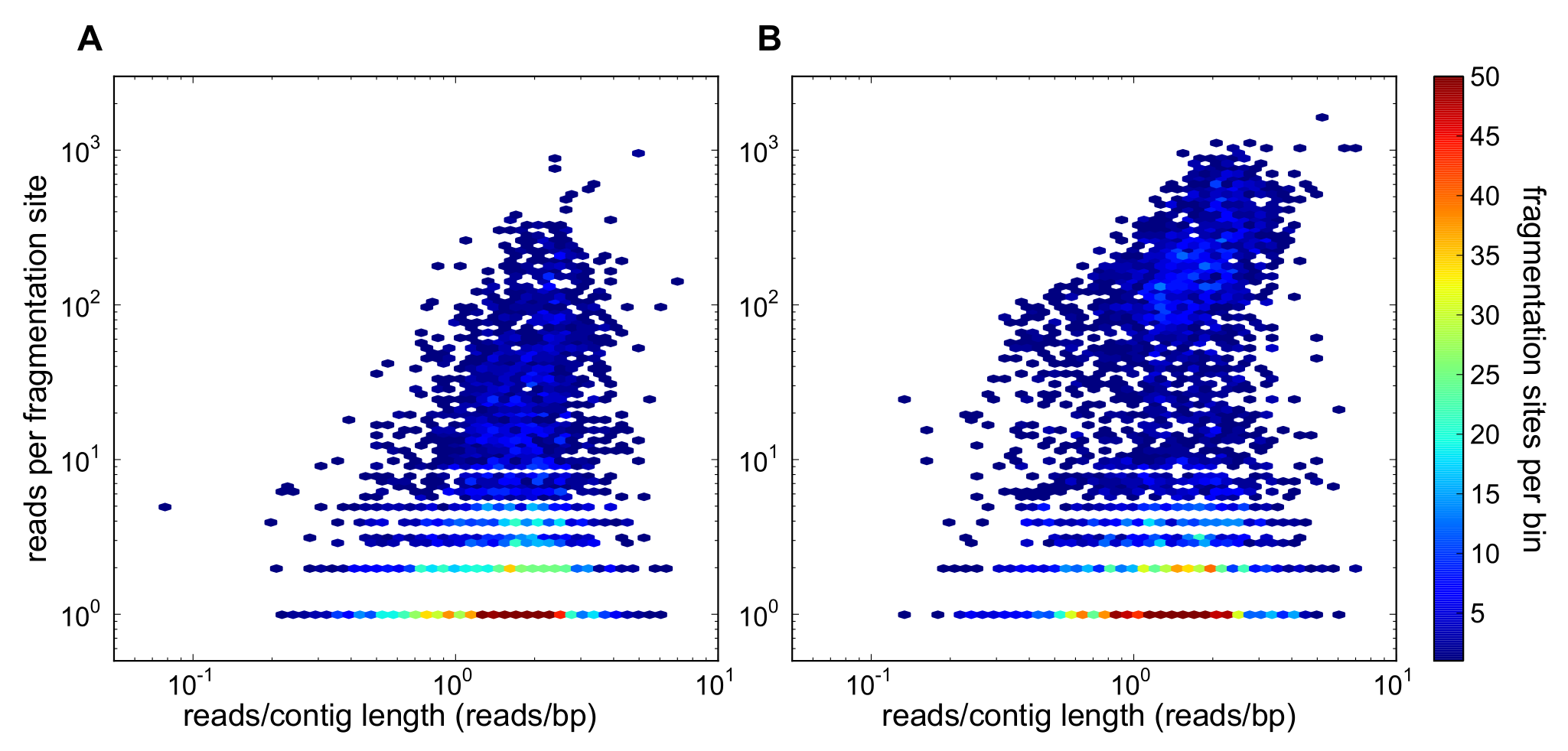

Supplement: Figure S30 — Distribution of supporting telomeric reads per alternative fragmentation site versus reads per contig length. Alternative fragmentation sites >400 bp from contig ends are hexagonally binned. The x-axis units are an estimate of relative nanochromosome copy number based on the total number of mapped Illumina reads, both telomeric and nontelomeric, per bp for each contig. On the y-axis, reads per fragmentation site were calculated for a 100 bp window centered on the site with the most reads supporting the alternative fragmentation site. (A) 454-telomeric end reads (2,792 contigs; 3,634 sites); (B) Illumina telomeric reads (3,331 contigs; 4,392 sites). (TIFF) [file pbio.1001473.s030.tif]
