# Supplementary material for: The Oxytricha trifallax Macronuclear Genome: A Complex Eukaryotic Genome with 16,000 Tiny Chromosomes
Source: PLoS Biol. 2013 Jan 29;11(1):e1001473. doi: 10.1371/journal.pbio.1001473 (PMC3558436; doi:10.1371/journal.pbio.1001473)
Supplement: Table S1 — Location of alternative fragmentation sites relative to inter- and intracoding sequence regions for two-gene nanochromosomes. Alternative fragmentation sites with decreasing numbers of supporting telomeric reads are shown in three successive columns. To exclude conventional TASs, only alternative fragmentation sites at least 100 bp away from either end of the contig were selected. Nanochromosomes with single alternative fragmentation sites were selected. AUGUSTUS gene predictions were used to determine inter-/intra-CDS regions. Similar trends were found for 454 telomeric reads. %GC was determined for a 50 bp window either side of alternative fragmentation sites. (RTF) [file pbio.1001473.s031.rtf]

Table S1. Location of alternative fragmentation sites relative to inter- and intra-coding sequence regions for two-gene nanochromosomes.

	Telomeric read support	
	≥ 10 telomeric reads	< 10 telomeric reads	1 telomeric read	
# alternatively fragmented contigs	1120	640	259	
Inter-CDS fragmentation sites	931	408	132	
Intra-CDS fragmentation sites	189	232	127	
Inter-CDS fragmentation sites/ Inter-CDS length (bp)	2.19E-03	1.30E-03	1.02E-03	
Intra-CDS fragmentation sites/ intra-CDS length (bp)	3.80E-05	7.54E-05	1.02E-04	
Inter-CDS length (bp)	424,645	314,116	129,129	
Intra-CDS length (bp)	4,973,157	3,077,138	1,239,388	
%GC of 100 bp window around inter-CDS fragmentation sites 	18.2	20.2	20.7	
%GC of 100 bp window around intra-CDS fragmentation sites	22.3	29.0	30.2	
