# Supplementary material for: The Oxytricha trifallax Macronuclear Genome: A Complex Eukaryotic Genome with 16,000 Tiny Chromosomes
Source: PLoS Biol. 2013 Jan 29;11(1):e1001473. doi: 10.1371/journal.pbio.1001473 (PMC3558436; doi:10.1371/journal.pbio.1001473)
Supplement: Table S2 — Location of alternative fragmentation sites relative to coding and noncoding sequence regions for single-gene nanochromosomes. Alternative fragmentation sites with decreasing numbers of supporting telomeric reads are shown in three successive columns. To exclude conventional TASs, only alternative fragmentation sites at least 100 bp away from either end of the contig were selected. Nanochromosomes with single alternative fragmentation sites were selected. CDS/non-CDS regions were determined from the AUGUSTUS gene predictions. Similar trends were observed for 454 telomeric reads (not shown). %GC was determined for a 50 bp window either side of alternative fragmentation sites. (RTF) [file pbio.1001473.s032.rtf]

Table S2. Location of alternative fragmentation sites relative to coding and noncoding sequence regions for single-gene nanochromosomes.

	Telomeric read support	
	≥ 10 telomeric reads	< 10 telomeric reads	1 telomeric read	
# alternatively fragmented contigs	515	545	270	
CDS fragmentation sites	48	264	152	
non-CDS fragmentation sites	467	281	118	
CDS fragmentation sites/ CDS length (bp)	3.71E-05	1.69E-04	1.88E-04	
Non-CDS fragmentation sites/ non-CDS length (bp)	1.02E-03	6.25E-04	5.73E-04	
CDS length (bp)	1,294,726	1,564,930	806,590	
Non-CDS length (bp)	458,091	449,866	205,777	
%GC of 100 bp window around CDS fragmentation sites %GC	27.4	32.4	33.0	
%GC of 100 bp window around non-CDS fragmentation sites	21.5	23.3	24.6	
