# Supplementary material for: The Oxytricha trifallax Macronuclear Genome: A Complex Eukaryotic Genome with 16,000 Tiny Chromosomes
Source: PLoS Biol. 2013 Jan 29;11(1):e1001473. doi: 10.1371/journal.pbio.1001473 (PMC3558436; doi:10.1371/journal.pbio.1001473)
Supplement: Table S3 — Estimates of nanochromosome copy number. Only the rRNA nanochromosome in this table is alternatively fragmented (with a site at 634 bp supported by 11 reads and two sites at 1,253 bp and 6,077 bp supported by a single read). 5′- and 3′-telomeric reads refer to reads that are mapped either to the 5′ or 3′ end as it is oriented in the genome assembly. (RTF) [file pbio.1001473.s033.rtf]

Table S3. Estimates of nanochromosome copy number.

Contig	Gene	Reads/bp	5' telomeric reads/
contig	3' telomeric reads/contig	All telomeric reads/
contig	qPCR estimate	
Contig451.1	rRNA	34.24	10353	10028	20,381	5.16E+07	
Contig22209.0	TEBP-α	3.02	71	660	731	2.61E+06	
Contig22794.0	DNA Pol-α	1.58	322	208	530	1.88E+06	
Contig5780.0	RNA Pol II	0.29	2	70	72	2.69E+05	
Contig15858.0	Xrcc-3	0.57	135	87	222	2.57E+05	
