# Supplementary material for: The Oxytricha trifallax Macronuclear Genome: A Complex Eukaryotic Genome with 16,000 Tiny Chromosomes
Source: PLoS Biol. 2013 Jan 29;11(1):e1001473. doi: 10.1371/journal.pbio.1001473 (PMC3558436; doi:10.1371/journal.pbio.1001473)
Supplement: Table S4 — Large predicted proteins. The 20 longest nanochromosomes, excluding cases that appear to be redundant (i.e., quasi-alleles), are shown. None of these nanochromosomes is alternatively fragmented. Nanochromosome lengths include telomeres. Protein domain names are abbreviations from Pfam-A (version 26). Semicolons separate predicted protein lengths and protein domain architectures. (RTF) [file pbio.1001473.s034.rtf]

Table S4. Large predicted proteins.

Contig	Large protein name (Pfam domain list)	Protein length (aa)	Nanochromo-some length (bp)	
Contig7580.0	171 x fn3; 9 x Filamin, 7 x PA14	21,680	66,022	
Contig1417.1	Unknown, AAA_6	651; 8,308	29,046	
Contig19357.0	4 x TIG; 11 x TIG	2,862; 3,844	27,060	
Contig20846.0	ZZ, UQ_con, 2 x ThiF, UBA_e1_thiolCys, UBACT	7,181	22,965	
Contig6505.0	7 x TIG; 3 x GCC2_GCC3	7,336	22,354	
Contig289.1	PAS_9; PAS_9; Pkinase; Unknown	1,667; 1,653; 506; 1,705	19,975	
Contig19385.0	EF_hand5; EF_hand_6, EF_hand_5, EF_hand_6	615; 5,623	19,378	
Contig11558.0	Unknown	6,015	18,662	
Contig354.1	AAA_6, AAA_7, MT, AAA_9, Dynein_heavy	6,053	18,574	
Contig9088.0	Unknown; Unknown	678; 4,638	18,501	
Contig983.0	Unknown	5,763	18,371	
Contig4708.0	UQ_con, ThiF x 2, UBA_e1_thiolCys, UBACT x 2, UBA_e1_C	5,674	17,667	
Contig837.1	Unknown	5,347	17,577	
Contig473.1	~20 x GCC2_GCC3 domain; Unknown	4,977; 455	17,346	
Contig3496.0	HECT domain	5,579	17,246	
Contig743.1	Unknown; Unknown	2,552; 1,393	17,061	
Contig20303.0	E3_UbLigase_R4	5,512	16,978	
Contig2629.0	Unknown	5,149	16,971	
Contig948.1	Laminin_EGF	4,900	15,909	
Contig13955.0	AAA_5, DENN	5,200	15,895	
