# Supplementary material for: The Oxytricha trifallax Macronuclear Genome: A Complex Eukaryotic Genome with 16,000 Tiny Chromosomes
Source: PLoS Biol. 2013 Jan 29;11(1):e1001473. doi: 10.1371/journal.pbio.1001473 (PMC3558436; doi:10.1371/journal.pbio.1001473)
Supplement: Table S5 — Oxytricha nucleic-acid-associated protein domains not found in Paramecium and Tetrahymena. aJudging from multiple sequence alignments, domain appears to exist in Paramecium (GSPATP00020413001) and Tetrahymena (TTHERM_00721450) but was not detected by hmmscan (HMMER3) bindependent E-value greater than the threshold (0.001), but domain exists (e.g., protein TTHERM_01211800 in Tetrahymena). cProtein encoded on contigs, with no telomeric repeats, that are likely bacterial contaminants dPredicted protein for an incompletely assembled part of a previously characterized mitochondrial plasmid encoding a viral/organellar type DNA polymerase [69]. eIndependent E-value greater than the threshold (0.001) used, but domain exists in Paramecium [124] and Tetrahymena. fIndependent E-value greater than the threshold (0.001) used, but sequence alignments of proteins containing N-terminal domain (TFIIA_gamma_N) to homologs from Oxytricha suggest that hmmscan failed to detect this domain in Tetrahymena and Paramecium. (RTF) [file pbio.1001473.s035.rtf]

Table S5. Oxytricha nucleic-acid associated protein domains not found in Paramecium and Tetrahymena.

Domain i-Eval	Protein ID	Pfam ID	Domain name	Domain description	
4.30E-242	Contig22260.0.g8	PF07404	TEBP_beta	Telomere-binding protein beta subunit (TEBP beta)	
1.30E-51	Contig11834.0.g57	PF07404	TEBP_beta	Telomere-binding protein beta subunit (TEBP beta)	
1.40E-25	Contig1486.1.g68	PF07404	TEBP_beta	Telomere-binding protein beta subunit (TEBP beta)	
8.70E-34	Contig4712.0.g109	PF04801	Sin_N	Sin-like protein conserved region	
6.50E-20	Contig18453.0.g8	PF08625	Utp13	Utp13 specific WD40 associated domain	
3.50E-19	Contig6051.0.g68a	PF04152	Mre11_DNA_bind	Mre11 DNA-binding presumed domain	
6.50E-15	Contig17739.0.g106a	PF04152	Mre11_DNA_bind	Mre11 DNA-binding presumed domain	
6.60E-19	Contig237.1.g127b	PF03847	TFIID_20kDa	Transcription initiation factor TFIID subunit A	
1.80E-18	Contig6077.0.g65c	PF01609	DDE_Tnp_1	Transposase DDE domain	
7.50E-05	Contig6077.0.g65c	PF01609	DDE_Tnp_1	Transposase DDE domain	
2.50E-16	Contig8748.0.g3	PF04719	TAFII28	hTAFII28-like protein conserved region	
1.20E-15	Contig11849.0.g52	PF06331	Tbf5	Transcription factor TFIIH complex subunit Tfb5	
3.30E-15	Contig15803.0.g96	PF02270	TFIIF_beta	Transcription initiation factor IIF, beta subunit	
5.60E-06	Contig517.1.g109	PF02270	TFIIF_beta	Transcription initiation factor IIF, beta subunit	
540.0	Contig517.1.g109	PF02270	TFIIF_beta	Transcription initiation factor IIF, beta subunit	
2.40E-05	Contig14938.0.g40	PF02270	TFIIF_beta	Transcription initiation factor IIF, beta subunit	
3.30E-14	Contig16730.0.g8	PF02269	TFIID-18kDa	Transcription initiation factor IID, 18kD subunit	
1.90E-13	Contig1439.1.g129	PF00589	Phage_integrase	Phage integrase family	
1.90E-13	Contig228.0.0.g36d	PF03175	DNA_pol_B_2	DNA polymerase type B, organellar and viral	
8.80E-11	Contig12197.0.g66	PF09507	CDC27	DNA polymerase subunit Cdc27	
3.70E-10	Contig16809.0.g65	PF09507	CDC27	DNA polymerase subunit Cdc27	
1.20E-10	Contig15376.0.g47	PF09088	MIF4G_like	MIF4G like	
1.10E-09	Contig798.1.g90	PF05236	TAF4	Transcription initiation factor TFIID component TAF4 family	
0.29	Contig798.1.g90	PF05236	TAF4	Transcription initiation factor TFIID component TAF4 family	
5.30E-09	Contig12696.0.g64	PF00707	IF3_C	Translation initiation factor IF-3, C-terminal domain	
8.30E-09	Contig6770.0.g23	PF07034	ORC3_N	Origin recognition complex (ORC) subunit 3 N-terminus	
1.80E-07	Contig7520.0.g66	PF08147	DBP10CT	DBP10CT (NUC160) domain	
2.40E-07	Contig469.0.g27	PF05486	SRP9-21	Signal recognition particle 9 kDa protein (SRP9)	
1.10E-06	Contig10901.0.1.g33e	PF06632	XRCC4	DNA double-strand break repair and V(D)J recombination protein XRCC4	
1.10E-06	Contig14147.0.g88f	PF02751	TFIIA_gamma_C	Transcription initiation factor IIA, gamma subunit	
3.10E-05	Contig8438.0.g48	PF03444	HrcA_DNA-bdg	Winged helix-turn-helix transcription repressor, HrcA DNA-binding	
0.00011	Contig9814.0.g17	PF04546	Sigma70_ner	Sigma-70, non-essential region	
250.0	Contig9814.0.g17	PF04546	Sigma70_ner	Sigma-70, non-essential region	
0.00042	Contig11991.0.g79	PF00216	Bac_DNA_binding	Bacterial DNA-binding protein	
110.0	Contig11991.0.g79	PF00216	Bac_DNA_binding	Bacterial DNA-binding protein	
