# Supplementary material for: The Oxytricha trifallax Macronuclear Genome: A Complex Eukaryotic Genome with 16,000 Tiny Chromosomes
Source: PLoS Biol. 2013 Jan 29;11(1):e1001473. doi: 10.1371/journal.pbio.1001473 (PMC3558436; doi:10.1371/journal.pbio.1001473)
Supplement: Table S6 — Oxytricha putative nucleic-acid-associated protein domains (not annotated in pfam2go) not found in Paramecium and Tetrahymena. Domains in this table are considered to have putative nucleic-acid-related functions based on Pfam descriptions and literature cited for these domains. aProteins encoded on contigs with no telomeric repeats. bProtein is truncated due to an incorrect intron prediction; this protein is an allelic variant of the protein Contig14154.0.g51, which is the correctly predicted allelic variant. cProtein encoded on contigs with no telomeric repeats that are likely bacterial contaminants. (RTF) [file pbio.1001473.s036.rtf]

Table S6. Oxytricha putative nucleic-acid associated protein domains (not annotated in pfam2go) not found in Paramecium and Tetrahymena.

Domain i-Eval	Protein ID	Pfam ID	Domain name	Domain description	
1.50E-36	Contig7118.0.g3	PF12762	DDE_Tnp_IS1595	ISXO2-like transposase domain	
2.20E-26	Contig20019.0.g82	PF12762	DDE_Tnp_IS1595	ISXO2-like transposase domain	
3.20E-24	Contig2438.0.g81	PF12762	DDE_Tnp_IS1595	ISXO2-like transposase domain	
5.70E-24	Contig18469.0.g6	PF12762	DDE_Tnp_IS1595	ISXO2-like transposase domain	
4.80E-14	Contig2419.0.g102	PF12762	DDE_Tnp_IS1595	ISXO2-like transposase domain	
1.60E-13	Contig400.0.g115	PF12762	DDE_Tnp_IS1595	ISXO2-like transposase domain	
1.70E-09	Contig18522.0.g12	PF12762	DDE_Tnp_IS1595	ISXO2-like transposase domain	
9.20E-09	Contig390.0.g93	PF12762	DDE_Tnp_IS1595	ISXO2-like transposase domain	
2.00E-08	Contig12079.0.g56	PF12762	DDE_Tnp_IS1595	ISXO2-like transposase domain	
6.20E-06	Contig17585.0.g103	PF12762	DDE_Tnp_IS1595	ISXO2-like transposase domain	
1.50E-05	Contig19130.0.g71	PF12762	DDE_Tnp_IS1595	ISXO2-like transposase domain	
2.10E-25	Contig22794.0.g89	PF12254	DNA_pol_alpha_N	DNA polymerase alpha subunit p180 N terminal	
2.30E-24	Contig566.1.g37	PF10551	MULE	MULE transposase domain	
8.30E-23	Contig19058.0.g68	PF10551	MULE	MULE transposase domain	
8.60E-12	Contig9900.0.g91	PF10551	MULE	MULE transposase domain	
5.50E-09	Contig261.0.g68	PF10551	MULE	MULE transposase domain	
5.40E-08	Contig2050.0.g38b	PF10551	MULE	MULE transposase domain	
1.20E-07	Contig14154.0.g51	PF10551	MULE	MULE transposase domain	
2.00E-07	Contig11552.0.g62	PF10551	MULE	MULE transposase domain	
5.40E-07	Contig21977.0.g75	PF10551	MULE	MULE transposase domain	
2.40E-05	Contig15275.0.g92	PF10551	MULE	MULE transposase domain	
0.00061	Contig414.1.g103	PF10551	MULE	MULE transposase domain	
7.70E-22	Contig1358.0.g71	PF03215	Rad17	Rad17 cell cycle checkpoint protein	
5.60E-11	Contig1358.0.g71	PF03215	Rad17	Rad17 cell cycle checkpoint protein	
5.20E-05	Contig21660.0.g93	PF03215	Rad17	Rad17 cell cycle checkpoint protein	
4.20E-17	Contig4212.0.g88c	PF13612	DDE_Tnp_1_3	Transposase DDE domain	
2.80E-15	Contig15014.0.g72	PF09778	Guanylate_cyc_2	Guanylylate cyclase	
9.80E-14	Contig22006.0.g86	PF04050	Upf2	Up-frameshift suppressor 2 	
3.80E-13	Contig271.1.g43	PF12397	U3snoRNP10	U3 small nucleolar RNA-associated protein 10	
500.0	Contig271.1.g43	PF12397	U3snoRNP10	U3 small nucleolar RNA-associated protein 10	
5.60E-13	Contig11820.0.g69	PF13695	zf-3CxxC	Zinc-binding domain	
1.00E-07	Contig12298.0.g44	PF13695	zf-3CxxC	Zinc-binding domain	
3.00E-08	Contig12629.0.g42	PF06943	zf-LSD1	LSD1 zinc finger	
7.20E-07	Contig12629.0.g42	PF06943	zf-LSD1	LSD1 zinc finger	
7.90E-08	Contig2050.0.g38	PF03101	FAR1	FAR1 DNA-binding domain	
1.90E-07	Contig14154.0.g51	PF03101	FAR1	FAR1 DNA-binding domain	
7.10E-07	Contig3272.0.g49	PF03101	FAR1	FAR1 DNA-binding domain	
0.00055	Contig18814.0.g95	PF03101	FAR1	FAR1 DNA-binding domain	
1.40E-07	Contig19665.0.g106	PF12923	RRP7	Ribosomal RNA-processing protein 7 (RRP7)	
6.2	Contig19665.0.g106	PF12923	RRP7	Ribosomal RNA-processing protein 7 (RRP7)	
7.90E-06	Contig2637.0.g64	PF12907	zf-met2	Zinc-binding	
0.00033	Contig2311.0.g58	PF12907	zf-met2	Zinc-binding	
9.30E-06	Contig22204.0.g75	PF08784	RPA_C	Replication protein A	
1.10E-05	Contig9844.0.g94	PF04500	FLYWCH	FLYWCH zinc finger domain	
7.60E-05	Contig19836.0.g32	PF04530	Viral_Beta_CD	Viral Beta C/D like family	
0.00011	Contig12218.0.g66	PF02002	TFIIE_alpha	TFIIE alpha subunit	
670.0	Contig12218.0.g66	PF02002	TFIIE_alpha	TFIIE alpha subunit	
0.00041	Contig16166.0.g42	PF05086	Dicty_REP	Dictyostelium (Slime Mold) REP protein	
0.00074	Contig22114.0.g34	PF13167	GTP-bdg_N	GTP-binding GTPase N-terminal	
1.3	Contig22114.0.g34	PF13167	GTP-bdg_N	GTP-binding GTPase N-terminal	
