# Supplementary material for: The Oxytricha trifallax Macronuclear Genome: A Complex Eukaryotic Genome with 16,000 Tiny Chromosomes
Source: PLoS Biol. 2013 Jan 29;11(1):e1001473. doi: 10.1371/journal.pbio.1001473 (PMC3558436; doi:10.1371/journal.pbio.1001473)
Supplement: Table S7 — Nucleic-acid-associated protein domains found in both Paramecium and Tetrahymena but not Oxytricha. Domains marked with * are present in translated ORFs, but were not originally detected as AUGUSTUS failed to predict them. Protein IDs are given for Tetrahymena. (RTF) [file pbio.1001473.s037.rtf]

Table S7. Nucleic-acid associated protein domains found in both Paramecium and Tetrahymena but not Oxytricha.
 
Domain i-Eval	Protein ID	Pfam ID	Domain name	Domain description	
5.00E-155	3831.m01522	PF05731	TROVE	TROVE domain	
2.70E-96	3825.m02594	PF03265	DNase_II	Deoxyribonuclease II	
8.50E-96	3825.m02591	PF03265	DNase_II	Deoxyribonuclease II	
1.00E-70	3812.m02544	PF03265	DNase_II	Deoxyribonuclease II	
8.60E-52	3825.m02590	PF03265	DNase_II	Deoxyribonuclease II	
1.40E-30	3715.m00008	PF03265	DNase_II	Deoxyribonuclease II	
1.50E-22	3825.m02590	PF03265	DNase_II	Deoxyribonuclease II	
2.50E-59	3832.m00153	PF02245	Pur_DNA_glyco	Methylpurine-DNA glycosylase	
7.00E-58	3673.m00009	PF02245	Pur_DNA_glyco	Methylpurine-DNA glycosylase	
4.10E-47	41.m00286	PF02137	A_deamin*	Adenosine deaminase	
5.60E-44	19.m00229	PF00445	Ribonuclease_T2	Ribonuclease T2	
5.20E-42	3813.m01636	PF00445	Ribonuclease_T2	Ribonuclease T2	
1.00E-40	3699.m00038	PF00445	Ribonuclease_T2	Ribonuclease T2	
1.00E-39	23.m00341	PF00445	Ribonuclease_T2	Ribonuclease T2	
2.50E-37	3704.m00018	PF00445	Ribonuclease_T2	Ribonuclease T2	
7.30E-19	139.m00077	PF00445	Ribonuclease_T2	Ribonuclease T2	
7.50E-19	139.m00081	PF00445	Ribonuclease_T2	Ribonuclease T2	
2.30E-18	139.m00079	PF00445	Ribonuclease_T2	Ribonuclease T2	
4.60E-21	227.m00035	PF00645	zf-PARP	Poly(ADP-ribose) polymerase and DNA-Ligase Zn-finger region	
1.70E-19	3734.m00016	PF00645	zf-PARP	Poly(ADP-ribose) polymerase and DNA-Ligase Zn-finger region	
3.90E-18	3828.m01316	PF00645	zf-PARP	Poly(ADP-ribose) polymerase and DNA-Ligase Zn-finger region	
2.50E-17	3676.m00059	PF00645	zf-PARP	Poly(ADP-ribose) polymerase and DNA-Ligase Zn-finger region	
1.50E-06	3710.m00083	PF00645	zf-PARP	Poly(ADP-ribose) polymerase and DNA-Ligase Zn-finger region	
1.60E-16	3822.m00628	PF04406	TP6A_N	Type IIB DNA topoisomerase	
1.10E-12	41.m00306	PF01974	tRNA_int_endo*	tRNA intron endonuclease, catalytic C-terminal domain	
9.10E-07	3825.m02533	PF01527	HTH_Tnp_1	Transposase	
0.0001	3825.m02533	PF01527	HTH_Tnp_1	Transposase	
2.20E-06	3708.m00952	PF01367	5_3_exonuc	5'-3' exonuclease, C-terminal SAM fold	
2.80E-06	3683.m00040	PF03835	Rad4	Rad4 transglutaminase-like domain	
9.60E-06	3677.m00087	PF01754	zf-A20	A20-like zinc finger	
0.00021	3810.m02101	PF03854	zf-P11	P-11 zinc finger	
0.00054	3706.m00133	PF03854	zf-P11	P-11 zinc finger	
