# Supplementary material for: The Oxytricha trifallax Macronuclear Genome: A Complex Eukaryotic Genome with 16,000 Tiny Chromosomes
Source: PLoS Biol. 2013 Jan 29;11(1):e1001473. doi: 10.1371/journal.pbio.1001473 (PMC3558436; doi:10.1371/journal.pbio.1001473)
Supplement: Table S8 — Data sources for genome assemblies. Data for the genome assemblies incorporated in the final meta-assembly may be downloaded from http://dx.doi.org/10.5061/dryad.d1013 [132]. (RTF) [file pbio.1001473.s038.rtf]

Table S8. Data sources for genome assemblies.


Assembly	Data source	Data details	Dryad DOI or Genbank accession for downloading	
TGI 2.1.8 assembly	Sanger reads and 454 reads	Sanger reads: Table S9. (Library prefixes OXAB-OXAO and 700 fosmid reads from OXAP). 454 reads: Table S10.	doi:10.5061/dryad.d1013
	
Fosmid assembly (used for validation only)	Sanger reads	Table S9. (Library prefix: OXAP, excluding 700 fosmid reads in TGI 2.1.8 assembly)	doi:10.5061/dryad.d1013
	
PE-Assembler/SSAKE assembly	Illumina paired end reads	Supporting Text: 'Illumina genomic library construction and sequencing'	doi:10.5061/dryad.d1013
	
IDBA assembly	Illumina paired end reads	Supporting Text: 'Illumina genomic library construction and sequencing'	doi:10.5061/dryad.d1013
	
ABySS assembly	Illumina paired end and single end reads	Supporting Text: 'Illumina genomic library construction and sequencing'	doi:10.5061/dryad.d1013
	
Meta-assembly	Primary source: Illumina assemblies
Secondary source: TGI assembly and Sanger reads	See description in 'Methods'	AMCR00000000; https://www.ncbi.nlm.nih.gov/bioproject/PRJNA74629	
