# Supplementary material for: The Oxytricha trifallax Macronuclear Genome: A Complex Eukaryotic Genome with 16,000 Tiny Chromosomes
Source: PLoS Biol. 2013 Jan 29;11(1):e1001473. doi: 10.1371/journal.pbio.1001473 (PMC3558436; doi:10.1371/journal.pbio.1001473)
Supplement: Table S9 — Genomic and RNA libraries Sanger sequenced on ABI3730 sequencers. (RTF) [file pbio.1001473.s039.rtf]

Table S9. Genomic and RNA libraries Sanger sequenced on an ABI3730.

Library sequence prefix	Vector	Plates	Comments	Insert size (kb)	
OXAB
	pSmart-HCKan
	2		1.2-1.5	
OXAC
	pSmart-HCKan
	2		1.0-1.2	
OXAD
	pSmart-HCKan
	2		0.8-1.0	
OXAE
	pSmart-HCKan
	62		2-4	
OXAF
	pSmart-HCKan
	1	Macronuclear DNA < 1.0 kb		
OXAG
	pSmart-HCKan
	1	Macronuclear DNA 2.0-3.0 kb		
OXAI
	pSmart-HCKan
	1	Macronuclear DNA 4.0-6.0 kb		
OXAJ
	pSmart-HCKan
	1	Macronuclear DNA 6.0-7.0 kb		
OXAK
	pSmart-HCKan
	1	Macronuclear DNA 7.0-8.0 kb		
OXAN
	pNZlinear
	6	20kb library		
OXAO
	potw13
	139	gDNA for WGS (1.5-4.0, 4.0-10 and
10-35 kb)	1-2 and 2-4 	
OXAO
	pSmart-HCKan
	204	gDNA for WGS		
OXAO
	pSmart-LCKan
	52	gDNA for WGS		
OXAP
	pcc01
	6	Fosmids (40 kb)		
OXAP
	pcc01
	353	Shotgun of fosmids	2-4	
OXAS
	pSmart-HCKan
	3	cDNAs		
OXAT
	pSmart-HCKan
	1	20kb Mitochondrial Library in pJAZZ-KA
		
OXAV
	pSmart-HCKan
	8	MIC Plasmid Subclones for Shotgun
		
OXAW
	pSmart-HCKan
	3	Mitochondrial Plasmid Subclones for Shotgun
		
OXAX
	pSmart-HCKan
	16	Mating RNA (0 hrs)
		
OXAY
	pSmart-HCKan
	16	Mating RNA (5 hrs) 
		
OXAZ
	pSmart-HCKan
	14	Mating RNA (13 hrs)
		
OXBA
	pSmart-HCKan
	15	Mating RNA (21 hrs)
		
OXBB
	pSmart-HCKan
	15	Mating RNA (30 hrs)
		
