# Supplementary material for: The Oxytricha trifallax Macronuclear Genome: A Complex Eukaryotic Genome with 16,000 Tiny Chromosomes
Source: PLoS Biol. 2013 Jan 29;11(1):e1001473. doi: 10.1371/journal.pbio.1001473 (PMC3558436; doi:10.1371/journal.pbio.1001473)
Supplement: Table S10 — 454 genomic DNA libraries. Short read archive data can be downloaded from http://www.ncbi.nlm.nih.gov/sra. (RTF) [file pbio.1001473.s040.rtf]

Table S10. 454 genomic DNA libraries.

Nanochromo-some size range (kb)	Library accession	Short read archive run ID	Library size (Mb)	
0.25-4.0	E8NW6VF01 E8NW6VF02	NA	~130	
4-10	E8GCV7U01 E8GCV7U02
FBJDESE01
FBJDESE02
FAVAZAW01
FAVAZAW02	NA	~270	
10-35	FA42LMG01
FA42LMG02	SRR005477	84.9	
> 7 kb	EYOHBTJ01
EYOHBTJ02
EX9PBLC01
EX9PBLC02
EX9PBLC03
EX9PBLC04	SRR000202
SRR000199
SRR000203
SRR000200
SRR000201
SRR000204	70.4
75.2
0.9
1.3
2.1
1.3	
Circular 1-2	F5BXT2T01
F5BXT2T02	NA	68.3
75.6	
Circular 2-3	F2M9Y7L01
F2M9Y7L02	NA	237
245	
Circular 3-5	F5I6MJ401
F5I6MJ402	NA	195
158	
