# Supplementary material for: The Oxytricha trifallax Macronuclear Genome: A Complex Eukaryotic Genome with 16,000 Tiny Chromosomes
Source: PLoS Biol. 2013 Jan 29;11(1):e1001473. doi: 10.1371/journal.pbio.1001473 (PMC3558436; doi:10.1371/journal.pbio.1001473)
Supplement: Table S11 — Meta-contig statistics after first CAP3 assembly before extension. “Single” refers to an SE being complete (≥1 5′ or 3′ telomeres). “Both” refers to one or more telomeres on both ends of the contig (≥1 5′ and ≥1 3′ ends). “Multiple” refers to greater than two ends on either end of the contig (≥2 5′ or ≥2 3′ ends). All lengths are given in bp. (RTF) [file pbio.1001473.s041.rtf]

Table S11. Meta-contig statistics after first CAP3 assembly before extension. 

	both telomeres	single telomere	zero telomeres	multiple telomeres	
number	7,841	11,174	6,949	854	
total length	26,300,000	26,000,000	12,200,000	4,300,000	
mean length	3,356	2,329	1,755	5,004	
std length	2,404	2,357	1,816	3,235	
max length	27,060	65,810	20,577	24,700	
min length	314	100	49	337	
