# Supplementary material for: The Oxytricha trifallax Macronuclear Genome: A Complex Eukaryotic Genome with 16,000 Tiny Chromosomes
Source: PLoS Biol. 2013 Jan 29;11(1):e1001473. doi: 10.1371/journal.pbio.1001473 (PMC3558436; doi:10.1371/journal.pbio.1001473)
Supplement: Table S12 — Meta-contig statistics after first extension. “Single” refers to an SE being complete (≥1 5′ or 3′ telomeres). “Both” refers to one or more telomeres on both ends of the contig (≥1 5′ and ≥1 3′ ends). “Multiple” refers to greater than two ends on either end of the contig (≥2 5′ or ≥2 3′ ends). All lengths are given in bp. (RTF) [file pbio.1001473.s042.rtf]

Table S12. Meta-contig statistics after first extension.

	both telomeres	single telomere	zero telomeres	multiple telomeres	
number	16,992	7,335	1,637	1,650	
total length	58,800,000	17,800,000	2,800,000	9,000,000	
mean length	3,458	2,427	1,683	5,468	
std length	2,601	2,719	1,785	3,598	
max length	66,022	65,810	24,070	24,700	
min length	314	100	49	343	
