# Supplementary material for: The Oxytricha trifallax Macronuclear Genome: A Complex Eukaryotic Genome with 16,000 Tiny Chromosomes
Source: PLoS Biol. 2013 Jan 29;11(1):e1001473. doi: 10.1371/journal.pbio.1001473 (PMC3558436; doi:10.1371/journal.pbio.1001473)
Supplement: Table S13 — Meta-contig statistics after CAP3 reassembly of extended contigs. “Single” refers to an SE being complete (≥1 5′ or 3′ telomeres). “Both” refers to one or more telomeres on both ends of the contig (≥1 5′ and ≥1 3′ ends). “Multiple” refers to greater than two ends on either end of the contig (≥2 5′ or ≥2 3′ ends). All lengths are given in bp. (RTF) [file pbio.1001473.s043.rtf]

Table S13. Meta-contig statistics after CAP3 reassembly of extended contigs.

	both telomeres	single telomere	zero telomeres	multiple telomeres	
number	16,009	6,072	1,419	1,513	
total length	54,800,000	14,700,000	2,300,000	8,300,000	
mean length	3,421	2,422	1,595	5,463	
std length	2,587	2,726	1,729	3,605	
max length	66,022	65,810	24,070	24,700	
min length	314	100	49	343	
