# Supplementary material for: The Oxytricha trifallax Macronuclear Genome: A Complex Eukaryotic Genome with 16,000 Tiny Chromosomes
Source: PLoS Biol. 2013 Jan 29;11(1):e1001473. doi: 10.1371/journal.pbio.1001473 (PMC3558436; doi:10.1371/journal.pbio.1001473)
Supplement: Table S14 — Meta-contig statistics after second extension. “Single” refers to an SE being complete (≥1 5′ or 3′ telomeres). “Both” refers to one or more telomeres on both ends of the contig (≥1 5′ and ≥1 3′ ends). “Multiple” refers to greater than two ends on either end of the contig (≥2 5′ or ≥2 3′ ends). All lengths are given in bp. (RTF) [file pbio.1001473.s044.rtf]

Table S14. Meta-contig statistics after second extension.

	both telomeres	single telomere	zero telomeres	multiple telomeres	
number	17,789	4,677	1,034	1,707	
total length	62,200,000	11,000,000	1,400,000	9,500,000	
mean length	3,499	2,354	1,390	5,547	
std length	2,763	2,279	1,598	3,636	
max length	66,022	30,067	27,555	30,135	
min length	314	100	49	305	
