# Supplementary material for: The Oxytricha trifallax Macronuclear Genome: A Complex Eukaryotic Genome with 16,000 Tiny Chromosomes
Source: PLoS Biol. 2013 Jan 29;11(1):e1001473. doi: 10.1371/journal.pbio.1001473 (PMC3558436; doi:10.1371/journal.pbio.1001473)
Supplement: Table S15 — Meta-contig statistics after CAP3 reassembly of second round of extended contigs. “Single” refers to an SE being complete (≥1 5′ or 3′ telomeres). “Both” refers to one or more telomeres on both ends of the contig (≥1 5′ and ≥1 3′ ends). “Multiple” refers to greater than two ends on either end of the contig (≥2 5′ or ≥2 3′ ends). All lengths are given in bp. (RTF) [file pbio.1001473.s045.rtf]

Table S15. Meta-contig statistics after CAP3 reassembly of 2nd round of extended contigs.

	both telomeres	single telomere	zero telomeres	multiple telomeres	
number	17,616	4,516	1,020	1,691	
total length	61,500,000	10,500,000	1,400,000	9,400,000	
mean length	3,492	2,325	1,348	5,544	
std length	2,764	2,264	1,369	3,631	
max length	66,022	30,067	17,188	30,135	
min length	314	100	49	305	
