# Supplementary material for: The Oxytricha trifallax Macronuclear Genome: A Complex Eukaryotic Genome with 16,000 Tiny Chromosomes
Source: PLoS Biol. 2013 Jan 29;11(1):e1001473. doi: 10.1371/journal.pbio.1001473 (PMC3558436; doi:10.1371/journal.pbio.1001473)
Supplement: Table S16 — Meta-contig statistics after chimera splitting and end trimming. “Single” refers to an SE being complete (≥1 5′ or 3′ telomeres). “Both” refers to one or more telomeres on both ends of the contig (≥1 5′ and ≥1 3′ ends). “Multiple” refers to greater than two ends on either end of the contig (≥2 5′ or ≥2 3′ ends). All lengths are given in bp. (RTF) [file pbio.1001473.s046.rtf]

Table S16. Meta-contig statistics after chimera splitting and end trimming.

	both telomeres	single telomere	zero telomeres	multiple telomeres	
number	16,174	7,089	1,469	1,386	
total length	51,600,000	18,200,000	2,500,000	5,700,000	
mean length	3,188	2,570	1,734	4,094	
std length	2,487	2,135	1,624	2,875	
max length	66,022	28,867	13,395	28,867	
min length	314	102	3	305	
