# Supplementary material for: The Oxytricha trifallax Macronuclear Genome: A Complex Eukaryotic Genome with 16,000 Tiny Chromosomes
Source: PLoS Biol. 2013 Jan 29;11(1):e1001473. doi: 10.1371/journal.pbio.1001473 (PMC3558436; doi:10.1371/journal.pbio.1001473)
Supplement: Table S17 — Meta-contig statistics for the final CAP3 assembly. “Single” refers to an SE being complete (≥1 5′ or 3′ telomeres). “Both” refers to one or more telomeres on both ends of the contig (≥1 5′ and ≥1 3′ ends). “Multiple” refers to greater than two ends on either end of the contig (≥2 5′ or ≥2 3′ ends). All lengths are given in bp. (RTF) [file pbio.1001473.s047.rtf]

Table S17. Meta-contig statistics for the final CAP3 assembly.

	both telomeres	single telomere	zero telomeres	multiple telomeres	
number	15,993	5,303	1,154	1,279	
total length	51,000,000	14,300,000	1,900,000	5,400,000	
mean length	3,187	2,694	1,655	4,208	
std length	2,475	2,135	1,544	2,921	
max length	66,022	22,354	13,395	29,046	
min length	314	102	3	305	
