# Supplementary material for: The Oxytricha trifallax Macronuclear Genome: A Complex Eukaryotic Genome with 16,000 Tiny Chromosomes
Source: PLoS Biol. 2013 Jan 29;11(1):e1001473. doi: 10.1371/journal.pbio.1001473 (PMC3558436; doi:10.1371/journal.pbio.1001473)
Supplement: Table S18 — Properties of intergenic regions. Prediction features were obtained for complete nanochromosomes (14,388 in total) only. Intergenic regions are between start and stop codons, including UTRs. Alternative fragmentation sites are those that are strongly supported by Illumina telomeric reads. %GC estimates exclude telomeric bases. Intergenic regions are subdivided according to whether they have a site of alternative fragmentation within the region or not. (RTF) [file pbio.1001473.s048.rtf]

Table S18. Properties of intergenic regions. 

Feature	Alternat-ively fragmented	Number	Mean (bp)	Min  (bp)	Max  
(bp)	%GC	
Unidirectional intergenic region	Yes	329	319	96	2,660	20.4	
	No	561	381	92	3,197	23.1	
Convergent intergenic region	Yes	300	249	63	1,598	20.3	
	No	471	229	53	1,945	20.2	
Divergent intergenic region	Yes	149	320	96	2,660	20.4	
	No	260	330	132	1,555	23.0	
