# Supplementary material for: The Oxytricha trifallax Macronuclear Genome: A Complex Eukaryotic Genome with 16,000 Tiny Chromosomes
Source: PLoS Biol. 2013 Jan 29;11(1):e1001473. doi: 10.1371/journal.pbio.1001473 (PMC3558436; doi:10.1371/journal.pbio.1001473)
Supplement: Table S19 — Missing Moco biosynthesis enzymes in ciliates. (RTF) [file pbio.1001473.s049.rtf]

Table S19. Missing Moco biosynthesis enzymes in ciliates.

Human enzyme name	Oxytricha homolog	Oligohymenophorean homologs	
MOCS1A	Contig1362.1.g29	Absent	
MOCS1B	Contig1786.0.1.g95 
Contig14433.0.1.g80
 Contig4549.0.g103 	Absent	
MOCS2A	Contig19982.0.g77	Absent	
MOCS2B	Contig19520.0.g100
 Contig21901.0.0 
Contig21901.0.0.g6	Absent	
MOCS3	Contig12255.0.g43	TTHERM_00530210 (Tetrahymena)
GSPATT00003659001
(Paramecium)	
Gephyrin G	Contig1475.1.g53	Absent	
Gephyrin E	Contig1475.1.g53	Absent	
