# Supplementary material for: The Oxytricha trifallax Macronuclear Genome: A Complex Eukaryotic Genome with 16,000 Tiny Chromosomes
Source: PLoS Biol. 2013 Jan 29;11(1):e1001473. doi: 10.1371/journal.pbio.1001473 (PMC3558436; doi:10.1371/journal.pbio.1001473)
Supplement: Table S20 — Small ribosomal proteins. Gene identifiers are given as contig identifiers with a gene suffix beginning with “g” followed by a number (which is arbitrary in this context). Only proteins ≤100 aa with domains found in Pfam 26.0 with an E-value<0.01 and with some homologs in UniProt that are ≤120 aa (to ensure that they are genuine small proteins) are listed. Where alternative fragmentation occurs, the nanochromosome length of the shortest putative isoform encoding the small protein is shown. Protein domains are taken from Pfam 26.0. Contig22302.0 is a multigene nanochromosome. aThese proteins are incorrectly predicted as gene fusions on the longer ribosomal/nonribosomal protein-encoding nanochromosome. (RTF) [file pbio.1001473.s050.rtf]

Table S20. Small ribosomal proteins.

Gene	Small protein (Pfam domain)	Protein length (aa)	Nanochromosome length (bp)	Alternatively fragmented	
Contig144.0.g19	rps21e	82	640	Yes	
Contig4448.0.g28	rps27ea	80	613	Yes	
Contig12436.0.g46	rps28e	69	642	Yes	
Contig16083.0.g100	rps30	70	698	Yes	
Contig22302.0.g104	rpl29e	61	1596	No	
Contig1196.1.g75	rpl37e	96	609	Yes	
Contig19246.0.g31	rpl37ae	100	647	Yes	
Contig6413.0.g98	rpl38	77	667	Yes	
Contig11091.0.g30	rpl39a	67	620	Yes	
Contig22113.0.g42	rpl41a	22	611	Yes	
