# Supplementary material for: The Oxytricha trifallax Macronuclear Genome: A Complex Eukaryotic Genome with 16,000 Tiny Chromosomes
Source: PLoS Biol. 2013 Jan 29;11(1):e1001473. doi: 10.1371/journal.pbio.1001473 (PMC3558436; doi:10.1371/journal.pbio.1001473)
Supplement: Table S21 — Small nonribosomal proteins. Gene identifiers are given as contig identifiers with a gene suffix beginning with “g” followed by a number (which is arbitrary in this context). All nanochromosomes in this table longer than 1 kb are predicted to be multigene nanochromosomes. Proteins 50–100 aa long with domains found in Pfam 26.0 (independent E-value<0.01) and with at least some homologs in UniProt that are ≤120 aa (to ensure that they are genuine small proteins) are listed. We excluded a few proteins that appeared to be truncated by incomplete assembly of their nanochromosomes. Nanochromosome lengths exclude telomeres. Where alternative fragmentation occurs, the nanochromosome length of the shortest putative isoform encoding the small protein is shown. Protein domain names are from Pfam 26.0. (RTF) [file pbio.1001473.s051.rtf]

Table S21. Small nonribosomal proteins.

Gene (on contig)	Small protein (Pfam domain)	Protein length (aa)	Nanochromosome length (bp)	Alternatively fragmented	
Contig7120.0.g58	ACPB	87	617	No	
Contig19552.0.g52	ACPB	95	815	No	
Contig442.1.g3	ATP_synt_H	79	4221	No	
Contig12965.0.g1	BolA	89	530	No	
Contig2366.0.g5	CKS	89	>4781	Yes	
Contig15320.0.g74	CKS	100	843	No	
Contig6369.0.g40	Complex1_LYR	100	5962	Yes	
Contig22295.0.g17	COX17	75	3351	Yes	
Contig1030.1.g108	COX17	87	5385	Yes	
Contig22375.0.g58	Cystatin	94	1506	Yes	
Contig11075.0.g35	Cyt-B5	97	885	No	
Contig10745.0.g100	DPM2	99	1836	Yes	
Contig4662.0.g106	Dpy-30	57	558	Yes	
Contig12107.0.g111	Dpy-30	70	772	No	
Contig14329.0.g32	dsDNA_bind	98	723	Yes	
Contig11127.0.g63	Dynein_light	73	5002	No	
Contig6144.0.g67	Dynein_light	89	646	No	
Contig12072.0.g49	EF_hand_5	84	650	No	
Contig18742.0.g67	efhand	92	588	Yes	
Contig19592.0.g15	Elf1	92	779	No	
Contig13980.0.g86	Evr1_Alr	90	4223	No	
Contig16998.0.g95	Fer2	89	2821	Yes	
Contig22565.0.g62	HMA	88	647	No	
Contig10117.0.g15	HSBP1	85	1048	No	
Contig14303.0.g107	LSM	79	2905	No	
Contig152.1.g18	LSM	81	2617	Yes	
Contig13737.0.g78	LSM	89	2928	Yes	
Contig19089.0.g58	LSM	91	722	No	
Contig4137.0.g49	LSM	99	>822	No	
Contig18757.0.g51	MOZART1	94	810	No	
Contig4029.0.g36	Pet191_N	88	>5925	No	
Contig15069.0.g61	Prefoldin_2	100	727	No	
Contig13324.0.g11	RAMP4	68	878	No	
Contig4686.0.g38	RNA_pol_N	71	3064	Yes	
Contig16788.0.g28	RRM_1	83	2532	No	
Contig3371.0.0.g49	sec61 gamma	76	596	No	
Contig19586.0.g90	Sec61_beta	90	841	No	
Contig1444.1.g5	SNARE	96	4628	Yes	
Contig11849.0.g52	tbf5	73	2011	No	
Contig431.1.g124	Tctex-1 	53	4908	Yes	
Contig19982.0.g77	ThiS	59	469	No	
Contig14701.0.g38	Ubiquitin	94	628	No	
Contig20641.0.0.g15	UPF0197	87	>4256	No	
Contig20146.0.g34	zf-CSL	87	2240	Yes	
Contig19561.0.g78	zf-H2C2_2 	95	2008	No	
Contig254.1.g19	zf-rbx1	96	2177	No	
Contig16087.0.g105	zf-RING_2	83	840	No	
Contig1988.0.g15	zf-Tim10_DDP	63	541	Yes	
Contig11876.0.g101	zf-Tim10_DDP	68	627	Yes	
Contig15415.0.0.g25	zf-Tim10_DDP	69	>3675	Yes	
Contig14329.0.g33	zf-Tim10_DDP	70	1353	Yes	
Contig760.1.g39	zf-Tim10_DDP	76	555	Yes	
Contig757.1.g34	zf-Tim10_DDP	77	1057	Yes	
Contig22868.0.g62	zf-Tim10_DDP	82	655	No	
Contig14811.0.g78	zf-Tim10_DDP	95	594	No	
Contig144.1.g8	zf-Tim10_DDP	95	>6596	No	
