# Supplementary material for: The Oxytricha trifallax Macronuclear Genome: A Complex Eukaryotic Genome with 16,000 Tiny Chromosomes
Source: PLoS Biol. 2013 Jan 29;11(1):e1001473. doi: 10.1371/journal.pbio.1001473 (PMC3558436; doi:10.1371/journal.pbio.1001473)
Supplement: Table S22 — RNA-seq counts for transcription initiation factor II domain protein genes. RNA expression values are given in normalized read counts for vegetative (“Fed”) cells and cells developing during conjugation (see Text S1: RNA-seq mapping and read counting). (RTF) [file pbio.1001473.s052.rtf]

Table S22. RNA-seq counts for transcription initiation factor II domain protein genes.


Gene name	Fed	0 hrs	10 hrs	20 hrs	40 hrs	60 hrs	Pfam domains	
Contig17369.0.g57	16	257	105	115	244	279	1x BRF1	1x TF_Zn_Ribbon	2x TFIIB	
Contig10554.0.g23	0	359	204	585	60	711	1x RNA_POL_M_15KD	1x TFIIS_C		
Contig13665.0.g61	1039	985	1109	890	343	372	1x TFIIA			
Contig14147.0.g88	133	291	237	121	202	170	1x TFIIA_gamma_N	1x TFIIA_gamma_C		
Contig237.1.g127	59	0	474	386	295	265	1x TFIID_20kDa			
Contig4406.0.g109	94	0	315	139	80	56	1x TFIID_30kDa			
Contig12484.0.g45	8	38	463	344	270	174	1x TFIID-18kDa			
Contig16730.0.g8	8	38	462	346	271	175	1x TFIID-18kDa			
Contig14938.0.g40	8	4	61	188	73	6	1x TFIIF_beta			
Contig15803.0.g96	0	15	24	39	15	16	1x TFIIF_beta			
Contig664.0.g57	8	8	56	189	75	6	1x TFIIF_beta			
Contig1015.0.g5	0	0	2315	918	311	124	1x TFIIS_C	2x TFIIS_M		
Contig14486.0.g34	0	0	2444	949	334	138	1x TFIIS_C	1x TFIIS_M		
Contig1475.1.g54	31	0	14	42	44	9	1x TFIIS_C			
Contig22233.0.g55	27	525	697	280	83	42	1x TFIIS_C	1x TFIIS_M	1x Med26	
Contig9889.0.g85	0	0	1295	480	454	443	1x TFIIS_M			
Contig4998.0.g90	8	83	856	337	184	399	2x TFIIB	1x TF_Zn_Ribbon		
Contig4674.0.g42	933	728	2461	1166	588	247	2x TFIID-31kDa			
Contig12218.0.g66	0	128	27	6	59	59	2x TFIIE_alpha			
Contig517.1.g109	0	0	5628	2097	1146	707	2x TFIIF_beta			
Contig922.1.g114	0	0	476	44	186	83	6x WD40	1x EF_hand_5	1x TFIID_90kDa	
Contig798.1.g90	3948	2834	2347	2392	1007	4308	2x TAF4			
Contig11849.0.g52	0	0	11	0	1	1	1x Tbf5			
Contig8748.0.g3	0	0	205	279	175	132	1x TAFII28			
Contig19222.0.g62	71	8	128	69	154	186	1x TAFII55_N			
Contig262.1.g32	843	351	638	707	578	506	2x TBP			
Contig9118.0.g56	0	0	0	0	218	239	2x TBP			
