# Supplementary material for: The Oxytricha trifallax Macronuclear Genome: A Complex Eukaryotic Genome with 16,000 Tiny Chromosomes
Source: PLoS Biol. 2013 Jan 29;11(1):e1001473. doi: 10.1371/journal.pbio.1001473 (PMC3558436; doi:10.1371/journal.pbio.1001473)
Supplement: Table S23 — Top 40 elevated domain counts in Oxytricha relative to Tetrahymena. The “enrichment” column measures the number of UCLUST clustered proteins in Oxytricha relative to proteins in Tetrahymena. The columns after “enrichment” count the number of proteins in which the Pfam domains are found for Oxytricha (Oxy), Tetrahymena (Tet), Paramecium (Par), and Perkinsus (Per). Nucleic-acid-related domains (bold) were classified from Pfam domains annotated with the “nucleic acid binding” GO term (GO:0003676) in pfam2go. Where there were missing Pfam domain annotations in pfam2go or there was literature associated with the Pfam identifier that suggested the domain was nucleic acid binding, we also classified the protein as nucleic-acid-related. (RTF) [file pbio.1001473.s053.rtf]

Table S23. Top 40 elevated domain counts in Oxytricha relative to Tetrahymena.

Pfam ID	Pfam name	Pfam description	nucleic acid-related	Enrichment	Oxy	Tet 	Par	Per	
PF00046	Homeobox	Homeobox domain	+	31.0	31	1	3	0	
PF02187	GAS2	Growth-Arrest-Specific Protein 2 Domain	-	28.0	28	1	0	0	
PF00909	Ammonium_transp	Ammonium Transporter Family		16.0	16	1	20	8	
PF06011	TRP	Transient receptor potential (TRP) ion channel		15.0	15	1	2	4	
PF13465	zf-H2C2_2	Zinc-finger double domain	+	13.0	65	5	8	0	
PF07534	TLD	TLD		12.2	146	12	72	3	
PF07707	BACK	BTB And C-terminal Kelch		10.0	10	1	2	2	
PF01918	Alba	Alba	+	9.0	9	1	17	8	
PF00658	PABP	Poly-adenylate binding protein, unique domain	+	8.5	17	2	7	5	
PF00535	Glycos_transf_2	Glycosyl transferase family 2		8.5	17	2	4	15	
PF00096	zf-C2H2	Zinc finger, C2H2 type	+	8.0	40	5	10	1	
PF03931	Skp1_POZ	Skp1 family, tetramerisation domain	-	8.0	8	1	5	8	
PF01067	Calpain_III	Calpain large subunit, domain III		8.0	8	1	5	0	
PF02197	RIIa	Regulatory subunit of type II PKA R-subunit	-	8.0	8	1	6	0	
PF00571	CBS	CBS domain	-	7.0	7	1	7	10	
PF03820	Mtc	Tricarboxylate carrier	-	7.0	7	1	2	9	
PF13385	Laminin_G_3	Concanavalin A-like lectin/glucanases superfamily		6.5	26	4	21	2	
PF00459	Inositol_P	Inositol monophosphatase family	-	6.0	6	1	3	3	
PF01214	CK_II_beta	Casein kinase II regulatory subunit	-	6.0	6	1	12	4	
PF00620	RhoGAP	RhoGAP domain	-	6.0	6	1	13	0	
PF03171	2OG-FeII_Oxy	2OG-Fe(II) oxygenase superfamily	-	6.0	6	1	0	6	
PF13908	Shisa	Wnt and FGF inhibitory regulator		6.0	6	1	0	0	
PF00643	zf-B_box	B-box zinc finger	+	5.9	184	31	57	6	
PF13188	PAS_8	PAS domain		5.0	15	3	6	0	
PF08005	PHR	PHR domain 		5.0	10	2	3	1	
PF13621	Cupin_8	Cupin-like domain		5.0	10	2	2	12	
PF11798	IMS_HHH	IMS family HHH motif	+	5.0	5	1	2	2	
PF05277	DUF726	Protein of unknown function (DUF726)		5.0	5	1	5	3	
PF05301	Mec-17	Touch receptor neuron protein Mec-17	-	5.0	5	1	5	1	
PF04145	Ctr	Ctr copper transporter family	-	5.0	5	1	5	2	
PF00294	PfkB	pfkB family carbohydrate kinase		5.0	5	1	5	19	
PF05188	MutS_II	MutS domain II	+	5.0	5	1	4	1	
PF02685	Glucokinase	Glucokinase	-	5.0	5	1	1	1	
PF00626	Gelsolin	Gelsolin repeat		5.0	5	1	6	5	
PF04057	Rep-A_N	Replication factor-A protein 1, N-terminal domain	+	5.0	5	1	0	3	
PF01981	PTH2	Peptidyl-tRNA hydrolase PTH2	-	5.0	5	1	2	3	
PF00817	IMS	impB/mucB/samB family	+	4.5	9	2	4	3	
PF13886	DUF4203	Domain of unknown function (DUF4203)		4.5	9	2	5	1	
PF13445	zf-RING_LisH	RING-type zinc-finger, LisH dimerisation motif		4.0	20	5	14	2	
PF03810	IBN_N	Importin-beta N-terminal domain	-	4.0	12	3	11	18	
