# Supplementary material for: The Oxytricha trifallax Macronuclear Genome: A Complex Eukaryotic Genome with 16,000 Tiny Chromosomes
Source: PLoS Biol. 2013 Jan 29;11(1):e1001473. doi: 10.1371/journal.pbio.1001473 (PMC3558436; doi:10.1371/journal.pbio.1001473)
Supplement: Table S24 — RNA-seq counts for homeodomain protein genes. RNA expression values are given in normalized read counts for vegetative (“Fed”) cells and cells developing during conjugation (see Text S1: RNA-seq mapping and read counting). (RTF) [file pbio.1001473.s054.rtf]

Table S24. RNA-seq counts for homeodomain protein genes.

Gene name	Fed	0 hrs	10 hrs	20 hrs	40 hrs	60 hrs	Pfam domain	
Contig1106.0.g3	0	8	0	0	0	1	1x Homeobox	
Contig12395.0.2.g17	333	102	20	13	46	66	1x Homeobox	
Contig13221.0.g5	8	8	92	317	155	56	1x Homeobox	
Contig13416.0.g86	0	0	0	0	0	1	1x Homeobox	
Contig14290.0.g8	349	309	179	455	421	430	1x Homeobox	
Contig14695.0.g7	0	0	0	0	0	1	1x Homeobox	
Contig15427.0.g9	110	38	0	0	20	2	1x Homeobox	
Contig15999.0.g111	0	0	0	7	1	4	3x Homeobox	
Contig1600.0.g71	0	0	0	0	1	8	1x Homeobox	
Contig17119.0.g24	47	38	46	143	84	54	1x Homeobox	
Contig17646.0.g10	196	8	128	139	99	65	1x Homeobox	
Contig18801.0.g18	0	8	53	12	62	49	1x Homeobox	
Contig18912.0.g54	8	8	122	285	180	448	1x Homeobox_KN	
Contig19293.0.g20	0	0	6	0	10	8	1x Homeobox	
Contig19316.0.g105	0	0	7	0	4	12	2x Homeobox	
Contig19558.0.g51	0	423	200	167	102	118	1x Homeobox	
Contig19622.0.g103	118	162	30	1	15	75	1x Homeobox	
Contig20133.0.g105	55	0	2	16	341	168	1x Homeobox	
Contig20279.0.g48	5599	2400	2390	669	669	459	1x Homeobox	
Contig20340.0.g88	0	0	0	0	2	1	1x Homeobox	
Contig20448.0.g83	0	0	0	51	6	41	2x Homeobox	
Contig20645.0.g12	0	83	3	0	109	33	3x Homeobox	
Contig20897.0.g1	0	0	120	55	20	17	1x Homeobox	
Contig21166.0.g49	345	309	179	452	417	431	1x Homeobox	
Contig21879.0.g73	0	0	0	7	1	3	1x Homeobox	
Contig21993.0.g119	153	143	112	220	496	1149	1x Homeobox	
Contig22307.0.g2	0	0	4	17	1	9	1x Homeobox	
Contig2250.0.g22	0	8	4	0	11	2	1x Homeobox	
Contig22715.0.g74	31	8	86	47	70	71	1x Homeobox_KN	
Contig3077.0.g101	0	23	4	7	24	31	1x Homeobox	
Contig4124.0.g49	0	4	4	0	11	2	1x Homeobox	
Contig535.1.g133	0	0	2	8	5	16	1x Homeobox	
Contig5570.0.g9	0	83	3	0	111	34	4x Homeobox	
Contig614.1.g103	400	53	205	136	148	260	1x Homeobox	
Contig6580.0.g110	0	4	3	0	10	2	1x Homeobox	
Contig6836.0.g67	0	4	3	0	10	2	1x Homeobox	
Contig7348.0.g53	0	0	0	0	0	1	1x Homeobox	
Contig8571.0.g1	39	192	21	60	111	114	1x Homeobox	
