# Supplementary material for: The Oxytricha trifallax Macronuclear Genome: A Complex Eukaryotic Genome with 16,000 Tiny Chromosomes
Source: PLoS Biol. 2013 Jan 29;11(1):e1001473. doi: 10.1371/journal.pbio.1001473 (PMC3558436; doi:10.1371/journal.pbio.1001473)
Supplement: Table S25 — Zinc finger protein domain counts in Oxytricha. The “enrichment” column measures the number of UCLUST clustered proteins in Oxytricha relative to proteins in Tetrahymena. The columns after “enrichment” count the number of proteins in which the Pfam domains are found for Oxytricha (Oxy), Tetrahymena (Tet), Paramecium (Par), Ichthyophthirius (Ich), and Perkinsus (Per). (RTF) [file pbio.1001473.s055.rtf]

Table S25. Zinc finger protein domain counts in Oxytricha.

Pfam ID	Pfam name	Pfam description	Enrichment	Oxy	Tet	Ich	Par	Per	
PF13465	zf-H2C2_2	Zinc-finger double domain	13.0	65	5	0	8	0	
PF00096	zf-C2H2	Zinc finger, C2H2 type	8.0	40	5	0	10	1	
PF00643	zf-B_box	B-box zinc finger	5.9	184	31	8	57	6	
PF13445	zf-RING_LisH	RING-type zinc-finger, LisH dimerisation motif	4.0	20	5	3	14	2	
PF13452	zf-MaoC	Metal-binding domain of MaoC dehydratase	4.0	4	1	1	2	1	
PF07535	zf-DBF	DBF zinc finger	4.0	4	1	0	8	3	
PF09360	zf-CDGSH	Iron-binding zinc finger CDGSH type	3.5	7	2	1	6	2	
PF02953	zf-Tim10_DDP	Tim10/DDP family zinc finger	3.2	19	6	0	17	12	
PF13832	zf-HC5HC2H_2	PHD-zinc-finger like domain	2.4	12	5	5	12	6	
PF13894	zf-C2H2_4	C2H2-type zinc finger	2.0	4	2	0	2	0	
PF01396	zf-C4_Topoisom	Topoisomerase DNA binding C4 zinc finger	2.0	2	1	0	3	0	
PF11789	zf-Nse	Zinc-finger of the MIZ type in Nse subunit	2.0	2	1	1	5	1	
PF14369	zf-RING_3	zinc-finger	2.0	2	1	0	0	4	
PF14260	zf-C4pol	C4-type zinc-finger of DNA polymerase delta	1.7	5	3	2	3	2	
PF13923	zf-C3HC4_2	Zinc finger, C3HC4 type (RING finger)	1.6	94	58	13	147	26	
PF12678	zf-rbx1	RING-H2 zinc finger	1.6	13	8	0	20	7	
PF01753	zf-MYND	MYND finger	1.5	9	6	3	36	10	
PF01428	zf-AN1	AN1-like Zinc finger	1.5	6	4	1	8	8	
PF05253	zf-U11-48K	U11-48K-like CHHC zinc finger	1.5	3	2	1	5	3	
PF10497	zf-4CXXC_R1	Zinc-finger domain of monoamine-oxidase A repressor R1	1.5	3	2	0	4	0	
PF04810	zf-Sec23_Sec24	Sec23/Sec24 zinc finger	1.4	7	5	2	12	9	
PF01529	zf-DHHC	DHHC palmitoyltransferase	1.4	65	47	19	112	38	
PF13639	zf-RING_2	Ring finger domain	1.3	172	129	11	290	111	
PF13913	zf-C2HC_2	zinc-finger of a C2HC-type	1.3	12	9	2	32	5	
PF00097	zf-C3HC4	Zinc finger, C3HC4 type (RING finger)	1.3	9	7	2	18	0	
PF02148	zf-UBP	Zn-finger in ubiquitin-hydrolases and other protein	1.3	9	7	3	6	7	
PF12171	zf-C2H2_jaz	Zinc-finger double-stranded RNA-binding	1.3	5	4	3	13	7	
PF04438	zf-HIT	HIT zinc finger	1.3	5	4	0	6	2	
PF00642	zf-CCCH	Zinc finger C-x8-C-x5-C-x3-H type (and similar)	1.0	25	25	11	61	103	
PF12756	zf-C2H2_2	C2H2 type zinc-finger (2 copies)	1.0	3	3	2	3	4	
PF13771	zf-HC5HC2H	PHD-like zinc-binding domain	1.0	3	3	1	22	0	
PF06221	zf-C2HC5	Putative zinc finger motif, C2HC5-type	1.0	1	1	1	1	0	
PF06220	zf-U1	U1 zinc finger	1.0	1	1	0	2	2	
PF13912	zf-C2H2_6	C2H2-type zinc finger	1.0	1	1	0	4	0	
PF03367	zf-ZPR1	ZPR1 zinc-finger domain	1.0	1	1	1	5	1	
PF05180	zf-DNL	DNL zinc finger	1.0	1	1	1	1	2	
PF07967	zf-C3HC	C3HC zinc finger-like 	1.0	1	1	0	1	0	
PF12251	zf-SNAP50_C	snRNA-activating protein of 50kDa MW C terminal	1.0	1	1	1	1	2	
PF10276	zf-CHCC	Zinc-finger domain	1.0	1	1	1	4	0	
PF08790	zf-LYAR	LYAR-type C2HC zinc finger 	1.0	1	1	0	2	2	
PF13920	zf-C3HC4_3	Zinc finger, C3HC4 type (RING finger)	0.9	36	40	10	101	33	
PF02891	zf-MIZ	MIZ/SP-RING zinc finger	0.8	5	6	0	33	5	
PF12874	zf-met	Zinc-finger of C2H2 type	0.8	4	5	2	4	12	
PF02176	zf-TRAF	TRAF-type zinc finger	0.8	7	9	1	11	0	
PF10601	zf-LITAF-like	LITAF-like zinc ribbon domain	0.6	18	31	7	65	8	
PF00641	zf-RanBP	Zn-finger in Ran binding protein and others	0.6	4	7	2	17	13	
PF05495	zf-CHY	CHY zinc finger	0.5	2	4	2	9	4	
PF05207	zf-CSL	CSL zinc finger	0.5	1	2	1	1	0	
PF00098	zf-CCHC	Zinc knuckle	0.4	12	28	9	31	90	
PF02207	zf-UBR	Putative zinc finger in N-recognin (UBR box)	0.3	5	19	2	11	1	
PF13917	zf-CCHC_3	Zinc knuckle	0.3	1	4	0	2	12	
