# Supplementary material for: The Oxytricha trifallax Macronuclear Genome: A Complex Eukaryotic Genome with 16,000 Tiny Chromosomes
Source: PLoS Biol. 2013 Jan 29;11(1):e1001473. doi: 10.1371/journal.pbio.1001473 (PMC3558436; doi:10.1371/journal.pbio.1001473)
Supplement: Table S26 — RNA-seq counts for poly-adenylate binding protein domain protein genes. RNA expression values are given in normalized read counts for vegetative (“Fed”) cells and cells developing during conjugation (see Text S1: RNA-seq mapping and read counting). (RTF) [file pbio.1001473.s056.rtf]

Table S26. RNA-seq counts for poly-adenylate binding protein domain genes.

Gene name	Fed	0 hrs	10 hrs	20 hrs	40 hrs	60 hrs	Pfam domains	
Contig11.0.0.g78	6414	20384	8136	13346	5414	12158	3x RRM_1	1x PABP		
Contig11450.0.g121	82	449	323	126	64	98	1x PABP			
Contig1154.0.g31	745	917	584	426	188	467	3x RRM_1	1x PABP		
Contig14655.0.g24	2533	3423	1527	1822	677	1996	3x RRM_1	2x PABP		
Contig15848.0.g105	118	23	7551	1510	554	358	4x RRM_1	1x RRM_6	1x PABP	
Contig1722.0.0.g34	118	0	4896	924	335	345	2x RRM_1	2x RRM_5	1x PABP	
Contig21044.0.g17	4689	1676	828	705	1524	976	4x RRM_1	1x PABP		
Contig2627.0.g82	55	15	7887	1549	598	371	4x RRM_1	1x PABP	1x RRM_6	
Contig2701.0.g46	345	7163	2111	3097	1109	2338	2x RRM_1	1x PABP		
Contig2870.0.0.g75	6328	20384	7978	13139	5318	11865	3x RRM_1	1x PABP		
Contig3505.0.1.g30	0	8	81	49	62	101	2x RRM_1	3x PABP		
Contig3808.0.0.g78	2650	17705	6587	10265	4650	9213	3x RRM_1	1x PABP		
Contig387.1.g66	1341	445	426	225	208	361	3x RRM_1	1x PABP		
Contig4155.0.1.g49	3960	18361	7022	10858	4850	9816	3x RRM_1	1x PABP		
Contig556.0.1.g19	0	8	83	51	84	96	2x RRM_1	3x PABP		
Contig6492.0.g61	702	902	566	413	184	446	3x RRM_1	1x PABP		
Contig7285.0.g44	796	1057	656	462	198	481	3x RRM_1	1x PABP		
Contig750.0.g46	16	106	40	8	12	8	1x PABP			
Contig759.0.g42	710	2253	597	785	248	700	3x RRM_1	2x PABP		
Contig8086.0.g74	8	106	38	7	12	6	1x PABP			
Contig8667.0.g68	306	7118	2078	3031	1095	2324	2x RRM_1	1x PABP		
Contig8742.0.g12	0	0	5	0	123	9	3x RRM_1	1x PABP		
Contig8927.0.g11	529	785	515	304	130	303	2x RRM_1	1x RRM_6	1x PABP	
Contig964.1.g31	5787	10598	4089	6103	2118	4546	3x RRM_1	2x PABP		
