# Supplementary material for: The Oxytricha trifallax Macronuclear Genome: A Complex Eukaryotic Genome with 16,000 Tiny Chromosomes
Source: PLoS Biol. 2013 Jan 29;11(1):e1001473. doi: 10.1371/journal.pbio.1001473 (PMC3558436; doi:10.1371/journal.pbio.1001473)
Supplement: Table S27 — RNA-seq counts for replication protein A domain protein genes. RNA expression values are given in normalized read counts for vegetative (“Fed”) cells and cells developing during conjugation (see Text S1: RNA-seq mapping and read counting). (RTF) [file pbio.1001473.s057.rtf]

Table S27. RNA-seq counts for replication protein A domain-containing genes.

Gene name	Fed	0 hrs	10 hrs	20 hrs	40 hrs	60 hrs	Pfam domains	
Contig22204.0.g75	71	1196	1312	742	1047	396	1x RPA_C			
Contig13699.0.g10	0	0	0	0	115	392	2x tRNA_anti	1x Rep-A_N	1x Rep_fac-A_C	
Contig14779.0.g81	0	0	0	0	119	410	2x tRNA_anti	1x Rep-A_N	1x Rep_fac-A_C	
Contig16302.0.g4	0	0	8	0	161	70	2x tRNA_anti	1x Rep-A_N	1x Rep_fac-A_C	
Contig2420.0.g63	86	106	1148	514	646	337	2x tRNA_anti	1x Rep-A_N	1x Rep_fac-A_C	
Contig5713.0.g13	1059	181	1800	448	273	374	1x Rep_fac-A_C	1x Rep-A_N		
Contig6592.0.0.g75	0	0	53	0	235	162	3x WD40	1x Rep_fac-A_C	2x tRNA_anti	
Contig9061.0.g16	0	0	7	6	185	154	1x Rep_fac-A_C	2x tRNA_anti		
Contig9141.0.0.g79	0	0	53	0	231	161	1x Rep_fac-A_C	3x WD40	2x tRNA_anti	
Contig9380.0.g29	78	68	273	902	132	398	2x tRNA_anti	1x Rep_fac-A_C		
Contig9697.0.1.g33	0	0	1	0	83	48	1x Rep_fac-A_C			
Contig987.1.g60	0	0	60	32	219	171	1x Rep_fac-A_C			
