# Supplementary material for: The Oxytricha trifallax Macronuclear Genome: A Complex Eukaryotic Genome with 16,000 Tiny Chromosomes
Source: PLoS Biol. 2013 Jan 29;11(1):e1001473. doi: 10.1371/journal.pbio.1001473 (PMC3558436; doi:10.1371/journal.pbio.1001473)
Supplement: Table S28 — Total RNA sources for poly(A)-selected mRNA. aRiboMinus Eukaryote Kit (Invitrogen, Carlsbad, CA). (RTF) [file pbio.1001473.s058.rtf]

Table S28. Total RNA sources for poly(A)-selected mRNA.

Sample	Index and Sequence	Fragmented	RiboMinus a	
0 Hour	01_ATCACG	Covaris	No	
20 Hour	02_CGATGT	Covaris	No	
0 Hour	03_TTAGGC	None	No	
20 Hour	04_TGACCA	None	No	
0 Hour	05_ACAGTG	Covaris	Yes	
20 Hour	06_GCCAAT	Covaris	Yes	
0 Hour	07_CAGATC	None	Yes	
20 Hour	08_ACTTGA	None	Yes	
