# Supplementary material for: The Oxytricha trifallax Macronuclear Genome: A Complex Eukaryotic Genome with 16,000 Tiny Chromosomes
Source: PLoS Biol. 2013 Jan 29;11(1):e1001473. doi: 10.1371/journal.pbio.1001473 (PMC3558436; doi:10.1371/journal.pbio.1001473)
Supplement: Text S1 — Supporting Results, Materials and Methods. Contents of file: Supporting Results, p. 3. Macronuclear genome validation, p. 3. Analysis of low frequency variants, p. 4. Pfam domains detected for CEGs missing in Oxytricha, p. 5. Investigation of alternative fragmentation sites in relation to mapped RNA-seq data, p. 6. Frequent colocation of ncRNA- and protein-encoding genes, p. 8. Examination of discrepancies between predicted and experimentally determined alternative fragmentation isoforms of the highly fragmented Contig14329.0, p. 9. Telomere addition sites, p. 10. Introns and untranscribed and untranslated regions, p. 13. Gene-less contigs or nanochromosomes, p. 15. Reads containing both putative telomeric repeats are not genuine nanochromosomes, p. 16. Analysis of short protein and ncRNA-encoding nanochromosomes, p. 17. Characterization of the longest predicted Oxytricha protein, p. 21. The Oxytricha macronucleus encodes a smaller proteome than Tetrahymena, p. 22. Additional differences in the nucleic acid related predicted proteomes of ciliates, p. 24. Proliferation of nucleic-acid binding domains in Oxytricha, p. 26. Differences in the non-nucleic acid related predicted proteomes of ciliates, p. 31. Supporting Materials and Methods, p. 35. Southern analysis of alternative nanochromosome isoforms, p. 35. qPCR to estimate relative nanochromosome copy number, p. 35. Preparation of nanochromosome DNA for Sanger/454 sequencing, p. 36. Whole nanochromosome shotgun library construction, p. 37. Whole genome shotgun (1.5–35 kb) library construction and sequencing, p. 37. 454 sequencing of DNA from nanochromosome size fractions, p. 38. Whole genome shotgun (>10 kb) fosmid library construction, p. 38. Whole nanochromosome telomere-based library construction, p. 39. Illumina genomic library construction and sequencing, p. 43. Read mapping rationale, p. 43. Determination of pN/pS values, p. 45. Genome assembly validation and redundancy analysis, p. 45. Classification of stron [file pbio.1001473.s059.rtf]

Supporting Text S1: Table of Contents 
Supporting Results	3
Macronuclear genome validation	3
Analysis of low frequency variants	4
Pfam domains detected for CEGs missing in Oxytricha	5
Investigation of alternative fragmentation sites in relation to mapped RNA-seq data	6
Frequent colocation of ncRNA- and protein-encoding genes	8
Examination of discrepancies between predicted and experimentally determined alternative fragmentation isoforms of the highly fragmented Contig14329.0	9
Telomere addition sites	10
Introns and untranscribed and untranslated regions	13
Gene-less contigs or nanochromosomes	15
Reads containing both putative telomeric repeats are not genuine nanochromosomes	16
Analysis of short protein and ncRNA-encoding nanochromosomes	17
Characterization of the longest predicted Oxytricha protein	21
The Oxytricha macronucleus encodes a smaller proteome than Tetrahymena	22
Additional differences in the nucleic acid related predicted proteomes of ciliates	24
Proliferation of nucleic-acid binding domains in Oxytricha	26
Differences in the non-nucleic acid related predicted proteomes of ciliates	31
Supporting Materials and Methods	35
Southern analysis of alternative nanochromosome isoforms	35
qPCR to estimate relative nanochromosome copy number	35
Preparation of nanochromosome DNA for Sanger/454 sequencing	36
Whole nanochromosome shotgun library construction	37
Whole genome shotgun (1.5 – 35 kb) library construction and sequencing	37
454 sequencing of DNA from nanochromosome size fractions	38
Whole genome shotgun (> 10 kb) fosmid library construction	38
Whole nanochromosome telomere-based library construction	39
Illumina genomic library construction and sequencing	43
Read mapping rationale	43
Determination of pN/pS values	45
Genome assembly validation and redundancy analysis	45
Classification of strongly- and weakly-supported alternative fragmentation sites	47
Determination of sequences surrounding telomere addition sites	48
RNA isolation, NuGEN cDNA synthesis and Illumina sequencing	48
RNA-seq mapping and read counting	50
Gene prediction	52
Length determination of “untranscribed” and untranslated regions	55
Protein domain identification and GO term selection	55
tRNA searches	57
Euplotes crassus culturing, DNA isolation and preliminary macronuclear genome assembly	57
Supporting References	59


Supporting Results


 Macronuclear genome validation

We adopted a custom genome assembly validation approach to accommodate the unusual Oxytricha macronuclear genome structure, taking advantage of sequence matches to three different kinds of sequence data: (i) 454 telomeric end reads, (ii) Sanger mate pair reads, and (iii) independent assemblies of long nanochromosome fosmid libraries (see 'Methods': 'Genome assembly validation and redundancy analysis'  and Table S8-10). Since some sequence regions corresponding to the 454 telomeric end reads and Sanger mate pairs have been incorporated into our final assembly, these data sources are not completely independent sources for assembly validation, but they are nevertheless still informative. Detection of misassembles that result from incorrect assembly of repeats [1] was not a primary concern, as the Oxytricha macronuclear genome is a relatively high complexity genome [2].  
The majority (74%) of the nanochromosomes in our final genome assembly have at least one pair of matched telomeric 454 end reads (where each of the matches is ≥ 45 bp long and ≥ 85% identical; see 'Methods'). The experimental method used to obtain these telomeric end reads limits the ability to validate nanochromosomes > 5 kb long (see 'Methods'). However, many nanochromosomes > 5 kb long are fully supported by nanochromosome end-to-end Sanger mate pair matches (though the number of nanochromosomes validated this way decrease with nanochromosome length; Figure S11 and Figure S12). Nanochromosomes that were not supported by either 454 telomeric end reads or end-to-end Sanger mate pair/read matches tended to be lower copy number nanochromosomes (Figure S11B) which could indicate there were either too few reads to provide support for low copy number nanochromosomes, or possibly errors in assembly. 88% of the assembled nanochromosomes were validated by either of these two methods (Figure S11A).

We validated the longest assembled nanochromosomes (10 - 66kb) by comparing them to the assembled contigs of long nanochromosomes cloned by fosmids (see 'Methods'). 118 out of 147 contigs (80%) with fosmid contig matches matched the fosmids from end to end, typically spanning the entire contig. Of the 29 contigs which we were unable to validate by this method, 14 only had short matches (< 3 kb) and 9 matched the majority of the contig with the exception of regions < 1.5 kb from one of the two contig ends. Nanochromosome breakage and chimeric ligation during the fosmid cloning may have precluded validation of these contigs.

Nanochromosome isoforms from the most complex alternatively fragmented nanochromosome — an 8 kb region with 8 genes and multiple isoforms — were validated by Southern blotting (see 'Extensive alternative nanochromosome fragmentation').


 Analysis of low frequency variants
We see no reason to doubt the authenticity of most of the lower frequency variant/low SNP heterozygosity nanochromosome variants, based on both visual inspection of the reads mapping to these nanochromosomes, and also the fact that these nanochromosomes comprise a small percentage of all the nanochromosomes (2.6% of nanochromosomes have a mean SNP heterozygosity <0.99% and a median variant frequency of 5-10%; Figure 5B). Furthermore, there are low levels of potentially incorrectly mapped reads, e.g. 3.8% of the heterozygous matchless contigs have > 1% of their reads mapped to multiple contigs in the assembly (mean of 12%). Therefore these potentially incorrectly mapped reads only contribute a small fraction of the observed variants (and hence excluding these contigs has a negligible effect on Figure 5A and 5B). Read mismapping also does not appear to contribute significantly to these lower frequency variants as observed from the pN/pS of coding sequences from single-gene nanochromosomes. With the exclusion of a small fraction of outliers with pN/pS > 0.6, comprising 4% of nanochromosomes with low variant frequencies (which may result from incorrectly mapped and paralogous reads, or simply the higher likelihood of observing higher pN/pS values due numerical fluctuations at lower polymorphism levels), the mean pN/pS is similar for low and high frequency variants (Figure S13). For instance, pN/pS is almost identical for nanochromosomes with variants at < 10% median variant frequency (0.0745) and variants at > 30% median variant frequency (0.0744). These similar pN/pS values also imply that the low median variant frequencies observed for nanochromosomes do not result from sequencing errors or miscalling of the variants which would both elevate pN/pS for the low median frequency variant nanochromosomes relative to the high median frequency variant nanochromosomes.


Pfam domains detected for CEGs missing in Oxytricha
The following CEGs had PFAM domain matches (with the prefix “PF”) to Oxytricha proteins: KOG0122 - PF03152, KOG3237 - PF03998, KOG2606 - PF02338, KOG1816 - PF03152, KOG1468 - PF01008, KOG2638 - PF01704, KOG1760 - PF01920, KOG3479 - PF02953, KOG1733 - PF02953, KOG2531 - PF00370 and PF02782, KOG3482 - PF01423, KOG3499 - PF01781, KOG1758 - PF02823.


Investigation of alternative fragmentation sites in relation to mapped RNA-seq data
Since the complete extent of the predicted genes' UTRs are not well-defined (see 'Methods') and there may be errors in gene predictions, it is difficult to precisely determine whether alternatively fragmented sites lie within or between genes. Hence, instead of using gene predictions, we exploited expressed sequence data to determine where these sites map relative to transcribed regions. For each alternative fragmentation site, we determined whether the site is located within a transcribed region, by finding at least one RNA-seq read pair spanning the site. For strongly-supported alternative fragmentation sites determined by Illumina telomeric reads, 18% were spanned by one or more RNA-seq pairs. We saw no avoidance of alternative fragmentation of transcribed regions spanned by RNA-seq reads, (average number of Illumina telomeric reads supporting alternatively fragmented sites spanned by RNA-seq data mean of 180 (stddev 186) vs. those not spanned, mean 186 (stddev 192)). 

Approximately 78% of the alternative fragmentation sites spanned by RNA-seq reads appear to reside where they are least likely to be deleterious — within UTRs (167/213; ~81% of these sites reside in 3' UTRs (~136/167 contigs, but 3' UTRs are also longer than 5' UTRs — see 'Gene predictions'). Since it is common for transcripts to run close to and even through telomeres (see 'Gene predictions') it would be interesting to determine whether alternative fragmentation in 3' UTR regions could have any functional consequences (e.g. by affecting mRNA stability).

Twenty alternative fragmentation sites were found in predicted intronic regions, but only one of these cases (gene 1 of Contig17419.0) was spliced in our RNA-seq data. For this case, alternative fragmentation will produce a nanochromosome isoform (isoform 2) containing the last CDS of gene 1 (19 aa long), but with no start codon. This suggests that even if this region were transcribed in this isoform (isoform 2), it would not be translated (Figure S14). For some of the remaining introns without RNA-seq evidence, it appears that the gene prediction software, AUGUSTUS, may have incorrectly fused adjacent genes by predicting a linking intron, since some predicted proteins had domain architectures that have never been observed in the Pfam database.

22/26 of the alternative fragmentation sites spanned by RNA-seq reads in CDSs appear to be located close to the boundaries of predicted CDSs. This implies that alternative fragmentation in CDS regions produce nanochromosome isoforms encoding a minimal amount of CDS. 17/21 of the truncated CDSs encoded on the shorter alternative nanochromosome isoforms are directed away from the new telomere arising from alternative fragmentation, and hence they will retain the original stop codon if they are translated in the same reading frame as the untruncated form (Figure S15). It is questionable whether these truncated CDS regions are transcribed, let alone translated, since they may lack a promoter. Of the four contigs with an alternative fragmentation site predicted well within CDS-encoding regions, one (Contig395.1) has only 11 telomeric reads mapping to the alternative fragmentation site compared to an average of 675 for the two telomeric ends from the longer isoform. In this case, the shorter nanochromosome isoforms may be numerically insignificant compared to the longer isoforms that encode full-length genes. We cannot rule out that the remaining three contigs also have predicted genes that are artificially fused (from RNA-seq data alone), as may be the case for some alternative fragmentation sites in introns.
 
Frequent colocation of ncRNA- and protein-encoding genes
A careful search for ncRNAs in the Oxytricha macronuclear genome performed on a preliminary genome assembly suggested that only a small fraction of this genome encodes ncRNAs [6]. AUGUSTUS sometimes predicts protein-coding regions that overlap ncRNAs, which may inflate estimates of protein-coding genes. However this overlap is relatively small (e.g. 5/34 non-alternatively fragmented nanochromosomes encoding tRNAs have predicted CDSs that overlap tRNAs; Figure S16). Some key ncRNA-encoding nanochromosomes do not have protein gene predictions that overlap the ncRNAs, e.g. the telomerase RNA (encoded by Contig19649.0). However, additional ncRNA encoding nanochromosomes, such as those encoding the 5.8S, 28S and 18S rRNA (Contig451.1) and the 5S ncRNA (Contig14476.0 and Contig17968.0), do have predicted overlapping protein-coding genes. Such cases will need experimental confirmation in the future to refine gene predictions.

Apart from ncRNAs with putatively overlapping incorrect gene predictions, many ncRNA genes do appear to reside on nanochromosomes with genuine protein-coding genes. Since Oxytricha has relatively few putative ncRNAs, and most of these were detected by methods designed to find ncRNAs on non-protein coding nanochromosomes [6], we examined the nanochromosomal positions of tRNAs, because they are an abundant and easily identifiable without such detection biases. tRNA genes frequently reside on protein-coding nanochromosomes: 73% of non-alternatively fragmented tRNA-encoding nanochromosomes (25 of the 34) also have predicted protein-coding genes. For the developmental time points we sampled, each of the predicted protein regions has at least one pair of RNA-seq reads mapped to it, suggesting that they are transcribed. Addition of the 28 alternatively fragmented tRNA-encoding nanochromosomes to the 34 non-alternatively fragmented tRNA-encoding nanochromosomes suggests that 85% of tRNA genes potentially reside on protein-coding nanochromosomes (half of the 28 alternatively fragmented tRNA-encoding nanochromosomes also exist as isoforms encoding the tRNA alone). Other alternatively fragmented nanochromosomes encode ncRNAs, including the nanochromosome encoding the most ncRNAs (12 snoRNAs [6]; Contig8800.0 in the Oxytricha reference macronuclear genome assembly) and the 5.8S, 28S and 18S rRNA nanochromosome. It is possible that the tRNA genes may colocate with protein-coding genes more often than other classes of ncRNA), both for alternatively fragmented nanochromosomes (46% of tRNA-encoding nanochromosomes also have protein-encoding isoforms) and non-alternatively fragmented nanochromosomes (73% of non-alternatively fragmented tRNA-coding nanochromosomes have protein-coding genes). We do not expect other less highly expressed ncRNAs to be situated on nanochromosomes with protein-coding genes as often as tRNAs.


 Examination of discrepancies between predicted and experimentally determined alternative fragmentation isoforms of the highly fragmented Contig14329.0
We were unable to detect two predicted isoforms for Contig14329.0 (Figure 6) by Southern hybridization: isoform F (3.2kb) and isoform G (2.2 kb). Isoform F is predicted by a low number of 454-telomeric reads and has no Illumina telomeric reads supporting the fragmentation site between genes 5 and 6. Isoform G is not supported by Illumina telomeric reads at the fragmentation site within gene 2, and only has 5 supporting 454 telomeric reads, but, due to the windowing approach used to count reads, this number is inflated to 20 by telomeric reads from the fragmentation site just upstream of this site. Likewise, isoform H (not tested in the Southern analysis) has no supporting Illumina telomeric reads at the site of intragenic fragmentation in gene 2. Assuming that the 454 telomeric reads indicate genuine telomere addition sites, for isoforms F, G and H, the lack of supporting Illumina telomeric reads may indicate that these forms are absent in the reference JRB310 strain, but present in the complementary JRB510 strain (or the progeny of the JRB310 and JRB510 strains), since the 454 telomeric reads also contain alleles from strain JBR510 (see 'Methods').


Telomere addition sites
Substantial variation is also present at the telomere addition sites (TASs) on the ends of nanochromosomes (and may be allele-specific too) [3]. A study of smaller-scale TAS variation at the Oxytricha trifallax type locus (81-macronuclear locus) revealed considerable variation in the exact addition site (over tens of bases) for the two central “common region” nanochromosome sites, whereas the two distal, non-shared nanochromosome ends possessed only a single TAS [3]. With deep sequencing, we also discovered small-scale variation in the distal, non-alternatively fragmented TASs for this locus, with nine different sites for the “right arm” distal site (over a 20 bp span) and three different sites for the “left arm” distal site (over an 8 bp span; for the same strain (JRB310) used in the earlier study). However, in agreement with [3], there is greater positional variability for alternatively fragmented TASs (AF-TASs; we only consider sites that are ≥ 250 bp away from contig ends when assessing alternative fragmentation sites) than for non-alternatively fragmented sites (non-AF-TASs; Figure S17). The median distance from the most used telomere addition site is 0 for non-AF sites compared to 10 for AF-sites in 200 bp intervals centered on these sites. While the ends of nanochromosomes have half as much TAS variation as these AF intervals, and it is also possible that genome assembly may further reduce the amount of visible TAS variation at the ends of assembled nanochromosomes, we still think that the level of positional variability in AF-TASs is greater than that of non-AF-TASs consistent with the observations of Williams et al. 2002. Williams et al. 2002 proposed the “weak-site” model to explain this observation. 

Surprisingly, no specific cis acting motifs have been found associated with TASs in Oxytricha. Instead, a diffuse signal has been proposed [4,5] for the sequence region preceding the non-alternatively fragmented TASs. A region of at least 50 bp preceding the TAS has distinctive, periodic biased composition [4,5] (Figure S18C). These base composition biases at either end of the nanochromosomes are inverted with respect to one another, in a similar manner to terminal inverted repeats of transposons and to the nanochromosomal terminal inverted motifs in Euplotes nanochromosomes proposed to be excised by the Euplotes Tec transposons [34-37]. A very similar, distinctive pattern of biased base composition is also present in Stylonychia (Figure S19), which is more closely related to Oxytricha (at a divergence of 0.4 substitutions per 4-fold synonymous site from Oxytricha trifallax [6]), suggesting that nanochromosome ends are generated by a common molecular mechanism in both of these ciliates. A chromosome breakage signal with a similar terminal inverted motif to that of Euplotes was proposed for a nanochromosome in Stylonychia [7]. However, unlike Euplotes, the proposed motif was only found in close proximity to the telomeres at one of the two nanochromosome ends. Although inverted repeats are found in the subtelomeric regions of most Oxytricha nanochromosomes, this may also be a consequence of the AT richness of these regions; the putative breakage motif found in Stylonychia is not common to most Oxytricha nanochromosomes [8]. There is also a distinctive ~10-11 bp periodicity in the declining frequency of alternative TAS usage for the ~40 bp closest to the most common non-AF-TAS (Figure S17).

Since most AF sites are directional (giving rise to a longer isoform and a shorter isoform; see previous section) it is possible to distinguish between sequence regions that are “present” or “absent” in the shorter isoform on either side of the AF site. The present sequence regions of strongly-supported AF-TASs have the same general relation of T > A and C > G over a similar span to non-AF-TASs. For AF-TASs the same periodic signal as non-AF-TASs is observed for ~30 bp in TAS sequences inferred from reads (Figure S20B) versus those derived from contigs that mask this signal (Figure S20A). Micronuclear genome sequence data will be essential to characterize TAS regions since we were unable to obtain absent TAS regions that were reliably free of telomere-derived bases. For AF-TASs there is a gradual decline in %AT, with a corresponding rise in %GC downstream of the TAS (Figure S18B). In the absent region, over a longer range of at least 350 bp away from the AF-TAS, the T frequency is elevated relative to the A frequency (Figure S18B) — the reverse of the 350 bp AF-TAS present region (Figure S18A) — but this likely reflects coding sequence AT biases that result from an excess of genes oriented in a convergent transcriptional direction relative to the divergent transcriptional direction (see 'Gene predictions').


For both AF- and non-AF-TASs, the -1 position in the present regions is almost devoid of G (e.g. it is 0.5% and 0.2% G, for contig-derived AF- vs. non-AF-TAS sequences, respectively; Figure S20). For AF-TASs the absent region +1 base is enriched in G (e.g. 16.4% for contig-derived TAS sequences) relative to bases further away from the TAS in this region (10.8% for contig-derived TAS sequences) (Figure S20). Euplotes, which has an orthologous telomerase RNA and the same telomeric repeats, also has an extremely low G content at the same position (Figure S21). This may result from G incorporation of this base into the telomeric repeat by telomerase [3,9].

Since the long-range base composition frequencies of AF- and non-AF-TAS positions are similar, they are likely generated by the same or a closely related molecular mechanism. Both the observation of distinct base preferences between the AF- and non-AF-TAS positions closest to the TAS, as well as the greater variability in inter-positional base frequencies for non-AF-TASs than AF-TASs are consistent with either a “weak-signal” hypothesis for alternative fragmentation or the presence of a novel, but closely related alternative fragmentation signal. 

Introns and untranscribed and untranslated regions
Oxytricha's introns are intermediate in size (mean 93 bp) between those of Tetrahymena (mean 165 bp [18]) and the very short introns of Paramecium (mean 25 bp [23,24]). However, Oxytricha's intron length distribution is trimodal (Figure S22), with decreasing modes at ~38, ~53 and ~83 bp, versus a unimodal distribution in Paramecium [23] (25 bp mode) and Tetrahymena (~58 bp mode; Figure S23). Splicing signals increase in length from shorter to longer intron modes (Figure S24 and Figure S25), consistent with a general relationship between intron length and splice signal strength in metazoa and fungi [25], rather than a specific property of the three different intron length modes. Bimodal intron length distributions have been observed for other eukaryotes (e.g. [26]). One possible explanation for multimodal intron length distributions is different components of the spliceosomal machinery that could lead to different intron length preferences. Alternatively, the spliceosomal machinery may have shifted its length preference over evolution, with some introns having not yet acquired the length mutations to fit the new bi- or unimodal preferences.

Transcription units often begin and end close to telomeres (median 73 and 25 bp at 5' and 3' ends; Figure S26A,B), consistent with a smaller survey in a related stichotrich [27], Sterkiella histriomuscorum [28]. Transcription may even run into telomeres (Figure S26B). Oxytricha 3' UTRs frequently end in the subtelomeric signal region that extends ~50 bp inwards from either telomere (and is almost identical on both ends, irrespective of gene orientation) [8]. This also suggests that telomere end-binding proteins may serve as transcription terminators. 

An inverse length relationship exists for untranslated regions: 5' UTRs are shorter than 3' UTRs (median 34 vs. 78 bp; Figure S26C,D). The few predicted genes with 3' UTRs > 500bp (154 examples) or negative 3' UTR lengths (265 examples) may be incorrect gene predictions, alternative polyadenylation sites or incorrectly mapped RNA-seq data. The total landscape of noncoding DNA flanking the predicted genes (combining untranscribed, UTS, regions lacking RNA-seq reads, and predicted UTRs, which are similar length) suggests a median of 168 bp upstream and 148 bp downstream flanking DNA (mean lengths 266 and 210 bp respectively) and compact intergenic regions (mean lengths 229-381 bp, depending on gene orientation and alternative fragmentation; Table S18). The extreme minimalism of these flanking regions makes Oxytricha an attractive model for future discovery of cis-regulatory regions, particularly in phylogenetic footprinting of the Oxytricha macronuclear genome in conjunction with macronuclear genomes of related ciliates at a suitable evolutionary divergence from Oxytricha.


  Gene-less contigs or nanochromosomes
The remaining 2,063 contigs that lack gene predictions are typically short (mean length 1,711 bp for 606 two-telomere-containing contigs; 1,282 bp for all gene-less contigs) and often incomplete nanochromosomes, missing either (917) or both (545) telomeres (only 29% of this set were complete vs. 75% of the contigs with gene predictions). Only 30 of these gene-less contigs were alternatively fragmented (Figure S9). Reducing the minimum CDS gene prediction length parameter from 150 to 75 bp only predicts 11 additional genes, suggesting that this is not simply a consequence of the inability to detect genes encoding proteins < 50 aa. Just 9% (190) of these contigs had BLASTX matches with E-values ≤ 1e-5 to a predicted protein in Paramecium or Tetrahymena, and 11% (229) had BLASTX matches to the UniProt UniRef90 database (release 2011_09 [29]; E-value ≤ 1e-5) compared to 56% of contigs with predicted proteins. AUGUSTUS run with a parameter that enables partial gene predictions predicts 239 additional incomplete genes among these contigs (with a mean summed CDS length of 469 bp), missing either a stop or start codon. 55 of these incomplete predicted proteins had BLASTP matches to the UniRef90 database with E-values ≤ 1e-5.


Since BLAST may fail to detect sequence similarity when the query proteins are very short or divergent, we searched for evidence of transcription from those contigs that lack gene predictions. A substantial percentage — 39% (808 cases) — also lacked evidence of expression in the form of RNA-seq reads. In contrast, only 7% (1,424) of contigs with predicted genes had no mapped reads in our RNA-seq data. Contigs that lacked both gene predictions and expression evidence are frequently shorter (mean length 874 bp) than those with no gene predictions and expression data (mean length 1,543 bp). Similarly, contigs that have gene predictions but no expression data are shorter (mean length 1,726 bp) than those with both gene predictions and evidence of expression (mean length 3,210 bp); however shorter contigs are also less likely to have mapped RNA-seq reads. In summary, failure to predict genes, find signs of gene expression or sequence similarity to homologs may be due to failure of the methods rather than due to the absence of genes. Hence, only few if any authentic nanochromosomes appear to lack genes.
 

Reads containing both putative telomeric repeats are not genuine nanochromosomes
Since the assemblers we used have contig length cut-offs that may miss short nanochromosomes, we examined raw Illumina and Sanger reads. For both sequence sources, we detected in the total reads a tiny fraction of reads with both 5' and 3' terminal telomeric repeats in single reads. In the paired-end Illumina read data, 27 reads (including 12 paired-ends) matched the regular expression: CCCCAAAACCCC.*GGGGTTTTGGGG (0.000047% of 57 million reads). Excluding pairs of reads generated from each end of the sequence, the Illumina sequences appeared to be distinct from each other. We identified 40 two-telomere Sanger reads (including 18 pairs; out of a total 664,000 Sanger reads), using the regular expression CCCCAAAACCCC.{10,100}GGGGTTTTGGGG, which were all distinct from the Illumina reads. We also found 79 Sanger reads with the regular expression CCCCAAAACCCC.{10,500}GGGGTTTTGGG. None of these short telomere-bearing Sanger reads have statistically significant BLASTX E-values (< 0.001) unless they have BLASTN matches to protein-coding regions of longer nanochromosomes. 

One pair of Illumina reads may have as few as two nontelomeric bases (TT; the entire nanochromosome sequence is (C4A4)20C4TTG4T4G4; the only two possibly non-telomeric bases are italicized). All 17 of the reads that can be reliably mapped (BLASTN E-value < 1e-4; with their telomeres removed) to our final assembly map to the ends of the assembled nanochromosomes. Since these reads occur at extremely low abundances, and do not appear in both raw sequence data sets, they may either be telomere-capped chad left over from aberrant macronuclear genome development [12,13] or broken pieces of macronuclear DNA that were capped with telomeres. These ultra-short nanochromosomes are probably transitory, biological noise, rather than functional molecules, and, since they were detected at low copy number, they have a high probability of being lost during asexual replication due to amitosis.


Analysis of short protein and ncRNA-encoding nanochromosomes
Because the Oxytricha macronuclear genome is highly fragmented, with one or a few genes located on each nanochromosome, and because it contains relatively few introns per gene, it is well-suited to identification and characterization of both extremely small (“dwarf”) proteins and extremely large (“giant”) proteins. Excluding the possible developmental artifacts resulting in the shortest putative nanochromosome, the limited number of short nanochromosomes in the Oxytricha macronuclear genome suggests that there are relatively few small proteins and ncRNAs encoded in this genome. Size selection of sheared DNA during the Illumina library preparation is not likely to hamper our ability to detect short nanochromosomes, since the fragmented DNA was selected at approximately the observed size limit of nanochromosomes [2,10]. There are also few very long nanochromosomes implying a paucity of very long genes in this genome. 

A total of 643 proteins (2.6% of all predicted proteins) were predicted to be between 49-100 amino acids long. Only a small fraction of these proteins have detectable homologs, e.g. 19.4% of predicted proteins have BLASTP hits to the UniProt UniRef90 database (downloaded on September 21 2011), with an E-value < 1e-3. The presence of only one stop codon in the Oxytricha genetic code implies a higher probability of predicting protein-coding ORFs in the macronuclear genome in random, non-coding stretches of sequence relative to most organisms that use the canonical genetic code. Therefore, experimental data will be required to verify these predicted proteins. To examine a reliable set of small proteins, and the nanochromosomes that encode them, we focused on a set of 66 short (≤ 100 aa), well-conserved proteins with known protein homologs (Table S19). 

The shortest ncRNA-bearing nanochromosome is 540 bp excluding telomeres and encodes just one tRNA-Gln(CUG) (Contig15672.0); however, the shortest nanochromosome that has a region with substantial sequence similarity to a known protein is a mere 469 bp excluding telomeres (Contig19982.0) and encodes a 98 aa ThiS/MoaD family protein, also known as MOCS2A in humans (Pfam: PF02597 - independent E-value 8e-13; this protein has the characteristic GG C-terminal amino acids). ThiS/MoaD proteins are sulfur-carrying proteins, required for sulfur transfer during molybdopterin synthesis [11]. This protein does not appear to be encoded in the oligohymenophoreans (Table S19; see 'Differences in the non-nucleic acid related predicted proteomes of ciliates'). While this is the shortest known protein-coding nanochromosome in our assembly, it does not encode the smallest protein in the predicted Oxytricha proteome, which is rpl41,the shortest ribosomal protein at 22 amino acids. This protein is encoded on a 670 bp nanochromosome (excluding telomeres) that is an alternatively processed isoform only visible by inspection of the assembly of a longer nanochromosome (Contig22113.0; 3.1 kb) that subsumes it. RNA-seq data identify a 413 bp region of this nanochromosome corresponding to a 5' UTR (> 57 bp), a CDS (69 bp) and a 3' UTR (> 274 bp). All but one of the ten small ribosomal proteins (≤ 100 aa; Table S20) are encoded on short subsumed nanochromosome isoforms. For ribosomal proteins ≤ 100 aa long, including the tiny 22 aa rpl41 protein, the lengths of the shortest alternatively fragmented nanochromosomes that encode them are all similarly ~600-700 bp long (Table S20).

In contrast to ribosomal protein-coding nanochromosomes, 22 of 52 nanochromosomes encoding non-ribosomal proteins ≤ 100 aa long with known protein domains in Pfam are subsumed within longer nanochromosomes (Table S21). A similar fraction (46%; 42/93) of tRNA-encoding contigs were alternatively fragmented (see 'Frequent colocation of ncRNA- and protein-encoding genes'). This implies that during assembly a substantial fraction of short nanochromosomes were subsumed by larger, alternatively fragmented nanochromosome isoforms. Small nonribosomal proteins are encoded on alternatively fragmented nanochromosomes at a rate much higher than proteins in general, though less often than the small ribosomal proteins. For small nonribosomal proteins (Table S21), single-gene protein nanochromosomes and nanochromosome isoforms are a similar mean length (687 bp) to that of ribosomal proteins (638 bp), suggesting that nanochromosome length is not the only explanation for why small ribosomal protein nanochromosomes are so often alternatively fragmented. 

Ribosomal proteins are more frequently encoded on alternatively fragmented nanochromosome genes than other proteins in general. Even excluding the 10 smallest ribosomal proteins, 44% (55/125) of all ribosomal proteins are encoded on alternatively fragmented nanochromosome genes. Though ribosomal proteins are smaller than most Oxytricha proteins, with a mean length of 224 aa (excluding possible mis-predictions > 900 aa long), even after selecting proteins of similar mean length (210-230 aa; mean 219 aa; corresponding to 587 contigs) non-ribosomal proteins are encoded on alternatively fragmented nanochromosomes much less frequently, at approximately 14%, than ribosomal proteins (Pearson's chi-squared test p < 0.0001; similar results are obtained with expanded protein length ranges that maintain a similar mean protein length).

The shortest alternatively fragmented nanochromosome we assembled is 994 bp long (Contig13851.0) with an alternative fragmentation site ~180 bp from the non-alternatively fragmented telomeric end that is upstream of the transcription start site. No RNA-seq reads map to the short region upstream of the alternative fragmentation site, whereas a predicted gene downstream of this site is well-covered by mapped RNA-seq reads. While the distinction between alternative fragmentation sites and conventional TASs is arbitrary over these smaller intervals, we observe that few conventional telomere addition sites vary more than ~50 bp from the most used telomere addition site (Figure S17). It is possible that this ~180 bp alternatively fragmented region plays a role in transcription regulation, which would be the first example of transcription regulation by nanochromosome length variation. This nanochromosome encodes a single-domain, 84 amino acid protein with an EF_hand_5 domain (PF13499) comprising the entire protein, which is unusual for EF_hand_5 domain-containing proteins which typically have two or more such domains or are present with other domains. We consider this annotation credible since we identified additional small proteins in UniProt that contain just single EF_hand_5 domains, notably in ciliates (e.g. UniProt accession A09DIR9 in Paramecium and G0QT60 in Ichthyophthirius) and also the naked mole rat, Heterocephalus glaber (G5BF31), plus three other single EF_hand_5 domain paralogs in Oxytricha (Contig12072.0.g49, Contig12687.0.g83 and Contig9213.0.g19).


Characterization of the longest predicted Oxytricha protein
The human Titin gene encodes the largest known proteins to date (27,000 - 33,000 amino acids, depending on which of 8 alternatively spliced mRNAs is translated, based on UniProt) and primarily comprises extensive repeats of immunoglobulin-like and fibronectin type III (fn3; Pfam accession: PF00041) domains. On the longest side of the nanochromosome length distribution, the massive Oxytricha protein of over 21,000 amino acids encoded on 66 kb Contig7580.0 also contains extensive fibronectin type III domains, but lacks other Titin domains (e.g. the I-set domains and the C-terminal protein kinase domain) and possesses N-terminal PA14 (3×) and filamin (7×) domain repeats (PF07691 and PF00630, respectively) not present in Titin, suggesting that it is not orthologous to Titin. Filamin domains are actin binding [14], and hence it is likely that this massive Oxytricha protein serves a structural role. Two other eukaryotes possess proteins with fn3, PA14 and filamin domains: the alveolate, Perkinsus marinus (UniProt IDs: C5L2F8 and C5LIX2) and a stramenopile (a sister group to alveolates [15]) Phytophthora infestans (UniProt ID: D0N2H9). We found no convincing homologs of this protein in the Paramecium or Tetrahymena macronuclear genomes. Since this predicted Oxytricha protein does not appear to be orthologous to Titin, but is a giant protein, we have named it Jotin, after a Norse giant (jötunn). 


 The Oxytricha macronucleus encodes a smaller proteome than Tetrahymena
From our nanochromosome number estimates of ~15,600 and an average of 1.18 genes per nanochromosome (Table 2), we estimate that Oxytricha has a haploid gene complement of ~18,500. This is smaller than that of the free-living oligohymenophoreans Paramecium (40,000 [16]) and Tetrahymena (24,700 [17]), but larger than the parasitic oligohymenophorean, Ichthyophthirius multifiliis (8,100 [17]). Furthermore, Oxytricha's macronuclear genome contains few tRNA paralogs (including alleles, < 100 tRNAs were predicted) and two identical 5S rRNA paralogs (encoded on Contig14476.0 and Contig17968.0 which are 84.2% sequence identical over an upstream 278 bp from the 5S rRNAs and 100% identical over the remaining 299 bp), whereas the genomes of the three other oligohymenophoreans appear to contain multiple paralogs of these genes. Paramecium has ~200 tRNA and 26 5S rRNA genes [19,20]; Tetrahymena has ~700 tRNA and 174 5S rRNA genes [18]; and Ichthyophthirius has 144 tRNA and 13 5S rRNA genes. Therefore, even though the parasitic Ichthyophthirius has a proteome size less than half that of Oxytricha, it has considerably more paralogs of these ncRNA genes. 

Since Paramecium has undergone up to four whole genome duplications, we wanted to know if Oxytricha also has fewer paralogs of protein-coding genes than Tetrahymena. Though there may be some gene duplicates in the Tetrahymena macronuclear genome [18,21], contrary to earlier conclusions from the Paramecium macronuclear genome analysis [16], whole genome duplications were not observed in both the Tetrahymena macronuclear genome draft [18] and a more recent analysis of Tetrahymena and Ichthyophthirius that found no blocks of synteny greater than expected by chance [17]. In contrast to Paramecium and yeast [22], which show clear evidence of retention of ribosomal protein paralogs following whole genome duplication, we found little evidence of paralogy in these proteins in Tetrahymena and Oxytricha suggesting that neither species has undergone a whole genome duplication. More generally, protein clustering suggests that there is little difference in the relative levels of paralogy between Oxytricha and Tetrahymena for clusters with moderately divergent to closely related members (Figure S27). It is possible that either or both Tetrahymena and Oxytricha may have many, more anciently duplicated genes, especially since up to 40% of Tetrahymena's genome is located in duplicated blocks [21].

Even though Oxytricha undergoes more complex genome rearrangements, and requires genes for encystment/excystment the non-cyst-forming Tetrahymena would not need, several possibilities might explain Oxytricha's predicted smaller proteome, other than the explanation that Tetrahymena may simply have many tandemly duplicated genes. Consistent with small numbers of tRNA and rRNA genes in Oxytricha, the phenomenon of variable DNA amplification may make close paralogs redundant, leading to their loss from the macronuclear genome. It is also possible that gene rearrangement may either mask the assembly of similar paralogs (or “canalize” them), or select against similar paralogs whose templates could act on each other, as they do for alleles [12] since this would result in ambiguous, error-prone rearrangement.  


Additional differences in the nucleic acid related predicted proteomes of ciliates
Non-oligohymenophorean transcription initiation factors. Table S6 and Table S7 list other interesting nucleic-acid associated domains that appear to be present in Oxytricha but absent in Tetrahymena and Paramecium; with the caveat that the sensitivity of the searches (based on current Pfam domain models and HMMER3's hmmscan) will strongly influence these results. In addition, any proteins or domains that have evolved more rapidly in oligohymenophoreans would be difficult to recognize, and we would also miss genes that are micronuclear-encoded in oligohymenophoreans. Example protein domains that are potentially Oxytricha-specific include the XRCC4 domain, which is involved in nonhomologous end joining (in particular in V(D)J recombination), the Mre11_DNA_bind domain, which is involved in microhomology-based DNA joining, and the CDC27 domain, which is a subunit of DNA polymerase delta. However, there is sequence and experimental evidence for both XRCC4 [30] and Mre11_DNA_binding [31] domains in ciliates, suggesting that our HMMER3 searches were not sufficiently sensitive to detect these domains. Nine transcription initiation factor domains in Oxytricha appear to be absent from the oligohymenophoreans: TFIID_20kDa, TAFII28, Tbf5, TFIIF_beta (3 paralogs), TFIID-18kDa, TAF4, TFIIA_gamma_C, TFIIE_alpha and TFIID_90kDa. In contrast, no transcription initiation factor domains were absent in Oxytricha but present in the oligohymenophoreans. Hence that there may be fundamental differences in transcription between oligohymenophoreans and Oxytricha. We also detected some of these domains (TFIIF_beta, TFIID-18kDa, TFIIE_alpha and TFIID_90kDa) in Euplotes (however its genome assembly is incomplete).

Some putatively Oxytricha-specific genes encoding transcription initiation factors appear substantially upregulated during development in conjunction with core RNA Polymerase II subunits (i.e. Contig8748.0.g3 - TAFII28; Contig14938.0.g40 and Contig664.0.g57 - TFIIF_beta; Contig12484.0.g45/Contig16730.0.g8 - TFIID-18kDa; Contig922.1.g114 - TFIID_90kDa; Table S22). It will be interesting to determine whether any of these TFII proteins have a specific role in macronuclear genome development, rather than a more general role in mRNA synthesis during conjugation.

 TATA-binding proteins. Though they are present in both Oxytricha and oligohymenophoreans, it is possible that some of the functional differentiation among ciliate TATA-binding proteins (TBP) could be associated with their length variation. The expression patterns of the two Tetrahymena TBP paralogs (TBP - PF00352) during conjugation in the Tetrahymena functional genomics database (TetraFGD [32,33]) are distinct from those in Oxytricha: both TBP genes in Tetrahymena (TTHERM_00575350 and TTHERM_00082170) appear upregulated during conjugation. One protein localizes to both macronuclei and micronuclei during early conjugation [118]. In contrast, the expression of one of the Oxytricha TBP domain genes (Contig262.1.g32) remains relatively constant across all time points we analyzed, while the other (Contig9118.0.g56) was exclusively expressed at 40 and 60 hours. Closer inspection of the protein sequences revealed that both Tetrahymena paralogs are approximately the same length (231 and 246 aa long, while the four Paramecium TBP domain proteins vary from 180 - 208 aa) whereas the Oxytricha paralogs appear to have originated from a separate duplication and have long, very divergent (~16.7% amino acid identity) N-terminal extensions, creating unusually long 500 and 502 aa proteins, placing them among the largest 3% of TBP domain proteins in Pfam. We note that N-terminal variation of TBP domain-containing proteins often results in major functional differences [34,35], suggesting that these proteins may have important roles in regulating gene expression in Oxytricha's distinct nuclei.


 Proliferation of nucleic-acid binding domains in Oxytricha
While absence or presence of protein domains may indicate important functional differences among ciliates, lineage-specific protein domain expansions (or losses) are also relevant. We therefore searched for such expansions relative to Tetrahymena, and included Ichthyophthirius and Paramecium for comparison (Table S23 and http://trifallax.princeton.edu/cms/raw-data/gene_annotation/pfam_annotations/pfam_domain_annotation_comparison.ods/view ). The proteomes of two pathogenic alveolates provided outgroups: Perkinsus marinus, with a large proteome of 23,654 predicted proteins, and Plasmodium falciparum [49] whose small proteome contains just ~5,350 predicted proteins.

Homeodomain proteins. Nucleic acid binding domains notably differ between Oxytricha and Tetrahymena.  Homeodomain proteins have undergone the greatest relative domain expansion in Oxytricha (31 in Oxytricha vs. 1 in Tetrahymena; Pfam domain: Homeobox - PF00046). Both Oxytricha and Paramecium possess TALE homeodomain proteins [50,51] (Homeobox_KN; PF05920; 2 and 13 respectively), unlike Tetrahymena and Ichthyophthirius. Because they are transcription factors associated with development, homeodomain proteins are best studied in plants, animals and fungi, but they are also distributed among diverse protist lineages (examples include the heterokonts, e.g. Phytophthora; excavates, e.g. Trichomonas; and amoebozoa, e.g. Dictyostelium) [52,53]. Homeodomain proteins in unicellular yeast and Chlamydomonas participate in mating type determination [54-57]. Some homeodomain proteins, such as Drosophila bicoid, bind to both DNA and RNA [58,59]. Expression of at least some Oxytricha homeodomain proteins increases during sexual development (e.g. Contig18912.0.g54 and Contig13221.0.g5; Table S24), suggesting they might participate in macronuclear development.

Zinc finger proteins. Zinc finger domains represent three of the largest Oxytricha domain expansions relative to oligohymenophoreans (e.g. zf-H2C2_2: 65 in Oxytricha vs. 5 in Tetrahymena, zf-C2H2 — 40 vs. 5, and zf-B_box — 184 vs. 31). These 3 domains are also 8.1, 4, and 3.2 × more abundant, respectively, in Oxytricha's proteome than Paramecium's, despite multiple whole genome duplications in Paramecium. zf-B_box domain proteins appear the most common nucleic acid binding proteins in Oxytricha, representing its fourth most abundant protein class, after protein kinase domain proteins (814; PF00069), EF-hand domain proteins (233; PF13499) and ankyrin repeat proteins (193; PF12796). B-box zinc fingers often form large protein families in other organisms (e.g. 216 proteins in humans, and 392 in Danio rerio, as judged from the Pfam-A 26.0 database).


Oxytricha and Tetrahymena have similar numbers of proteins per domain (mean 3.7 vs. 3.9; Ichthyophthirius has a mean of 1.9 proteins per domain; Paramecium has 6.9; Perkinsus 4.14 and Plasmodium 1.2), which suggests Oxytricha has not generally undergone expansions of protein families with annotated domains. Moreover the zf-H2C2_2 and zf-C2H2 domains are often present in the same protein. Other large families of zinc finger domains in Oxytricha (Table S25) are somewhat expanded relative to Tetrahymena, (e.g. 94 zf-C3HC4_2-containing proteins vs. 58; 65 zf-DHHC proteins vs. 47, and 172 zf-RING_2 proteins vs. 129. As a consequence of such zinc finger protein expansion, Oxytricha has 61% more zinc finger domains than Tetrahymena (1154 vs. 714; Perkinsus has 749 zinc finger domains; Table S25). Like the homeodomain proteins, many of these zinc finger proteins are probably transcription factors; however zinc fingers may also act as protein- or RNA-binding domains [60]. Unlike Oxytricha, Tetrahymena has no comparable expansions of zinc finger proteins (Table S25). A large expansion of the TLD domain (PF07534) in Oxytricha relative to Tetrahymena  is also frequently associated with zinc fingers (73 zf-B_box, 13 zf-C3HC4_2, 4 zf-RING_LisH, 3 zf-C3HC4_3 proteins, and 1 zf-RING_2 protein also have this domain). While the TLD domain has no known function, a yeast screen for proteins involved in telomere capping identified a protein RTC5 (“restriction of telomere capping” protein 5) with this domain [61]. 

Excluding zinc finger domains, homeodomains, and response regulator domains (another large family of transcription-associated proteins that are elevated in Oxytricha relative to Tetrahymena) we found a modest ~26% increase in general of transcription-associated domain proteins (defined as the 103 Pfam domains that had the word “transcription” in any of the associated GO term descriptions in pfam2go.txt) (171 in Oxytricha  vs. 135 in Tetrahymena, with no more than 14 proteins per domain; proteins with multiple “transcription” domains were scored more than once). This increase in transcription-associated proteins, particularly zinc fingers, does not support the proposal that nanochromosome copy number variation might lead to a decreased need for transcription factors because DNA copy number could influence gene expression levels [62]. On the other hand, it would be consistent with a need to transcribe the entire maternal macronuclear genome during genome rearrangement [12]. Alternatively the proliferation of these proteins may be associated with the evolution of a new mode of transcription with well-defined boundaries and few long-range cis-acting effects.

Poly-adenylate binding protein domain and Pumilio family RNA binding repeat domain proteins. Two classes of RNA binding protein domains that bind the 3' UTR in other organisms are abundant in Oxytricha relative to Tetrahymena: the poly-adenylate binding protein domain (PABP - PF00658, 17 in Oxytricha vs. 2 in Tetrahymena) and the Pumilio family RNA binding repeat domain (PUF - PF00806; 12 vs. 3). Based on Pfam database, it is unusual for single-celled organisms to contain as many PABP proteins [63] as Oxytricha. A few PABP proteins (Contig2627.0.g82/Contig15848.0.g105 and Contig1722.0.0.g34) are highly upregulated during conjugation (Table S26). Though named for the Pumilio protein in Drosophila that binds to sequence elements in the 3' UTR of hunchback mRNAs [64,65] post-transcriptional control by 3' UTR binding to PUF proteins is common in other eukaryotes too [66]. The abundance of both PABP and PUF RNA-binding proteins in Oxytricha may indicate the presence of sophisticated post-transcriptional regulation machinery. 

Alba protein homologs. The RNA/DNA binding domain Alba (PF01918) is also overrepresented in Oxytricha relative to Tetrahymena (9 vs. 1 proteins; Paramecium has 17 of these proteins and Perkinsus has 8). Alba proteins are highly expressed DNA-binding proteins found in the crenarchaeon Sulfolobulus [67], and deacetylation of one of these proteins (Ssh10b) by an archaeal Sir2 homolog results in lower DNA binding affinity (Alba=”acetylation lowers binding affinity”) [68]. Alba proteins may have evolved from an RNA-binding ancestor, that also gave rise to RNase P/MRP proteins [69]. 

An Alba homolog in Stylonychia, Mdp2, co-expresses with a Piwi-like gene (Mdp1) during conjugation [70], and defines a distinct subfamily within the Alba superfamily [69]. During early conjugation (10 hours), the most abundantly transcribed gene in Oxytricha is an Alba protein (Contig20822.0.g90; not included in our Alba domain count because its independent E-value is slightly greater than the HMMER3 E-value cutoff we used). The best BLASTP match to Stylonychia Mdp2 in Oxytricha (Contig12508.0.g82; also see Genbank accession: HM582450) has no Alba domains detected by HMMER3, but both the Oxytricha protein and the Stylonychia Mdp2 have weaker Oxytricha Alba protein BLASTP matches (e.g. Contig15988.0.g66 E-value 2e-11), which suggests that Contig12508.0.g82 is also an Alba protein. An Oxytricha homolog of Mdp1 (genbank accession: AAM96947.1) is encoded by the fourth most highly transcribed gene at this time (Contig16116.0.g20; Genbank accession: AEX87959.1). Plasmodium Alba proteins bind nonspecifically to DNA and to subtelomeric and telomeric regions [71] and also to polyA and polyU ssRNAs [72].

Replication protein A homologs. The relative expansion of “Replication factor-A protein 1, N-terminal domain” (Rep-A_N) in Table S23 (found in 5 proteins in Oxytricha vs. 1 in Tetrahymena) is also noteworthy, because a Replication protein A (RPA) homolog in Tetrahymena affects telomerase processivity [73], and that there are likely interactions between telomere end-binding proteins, which have also undergone expansion, and the telomerase complex [74,75]. Searches for the Replication factor-A C terminal domain (Rep_fac-A_C) reveal 9 homologs of this protein in Oxytricha vs. 3 in Tetrahymena. In the Pfam database, some RPA-like proteins have both N- and C-terminal domains, while others have only the C-terminal domain. Many ciliate proteins also have a tRNA_anti domain (PF01366) detected. All but one (Contig5713.0.g13) of Oxytricha's RPA homologs are substantially upregulated during conjugation (Table S27). We propose there may be a diverse set of interactions among these nine RPA paralogs and some of the six TeBP-α and three TeBP-β paralogs at each of Oxytricha's several million telomeres. 


Differences in the non-nucleic acid related predicted proteomes of ciliates
While assessing genome completeness we noted that three metabolic pathways are apparently missing key enzymes in one or more ciliates: the oxidative pentose phosphate pathway (OPPP), the Molybdenum cofactor (Moco) pathway and the vitamin B12 dependent methionine synthase pathway (see 'The macronuclear genome encodes all the genes necessary for vegetative growth'). 

Missing oxidative pentose phosphate pathway enzymes. Other than the three OPPP enzymes that were not identified during screens for core eukaryotic genes (glucose-6-phosphate dehydrogenase (G6PD), gluconolactonase (6PGL) and 6-phosphogluconate dehydrogenase (6PGD), Oxytricha appears to be missing additional pentose phosphate pathway (PPP) enzymes including ribose 5-phosphate isomerase, transaldolase, ribulose-phosphate 3-epimerase. Analyses of the Ichthyophthirius macronuclear proteome revealed that it is also missing some of these OPPP enzymes, which is less remarkable because it is a parasite [17]. Oxytricha, Tetrahymena and Ichthyophthirius possess deoxyribose-phosphate aldolase, which appears to be missing in Paramecium (Figure S4). In a sister clade to the ciliates, the dinoflagellate Perkinsus marinus also appears to have lost the same three OPPP enzymes. In Apicomplexa, another sister clade, some members, such as Plasmodium falciparum and Babesia bovis, possess all three of these enzymes missing in ciliates, while others, such as Cryptosporidum parvum, may be missing more. Since Perkinsus is an early branching dinoflagellate [36], it is likely that these three enzymes were lost in a common ancestor of ciliates and dinoflagellates.

The absence of OPPP enzymes in ciliates is reminiscent of archaea, where they are normally absent [37]. Inspection of KEGG pathways [38] suggests that absence of OPPP enzymes is rare in eukaryotes, with the exception of some non-apicomplexan parasites such as Giardia lamblia and Entamoeba histolytica. The loss of OPPP enzymes is occurs variably in bacteria, more common to small genomes (e.g. Mycoplasma, Wolbachia and Rickettsia) and certain anaerobic lineages (Clostridium, Veillonella, Fusobacterium, Eubacterium). Additional nonoxidative PPP enzymes are also often absent in archaea [37], such as transaldolase, which is also missing from Oxytricha. Unlike archaea, there is no indication that the alternative, ribulose monophosphate pathway is used for pentose synthesis. Ciliates are capable of synthesizing pentoses such as ribose-5P via non-OPPP-dependent pathways [39,40], which may begin with metabolites like fructose-6P and glyceraldehyde-3P (Figure S4). 


Missing molybdenum cofactor (Moco) associated enzymes. Seven enzymes associated with Moco (synthesized from molybdopterin and molybdate) are present in Oxytricha but missing in Tetrahymena and Paramecium [41] (Table S19)., Molybdenum usage in eukaryotes is only known in Moco and this pathway is often absent in parasites [41], Tetrahymena and Paramecium have retained a single Moco biosynthesis enzyme MOCS3 (also known as “adenylyltransferase and sulfurtransferase”; TTHERM_00530210 in Tetrahymena and GSPATT00003659001 in Paramecium), which might participate in another pathway. Oxytricha, unlike the oligohymenophoreans, also possesses a Moco-requiring sulfite oxidase enzyme (Contig309.1.g98 and Contig1151.1.g16), a Moco sulfurase enzyme (MOSC domain [42]; PF03473; Contig19960.0.g50) required for Moco maturation by the addition of a sulfido group to molybenum [43,44]) and an ATP sulfurylase/APS kinase protein (PF01747/PF01583) that is capable of catalysis using molybdate instead of sulfate (Contig2176.0.g95) [45,46].

Oxytricha also has two pterin-related enzymes absent from oligohymenophoreans: a vitamin B12-dependent methionine synthase (Contig2674.0.g39) with a pterin binding domain (Pterin_bind; PF00809) and 6-pyruvoyl tetrahydropterin synthase (PTPS; Contig1486.0.g86 and Contig17109.0.g103). No other surveyed ciliates possess vitamin B12-independent methionine synthases., There is a protein in Tetrahymena with a homocysteine S-methyltransferase domain (PF02574; TTHERM_00637220) like the Oxytricha vitamin B12-dependent methionine synthase, but this protein is missing all the other conventional vitamin B12-dependent domains and also has a unique domain in Pfam (DUF3661; PF12400). Therefore, Tetrahymena, like Oxytricha, may also be capable of producing methionine from homocysteine. The vitamin B12 binding domain of methionine synthase is also present in another Oxytricha protein, Methylmalonyl-CoA mutase (MM_CoA_mutase; PF01642; in Contig7539.0.g62), absent from oligohymenophoreans. Methylenetetrahydrofolate reductase (MTHFR; PF02219) which is required to synthesize 5-methyltetrahydrofolate, and, in turn, methionine from homocysteine, is also present in Oxytricha (Contig12611.0.g72) but not oligohymenophoreans [47], and the same appears true for cob(I)yrinic acid a,c-diamide adenosyltransferase (PF01923; Contig14400.0.2.g70) which converts vitamin B12 to its active form, coenzyme B12 (Paramecium has a weak match which may be a false positive: GSPATP00010835001). Vitamin B12-using enzymes are sparsely distributed throughout the major eukaryotic groups but have been found in Perkinsus as well as stramenopiles [48]; hence the absence of these enzymes in Tetrahymena and Paramecium may reflect loss in oligohymenophoreans.
Supporting Materials and Methods

Southern analysis of alternative nanochromosome isoforms
Southern blots of Contig14329.0 are shown in Figure S5. Sucrose-purified JRB310 macronuclear DNA from the same preparation sequenced by Illumina sequencing was analysed on an ethidium bromide stained 0.3% SeaKemGold agarose gel (Lonza). DNA was depurinated in the gel (0.25% HCl 20 min; washed twice in 0.4 M NaOH for 10 min) and transferred to a Hybond XL membrane in 0.4 M NaOH using a Nytran TurboBlotter (Schleicher & Schuell). Labelled probe was generated by means of random priming (Prime-It, Stratagene) of the Oxytricha trifallax JRB310 PCR products (gel purified) corresponding to two sets of primers (see caption of Figure S5 for the primer sequences). After overnight hybridization at 60 °C in Church's buffer (0.5 M NaPO4, pH 7.2, 1% BSA, 1 mM EDTA, 7% SDS) the membrane was washed twice in 0.2×SSC with 0.1% SDS (30 min, 60 °C).


qPCR to estimate relative nanochromosome copy number
Quantitative PCR (qPCR) was performed on the same JRB310 macronuclear DNA preparation used for Illumina sequencing. Primers were designed for five different nanochromosomes chosen to span copy number variation as assessed by mapped Illumina reads. Sequences of the PCR primers have been provided in the caption of Figure S7. PCR was performed using Fast-Start Taq DNA Polymerase (Roche) on genomic DNA and the PCR products were purified using the Minelute PCR Purification kit (Qiagen). Five to six 10-fold serial dilutions of these purified PCR products were used as a standard in qPCR. All the reactions were performed in triplicates and the mean Ct values were used to prepare a standard curve for each set of primer pairs. 10 ng of purified macronuclear DNA was used to test the copy number of respective contigs in the genomic DNA and the Ct values were fitted on respective standard curves to obtain the relative copy number for each nanochromosome.


Preparation of nanochromosome DNA for Sanger/454 sequencing
DNA isolated from Oxytricha macronuclear preparations were treated as follows: 25 µg DNA was suspended in a final 1× concentration of end repair buffer with 6 µl end repair enzyme mix (Lucigen DNA Terminator, Madison WI) and incubated at room temperature (~20 ºC) for 30 minutes followed by an inactivation step at 65 ºC for 20 minutes. In all, 1,200 µg of end-repaired nanochromosomes were size selected using six 0.7% Bio-Rad Low Melt Agarose /1× TAE gels. DNA was excised from agarose gels after running 12 hours at 50V and staining with SYBR Green I nucleic acid gel stain. To excise DNA fractions, the gel was visualized using a dark reader and DNA size fractions of 0.25 – 1.5 kb, 1.5 – 4.0 kb, 4 – 10 kb, and 10 – 35 kb were isolated. Each gel slice was diced, weighed, and melted in an equal volume of 1× TAE buffer at 65 ºC. The gel solutions were then cooled to 37 ºC and then treated with AgarAce (0.25 – 0.5U) overnight at 37 ºC.  AgarAce-treated gel solutions were extracted with an equal volume of water-saturated phenol in which the aqueous phase was recovered and reduced by 2-butanol (Sigma-Aldrich) reduction to ~ 400 µl for each isolated DNA fraction. DNA was ethanol precipitated, washed with 75% ethanol, air-dried, and suspended in 200 µl in TE buffer (10mM Tris:1mM EDTA; pH 8.0).


Whole nanochromosome shotgun library construction
We titrated the smaller whole chromosome libraries (0.25 – 1.5 kb, 1.5 – 4.0 kb, and 4 – 10 kb nanochromosome fragments) and evaluated ligation efficiencies using the CloneSmart® low and high copy transcription-free pSMART®-LCKan and pSMART®-HCKan vectors, respectively (Lucigen, Madison, WI). In short, each reaction used 2.5 µl of 4× CloneSmart® vector premix, 6.5 µl DNA solution (300 or 500 ng DNA mixed with nuclease-free water), and 1.0 µl CloneSmart® DNA Ligase was incubated at room temperature for 30 minutes and then the ligase was heat-inactivated at 70 ºC for 15 minutes. Ligations were dialyzed against water. Ligated DNA, electro-competent cells (Lucigen E. cloni 10G), and electroporation cuvettes (1.0 mm) were chilled on ice for 30 minutes. For each electroporation, 25 µl cells were mixed with 1 µl of each ligation product and electroporated using the BioRad Gene Pulser Xcell with the following settings: 10 µF, 600 ohms, and 1.8 kV. Cells were recovered in 975 µl Recovery Medium (Lucigen part no. 98226-2), transferred to a fresh tube and incubated at 37 ºC for one hour while shaking. Each electroporation outgrowth was plated on 2× YT Kanamycin (40 µg/ml) plates and incubated overnight at 37 ºC.


Whole genome shotgun (1.5 – 35 kb) library construction and sequencing
For whole genome shotgun sequencing, the isolated nanochromosome DNA was sheared using the GeneMachine® HydroShear® DNA Shearing Device (Genomic Instrumentation Services, Inc. Foster City, CA).  ~20 µg DNA of the 1.5 – 4.0 kb fraction we had previously isolated was sheared using the standard orifice, so that the resulting fragment size distribution was 1.0 – 2.0 kb. ~50 µg DNA of the 4.0 – 10.0 kb fraction and 10-35 kb fraction previously isolated was sheared using the standard orifice to generate a 2.0 – 4.0 kb fragment distribution. Each sheared DNA population was subcloned (Table S9) and paired-end sequenced per Genome Institute library and Sanger sequencing protocols respectively. This Sanger sequence data can be downloaded from ftp://ftp-private.ncbi.nlm.nih.gov/pub/TraceDB/oxytricha_trifallax/.


454 sequencing of DNA from nanochromosome size fractions
In addition to Sanger sequencing, we collected additional nanochromosomes from the 0.25-4.0 kb, 4.0-10.0 kb, 10-35 kb, and >7 kb fractions (Table S10). DNA from each fraction was isolated as described in the Whole Chromosome Shotgun methods. DNA was processed for 454 sequencing according to standard methods [76]. 454 sequence data can be downloaded from the NCBI short read archive at https://www.ncbi.nlm.nih.gov/sra/SRX000032 .


Whole genome shotgun (> 10 kb) fosmid library construction
Approximately 20 µg genomic DNA was sheared (Gene Machines Hydroshear, large orifice, speed code 22, 10 passes, retention time of 20) and end-repaired according to the CopyControl Fosmid Library Production Kit (Epicentre, Madison WI). End-repaired DNA was resolved by pulse-field gel electrophoresis using the CHEF Mapper XA Pulsed Field Electrophoresis System (Bio-Rad). The gel-run parameters were as follows: 1% GTG agarose/0.5× TBE buffer, 6 V/cm, included angle, 120°; initial switch time, 0.5 sec.; final switch time, 1.5 sec.; run time, 11 h; ramping, linear; temperature, 14°C.  DNA fragments migrating to 10 – 50 kb in length were excised and purified after a second size selection in 1% low-melting point (LMP) agarose (Bio-Rad). The second size selection gel was run at 30 V for 16 h and stained with SYBR-Green. DNA fragments were visualized with a blue light, excised, melted at 65 °C, and treated with AgarAce (Promega, 1.5 U per 100 mg agarose) in 0.5× TBE buffer at 42°C for 1 h. AgarAce treatment was followed by a single phenol extraction, and the DNA solution was minimized using sec-butanol and ethanol precipitated with 0.1 M NaCl. DNA was suspended in 10 μL of molecular biology grade H20 (Sigma). DNA was desalted by drop dialysis (MF-Millipore 0.025 μm pore-size membrane filters) and ligated to pCC1FOS™ DNA. Fosmid clones were packaged using MaxPlax λ packaging extract according to manufacturer's instructions. Packaged fosmid clones were stored at 4 °C over chloroform in 1 ml of phage dilution buffer (10 mM Tris-HCl at pH 8.3, 100 mM NaCl, 10 mM MgCl2). Transductants were isolated on LB agar plates supplemented with 25 μg/mL chloramphenicol (Cm25) and S-GAL (Sigma). Clones were picked into shallow growth plates with 240 μL of TB, Cm25, supplemented with 8% glycerol and grown at 37 °C overnight while shaking. Overnight growth was sub-cultured into 1.2 mL of LB, Cm25 and incubated overnight at 37 °C. Glycerol stocks were stored at –80 °C. DNA isolated from fosmid clones (353) was sheared, subcloned and paired-end sequenced per Genome Institute Sanger sequencing protocols. Fosmid data can be downloaded from ftp://ftp-private.ncbi.nlm.nih.gov/pub/TraceDB/oxytricha_trifallax/. 


Whole nanochromosome telomere-based library construction
Isolated Oxytricha macronuclear DNA was treated with 20 Units RNAse I (Lucigen, Madison WI) for 30 min at 37 ºC followed by a 15 min heat inactivation step at 70 ºC. The RNAse-treated DNA was ethanol precipitated by adding 15 µg GlycoBlue™ (Ambion), 1/10th volume 3M NaOAc and three volumes 100% ethanol.  The DNA was suspended in 200 µl 10mM Tris-HCl pH 7.6 (EB). In total, 118 µg of DNA was size-selected by agarose gel electrophoresis using a 1% LE/1× TAE gel (20 cm), and the DNA was electrophoresed at 90 V for 3 hours. The 1-2 kb, 2-3 kb, and 3-5 kb (Table S10) telomere-containing fractions were excised and recovered following Qiagen's QIAquick Gel Extraction Kit protocol. For each sample, we used three QIAquick columns and eluted in 40µl EB recovering 120 µl size-selected DNA fragments.  DNA yields for reach fraction were: 1-2 kb – 5.1 µg; 2-3 kb – 5.6 µg; and 3-5 kb – 6.7 µg.

To sequence from the ends of nanochromosomes we synthesized a unique duplex telomere cap adapter (Integrated DNA Technologies, Coralville, IA) specific to the single-stranded Oxytricha trifallax telomeric sequence GGGGTTTT (Figure S28). The oligonucleotide sequences were Cap Top 5' AAGCGTGGCAGCCCCCAAAACCCCAAAA and Cap Bottom 5'-pGCTGCCACGCTTAC (where p incorporates a 5' phosphate group; the duplex structure is shown in Figure S28).

The ligation reactions for each of the three fragment sizes were performed in a total volume of 300 µl using 5-6 µg of size-selected DNA, 2× Quick Ligase Buffer (New England Biolabs, Beverly, MA), 1 µM telomere cap adapters, and 7.5 µl Quick Ligase (NEB). The reaction was incubated 15 min at room temperature, and the ligation products were purified following the Qiagen QIAQuick column purification methods. Each ligation was eluted twice with 30 µl EB and then prepared for circularization. 

Taking advantage of the 3' CA dinucleotide overhang present in chromosomal fragments containing proximal and distal telomere cap adapters, we performed a second ligation using an internal adapter containing a biotinylated thymine residue (Figure S28) and an engineered 3' gap to prime nick translation through the telomeric sequence.

Each circularization ligation used 1 µg of telomere cap-adapted DNA, 1× Quick Ligase buffer, and 12.5 µl Quick ligase in a 500 µl volume. For the 1-2 kb fraction, we performed three reactions using 1.5 µl of 2 µM of internal adapter, the 2-3 kb fraction included 0.9 µl of 2 µM internal adapter in five ligations reactions, and for the 3-5 kb fraction there was sufficient material for three ligations using 0.5 µl of the 2 µM internal adapter. Each reaction was incubated for 15 min at room temperature followed by QiaQuick column purification and elution in 60 µl EB. DNA fragments that did not circularize were treated with ATP-dependent DNAse Plasmid-Safe™ (EpiCentre, Madison WI) to digest away residual linear dsDNA. Each 100 µl reaction included 1.25 mM ATP, 1× Plasmid-Safe™ buffer, and 10 U Plasmid-Safe™ enzyme. The reactions were incubated at 37 ºC for 40 min, and the circularized macronuclear chromosomes were purified using QiaQuick spin columns and eluted in 60 µl EB. The total DNA recoveries are based on NanoDrop 10000 spectrophotometer readings. Starting with 3 µg, 5 µg, and 3 µg DNA for the 1 – 2 kb, 2 – 3 kb, and 3 – 5 kb DNA inputs, respectively, we recovered 240 ng, 1030 ng, and 258 ng of DNA post Plasmid-Safe™ treatment.  

Next, we performed nick translation using 200 ng DNA. Thus, we performed a single reaction for the 1 – 2 kb and 3 – 5 kb fractions, and five reactions for the 2-3 kb fraction.  For each reaction, we added 1/10th volume NEB buffer 2, 1 mM dNTPs, 20U DNA Polymerase I (New England Biolabs, Beverly MA) and incubated the reactions for 45 min in an ice water bath.  Each reaction was purified using QiaQuick spin columns and eluted in 60 µl EB. Samples were then treated using 500 U S1 nuclease (Invitrogen, Carlsbad CA) in 1× buffer and 300 mM NaCl for 15 min at 37 ºC. The digestion products were purified using QiaQuick spin columns and eluted in 60 µl EB. Prior to DNA immobilization, we performed DNA end-repair using Lucigen's DNATerminator® End Repair Kit (Lucigen, Madison WI) per the manufacturers instructions, QiaQuick purified the DNA, and eluted in 50 µl EB.

For library immobilization, 25 µl of M270 streptavidin-coated paramagnetic partices (Dynal, UK) were washed with 50 µl 2× binding buffer (BB) and suspended in 50 µl fresh 2× BB in which 50 µl of the purified nick-translated macronuclear chromosomes were immobilized by the biotinylated thymine residue present within the internal adapter. The M270 particle/DNA mixture was incubated at room temperature for 15 min while gently rocking. The immobilized DNA fragments were washed thrice with 500 µl TE buffer and suspended in 50 µl of the 454 Titanium paired-end adapter ligation mix (1× Quick Ligase Buffer, 5 µl adapter mix (454/Roche, Branford CT), and 5 µl Quick Ligase). The ligation was carried out for 15 min at room temperature, and the adapter-ligated DNA products were washed thrice with 500 µl TE buffer. Following the 454 library protocol, we performed the adapter fill-in reaction by adding the master mix directly to the immobilized adapter-ligated DNA fragments. The 50 µl master mix included 1× ThermoPol Buffer (NEB), 400 µM dNTPs, and 24 U Bst DNA polymerase (NEB).  The reaction was incubated for 15 min at 50 ºC, washed thrice with 500 µl TE buffer, and suspended in 20 µl EB. 

For 454 sequencing, library amplification only used 10 µl of the immobilized library fragments and was followed by the 454 3 kb paired-end sequencing protocol [77]. Processed data with the internal adaptors removed, thereby splitting out the two telomeric ends can be downloaded at: http://trifallax.princeton.edu/cms/raw-data/genome/mac/reads/454-seq/processed_454_telomeric_end_reads/.


Illumina genomic library construction and sequencing
For single-end sequencing of Oxytricha macronuclear DNA, 2.25 µg of genomic DNA was sheared with a nebulizer for 6 minutes at 42 PSI. Sheared DNA was processed with Illumina's Genomic DNA Sample Prep Kit per manufacturer's instructions. DNA was sequenced at 6 pM for 1 lane and 7 pM for 3 lanes, on the GAIIX with Illumina's Single Read Cluster Generation kit V4 and Sequencing kit V4 (101 bp). For paired-end sequencing of Oxytricha macronuclear DNA, 5 µg of genomic DNA was sheared with a nebulizer for 6 minutes at 42 PSI. Sheared DNA was processed with Illumina's Paired-end DNA Sample Prep Kit per manufacturer's instructions. Invitrogen's Egel system was used for size selection of a 500 bp band. The sample was sequenced at 5 pM on HiSeq 2000, with Illumina's HiSeq Paired-end Cluster Generation kit and HiSeq sequencing kit (104 bp). Illumina sequence data can be downloaded from http://www.ncbi.nlm.nih.gov/sra/SRX190400 (paired end) and http://www.ncbi.nlm.nih.gov/sra/SRX190401 (single end).


Read mapping rationale
To map Illumina reads to our final assembly, we used gmapper version 2.1.1b in single-end mapping mode with default parameters. Since we wanted to map the majority of reads from both possible alleles, while restricting the quantity of incorrectly mapped reads from paralogs, we selected paired mapped reads where either of the members of the pair were ≥ 94% identical to the contig consensus to which they mapped and single mapped reads that were ≥ 94% identical to the contig consensus. The total number of mapped reads is reduced by 1% from the default mapped reads by applying this cutoff. A trade-off of this cutoff is that it may eliminate genuine, allelic reads with higher numbers of polymorphisms. The cutoff was chosen after examining the number of mapped positions with at least 3 different bases among the reads, since this provides a lower bound on potential incorrect read mapping. Sites were defined as potentially incorrectly mapped if at least 3 of the bases each occurred at a frequency of > 5%, the least of which was represented by ≥ 2 reads, for positions with ≥ 20× read coverage. 18.0% of the contigs had potentially incorrectly mapped sites before the identity cutoff was applied (with a median of 5 such sites per contig; the distribution is right-skewed with a mode at 1 site per contig), while 9.5% of the contigs had these potentially incorrectly mapped sites with the ≥ 94% identity cutoff (with a median of 2 incorrectly mapped sites per contig for contigs with these sites). Given their relatively low frequency, some of these sites may result from sequencing errors rather than incorrectly mapped reads (e.g. excluding sites with < 5 reads supporting the minor third base decreases the potentially incorrectly mapped sites to 6.8% of contigs). The estimated mean SNP heterozygosity of heterozygous nanochromosomes decreases substantially from 4.9% (stddev 3.2%) to 4.0% (stddev 1.8%) by applying the identity cutoff to the mapped reads (see 'Extensive genome homozygosity and high SNP heterozygosity').


Determination of pN/pS values
Predicted coding sequences for nanochromosomes without non-self BLAT matches with the major/minor variants determined from the VarScan output were aligned to determine pN/pS values of coding sequences of single-gene nanochromosomes. pN/pS values were estimated by codeml, from the PAML package (version 4.4) [79], with the default parameter file (codeml.ctl) for coding sequences (seqtype=2) in pairwise mode (runmode=-2) and with the ciliate genetic code icode=5.


Genome assembly validation and redundancy analysis
We verified our final genome assembly with three different data sources: (i) 454 telomeric end reads; (ii) Sanger mate pair reads; and (iii) assemblies of Sanger-sequenced fosmids.

454 telomeric end reads were used to verify the ends of nanochromosomes, but were only useful for nanochromosomes < 5 kb long due to the size limit of the libraries (1-2, 2-3, 3-5 kb libraries). The telomeric ends of nanochromosomes were considered verified when we counted at least one paired read match to each of the ends of the nanochromosome. Ends were mapped with lastal (“-u 3”) from LAST [81,82] (v159) after masking the genome telomeric ends prior to creating a LAST Oxytricha genome database. LAST matches were counted when they were at ≥ 85% identical and ≥ 45 bp long. The relaxed identity threshold was chosen to permit mapping of reads from any of the 4 possible alleles and to accommodate the high error rate of 454 reads. Only matches that were no more than 120 bp from the ends of contigs were counted, to accommodate variation in telomere lengths and telomere addition location.

We also used Sanger reads to validate our contig assemblies, by determining whether there were overlaps (≥ 100 bp long) between mate pair outer “spans” (the regions between the ends of the mate pair reads) and single Sanger reads that could be chained from one end of the contig to the other, with the read ends ending no more than 50 bp from either end of the contig (Figure S29). To map Sanger reads we used gmapper (version 2.0.2) in default, single-end mode, followed by filtering out paired reads where one of the matching reads was < 90% identical to the contig and single reads where the matching read was < 90% identical to the contig.

Fosmid clone contigs (assembled by PCAP with standard parameters) were used to validate the final assemblies of longer contigs (> 10 kb). None of the raw fosmid sequence data (~150,000 reads) used to assemble these contigs was incorporated into our assemblies, but 700 fosmid reads (starting with the library prefix OXAP) were incorporated into the TGI 2.1.8 assembly. We determined whether there were end-to-end BLASTN (version 2.2.25+, with default parameters and masking turned off) matches (≥ 100 bp long and ≥ 90% identical) overlapping by ≥ 100 bp. Contigs were considered to be validated if the end-to-end matches were no more than 120 bp from either end of the contig. 
To analyze redundancy within our genome assemblies we determined the number of non-self matching contigs per assembly contig in each of the assemblies. BLAT matches that were ≥ 100 bp long (shorter matches will result in excessive matches due to the inclusion of telomeric repeats) and 90-99% identical were selected for analysis. We only counted matches for contigs that were ≥ 200 bp long (since shorter contigs are typically assembly “chaff”).


Classification of strongly- and weakly-supported alternative fragmentation sites
The number of supporting reads per alternative fragmentation site varies by orders of magnitude (Figure S30A and Figure S30B). Many sites have few reads supporting the fragmentation site suggesting that they are weakly fragmented: for Illumina telomeric reads, from 4392 fragmentation sites (ignoring site orientation relative to contig orientation) 881 sites (20%) have just a single supporting read. Furthermore the correspondence between alternative fragmentation sites predicted by 454- and Illumina telomeric reads is low when the number of supporting reads is low (e.g. ~13% of the predicted sites supported by single Illumina telomeric reads were also predicted by the 454 telomeric reads (sites were considered to be supported if the site predicted by 454 telomeric reads was within 50 bp of the site predicted by Illumina telomeric reads)). 

Given the variation in the number of supporting reads per fragmentation site, we categorized each fragmentation site as strongly-supported, if there were ≥ 10 supporting Illumina telomeric reads for the site, and weakly-supported when there were < 10 supporting Illumina telomeric reads. From the Illumina telomeric reads we estimate that 9.5% (1622/17109) of nanochromosomes are alternatively fragmented with strongly-supported sites. Since the mean number of supporting 454 telomeric reads per site predicted from 454 telomeric reads is 26% that of Illumina telomeric reads per site predicted from Illumina telomeric reads (23.4 (454 telomeric reads per site) / 89.2 (Illumina reads per site)), we consider strongly-supported sites from 454 telomeric reads to be sites with ≥ 4 454 telomeric reads per site (and weakly-supported sites are those with < 4 such reads per site), which yields a similar estimate of 10% of nanochromosomes (1696/17109) being alternatively fragmented.


Determination of sequences surrounding telomere addition sites
Two methods were used to obtain sequences surrounding telomere addition sites (TASs). The first method produced contig-derived TAS sequences, by extracting sequences from the contig consensus up- or downstream of the 3' TAS (with telomeres masked by the regular expression GGGGTTTTGGGG[GT]*) identified by each mapped read within a 50 bp window surrounding the previously identified alternative fragmentation sites. The second method only considered TASs more than 400 bp away from the nanochromosome ends for alternatively fragmented sites, and TASs less than 200 bp away from the nanochromosome ends for non-alternatively fragmented sites. The second method produced read-derived TAS sequences by extracting sequences from mapped reads preceding the most commonly used TASs around the fragmentation sites we had previously determined.


RNA isolation, NuGEN cDNA synthesis and Illumina sequencing
Briefly, the events occurring, after equal mixing of the JRB310 and JRB510 cells, at the specific developmental time points we isolated RNA from Oxytricha cells are:
¥	0 hrs: food shortage (not starving, not inducing cysts, cells prepared for conjugation); or "induced for mating"
¥	10 hrs: ~10% paired and very early meiosis. The rest are pre-pairing.
¥	20 hrs: increased numbers of pairs and early meiosis. ~5% very early anlage (pre-polytene)
¥	40 hrs: the majority of cells are in the polytene stage of anlagen development.
¥	60 hrs: the majority of cells are in the DNA poor stage of anlagen development.

First RNA-seq libraries. We generated additional expression data using total RNA isolated from the 0 and 20 hr mating series (August 2010) with a TRIzol extraction kit (Invitrogen). We assayed the isolated RNA using the Agilent Bioanalyzer RNA Nano chips and found the samples to be partially degraded with RIN (RNA integrity) values of 5.7 and 4.0 for the 0 and 20 hr isolation, respectively. In an attempt to remove the lower molecular fragments, we treated 25 µl of each sample with 1.8× volumes of Agencourt® RNACLEAN® XP beads per the manufacturers protocol and eluted the RNA in 30 µl nuclease-free water (Agencourt Bioscience Corp., Beverly, MA).  In addition, we evaluated ribosome depletion methods. 500 ng of RNACLEAN RNA was mixed with 5 µl each Prokaryote (P/N 46-7777) and RiboMinus™ Eukaryote Kit probes and followed the manufacturers instructions (Invitrogen, Carlsbad, CA). We used the Ovation® RNA-Seq System (NuGEN Technologies, Inc. San Carlos, CA) and converted 100 ng of total RNA into cDNA per the Ovation protocol. For subsequent Illumina library construction, 500 ng cDNA was used as input and followed the Illumina three primer indexing protocol with minor fragmentation modifications. Libraries were constructed from either non-fragmented (to represent the low molecular weight population) or fragmented cDNA. Fragmentation utilized the Covaris MiniTube system with the following conditions: Duty Cycle: 20%, Intensity: 5, Cycles/burst: 500, Time: 120 sec. All library ligations were PCR cycle optimized, quantified, and diluted to 2 nM stocks. The 0 hr and 20 hr libraries were pooled and sequenced on single lane of an Illumina GAIIx and HiSeq instrument. The library indices and treatments are listed in Table S28.
Second RNA-seq libraries. Poly(A) mRNA was isolated from JRB310 total RNA at the same time points: fed, 0 hr, 10 hr, 20 hr, 40 hr and 60 hr (T0-T60) per the µMACS mRNA Isolation Kit protocol and recovered in 50 µl elution buffer (Miltenyi Biotec, Germany). All of these time points had similar levels of degradation (RIN 4.0-4.9). 5 µl of the mRNA was converted into cDNA with the Ovation® RNA-Seq System (NuGEN Technologies, Inc. San Carlos, CA), and 500 ng of cDNA used in standard, non-indexed, paired-end, Illumina library construction, except that we bypassed the cDNA fragmentation due to the large amount of low molecular weight molecules. After library PCR optimization, we size-selected and removed library fragments ≤ 200 bp and ≥ 500 bp using SPRI technology. 0.7× volumes of AmpureXP beads were added to each sample resulting in binding library fragments less that 200 bp. The supernatant was added to a second tube in which 90 µl of AmpureXP beads were added and 60 µl of the bead solution (not beads) was removed. The second SPRI mixture bound library fragments less than 500 bp. The beads were washed twice with 500 µl 70% ethanol, air-dried, and the size-select library DNA was eluted in 20 µl of 10mM Tris-HCl, pH 7.6. Each library was diluted to a 2 nM stock and sequenced separately on one Illumina HiSeq lane.


RNA-seq mapping and read counting
RNA-seq data for cells collected at 0, 10, 20, 40 and 60 hr from the mating time course of JRB310 and JRB510 strains, as well as a for vegetative (fed) JRB310 cells was filtered to remove rRNA and contaminants by eliminating all reads that mapped to the Oxytricha rRNA nanochromosome, unmasked scaffolds from the Chlamydomonas reinhardtii genome (version 4.0 - http://genome.jgipsf.org/Chlre4/download/Chlre4_genomic_scaffolds.fasta.gz), and the Oxytricha mitochondrial genome (Genbank accession: JN383843), using gmapper for read mapping (ver. 2.1.1b; default parameters; [83]). 

 BLAT was used to map the rRNA/contaminant filtered reads (with the switches “-noHead -stepSize=5 -minIdentity=92”). BLAT match result files were produced in the maf file format and BLAT's own psl file format. The psl files were then filtered by the filterPSL command from the AUGUSTUS distribution with the “--paired” switch (see: http://augustus.gobics.de/binaries/readme.rnaseq.html ). maf files were converted to the SAM (Sequence Alignment/Map) format [84] by the maf-convert.py from the LAST distribution [81,82,85] and filtered to contain the same matches as those in the psl file. Since the maf-convert.py script can only produce unspliced sam formatted files, we wrote a script that associated pairs of matches to produce a 'spliced' sam file from an 'unspliced' sam file. To exclude spurious short matches that might cause false intron predictions, only associated match pairs with matches ≥ 30 bp long were retained. Non-intronic reads were discarded if the aligned region was < 50 bp long.

Mapped reads were tallied with the htseq-count program from the HTSeq package [86] and normalized by the default method of DESeq [87] to account for differences in library sizes (size factors were 0.1883470, 0.2277055, 2.4298019, 1.2311910 2.4208376 and 4.3867225 for fed, 0, 10, 20, 40 and 60 hour time points respectively). Raw and normalized counts are available from: (http://trifallax.princeton.edu/cms/raw-data/transcriptome/raw_read_counts.txt/view and http://trifallax.princeton.edu/cms/raw-data/transcriptome/normalized_read_counts.txt). To compare transcription levels of genes within developmental time points, we divided the total number of reads mapped to the genes by the length of their CDSs.
Gene prediction
To train AUGUSTUS (version 2.5.5) [88,89] we used 84 Oxytricha genes, including 24 genes deposited in Genbank and 60 genes that were manually annotated at an early stage of our genome project or from visual inspection of Exonerate alignments [90] of Paramecium protein homologs from ParameciumDB [19] (parameters: -s 300 --percent 50 --model protein2genome --geneticcode 6; Ptetraurelia_peptides_v1.56.fasta) to the Oxytricha macronuclear genome. All these genes were encoded by single-gene nanochromosomes. Since Exonerate alignments typically did not define the precise ends of genes, we completed the CDS prediction by extending the gene ends, such that: (i) each of the selected Exonerate CDSs had a single possible start codon > 20 bp downstream of its telomeric sequence, in the same reading frame as the remainder of the coding sequence of the first exon, and no more than 30 nucleotides from the start codon upstream of the Exonerate alignment; (ii) we only selected genes where the first possible stop codon downstream of the alignment if this stop codon was no more than 400 bp upstream of the telomeric sequence. We also included 25 genes from single-gene nanochromosomes with intronless genes for which we were able to obtain 3' UTR or 5' and 3' UTR information by mapped RNA-seq data, to train AUGUSTUS UTR prediction in order to incorporated mapped RNA-seq data as “hints”. According to the recommendations in the AUGUSTUS documentation, we only chose proteins that were < 70% identical to each other. The final data set of 109 genes was split into training and test data sets of 55 and 54 genes respectively. We also extracted 149 introns from complete, single-transcript nanochromosomes predicted by Cufflinks [91] (default parameters), from Oxytricha 0 hr and 20 hr RNA-seq samples, to provide additional donor/acceptor splice site training information required by AUGUSTUS.

For gene prediction, AUGUSTUS was run with the switches --UTR=on and --alternatives-from-evidence=true, with the default extrinsic evidence configuration file provided with the AUGUSTUS source code. We recompiled AUGUSTUS after decreasing the default minimum length of intron hints in the source code (extrinsicinfo.cc) from 39 to 25 bp to allow AUGUSTUS to evaluate hints for shorter Oxytricha introns. To produce exonic hints for AUGUSTUS, the filtered, BLAT mapped RNA-seq data (for 0 and 20 hr time points) was processed according to the instructions on the AUGUSTUS website (http://augustus.gobics.de/binaries/readme.rnaseq.html). Since the default AUGUSTUS RNA-seq intron extraction procedure had a high rate of extraction of dubious introns, we wrote a SAM file-based intron finding script to produce intronic hints from the BLAT output (see RNA isolation, sequencing, mapping and read counting). This script outputs putative introns if the mean (nonintronic) sequence coverage over the putative intron regions (excluding 5 bp on either end of the intron to cater for small misalignments) is ≤ 10× the intronic coverage (this excludes infrequently spliced alternatively spliced introns). Where overlapping introns were predicted for a putative intronic region, only the intron with maximal coverage was reported (this removes the majority of putative lower coverage introns, which appear to be alignment errors rather than genuine introns).

After training AUGUSTUS on this limited (bootstrap) data set, we curated 217 AUGUSTUS gene predictions to retrain AUGUSTUS (15 of which were multi-gene nanochromosomes while the rest were single gene nanochromosomes; 25 nanochromosomes contained intronless genes). All of the introns for these curate gene predictions were supported by mapped RNA-seq reads, and appeared to be complete genes encoding proteins of similar length to BLAST best hits to proteins in the Genbank NR database. As per the AUGUSTUS training instructions, only genes with protein translations that were < 70% identical to each other were selected. The final training, test, configuration, and gene prediction GFF files are available on the Oxytricha macronuclear genome server (http://trifallax.princeton.edu/cms/rawdata/geneprediction/augustus_training/ and http://trifallax.princeton.edu/cms/rawdata/gene-prediction/augustus_prediction/ ; AUGUSTUS gene prediction specificity and sensitivity results are summarized at the end of http://trifallax.princeton.edu/cms/rawdata/geneprediction/augustus_training/25_mRNAs_plus_84.gb.test.results.gff).

We have also made de novo gene predictions from the “nano-genefinder” (derived from the HMM gene model of the “nanoclassifier” [6]) for the reference Oxytricha macronuclear assembly available, though we have not used these predictions directly, since this software was previously used to predict genes in a preliminary Oxytricha assembly [6] (http://trifallax.princeton.edu/cms/raw-data/gene-prediction/nano-genefinder/ ), and since these predictions may be useful in analyzing ambiguous or potentially incorrect AUGUSTUS gene predictions. Though we used a different training data set, we found that the gene prediction sensitivity and specificity were comparable for the same test data set used to analyze AUGUSTUS's gene prediction performance. The source code for the nano-genefinder is also available (http://trifallax.princeton.edu/cms/rawdata/geneprediction/nanogenefinder/nano_genefinder.tar.gz/view ).


Length determination of untranscribed” and untranslated regions
To determine 5' transcription start sites (TSSs), we selected 703 sequences containing identifiable 5' adaptor sequences from an Oxytricha pilot EST project (from RNA obtained 0, 5, 13, 21 and 30 hours post conjugation). These sequences were generated by 5'-RLM RACE (Ambion; now Life Technologies) using the standard protocol. We mapped adaptor trimmed reads to the Oxytricha macronuclear genome with BLAT (default parameters; 672 out of 703 reads mapped). 5' untranslated region (5' UTR) and 5' “untranscribed” region (5' UTS) lengths (up to the telomere addition sites) were determined for TSS's of single-gene, non-alternatively fragmented nanochromosomes, with start codons for 5' UTRs corresponding to those from our gene predictions.

To determine 3' UTR and 3' UTS lengths we used all our contaminant filtered RNA-seq data. Since poly-A tails were hard clipped from our original mapped RNA-seq reads, we remapped the original reads with BLAT (default parameters) to determine the poly-A locations (for poly-A tracts ≥ 6 bp and 90% A that did not match the reference genome). 3' UTR and 3' UTS lengths were calculated for both the most frequently used polyadenylation sites and the polyadenylation sites closest to telomere addition sites.


Protein domain identification and GO term selection

We used hmmscan from the HMMER3.0 package [92] with the default parameters and the Pfam-A 26.0 profile HMM database (ftp://ftp.sanger.ac.uk/pub/databases/Pfam/releases/Pfam26.0) to search for protein domains in the predicted proteins from the macronuclear genomes of Paramecium (Ptetraurelia_peptides_v1.62.fasta from http://paramecium.cgm.cnrs-gif.fr/download/fasta) and Tetrahymena (tta1_oct2008_finalrelease.aa from http://ciliate.org/index.php/home/downloads), as well as all peptides ≥ 30 aa long from six frame translations of the Euplotes crassus genome assembly (search results are available at http://trifallax.princeton.edu/cms/raw-data/gene_annotation/pfam_annotations/). The reference protein data sets for Ichthyophthirius multifiliis, Perkinsus marinus and Plasmodium falciparum 3D7 were also downloaded from UniProt (Jan 2012) and were searched in the same way. To eliminate allelic redundancy prior to HMMER searches, Oxytricha proteins were first clustered by UCLUST [93] at a 90% cluster identity threshold, with the query and target alignment fractions both set to 80% coverage, and then the longest representative sequences were selected for HMMER3 searches (clustered proteins are available at: http://trifallax.princeton.edu/cms/raw-data/gene-prediction/newer_augustus_gene_prediction/oxy_fin.split.6.asm.rnaseq_all_cds.v2.fixed.uclust90.pep/view).

We searched for protein domains specific to Oxytricha, but not to Paramecium and Tetrahymena, with a custom Python script with the thresholds for considering domains set as independent E-value ≤ 0.001 and conditional E-value ≤ 0.1 for at least one of the domain matches in potentially repeated domain matches. Where domains overlapped, we selected the domain with the lowest independent E-value. GO terms were assigned to Pfam identifiers, where possible, using the pfam2go mapping between Pfam identifiers and GO terms (http://www.geneontology.org/external2go/pfam2go - 2011/12/24). We assigned all the domains which had the parent terms “nucleic acid metabolic process” (GO:0090304) in the GO biological process ontology or had the term “nucleic acid binding” (GO:0003676) in the molecular function ontology to “nucleic acid-related” proteins, using the goatools Python module (https://github.com/tanghaibao/goatools/ ) to parse the GO OBO v1.2 definition file (http://www.geneontology.org/ontology/obo_format_1_2/gene_ontology_ext.obo).

Since many Pfam domains had no GO assignment in the pfam2go mapping, we inspected the Pfam descriptions of unannotated proteins present in Oxytricha but absent in Paramecium and Tetrahymena by eye to find additional putative nucleic acid binding domains.


tRNA searches
We used tRNAscan [94] with default parameters to search for tRNAs. A complete list of putative tRNAs are available at http://trifallax.princeton.edu/cms/raw-data/gene-prediction/tRNAs/oxy_fin.split.6.asm.tRNAscan-SE .


Euplotes crassus culturing, DNA isolation and preliminary macronuclear genome assembly
DNA from a mating culture of two Euplotes crassus lines (CT5 and CT27 [95]) was isolated at a stage when most cells were estimated to be in early anlage development, and hence may contain micronuclear DNA and developing macronuclear DNA, but the assembly appears to be comprised predominantly of macronuclear contigs lacking IESs (unpublished observations). This DNA was isolated using a TRIzol extraction kit (Invitrogen) following the manufacturer's protocol, from cells cultured and harvested as previously described [96]. 

1 µg of Euplotes genomic DNA was sheared with a nebulizer for 6 minutes at 42 PSI.  Samples were processed with Illumina's Genomic DNA Sample Prep kit per manufacturer's instructions. The sample was sequenced at 7 pM for 1 lane on the GAIIX with Illumina's Single Read Cluster Generation kit v4 and Sequencing kit v4 (sequences in the fastq format are available for download at http://trifallax.princeton.edu/cms/raw-data/genome/euplotes/s_1_reads_passed_filter.fastq.gz/view ).

We assembled (17,378,411) 100 bp single-end Illumina reads, trimmed with TQS_fastq.py (with parameters: -t 10 and -c 20) with IDBA (default parameters) and then extended this assembly with the same reads, using SSAKE (v3.7) [97] in TASR mode [98] (with -t 10 -c 20 -e 33). This produced a 51 Mb assembly with ~2,500 full-length nanochromosomes (mean length of 1,264 bp) and 155,612 contigs (http://trifallax.princeton.edu/cms/raw-data/genome/euplotes/idba_fastqT10C20E33.contig/view ).
Supporting References
1. Phillippy AM, Schatz MC, Pop M (2008) Genome assembly forensics: finding the elusive mis-assembly. Genome Biol 9: R55.
2. Prescott DM (1994) The DNA of ciliated protozoa. Microbiol Rev 58: 233-267.
3. Williams KR, Doak TG, Herrick G (2002) Telomere formation on macronuclear chromosomes of Oxytricha trifallax and O. fallax: alternatively processed regions have multiple telomere addition sites. BMC Genet 3: 16.
4. Prescott DM, Dizick SJ (2000) A unique pattern of intrastrand anomalies in base composition of the DNA in hypotrichs. Nucleic Acids Res 28: 4679-4688.
5. Cavalcanti AR, Stover NA, Orecchia L, Doak TG, Landweber LF (2004) Coding properties of Oxytricha trifallax (Sterkiella histriomuscorum) macronuclear chromosomes: analysis of a pilot genome project. Chromosoma 113: 69-76.
6. Jung S, Swart EC, Minx PJ, Magrini V, Mardis ER, et al. (2011) Exploiting Oxytricha trifallax nanochromosomes to screen for non-coding RNA genes. Nucleic Acids Res 39: 7529-7547.
7. Jonsson F, Steinbruck G, Lipps HJ (2001) Both subtelomeric regions are required and sufficient for specific DNA fragmentation during macronuclear development in Stylonychia lemnae. Genome Biol 2: RESEARCH0005.
8. Cavalcanti AR, Dunn DM, Weiss R, Herrick G, Landweber LF, et al. (2004) Sequence features of Oxytricha trifallax (class Spirotrichea) macronuclear telomeric and subtelomeric sequences. Protist 155: 311-322.
9. Klobutcher LA, Gygax SE, Podoloff JD, Vermeesch JR, Price CM, et al. (1998) Conserved DNA sequences adjacent to chromosome fragmentation and telomere addition sites in Euplotes crassus. Nucleic Acids Res 26: 4230-4240.
10. Swanton MT, Heumann JM, Prescott DM (1980) Gene-sized DNA molecules of the macronuclei in three species of hypotrichs: size distributions and absence of nicks. DNA of ciliated protozoa. VIII. Chromosoma 77: 217-227.
11. Zhang W, Urban A, Mihara H, Leimkuhler S, Kurihara T, et al. (2010) IscS functions as a primary sulfur-donating enzyme by interacting specifically with MoeB and MoaD in the biosynthesis of molybdopterin in Escherichia coli. J Biol Chem 285: 2302-2308.
12. Nowacki M, Vijayan V, Zhou Y, Schotanus K, Doak TG, et al. (2008) RNA-mediated epigenetic programming of a genome-rearrangement pathway. Nature 451: 153-158.
13. Mollenbeck M, Zhou Y, Cavalcanti AR, Jonsson F, Higgins BP, et al. (2008) The pathway to detangle a scrambled gene. PLoS One 3: e2330.
14. Wang K, Singer SJ (1977) Interaction of filamin with f-actin in solution. Proc Natl Acad Sci U S A 74: 2021-2025.
15. Adl SM, Simpson AG, Farmer MA, Andersen RA, Anderson OR, et al. (2005) The new higher level classification of eukaryotes with emphasis on the taxonomy of protists. J Eukaryot Microbiol 52: 399-451.
16. Aury JM, Jaillon O, Duret L, Noel B, Jubin C, et al. (2006) Global trends of whole-genome duplications revealed by the ciliate Paramecium tetraurelia. Nature 444: 171-178.
17. Coyne RS, Hannick L, Shanmugam D, Hostetler JB, Brami D, et al. (2011) Comparative genomics of the pathogenic ciliate Ichthyophthirius multifiliis, its free-living relatives and a host species provide insights into adoption of a parasitic lifestyle and prospects for disease control. Genome Biol 12: R100.
18. Eisen JA, Coyne RS, Wu M, Wu D, Thiagarajan M, et al. (2006) Macronuclear genome sequence of the ciliate Tetrahymena thermophila, a model eukaryote. PLoS Biol 4: e286.
19. Arnaiz O, Sperling L (2011) ParameciumDB in 2011: new tools and new data for functional and comparative genomics of the model ciliate Paramecium tetraurelia. Nucleic Acids Res 39: D632-636.
20. Arnaiz O, Cain S, Cohen J, Sperling L (2007) ParameciumDB: a community resource that integrates the Paramecium tetraurelia genome sequence with genetic data. Nucleic Acids Res 35: D439-444.
21. Martens C, Vandepoele K, Van de Peer Y (2008) Whole-genome analysis reveals molecular innovations and evolutionary transitions in chromalveolate species. Proc Natl Acad Sci U S A 105: 3427-3432.
22. Kellis M, Birren BW, Lander ES (2004) Proof and evolutionary analysis of ancient genome duplication in the yeast Saccharomyces cerevisiae. Nature 428: 617-624.
23. Jaillon O, Bouhouche K, Gout JF, Aury JM, Noel B, et al. (2008) Translational control of intron splicing in eukaryotes. Nature 451: 359-362.
24. Russell CB, Fraga D, Hinrichsen RD (1994) Extremely short 20-33 nucleotide introns are the standard length in Paramecium tetraurelia. Nucleic Acids Res 22: 1221-1225.
25. Dewey CN, Rogozin IB, Koonin EV (2006) Compensatory relationship between splice sites and exonic splicing signals depending on the length of vertebrate introns. BMC Genomics 7: 311.
26. Sakharkar M, Passetti F, de Souza JE, Long M, de Souza SJ (2002) ExInt: an Exon Intron Database. Nucleic Acids Res 30: 191-194.
27. Zoller SD, Hammersmith RL, Swart EC, Higgins BP, Doak TG, et al. (2012) Characterization and Taxonomic Validity of the Ciliate Oxytricha trifallax (Class Spirotrichea) Based on Multiple Gene Sequences: Limitations in Identifying Genera Solely by Morphology. Protist.
28. Lescasse R, Yang T, Grisvard J, Villalobo E, Moch C, et al. (2005) Gene structure of the ciliate Sterkiella histriomuscorum based on a combined analysis of DNA and cDNA sequences from 21 macronuclear chromosomes. Chromosoma 114: 344-351.
29. (2011) Ongoing and future developments at the Universal Protein Resource. Nucleic Acids Res 39: D214-219.
30. Kapusta A, Matsuda A, Marmignon A, Ku M, Silve A, et al. (2011) Highly precise and developmentally programmed genome assembly in Paramecium requires ligase IV-dependent end joining. PLoS Genet 7: e1002049.
31. Paull TT, Gellert M (2000) A mechanistic basis for Mre11-directed DNA joining at microhomologies. Proc Natl Acad Sci U S A 97: 6409-6414.
32. Miao W, Xiong J, Bowen J, Wang W, Liu Y, et al. (2009) Microarray analyses of gene expression during the Tetrahymena thermophila life cycle. PLoS One 4: e4429.
33. Xiong J, Lu X, Zhou Z, Chang Y, Yuan D, et al. (2012) Transcriptome analysis of the model protozoan, Tetrahymena thermophila, using Deep RNA sequencing. PLoS One 7: e30630.
34. Koide R, Kobayashi S, Shimohata T, Ikeuchi T, Maruyama M, et al. (1999) A neurological disease caused by an expanded CAG trinucleotide repeat in the TATA-binding protein gene: a new polyglutamine disease? Hum Mol Genet 8: 2047-2053.
35. Nakamura K, Jeong SY, Uchihara T, Anno M, Nagashima K, et al. (2001) SCA17, a novel autosomal dominant cerebellar ataxia caused by an expanded polyglutamine in TATA-binding protein. Hum Mol Genet 10: 1441-1448.
36. Saldarriaga JF, McEwan ML, Fast NM, Taylor FJ, Keeling PJ (2003) Multiple protein phylogenies show that Oxyrrhis marina and Perkinsus marinus are early branches of the dinoflagellate lineage. Int J Syst Evol Microbiol 53: 355-365.
37. Soderberg T (2005) Biosynthesis of ribose-5-phosphate and erythrose-4-phosphate in archaea: a phylogenetic analysis of archaeal genomes. Archaea 1: 347-352.
38. Kanehisa M, Goto S, Sato Y, Furumichi M, Tanabe M (2012) KEGG for integration and interpretation of large-scale molecular data sets. Nucleic Acids Res 40: D109-114.
39. Borowitz MJ, Stein RB, Blum JJ (1977) Quantitative analysis of the change of metabolite fluxes along the pentose phosphate and glycolytic pathways in Tetrahymena in response to carbohydrates. J Biol Chem 252: 1589-1605.
40. Eldan M, Blum JJ (1975) Presence of nonoxidative enzymes of the pentose phosphate shunt in Tetrahymena. J Protozool 22: 145-149.
41. Zhang Y, Gladyshev VN (2008) Molybdoproteomes and evolution of molybdenum utilization. J Mol Biol 379: 881-899.
42. Anantharaman V, Aravind L (2002) MOSC domains: ancient, predicted sulfur-carrier domains, present in diverse metal-sulfur cluster biosynthesis proteins including Molybdenum cofactor sulfurases. FEMS Microbiol Lett 207: 55-61.
43. Wollers S, Heidenreich T, Zarepour M, Zachmann D, Kraft C, et al. (2008) Binding of sulfurated molybdenum cofactor to the C-terminal domain of ABA3 from Arabidopsis thaliana provides insight into the mechanism of molybdenum cofactor sulfuration. J Biol Chem 283: 9642-9650.
44. Schwarz G, Mendel RR, Ribbe MW (2009) Molybdenum cofactors, enzymes and pathways. Nature 460: 839-847.
45. Wilson LG, Bandurski RS (1958) Enzymatic reactions involving sulfate, sulfite, selenate, and molybdate. J Biol Chem 233: 975-981.
46. Reuveny Z (1977) Derepression of ATP sulfurylase by the sulfate analogs molybdate and selenate in cultured tobacco cells. Proc Natl Acad Sci U S A 74: 619-622.
47. Banerjee RV, Matthews RG (1990) Cobalamin-dependent methionine synthase. FASEB J 4: 1450-1459.
48. Zhang Y, Rodionov DA, Gelfand MS, Gladyshev VN (2009) Comparative genomic analyses of nickel, cobalt and vitamin B12 utilization. BMC Genomics 10: 78.
49. Gardner MJ, Hall N, Fung E, White O, Berriman M, et al. (2002) Genome sequence of the human malaria parasite Plasmodium falciparum. Nature 419: 498-511.
50. Bertolino E, Reimund B, Wildt-Perinic D, Clerc RG (1995) A novel homeobox protein which recognizes a TGT core and functionally interferes with a retinoid-responsive motif. J Biol Chem 270: 31178-31188.
51. Burglin TR (1997) Analysis of TALE superclass homeobox genes (MEIS, PBC, KNOX, Iroquois, TGIF) reveals a novel domain conserved between plants and animals. Nucleic Acids Res 25: 4173-4180.
52. Iyer LM, Anantharaman V, Wolf MY, Aravind L (2008) Comparative genomics of transcription factors and chromatin proteins in parasitic protists and other eukaryotes. Int J Parasitol 38: 1-31.
53. Derelle R, Lopez P, Le Guyader H, Manuel M (2007) Homeodomain proteins belong to the ancestral molecular toolkit of eukaryotes. Evol Dev 9: 212-219.
54. Shepherd JC, McGinnis W, Carrasco AE, De Robertis EM, Gehring WJ (1984) Fly and frog homoeo domains show homologies with yeast mating type regulatory proteins. Nature 310: 70-71.
55. Kurvari V, Grishin NV, Snell WJ (1998) A gamete-specific, sex-limited homeodomain protein in Chlamydomonas. J Cell Biol 143: 1971-1980.
56. Wilson NF, O'Connell JS, Lu M, Snell WJ (1999) Flagellar adhesion between mt(+) and mt(-) Chlamydomonas gametes regulates phosphorylation of the mt(+)-specific homeodomain protein GSP1. J Biol Chem 274: 34383-34388.
57. Lee JH, Lin H, Joo S, Goodenough U (2008) Early sexual origins of homeoprotein heterodimerization and evolution of the plant KNOX/BELL family. Cell 133: 829-840.
58. Dubnau J, Struhl G (1996) RNA recognition and translational regulation by a homeodomain protein. Nature 379: 694-699.
59. Rivera-Pomar R, Niessing D, Schmidt-Ott U, Gehring WJ, Jackle H (1996) RNA binding and translational suppression by bicoid. Nature 379: 746-749.
60. Font J, Mackay JP (2010) Beyond DNA: zinc finger domains as RNA-binding modules. Methods Mol Biol 649: 479-491.
61. Addinall SG, Downey M, Yu M, Zubko MK, Dewar J, et al. (2008) A genomewide suppressor and enhancer analysis of cdc13-1 reveals varied cellular processes influencing telomere capping in Saccharomyces cerevisiae. Genetics 180: 2251-2266.
62. Xu K, Doak TG, Lipps HJ, Wang J, Swart EC, et al. (2012) Copy number variations of 11 macronuclear chromosomes and their gene expression in Oxytricha trifallax. Gene.
63. Mangus DA, Evans MC, Jacobson A (2003) Poly(A)-binding proteins: multifunctional scaffolds for the post-transcriptional control of gene expression. Genome Biol 4: 223.
64. Murata Y, Wharton RP (1995) Binding of pumilio to maternal hunchback mRNA is required for posterior patterning in Drosophila embryos. Cell 80: 747-756.
65. Wharton RP, Sonoda J, Lee T, Patterson M, Murata Y (1998) The Pumilio RNA-binding domain is also a translational regulator. Mol Cell 1: 863-872.
66. Wickens M, Bernstein DS, Kimble J, Parker R (2002) A PUF family portrait: 3'UTR regulation as a way of life. Trends Genet 18: 150-157.
67. Forterre P, Confalonieri F, Knapp S (1999) Identification of the gene encoding archeal-specific DNA-binding proteins of the Sac10b family. Mol Microbiol 32: 669-670.
68. Bell SD, Botting CH, Wardleworth BN, Jackson SP, White MF (2002) The interaction of Alba, a conserved archaeal chromatin protein, with Sir2 and its regulation by acetylation. Science 296: 148-151.
69. Aravind L, Iyer LM, Anantharaman V (2003) The two faces of Alba: the evolutionary connection between proteins participating in chromatin structure and RNA metabolism. Genome Biol 4: R64.
70. Fetzer CP, Hogan DJ, Lipps HJ (2002) A PIWI homolog is one of the proteins expressed exclusively during macronuclear development in the ciliate Stylonychia lemnae. Nucleic Acids Res 30: 4380-4386.
71. Goyal M, Alam A, Iqbal MS, Dey S, Bindu S, et al. (2011) Identification and molecular characterization of an Alba-family protein from human malaria parasite Plasmodium falciparum. Nucleic Acids Res.
72. Chene A, Vembar SS, Riviere L, Lopez-Rubio JJ, Claes A, et al. (2011) PfAlbas constitute a new eukaryotic DNA/RNA-binding protein family in malaria parasites. Nucleic Acids Res.
73. Min B, Collins K (2009) An RPA-related sequence-specific DNA-binding subunit of telomerase holoenzyme is required for elongation processivity and telomere maintenance. Mol Cell 36: 609-619.
74. Xin H, Liu D, Wan M, Safari A, Kim H, et al. (2007) TPP1 is a homologue of ciliate TEBP-beta and interacts with POT1 to recruit telomerase. Nature 445: 559-562.
75. Wang F, Podell ER, Zaug AJ, Yang Y, Baciu P, et al. (2007) The POT1-TPP1 telomere complex is a telomerase processivity factor. Nature 445: 506-510.
76. Margulies M, Egholm M, Altman WE, Attiya S, Bader JS, et al. (2005) Genome sequencing in microfabricated high-density picolitre reactors. Nature 437: 376-380.
77. Jarvie T, Harkins T (2008) De novo assembly and genomic structural variation analysis with genome sequencer FLX 3K long-tag paired end reads. Biotechniques 44: 829-831.
78. Koboldt DC, Chen K, Wylie T, Larson DE, McLellan MD, et al. (2009) VarScan: variant detection in massively parallel sequencing of individual and pooled samples. Bioinformatics 25: 2283-2285.
79. Yang Z (1997) PAML: a program package for phylogenetic analysis by maximum likelihood. Comput Appl Biosci 13: 555-556.
80. Ranwez V, Harispe S, Delsuc F, Douzery EJ (2011) MACSE: Multiple Alignment of Coding SEquences Accounting for Frameshifts and Stop Codons. PLoS One 6: e22594.
81. Frith MC, Hamada M, Horton P (2010) Parameters for accurate genome alignment. BMC Bioinformatics 11: 80.
82. Kielbasa SM, Wan R, Sato K, Horton P, Frith MC (2011) Adaptive seeds tame genomic sequence comparison. Genome Res 21: 487-493.
83. David M, Dzamba M, Lister D, Ilie L, Brudno M (2011) SHRiMP2: sensitive yet practical SHort Read Mapping. Bioinformatics 27: 1011-1012.
84. Li H, Handsaker B, Wysoker A, Fennell T, Ruan J, et al. (2009) The Sequence Alignment/Map format and SAMtools. Bioinformatics 25: 2078-2079.
85. Frith MC, Wan R, Horton P (2010) Incorporating sequence quality data into alignment improves DNA read mapping. Nucleic Acids Res 38: e100.
86. Anders S (2012) HTseq: Analysing high-throughput sequencing data with python.
87. Anders S, Huber W (2010) Differential expression analysis for sequence count data. Genome Biol 11: R106.
88. Stanke M, Diekhans M, Baertsch R, Haussler D (2008) Using native and syntenically mapped cDNA alignments to improve de novo gene finding. Bioinformatics 24: 637-644.
89. Stanke M, Waack S (2003) Gene prediction with a hidden Markov model and a new intron submodel. Bioinformatics 19 Suppl 2: ii215-225.
90. Slater GS, Birney E (2005) Automated generation of heuristics for biological sequence comparison. BMC Bioinformatics 6: 31.
91. Trapnell C, Williams BA, Pertea G, Mortazavi A, Kwan G, et al. (2010) Transcript assembly and quantification by RNA-Seq reveals unannotated transcripts and isoform switching during cell differentiation. Nat Biotechnol 28: 511-515.
92. Eddy SR (2011) HMMER3.
93. Edgar RC (2010) Search and clustering orders of magnitude faster than BLAST. Bioinformatics 26: 2460-2461.
94. Lowe TM, Eddy SR (1997) tRNAscan-SE: a program for improved detection of transfer RNA genes in genomic sequence. Nucleic Acids Res 25: 955-964.
95. Jacobs ME, Sanchez-Blanco A, Katz LA, Klobutcher LA (2003) Tec3, a new developmentally eliminated DNA element in Euplotes crassus. Eukaryot Cell 2: 103-114.
96. Roth M, Lin M, Prescott DM (1985) Large scale synchronous mating and the study of macronuclear development in Euplotes crassus. J Cell Biol 101: 79-84.
97. Warren RL, Sutton GG, Jones SJ, Holt RA (2007) Assembling millions of short DNA sequences using SSAKE. Bioinformatics 23: 500-501.
98. Warren RL, Holt RA (2011) Targeted assembly of short sequence reads. PLoS One 6: e19816.

	
1
